# The Influence of the Electron Density in Acyl Protecting Groups on the Selectivity of Galactose Formation

**DOI:** 10.1021/jacs.2c05859

**Published:** 2022-10-27

**Authors:** Kim Greis, Sabrina Leichnitz, Carla Kirschbaum, Chun-Wei Chang, Mei-Huei Lin, Gerard Meijer, Gert von Helden, Peter H. Seeberger, Kevin Pagel

**Affiliations:** † Institute of Chemistry and Biochemistry, Freie Universität Berlin, 14195 Berlin, Germany; ‡ Fritz Haber Institute of the Max Planck Society, 14195 Berlin, Germany; § Max Planck Institute of Colloids and Interfaces, 14476 Potsdam, Germany

## Abstract

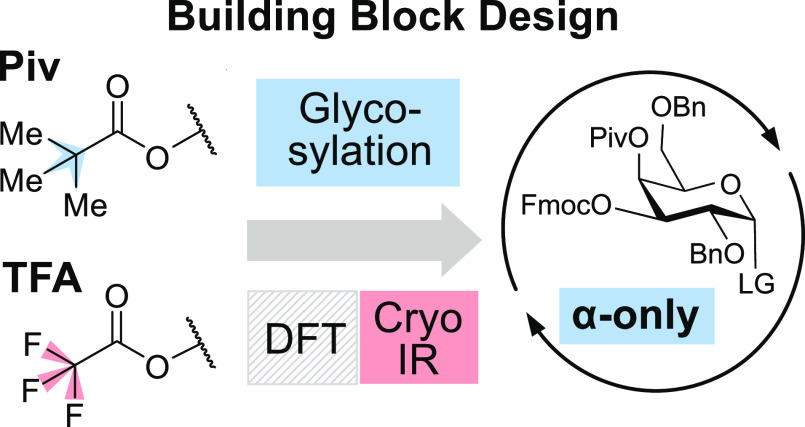

The stereoselective formation of 1,2-*cis*-glycosidic bonds is a major bottleneck in the synthesis of carbohydrates. We here investigate how the electron density in acyl protecting groups influences the stereoselectivity by fine-tuning the efficiency of remote participation. Electron-rich C4-pivaloylated galactose building blocks show an unprecedented α-selectivity. The trifluoroacetylated counterpart with electron-withdrawing groups, on the other hand, exhibits a lower selectivity. Cryogenic infrared spectroscopy in helium nanodroplets and density functional theory calculations revealed the existence of dioxolenium-type intermediates for this reaction, which suggests that remote participation of the pivaloyl protecting group is the origin of the high α-selectivity of the pivaloylated building blocks. According to these findings, an α-selective galactose building block for glycosynthesis is developed based on rational considerations and is subsequently employed in automated glycan assembly exhibiting complete stereoselectivity. Based on the obtained selectivities in the glycosylation reactions and the results from infrared spectroscopy and density functional theory, we suggest a mechanism by which these reactions could proceed.

## Introduction

The chemical synthesis of carbohydrates requires stereochemical control during glycoside formation. While neighboring-group participation is key to synthesizing 1,2-*trans* glycosides, methods to generate 1,2-*cis* glycosides are less reliable. Many biologically important oligosaccharides contain 1,2-*cis* linkages, such as the blood group systems^1^ or bacterial lipopolysaccharide antigens.^2,3^ Participation of remote acyl groups,^4−6^ chiral auxiliaries,^7^ or 4,6-benzylidene^8,9^ protecting groups helps to increase the ratio of 1,2-*cis* glycosides. Previous studies on galactose building blocks suggest that participating acetyl protecting groups at the C4 position lead to *cis*-selectivity (defined as α-selectivity for galactose).^10,11^ The remote acetyl protecting group is shielding the positive charge of the anomeric carbon by forming a temporary covalent bond that prevents nucleophiles from attacking the 1,2-*trans*-side, leading to 1,2-*cis*-selectivity. However, the ability of acetyl groups to remotely participate is limited, as the selectivity differs dramatically depending on the strength of the nucleophile. This is problematic because efficient glycan synthesis requires full stereocontrol. Total stereoselectivity is particularly important in sequential synthetic methods such as automated glycan assembly (AGA)^12^ to avoid the formation of complex mixtures of stereoisomers, which leads to a drastic drop in overall yield.

Besides high yields and an excellent stereoselectivity, differential protecting groups are a requirement for implementation in AGA. Therefore, strategies involving 4,6-*O*-di-*tert*-butylsilylene (DTBS) protecting groups, showing full α-selectivity in galactosylations,^13,14^ cannot be employed, as this protecting group would yield two nucleophilic OH groups after deprotection. Moreover, AGA requires an excess amount of promoters (NIS and TfOH); however, DTBS is labile toward such acidic conditions. While the position of the acyl protecting group and the influence of nucleophile strength have been investigated before,^10,15^ the effect of electron density on acyl protecting groups in galactosylations has been ignored.

Generally, the mechanism of glycosylation reactions is not entirely understood to date.^16,17^ It is generally accepted that the mechanism is governed by an S_N_1–S_N_2 continuum^18^ that can be shifted toward one side by adjusting various parameters. When it comes to the formation of α-selective linkages in galactose building blocks, a consistent increase in selectivity has been observed for C4-acylated building blocks.^4,10,11,15,19,20^ Strong evidence suggests that this selectivity is aided by remote participation of the C4-acyl group.^21^ On the other hand, it has been reported that the formation of β-triflates^22−24^ can lead to α-selectivity upon the attack of a nucleophile. Evidence for remote participation has been provided indirectly by bridged side products extracted from glycosylation reactions^25,26^ or directly by low-temperature NMR experiments in organic solvents^19,27^ and gas-phase infrared spectroscopy.^10,15,28,29^ It should be noted that the intermediate showing remote participation in solution can only be observed under very limited circumstances, as the lifetime of the glycosyl cation is usually shorter than the relaxation time in NMR experiments.^27^ Furthermore, glycosyl cations with remote acetyl groups were stabilized in super acids.^30^ Here, remote participation was not observed. However, all carbonyl groups are protonated, which drastically reduces their nucleophilicity. Hence, they are unable to engage in remote participation.

Here, we systematically investigate how electron-donating and electron-withdrawing substituents in acyl protecting groups influence the stereoselectivity of galactosylations. Custom-tailored galactosyl building blocks were investigated carrying pivaloyl (trimethylacetyl, **Piv**) or trifluoroacetyl (**TFA**) protecting groups at C4, C6, or both positions, while the remaining hydroxyl groups are benzylated. The building blocks (**4/6/4,6Piv** and **4/6/4,6TFA**) were assessed in glycosylation test reactions to determine their selectivity with four distinct nucleophiles. Their selectivities were compared to acetylated and benzylated building blocks **4Ac** and **4Bn**. In parallel, the glycosyl cation intermediates of the **4/6/4,6Piv** building blocks were structurally characterized using cryogenic gas-phase infrared (IR) spectroscopy in helium nanodroplets and density functional theory (DFT).^31,32^ This approach allows investigating the intermediate of S_N_1-like glycosylation reactions. Finally, the most promising building block, **4Piv**, was used in automated glycan assembly^12^ to synthesize an α(1,3)-d-trigalactopyranoside.

## Methods

The instrumental setup for gas-phase IR spectroscopy in helium nanodroplets has been described previously^33,34^ (see SI and Figure S1). Briefly, glycosyl cations are generated by nanoelectrospray ionization and subsequent in-source fragmentation of thioglycoside galactose building blocks. The mass-to-charge ratio of the generated ions can be monitored by a time-of-flight mass spectrometer. A quadrupole mass filter allows for mass-to-charge selection of the ions of interest that are then guided into a hexapole ion trap, where the ions are cooled to ca. 90 K by collisions with the helium buffer gas. A beam of superfluid helium nanodroplets (0.37 K) is generated by a pulsed Even–Lavie valve.^35^ The beam is guided through the ion trap, where the droplets pick up the ions and lead them to a detection region, where the beam of doped droplets overlaps with an IR beam generated by the tunable Fritz Haber Institute free-electron laser^36^ (FHI FEL). The interaction with resonant IR photons (1000–1800 cm^–1^) leads to the release of the probed glycosyl cations, which are subsequently detected by a second time-of-flight mass spectrometer. The ion count is plotted against the wavenumber to yield an IR spectrum. By comparison with computed harmonic frequencies, the structure of the probed ion can be determined. This approach and others based on infrared multiple photon dissociation (IRMPD) spectroscopy have successfully been applied to probe the structure of glycosyl cations exhibiting remote and neighboring group participation.^10,15,28,29,34,37−41^

For structural assignment, the experimental IR spectra are compared with theoretical spectra derived from computed structures. A genetic algorithm^42^ was employed to sample the conformational space of glycosyl cations at the PBE+vdW^TS^^43,44^ level of theory using *light* basis set settings, implemented in FHI-aims.^45^ The geometries of a subset of low-energy structures were reoptimized and their frequencies computed at the PBE0+D3/6-311+G(d,p)^46,47^ level of theory in Gaussian 16.^48^ All calculated IR spectra are normalized and scaled by an empirical factor of 0.965.^10,34^ The reoptimized geometries were used to compute accurate single-point energies at the DLPNO-CCSD(T)/Def2-TZVPP^49,50^ level of theory in ORCA.^51^ Pyranose ring puckers are assigned according to Cremer–Pople coordinates.^52^ The free energy at 90 K is used as a relevant parameter to rank the reoptimized structures. Detailed information on the computed structures, such as energetics, ring puckers, or *xyz*-coordinates, can be found in the SI.

## Results and Discussion

### Glycosylation Reactions

**Figure 1:**
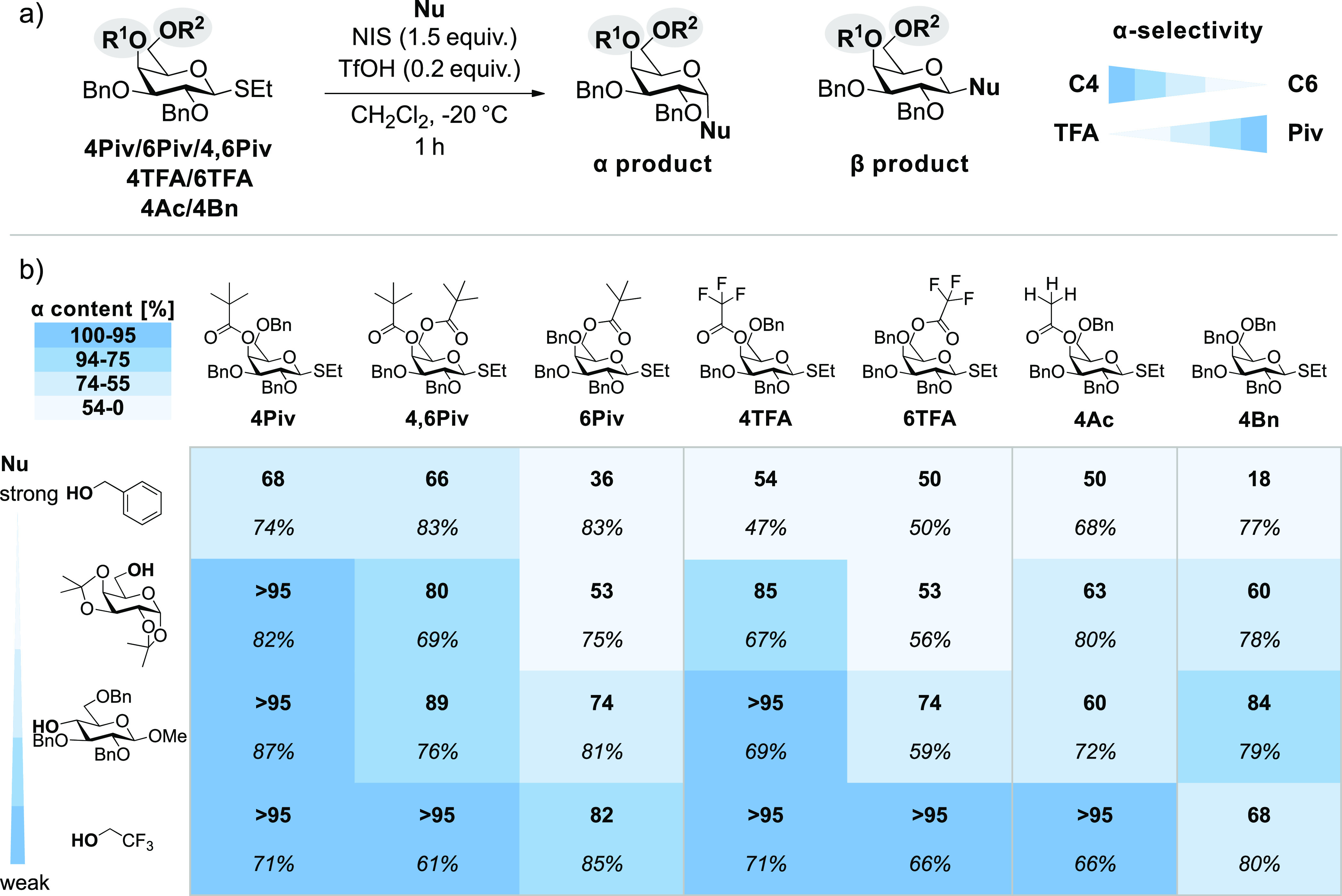
(a) Glycosylation conditions of galactose building blocks (17.5 mM) carrying either Piv, TFA, Ac, or Bn protecting groups at the C4 and C6 positions (**R**^**1/2**^). (b) Stereochemical outcome (**α content** in relation to β-content and *yield*) of the glycosylation reactions of the respective building blocks with different nucleophiles (**Nu**) of decreasing strength. Piv protecting groups at the C4 position lead to increased α-selectivity, while the selectivity is reduced for building blocks carrying the less electron rich TFA, Ac, or Bn protecting groups at that position. Protecting groups at the C6 position do not increase the α-selectivity. Results for **4,6TFA** cannot be shown, as this building block is rapidly decomposing.

Six galactose building blocks carrying pivaloyl or trifluoroacetyl protecting groups at C4, C6, or both positions were synthesized (see SI). Furthermore, two other galactose building blocks, known from previous studies, that are fully benzylated or carry an acetyl group at the C4 position were synthesized. The building blocks were employed in glycosylation reactions with four distinct nucleophiles of different strengths (Figure 1). Generally, weak nucleophiles lead to a higher α-selectivity, which decreases with increasing strength of the nucleophile, in agreement with previous reports.^15^ Glycosyl alcohols are weak nucleophiles,^53^ and hence the observed trend is desirable for the synthesis of α-glycosidic bonds. Furthermore, the α-selectivity is higher for building blocks with an acyl protecting group at C4 than for those with the protecting group at C6. Interestingly, for **4,6Piv**, the α-selectivity is lower than for **4Piv**, although an inverse trend has been reported for similar acetyl building blocks.^10^

Our research groups^4,10^ and others^11,15,29^ have found strong evidence suggesting that remote participation of the C4 protecting group is the origin of the increased α-selectivity of C4-acylated galactose building blocks. For C6-acyl groups, such an effect is not observed. Other groups reported strong evidence that the formation of β-triflates contributes to α-selectivity in glycosylations.^22−24^ The central question of this work is how α-selectivity can be modulated by alterations in the electron density of the acyl protecting groups. For building blocks carrying the acyl group at the C4 position, the electron-rich **4Piv** provides high α-selectivity. The electron-withdrawing **4TFA**, on the other hand, results in significantly lower α-selectivity for the strong nucleophile benzyl alcohol and a sugar nucleophile carrying a free OH group at C6. This result implies that an increase in the electron density on the carbonyl oxygen of the acyl group more likely leads to the formation of a covalent bond with the positively charged anomeric carbon and with that a better shielding of the β-side. However, counterintuitively, the α-selectivity for the **4TFA** building block is higher than expected. There are two possible explanations for this unexpected behavior. Either the electron-withdrawing groups do not inhibit remote participation, but rather weaken it (leading to an equilibrium, where both structures with and without remote participation are present), or a second mechanism, based on α-selective β-triflates could play a role here because their formation is favored due to the longer lifetime of the oxocarbenium species without remote participation.

To elucidate which mechanism is more likely, we performed the same set of test reactions on a **4Ac** building block. Evidence for remote participation on this and similar building blocks has previously been reported.^4,10,11,15^ Solely based on the electron density, this building block would exhibit an α-selectivity that is higher than that of **4TFA** but lower than that of **4Piv**. Interestingly, its selectivity is lower than that of **4TFA**. This finding suggests that in the case of **4TFA** remote participation does not play a role, but rather the formation of β-triflates. This finding is corroborated by a previous study on glucosyl donors, where it was found that dioxolenium ions are the intermediate of donors carrying electron-rich protecting groups, while triflates are the major intermediates when electron-withdrawing groups are used.^54^

The decreased selectivity for **4,6Piv** compared to **4Piv** and **4TFA** can likely be attributed to the steric effects because of the bulky Piv group. Remote participation of the C4-pivaloyl group is less efficient in this building block, as the C6-pivaloyl is partially blocking its trajectory for an intramolecular attack.

In contrast to the C4-acyl variant, a participating protecting group at C6 seems to have no (**6TFA**) or adverse effects (**6Piv**) on the α-selectivity. Adverse effects of C6-acetyl groups on the α-selectivity have been previously reported.^10,11^ With strong nucleophiles, **6Piv** predominantly forms β-products, whereas **6TFA** is not stereoselective. For weaker nucleophiles, the α-selectivity increases, which might be due to counterions or the formation of β-triflates as previously reported.^18,53,55^

As a reference, glycosylation reactions were performed on a fully benzylated galactose building block (**4Bn**). This building block generally exhibits a decreased α-selectivity compared to its C4-acylated counterparts, indicating the importance of a C4-acyl group in achieving high α-selectivity in galactosylations. Intriguingly, the glycosylation reaction with a sugar nucleophile carrying a free OH group at C4 shows a surprisingly high α-selectivity of 84%. Further, it is important to highlight the high yield of the coupling reactions of **4Piv** with sugars, as this is a crucial requirement for AGA.

### Cryogenic Infrared Spectroscopy and Density Functional Theory Investigations of Glycosyl Cations

**Figure 2:**
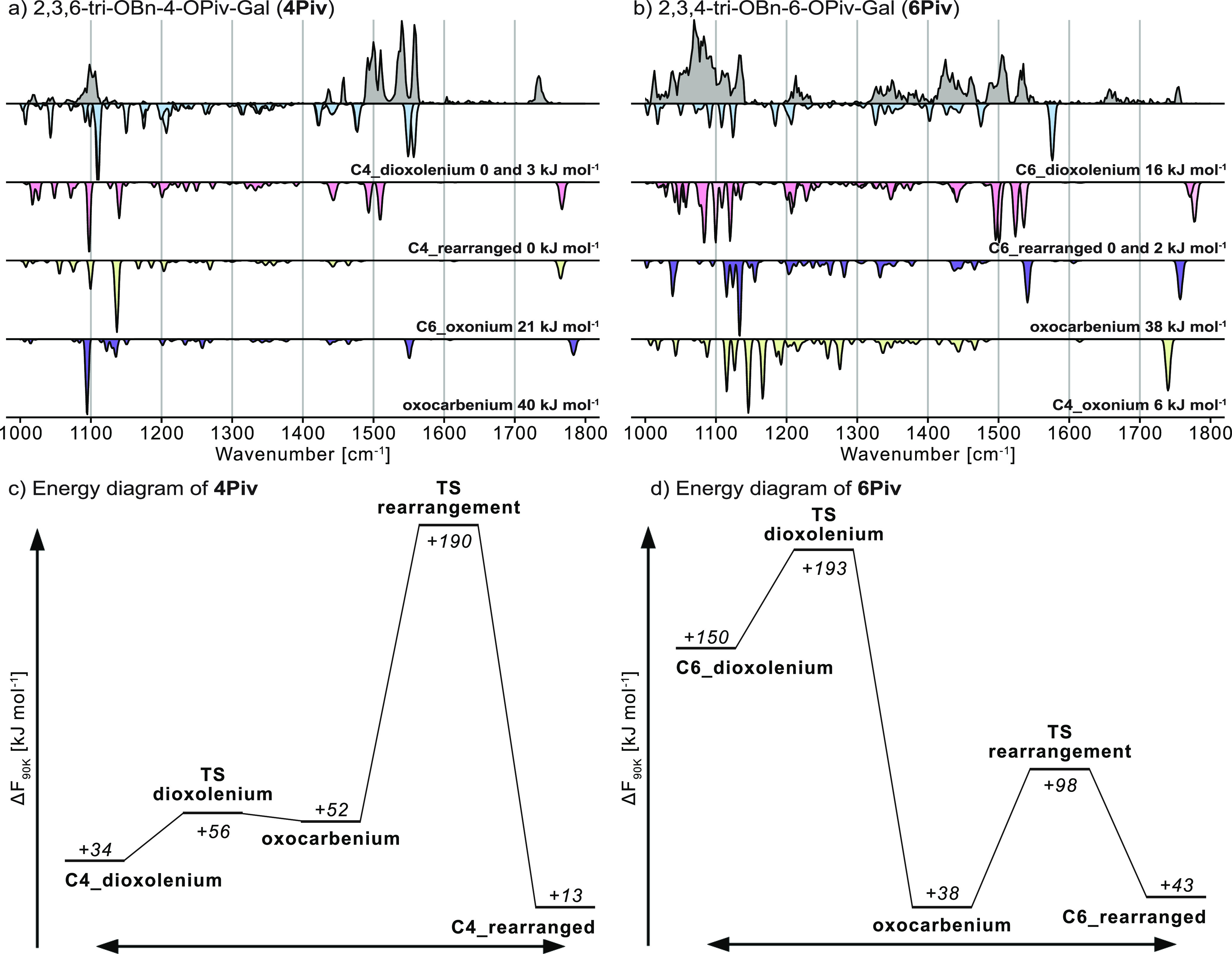
Cryogenic infrared spectra of (a) **4Piv** and (b) **6Piv** galactosyl cations (gray). Computed infrared spectra are shown in the inverted traces for structures showing remote acyl participation (dioxolenium, blue), rearrangement (red), remote benzyl participation (oxonium, yellow), and no participation (oxocarbenium, purple). For **4Piv** the positive charge at the anomeric carbon is mainly stabilized by remote participation of the C4-pivaloyl protecting group. However, further signals can be observed in the experimental spectrum that can be linked to an isoenergetic rearranged structure. The rearranged structure is the dominant motif in the experimental spectrum of the **6Piv** galactosyl cation. Energy diagrams of (c) **4Piv** and (d) **6Piv** show the barriers for remote participation and rearrangement (note that the minimum structures in the diagram do not necessarily correspond to the global minimum). The barrier for remote participation in **4Piv** (C4_dioxolenium) is surprisingly small.

**Figure 3:**
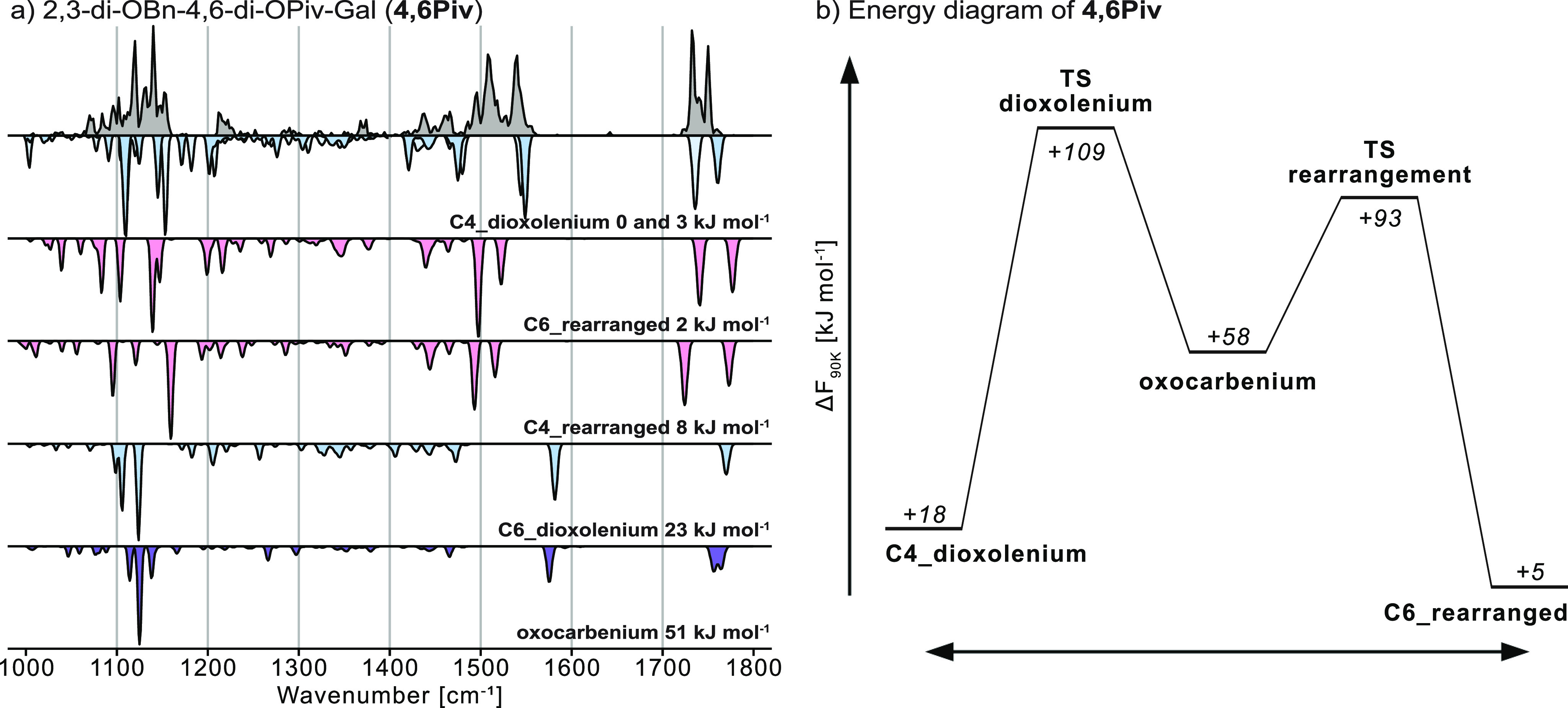
(a) Cryogenic infrared spectrum of the (a) **4,6Piv** galactosyl cation (gray). Computed infrared spectra are shown in the inverted traces for structures showing remote acyl participation (dioxolenium, blue), rearrangement (red), and no participation (oxocarbenium, purple). The positive charge at the anomeric carbon is mainly stabilized by remote participation of the C4-pivaloyl protecting group. However, further signals in the experimental spectrum can be linked to an isoenergetic rearranged structure. (b) Energy diagram of **4,6Piv** showing the barrier for remote participation and rearrangement (note that the minimum structures in the diagram do not necessarily correspond to the global minimum).

**Scheme 1:**
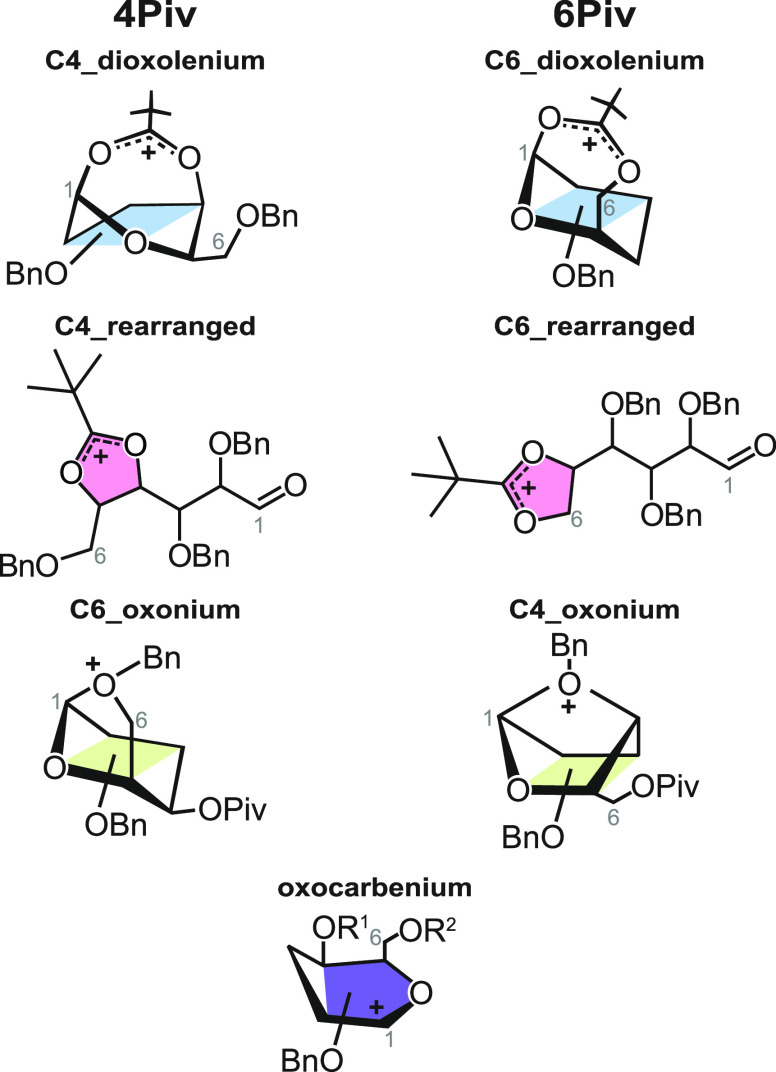
Structures That Can Be Adopted by (Left) **4Piv** and (Right) **6Piv** Galactosyl Cations Oxocarbenium structures can be adopted by both cations. Except for oxonium structures, all five structures can be adopted by **4,6Piv**.

In parallel to the test reactions, the intermediates of the glycosylations—the glycosyl cations—were structurally characterized by cryogenic IR spectroscopy and DFT calculations. Thioglycoside precursors were subjected to in-source fragmentation after nanoelectrospray ionization. Surprisingly, only in the case of pivaloylated building blocks this approach leads to the desired glycosyl cation intermediates. Trifluoroacetylated molecules on the other hand did not fragment sufficiently or decomposed by losing TFA (Figures S2 and S3). Therefore, only galactosyl cations of **4Piv**, **6Piv**, and **4,6Piv** were subjected to cryogenic IR spectroscopy (Figures 2a,b and 3a). The glycosyl cations of **4Ac** and **4Bn** were already probed with the same method in a previous publication.^10^ The experimental spectra can be divided into two main regions: (1) the fingerprint region (1000–1400 cm^–1^), which is predominantly populated by C–O and C–C stretching modes as well as C–H bending vibrations. Due to the complex nature of carbohydrates, this region is usually very challenging to model.^56,57^ (2) The functional group region (1400–1800 cm^–1^) contains most of the diagnostic vibrations of the investigated ions, such as symmetric and antisymmetric dioxolenium ν(O–C–O^+^) and carbonyl stretches ν(C=O). To determine the structure of the probed glycosyl cations, the IR spectra are compared to harmonic frequencies of sampled structures. The sampling mainly yielded dioxolenium structures, which exhibit remote participation of the C4- or the C6-acyl protecting group and oxocarbenium structures (Scheme 1), where no participation occurs at the anomeric carbon (C1). Furthermore, oxonium structures that feature participation of the C4- or C6-benzyl protecting groups at C1 were generated.

For **4Piv**, C4-dioxolenium structures are the lowest in energy and match the experimentally resolved signals at 1090–1110, 1540, and 1558 cm^–1^ well (Figure 2a). The presence of two absorption bands diagnostic for antisymmetric dioxolenium stretches is likely due to the presence of two conformers carrying this structural motif. However, the signals at 1492–1510 cm^–1^ cannot be explained with the sampled structures, and also the carbonyl stretch at 1734 cm^–1^ shows that another type of structures must be present. In a previous study,^15^ it was suggested that acyl groups may attack the C5 atom in glycosyl cations, leading to ring opening and an aldehyde as a product. Therefore, these rearranged ions have been added to the list of structural motifs and were sampled as well (Scheme 1). As the rearranged ions feature a five-membered dioxolenium moiety (compared to the seven-membered dioxolenium moiety observed for remote participation), they are expected to show diagnostic absorption bands in the functional group region.^58^ Indeed, the C4-rearranged structure is isoenergetic to the lowest-energy C4-dioxolenium structure, and its dioxolenium and carbonyl stretches match the remaining experimentally resolved absorption bands. The observations indicate that the spectra observed for **4Piv** are resulting from a mixture of C4-dioxolenium ions and rearrangement products present in the hexapole ion trap after ionization.

The C4-dioxolenium structure is in line with the α-selectivity observed in the glycosylation reactions. In contrast, our results indicate that the C4-rearrangement product is unique to the gas-phase conditions, as none of the expected side products is observed in the test reactions. The literature on the presence of rearranged structures in the condensed phase is generally scarce. Ring opening occurring to a minor degree after the glycosylation reaction of a glucosyl donor carrying three trichloroacetimidate groups has been reported.^59^ Based on our results the rearranged structure does not seem to play a dominant role in the here reported glycosylation reactions. Other structural motifs including C6-oxonium and oxocarbenium ions were sampled, and their harmonic frequencies are compared to the experimental infrared spectrum. Contrary to the dioxolenium and rearranged structures, their computed spectra do not match with the experiment. Based on this result and their higher relative free energy of 21 and 40 kJ mol^–1^, respectively, their presence in the ion trap can be ruled out.

For **6Piv**, the computed harmonic frequencies of the sampled C6-dioxolenium structure do not match the experimental spectrum (Figure 2b). Furthermore, the corresponding C6-rearranged structure is stabilized by −16 kJ mol^–1^, and its frequencies match the experimentally resolved absorption bands at 1421–1461, 1506, and 1533 cm^–1^ well. The oxonium structure is surprisingly low in energy (+6 kJ mol^–1^), but can, like the oxocarbenium structure (+38 kJ mol^–1^), be ruled out due to its poor spectral match. Hence, C6-acyl participation is unlikely to exist for Piv groups, in line with the poor α-selectivity of these building blocks.

These finding are corroborated by computed transition states that are connecting dioxolenium, oxocarbenium, and rearranged structures for **4Piv** and **6Piv** glycosyl cations displayed in the energy diagrams in Figure 2c,d. The geometries that are connected by the transition states do not necessarily correspond to the global minima that we previously sampled. For **4Piv**, the diagram shows that the surface is shallow except for the transition state leading from the oxocarbenium to the rearranged structure. The barrier for remote participation is surprisingly small (+4 kJ mol^–1^), and therefore remote participation is very likely occurring for this species. Hence, the high kinetic barrier that was postulated^60^ for this type of interaction does at least for the gas phase not exist. The relative barrier of +138 kJ mol^–1^ for rearrangement can according to previous studies^61,62^ be overcome using in-source fragmentation, leading to the thermodynamically stable rearranged ion. Once the energy in the ion source is high enough to overcome the transition state, thermodynamically stable species can coexist in the ion trap.

For **6Piv**, the formation of the rearranged product is favored both kinetically and thermodynamically. Furthermore, in previous studies on similar acetylated building blocks, the rearrangement was only observed for C6-acetylated galactosyl cations, whereas it was not reported for its C4-acetylated counterparts.^10,15^ Hence, the results suggest that increasing the electron density within the acyl protecting group enhances remote participation (in both the gas and the condensed phase), but also facilitates a gas-phase rearrangement of the ions. However, the latter does not have an implication on condensed-phase reactivity of the precursors.

For **4,6Piv**, the experimental IR signature (Figure 3a) is similar to that of **4Piv**. The absorption band at 1540 cm^–1^ is diagnostic for C4-dioxolenium structures, whereas the absorption bands at 1495 and 1508 cm^–1^ are diagnostic for the five-membered dioxolenium motif in rearranged structures. Although the predicted frequencies for C6- and C4-rearranged **4,6Piv** are similar, the C6-rearranged structure matches slightly better, especially in the carbonyl stretch region, and is also lower in energy than the C4-rearranged analog (+2 vs +8 kJ mol^–1^). The harmonic frequencies of computed low-energy C6-dioxolenium and oxocarbenium ions do not match the experimental data, and their relative free energies are significantly higher than those of the C4-dioxolenium and rearranged structures. Hence, similarly to **4Piv**, this result suggests that the formation of C4-dioxolenium intermediates with remote participation of the C4-pivaloyl group contributes to the α-selectivity of **4,6Piv** that can be observed in condensed-phase glycosylation reactions. Transition states connecting dioxolenium, oxocarbenium, and rearranged structures and subsequent energy diagrams were also computed for **4,6Piv** (Figure 3b). Here, both the transition states and the products are similar in energy, explaining their coexistence in the experiment. Furthermore, the barrier of C4-dioxolenium ion formation (remote participation) from oxocarbenium ions is significantly higher for **4,6Piv** than for **4Piv** (difference of +47 kJ mol^–1^). This finding highlights that the steric demand of two pivaloyl groups on one glycosyl cation is decreasing the efficiency of remote participation, likely being the cause for the decreased α-selectivity of **4,6Piv** compared to **4Piv** in glycosylation reactions.

Although it was not possible to generate glycosyl cations out of the TFA protected building blocks for cryogenic IR spectroscopy, it is still possible to compute their structures and energetics to rationalize the observed reactivity in glycosylation reactions. The energetics shown in Tables S3–S5 (**4/6/4,6Piv**) and Tables S6–S8 (**4/6/4,6TFA**) show that remote participation of the C4-pivaloyl leading to dioxolenium structures is favored by 40–51 kJ mol^–1^ over oxocarbenium structures in which no participation takes place. Structures with remote participation of C4-TFA can be generated, but their relative energetics are similar (2–4 kJ mol^–1^) to oxocarbenium structures. Interestingly, for **4TFA** C6-oxonium structures are stabilized by −24 kJ mol^–1^ compared to low-energy C4-dioxolenium structures. Such a structure was previously reported for a fully benzylated galactosyl cation, without a clear implication on the condensed phase reactivity.^10^ Furthermore, the calculations show that the C–O bond between the acyl protecting group and the anomeric carbon is, in comparison to Piv, significantly weakened when remote participation of TFA occurs (1.61 vs 1.52 Å). These results, as well as the energy diagram shown in Figure S7a, indicate that remote participation of the C4-trifluoroacetyl group is thermodynamically unfavored, while the energy of the transition state leading to a C4-dioxolenium structure is not particularly high. Furthermore, if remote participation takes place, the effect is weaker than for the C4-pivaloylated counterparts, indicating that it would be less efficient and therefore lead to a decreased α-selectivity. Yet, even though the α-selectivity of **4TFA** is clearly lower than that of **4Piv**, it is higher than one would expect without remote participation and higher than for **4Ac**, a precursor for which remote participation has previously been reported.^10,15^

The gas-phase conditions under which we study the glycosyl cations are not identical to those in the condensed phase during glycosylation reactions, yet there are clear correlations that are worth pointing out. In this study, it was found that C4-pivaloylated glycosyl cations are stabilized by remote participation in the gas phase. If that intermediate is attacked by a nucleophile, the α-product would preferentially be formed. Based on previous studies, there is a consistent trend in the condensed phase that C4-acylated species are more α-selective than their non- or differently acetylated counterparts.^4,10,11,15^ Furthermore, the bridged dioxolenium intermediate was linked to the α-selectivity observed for these building blocks by condensed phase studies in organic solvents using low-temperature NMR spectroscopy.^19^ Because of those findings, we are convinced that remote participation is at least contributing to the selectivity of C4-acylated building blocks.

The glycosylation reaction and its selectivity are governed by an S_N_1–S_N_2 continuum, and the herein presented selectivities are illustrating this continuum. The selectivity of the S_N_1 side is dominated by the structure of the glycosyl cation, whereas the S_N_2 side is dominated by the structure of the glycosyl triflates.^18,60^ In the condensed phase, the lifetime of the glycosyl cation is very short, leading to the quick formation of a thermodynamically stable intermediate that is potentially stereoselective.^24^ The exact mechanism of the glycosylation reaction is currently unclear. Based on the current knowledge it is likely that there are at least two pathways that are contributing to the selectivity observed in glycosylation reactions, depending on various parameters, such as the donor and acceptor reactivities, temperature, solvent, or promoters.^23,63^

This and other studies showed that remote participation of C6-acyl groups does not occur.^10,11,15^ Except for weak nucleophiles, C6-acylated building blocks are not α-selective. For building blocks carrying a C4-acyl group, it is established that remote participation occurs. The fundamental question of this manuscript is how the electron density in acyl protecting groups influences the stereochemical outcome of a glycosylation reaction. Here, the electron density increases as **4TFA** < **4Ac** < **4Piv**, while the α-selectivity increases as **4Ac** < **4TFA** < **4Piv**. As a consequence, it is not the α-selectivity that increases with increasing electron density on the C4-acyl protecting group, but rather the strength of remote participation. Also, based on these findings, remote participation alone can explain the high α-selectivity of the electron-rich **4Piv**, but not that of the less selective electron-deficient **4TFA** building block. Here, the longer lifetime of oxocarbenium-type intermediates without remote participation could favor the formation of β-triflates, leading to an increased α-selectivity.^54^ For **4Ac**, on the other hand, remote participation was previously established, but is not as selective as for **4Piv** due to the decreased electron density.

### Automated Glycan Assembly

**Figure 4:**
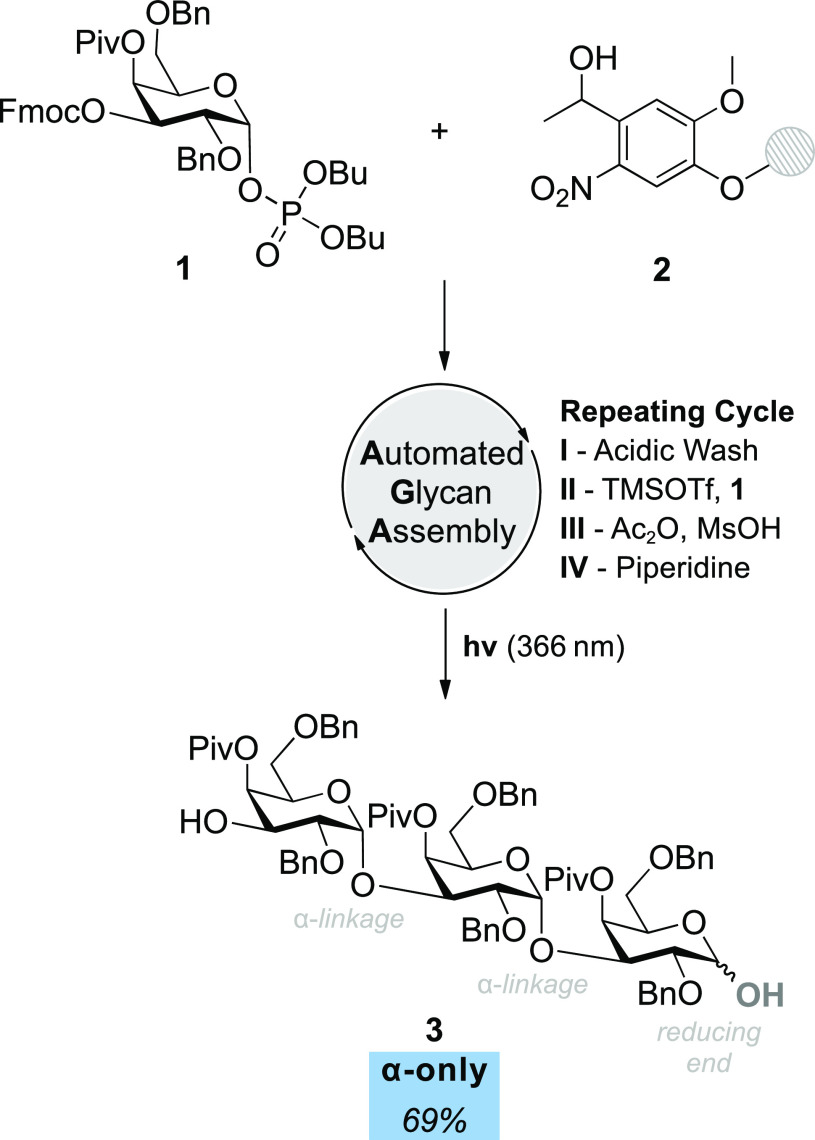
Automated glycan assembly employing the **4Piv** galactosyl building block **1** leads to an α(1,3)-d-trigalactopyranoside **3** with total α-selectivity (determined after the AGA assembly, but before isolation) and a yield of 69% in 6 h using solid support **2** and a coupling cycle consisting of module **I**, acidic wash; **II**, glycosylation; **III**, capping; and **IV**, Fmoc-deprotection, followed by photocleavage (for more detailed conditions see SI).

The combination of the results on the nature and position of the acyl groups on the α-selectivity led to the design of the **4Piv** building block **1** (Figure 4), which can be readily implemented in AGA workflows due to differential protecting groups. Temporary fluorenylmethoxycarbonyl (Fmoc) protection at the C3 position ensured regioselective extension, while the more reactive phosphate leaving group at C1 ensured high yields. Employed in AGA (Figure 4 and SI), **1** was used to assemble the α(1,3)-galactose trisaccharide **3** at high yield and with full α-selectivity.

## Conclusions

This study shows that electron-donating substituents on participating acyl protecting groups increase the efficiency of remote participation, leading to a higher α-selectivity in glycosylation reactions, as shown for pivaloyl groups. Computational results suggest that electron-withdrawing substituents, such as trifluoroacetyl groups, on the other hand, deactivate remote participation, possibly leading to a decrease in selectivity of the reaction. However, the **4TFA** building block is more α-selective than expected, which can be attributed to a favored formation of β-triflates. The presented data confirm that the C4 position plays a more important role in inducing selectivity than the C6 position. In the gas phase, remote participation of the C4-pivaloyl group can be observed, suggesting a role of that effect in the high α-selectivity for the **4Piv** building block. Furthermore, the computed barrier for remote participation is very low, and therefore it can be assumed that it is a fast process. The increased electron density in pivaloyl groups also leads to an increased rearrangement of glycosyl cations in the gas phase, for which no influence on the reactivity in solution was observed. The mechanistic insights were used to tailor a **4Piv** building block that was successfully employed in AGA to synthesize an α(1,3)-trigalactopyranoside with total α-selectivity. In summary, our results show how α-selective building blocks can be developed by rational design and thus provide guiding points on how to fine-tune the selectivity and efficiency of glycosylations.

## Supporting Information Available

A detailed description of the experiment, mass spectra, computed energetics, spectra, and structures (PDF)
*xyz*-coordinates (XYZ)

## Supplementary Material

ja2c05859_si_001.pdf

ja2c05859_si_002.xyz
