# Supplementary material for: The Influence of the Electron Density in Acyl Protecting Groups on the Selectivity of Galactose Formation
Source: J Am Chem Soc. 2022 Oct 27;144(44):20258–66. doi: 10.1021/jacs.2c05859 (PMC9650713; doi:10.1021/jacs.2c05859)
Supplement: Supplementary file 1 — ja2c05859_si_001.pdf [file ja2c05859_si_001.pdf]

Supplementary Information

The Influence of the Electron Density in Acyl Protecting Groups on the Selectivity of Galactose Formation

Kim Greis,[a,b†] Sabrina Leichnitz,[a,c†] Carla Kirschbaum,[a,b] Chun-Wei Chang,[a] Mei-Huei Lin,[a] Gerard Meijer,[b] Gert von Helden,[b] Peter H. Seeberger,*[a,c] Kevin Pagel*[a,b]

(a) Institut für Chemie und Biochemie, Freie Universität Berlin, Arnimallee 22, 14195 Berlin, Germany
(b) Fritz-Haber-Institut der Max-Planck-Gesellschaft, Faradayweg 4-6, 14195 Berlin, Germany
(c) Max-Planck-Institut für Kolloid- und Grenzflächenforschung, Am Mühlenberg 1, 14476 Potsdam, Germany

† K.G. and S.L. contributed equally.

Correspondence to: Prof. Dr. Kevin Pagel
kevin.pagel@fu-berlin.de

Prof. Dr. Peter Seeberger
peter.seeberger@mpikg.mpg.de

1 Mass Spectrometry and Infrared Spectroscopy 3
1.1 Experimental Setup 3
1.2 Mass Spectra 5
2 Computational Methods 7
2.1 Method Description 7
2.2 Energetics 9
2.3 Energy Hierarchies 15
2.4 Energy Diagrams 18
2.5 3D Structures 19
2.6 xyz-Coordinates of reoptimized structures 24
3 General Information 25
4 Materials and Conditions for Automated Synthesis 25
4.1 Materials and Measurements 25
4.2 Preparation of Stock Solutions 26
4.3 Modules for Automated Synthesis 26
4.4 Post-automated Synthesis Manipulations, Analysis and Purification 27
4.5 General Procedure for Glycosylations 28
4.6 Determination of Alpha/Beta Ratios and Purification 29
5 Synthesis of building blocks 30
6 Determination of alpha/beta-ratios 68
7 Automated Glycan Assembly of Building Block 1 147
8 Literature 151

1 Mass Spectrometry and Infrared Spectroscopy

1.1 Experimental Setup

The precursors were dissolved in a 9:1 (V:V) mixture of acetonitrile and water to yield 0.1 mM solutions. Pd/Pt coated glass capillaries (Sputter Coater HR 208, *Cressington*) for nano electrospray ionization (nESI) are pulled to a tip with an inner diameter of 1–2 μm using a micropipette puller (Model P-1000, *Sutter Instrument*).

Glycosyl cations were generated and probed using a custom-built helium droplet instrument (Figure S1). Glycosyl cations are formed after nESI (Z-spray) with a voltage of 1 kV to the tip of the capillary of the precursors, followed by in-source fragmentation of the generated ions. Commonly, nESI of the precursor leads to sodiated and protonated ions, however, labile leaving groups, such as SEt, can be cleaved by in-source fragmentation.

After passing through two ring-electrode ion guides, the ions of interest are mass-to-charge selected by a quadrupole mass filter. Then, the ions enter a quadrupole bender. If no voltage is applied, the ions directly pass through the bender to get to a time-of-flight detector to record mass spectra (Figures S2 and S3) and to monitor the ion signal. If a voltage is applied to the quadrupole bender, the ions are bent and enter a hexapole ion trap that is cooled to 90 K by liquid nitrogen in this experiment. The ions of interest are subsequently accumulated in the ion trap and thermalized by collisions with helium buffer gas.

Expansion of pressurized helium into the vacuum by a pulsed Even-Lavie valve leads to the formation of a beam of superfluid helium nanodroplets (0.4 K) that is traversing the ion trap, picking up ions, rapidly cool them, and guiding them to the detection region. Here, an infrared (IR) beam generated by the Fritz Haber Institute free-electron laser (FHI FEL[1]) overlaps with the ion beam. Upon the absorption of resonant photons, vibrational modes of the molecular ions are excited. The ions dissipate the energy to the helium matrix to get back to their ground state. After the absorption of multiple photons, the probed ions are release from the helium nanodroplets and detected by a time-of-flight detector. The ion yield can be plotted as a function of the IR wavenumber, leading to an IR spectrum (Figures 2 and 3). Due to the multiphoton absorption process, the intensities in the obtained IR spectrum do not scale linearly. As a first-order correction, the ion signal is divided by the energy of the IR macropulse.

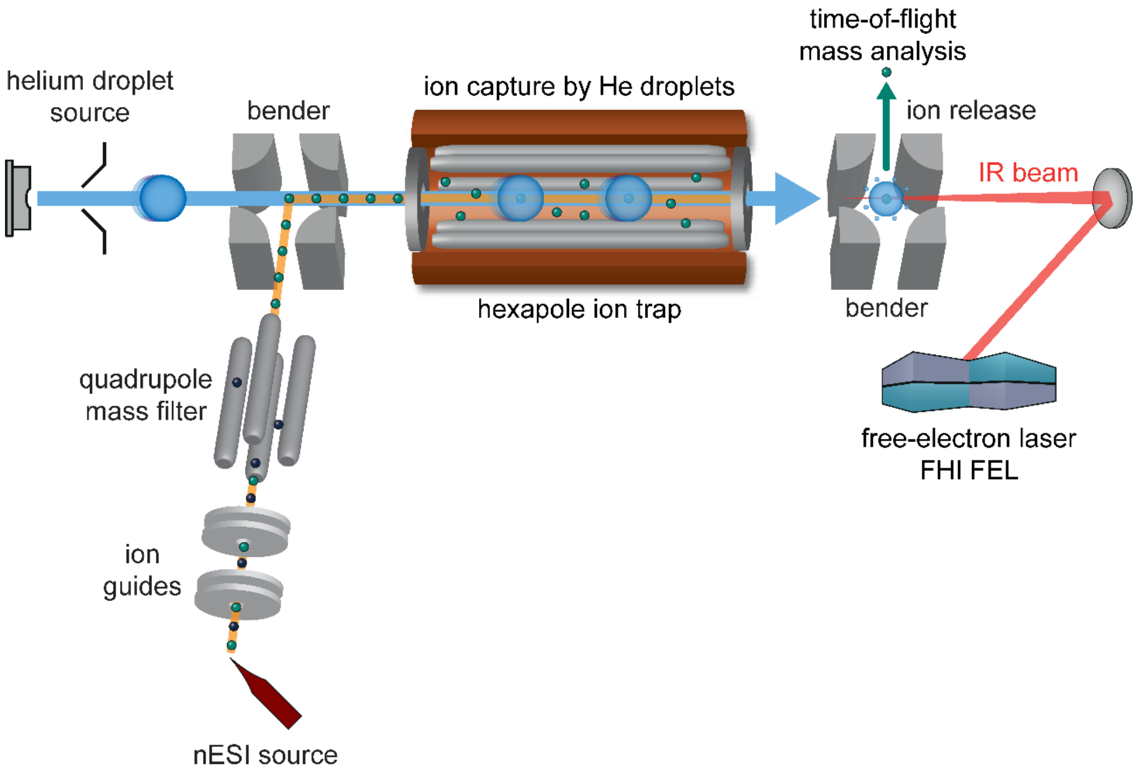

Figure S1. Schematic drawing of the custom-built helium droplet instrument combining mass spectrometry and infrared spectroscopy to probe mass-to-charge selected ions.

1.2 Mass Spectra

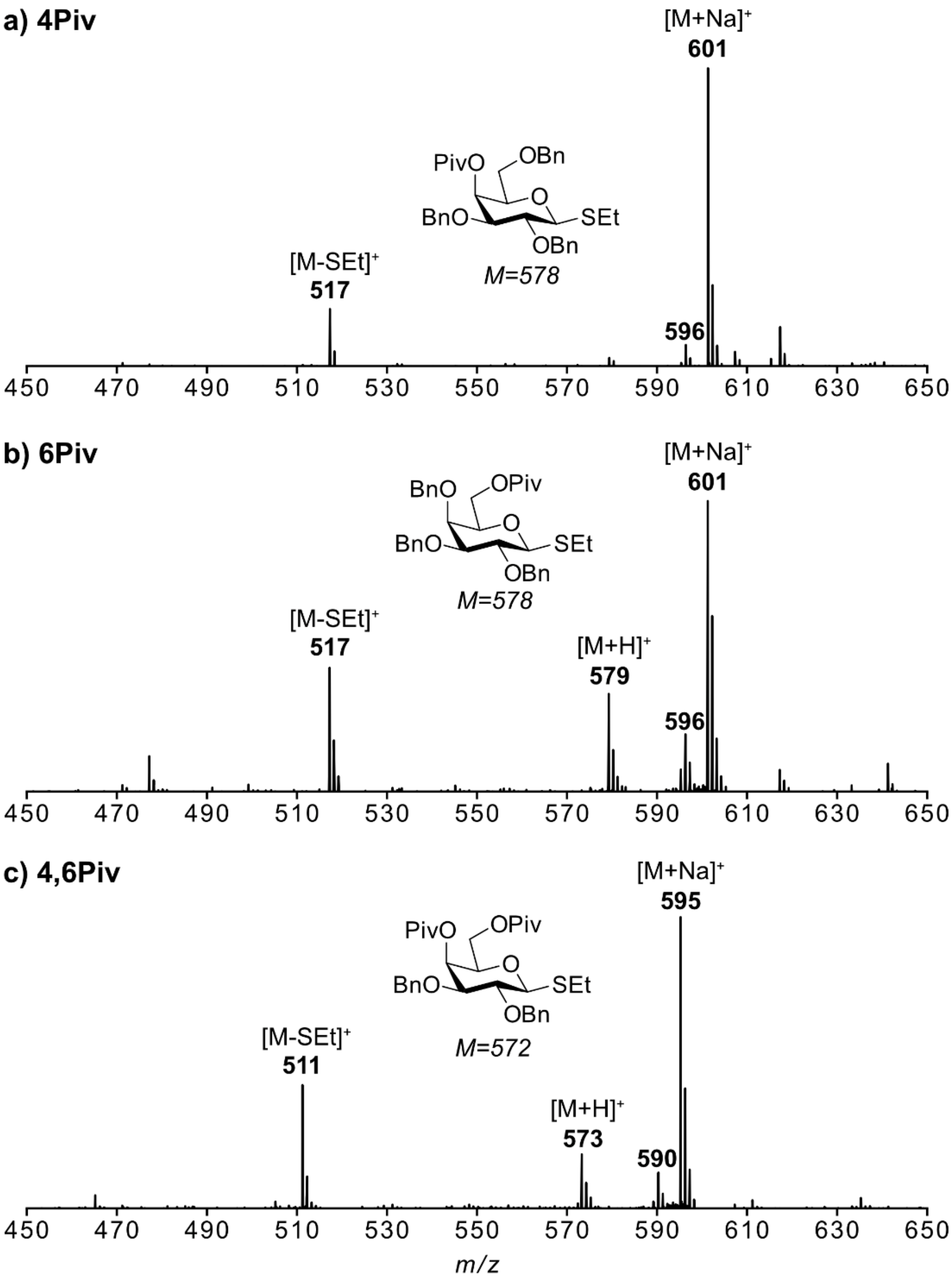

Figure S2. Mass spectra of β-thioethyl (SEt) precursors of (a) 2,3,6-tri-*O*-benzyl-4-*O*-pivaloyl-D-galactopyranosyl (**4Piv**), (b) 2,3,4-tri-*O*-benzyl-6-*O*-pivaloyl-D-galactopyranosyl (**6Piv**), and (c) 2,3-di-*O*-benzyl-4,6-di-*O*-pivaloyl-D-galactopyranosyl cations (**4,6Piv**) recorded on the helium droplet instrument. In-source fragmentation of precursor ions $[M+H]^+$ (*m/z* = 579 and 573), $[M+NH_4]^+$ (*m/z* = 596 and 590), and $[M+Na]^+$ (*m/z* = 601 and 595) leads to galactosyl cations (*m/z* = 517 and 511).

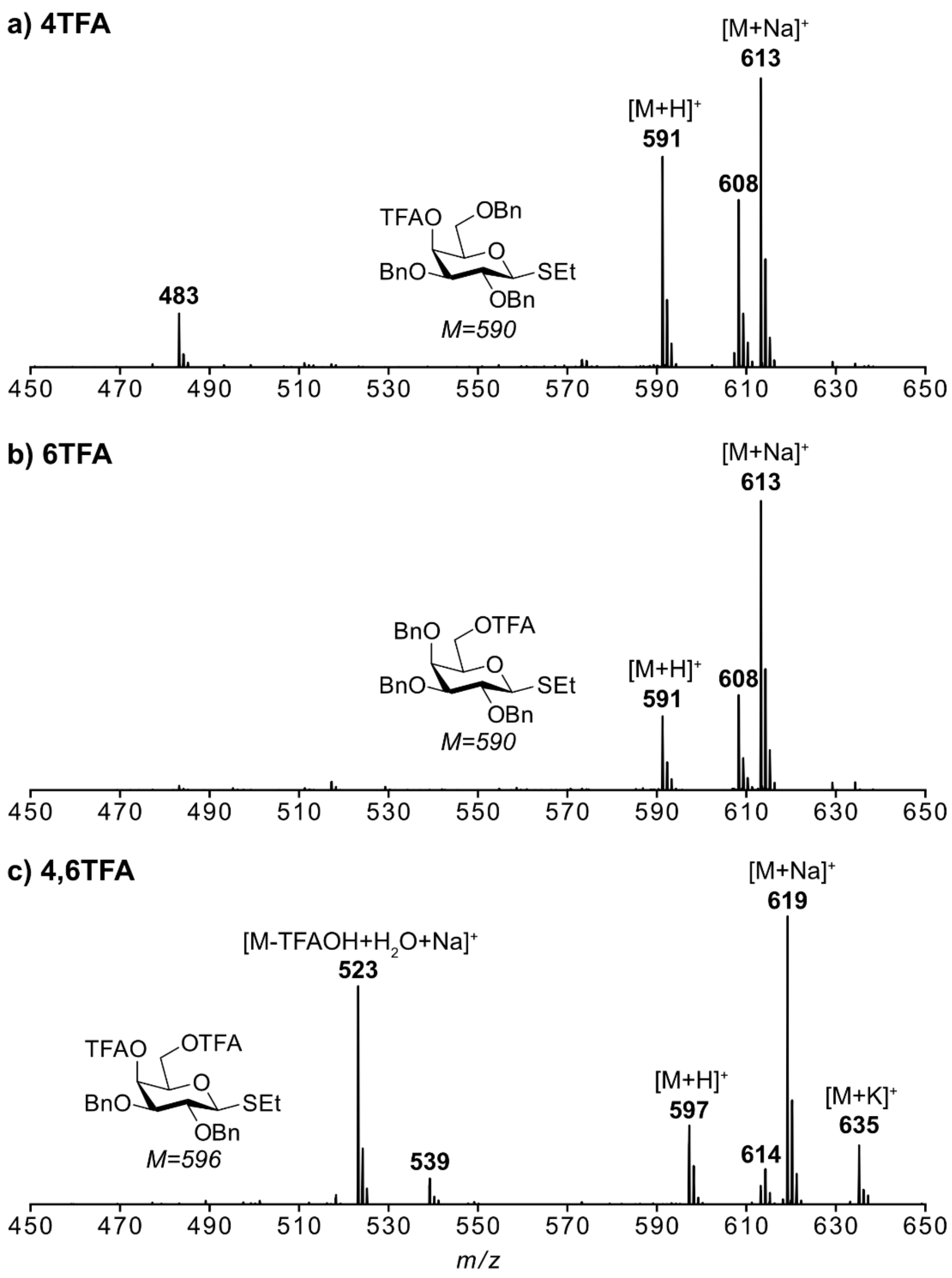

Figure S3. Mass spectra of (a) 2,3,6-tri-*O*-benzyl-4-*O*-trifluoroacetyl-D-galactopyranoside (**4TFA**), (b) 2,3,4-tri-*O*-benzyl-6-*O*-trifluoroacetyl-D-galactopyranoside (**6TFA**), and (c) 2,3-di-*O*-benzyl-4,6-di-*O*-trifluoroacetyl-D-galactopyranoside (**4,6TFA**) carrying a β-thioethyl (SEt) leaving group, recorded on the helium droplet instrument. In-source fragmentation conditions do not lead to fragmentation of the precursor ions $[M+H]^+$ (*m/z* = 591 and 597), $[M+NH_4]^+$ (*m/z* = 608 and 614), and $[M+Na]^+$ *(m/z* = 613 and 619). The **4,6TFA** building block partially hydrolyses, leading to the sodiated and potassiated molecular ions at *m/z* = 523 and 539.

2 Computational Methods

2.1 Method Description

The genetic algorithm (GA) FAFOOM[2] was used to sample the conformational space of intact **4Piv**, **6Piv**, **4,6Piv**, **4TFA**, **6TFA**, **4,6TFA** galactosyl cations. Furthermore, for **4Piv**, **6Piv**, and **4,6Piv** also the conformational space of rearranged galactosyl cations was sampled. With the GA all rotatable bonds and pyranose puckers can be sampled. After its generation, each structure is sent to the external software FHI-aims[3] (version 171221) for local DFT geometry optimization at the dispersion corrected PBE+vdW^{TS}[4-5] level of theory using *light* basis set settings for all atoms. For each galactosyl cation and their rearranged counterparts multiple separate GA runs were carried using the settings in Table S1. The number of generated structures is shown in Table S2 and the energy hierarchies are represented in Figures S4-S6. The GA sampling of the intact galactosyl cations yielded structures with five different modes of participation: dioxolenium-type structures exhibiting remote participation either from the C4- or the C6-acyl protecting group, oxonium-type structures exhibiting *non-classical* remote participation either from the C4- or the C6-benzyl protecting group, and oxocarbenium-type structures in which no participation occurs (Figures S8-S13).

Table S1. GA parameters used in initial search of intact galactosyl cations. For rearranged galactosyl cations the function to sample the pyranose pucker was switched off.

	Parameter	Value
Molecule	Distance_cutoff_1	1.2
	Distance_cutoff_2	2.15
	Rmsd_cutoff_uniq	0.25
GA settings	Popsize	10
	Prob_for_crossing	0.95
	Prob_for_mut_pyranosering	0.6
	Prob_for_mut_torsion	0.8
	Fitness_sum_limit	1.2
	Selection	Roulette wheel
	Max_mutations_torsion	3
	Max_mutations_pyranosering	1

From all the structures generated by the GA, several distinct low-energy structures for each mode of participation, specified in Table S2, were selected for reoptimization and harmonic frequency calculation at the PBE0+D3/6-311+G(d,p)[6-8] level of theory in Gaussian 16, Revision A.03[9] using default settings. The energies including the zero-point vibrational energy (ZPVE) and free energies at 90 K (the temperature in the hexapole ion trap) of the reoptimized structures are shown in Tables S3-S8 and free energy hierarchies in

Figures S4-S6. The computed IR spectra, generated from harmonic frequency calculations, were normalized and scaled by an empirical factor of 0.965.

In a last step, the structures reoptimized at the PBE0+D3/6-311+G(d,p) level of theory were used for single-point energy calculations at the DLPNO-CCSD(T)/Def2-TZVPP[10-11] level of theory as implemented in ORCA 5.0.3.[12] The relative single-point energies are reported in Tables S3-S8 as such and with the ZPVE plus free energy correction at 90 K derived from previous calculations at the PBE0 level of theory.

Table S2. Number of generated structures during GA sampling and reoptimized structures.

Galactosyl Cations	#(GA Structures)	#(Reoptimized Structures) **dioxolenium**		**rearranged**		**oxonium**		**oxocarbenium**
		**C4**	**C6**	**C4**	**C6**	**C4**	**C6**	
**4Piv** intact	599	10	–	–	–	–	5	5
**4Piv** rearranged	188	–	–	5	–	–	–	–
**6Piv** intact	548	–	10	–	–	5	–	5
**6Piv** rearranged	205	–	–	–	5	–	–	–
**4,6Piv** intact	673	10	5	–	–	–	–	5
**4,6Piv** rearranged	176	–	–	5	–	–	–	–
**4,6Piv** rearranged	162	–	–	–	5	–	–	–
**4TFA** intact	561	11	–	–	–	–	5	5
**6TFA** intact	533	–	3	–	–	5	–	5
**4,6TFA** intact	493	10	3	–	–	–	–	6

2.2 Energetics

Table S3. List of structures of the **4Piv** glycosyl cation reoptimized at the PBE0+D3/6-311+G(d,p) level of theory. Ring puckers, bond distances between the carbonyl oxygen of the C4-pivaloyl group and the anomeric carbon (C1), energies (ΔE, including zero-point-vibrational energy) and free energies (ΔF) at 90 K are assigned to each structure. Furthermore, single-point energies of these reoptimized structures at the DLPNO-CCSD(T)/Def2-TZVPP level of theory are indicated as such (ΔE, not including ZPVE) and with ZPVE plus free energy correction at 90 K (ΔF) derived from the PBE0 calculation. The infrared spectra of the structures labelled with an asterisk are represented in the manuscript. In the last six rows, the energetics of transition states connected to minima via intrinsic reaction coordinate (IRC) calculations are shown.

ID	Ring Pucker	d(C4=O—C1) [Å]	ΔE(PBE0+D3) [kJ mol^{-1}]	ΔF(PBE0+D3) [kJ mol^{-1}]	ΔE(DLPNO-CCSD(T)) [kJ mol^{-1}]	ΔF(DLPNO-CCSD(T)) [kJ mol^{-1}]
C4_dioxolenium/conf_00 (*)	1S5	1.52	0.00	0.00	0.00	0.05
C4_dioxolenium/conf_01	1S5	1.52	3.28	2.78	3.19	2.10
C4_dioxolenium/conf_02	1S5	1.52	3.07	1.80	4.29	2.25
C4_dioxolenium/conf_03	1S5	1.53	9.09	8.64	8.20	6.97
C4_dioxolenium/conf_04	1S5	1.52	9.36	7.69	9.08	6.87
C4_dioxolenium/conf_05	1S5	1.53	9.67	8.20	9.52	7.15
C4_dioxolenium/conf_06 (*)	1S5	1.51	5.76	4.22	5.64	2.56
C4_dioxolenium/conf_07	1S5	1.52	9.34	7.20	9.13	6.44
C4_dioxolenium/conf_08	1S5	1.54	11.19	8.40	11.93	7.62
C4_dioxolenium/conf_09	1S5	1.51	16.65	12.89	17.12	10.90
C4_rearranged/conf_00 (*)	–	5.93	12.71	8.84	13.44	0.00
C4_rearranged/conf_01	–	4.85	21.67	18.61	16.68	4.55
C4_rearranged/conf_02	–	4.93	27.51	23.14	26.06	12.83
C4_rearranged/conf_03	–	5.20	25.09	21.55	19.44	6.23
C4_rearranged/conf_04	–	4.96	31.85	27.36	27.70	13.52
C6_oxonium/conf_00 (*)	1C4	5.21	29.77	27.66	24.54	20.57
C6_oxonium/conf_01	1C4	5.19	31.26	30.20	25.46	22.45
C6_oxonium/conf_02	1C4	5.20	32.25	31.01	27.04	24.44
C6_oxonium/conf_03	1C4	5.19	31.27	30.22	25.46	22.47
C6_oxonium/conf_04	1C4	5.20	32.25	30.99	27.05	24.43
oxocarbenium/conf_00 (*)	4H3	5.20	40.13	37.80	50.01	40.34
oxocarbenium/conf_01	4H3	5.24	49.24	47.67	59.44	50.64
oxocarbenium/conf_02	E3	5.14	52.17	50.34	61.53	51.00
oxocarbenium/conf_03	4H3	5.16	49.25	44.04	62.48	48.75
oxocarbenium/conf_04	3H4	5.06	54.93	51.64	62.43	51.88
oxocarbenium/conf_IRC1	4H3	2.62	51.98	49.68	60.69	52.21
TS1	4E	2.33	52.65	52.16	62.72	55.80
C4_dioxolenium/conf_IRC1	1,4B	1.55	34.07	33.17	35.67	33.94
oxocarbenium/conf_IRC2	4H3	2.62	51.98	49.68	60.69	52.21
TS2	–	2.91	177.76	176.58	210.86	189.59
C4_rearranged/conf_IRC2	–	3.68	24.54	21.89	23.33	13.33

Table S4. List of structures of the **6Piv** glycosyl cation reoptimized at the PBE0+D3/6-311+G(d,p) level of theory. Ring puckers, bond distances between the carbonyl oxygen of the C6-pivaloyl group and the anomeric carbon (C1), energies (ΔE, including zero-point-vibrational energy) and free energies (ΔF) at 90 K are assigned to each structure. Furthermore, single-point energies of these reoptimized structures at the DLPNO-CCSD(T)/Def2-TZVPP level of theory are indicated as such (ΔE, not including ZPVE) and with ZPVE plus free energy correction at 90 K (ΔF) derived from the PBE0 calculation. The infrared spectra of the structures labelled with an asterisk are represented in the manuscript. In the last six rows, the energetics of transition states connected to minima via intrinsic reaction coordinate (IRC) calculations are shown.

ID	Ring Pucker	d(C4=O—C1) [Å]	ΔE(PBE0+D3) [kJ mol^{-1}]	ΔF(PBE0+D3) [kJ mol^{-1}]	ΔE(DLPNO-CCSD(T)) [kJ mol^{-1}]	ΔF(DLPNO-CCSD(T)) [kJ mol^{-1}]
C6_dioxolenium/conf_00	1C4	1.50	2.79	1.30	6.58	17.30
C6_dioxolenium/conf_01	1C4	1.50	2.87	3.73	4.51	17.70
C6_dioxolenium/conf_02 (*)	1C4	1.50	0.00	0.00	3.41	16.20
C6_dioxolenium/conf_03	1C4	1.49	8.16	8.35	10.56	22.87
C6_dioxolenium/conf_04	1C4	1.49	6.69	5.82	8.96	20.61
C6_dioxolenium/conf_05	1C4	1.51	9.50	8.31	12.13	23.69
C6_dioxolenium/conf_06	1C4	1.49	6.69	5.82	8.96	20.62
C6_dioxolenium/conf_07	1C4	1.49	6.60	5.88	8.75	20.35
C6_dioxolenium/conf_08	1C4	1.50	11.02	9.60	12.81	22.99
C6_dioxolenium/conf_09	1C4	1.50	3.44	3.39	6.19	19.92
C6_rearranged/conf_00	–	4.96	6.49	2.74	5.78	6.46
C6_rearranged/conf_01 (*)	–	4.68	6.56	2.11	2.89	2.05
C6_rearranged/conf_02 (*)	–	4.81	5.81	2.19	0.00	0.00
C6_rearranged/conf_03	–	5.30	10.80	5.66	8.47	5.85
C6_rearranged/conf_04	–	3.94	11.28	8.28	6.47	8.27
C4_oxonium/conf_00 (*)	1,4B	4.20	29.72	28.74	5.78	6.46
C4_oxonium/conf_01	1,4B	3.87	30.26	28.84	27.38	35.35
C4_oxonium/conf_02	1,4B	4.91	48.12	46.29	47.67	53.50
C4_oxonium/conf_03	1,4B	4.76	51.54	49.57	50.96	56.74
C4_oxonium/conf_04	1,4B	3.84	50.14	46.17	47.37	50.62
oxocarbenium/conf_00 (*)	4E	5.04	23.40	21.99	32.72	38.14
oxocarbenium/conf_01	4H3	4.07	33.59	28.72	45.83	45.63
oxocarbenium/conf_02	4H3	3.96	36.33	33.29	46.21	48.24
oxocarbenium/conf_03	3E	3.56	37.68	34.01	46.97	48.65
oxocarbenium/conf_04	3E	3.56	37.68	34.02	46.97	48.66
oxocarbenium/conf_IRC1	4E	4.97	23.52	22.08	32.72	38.01
TS1	E5	1.96	171.70	172.64	185.11	192.54
C6_dioxolenium/conf_IRC1	OS2	1.51	137.11	136.18	138.16	149.67
oxocarbenium/conf_IRC2	4E	5.04	23.29	22.00	33.41	38.83
TS2	–	4.81	81.38	80.53	98.36	98.23
C6_rearranged/conf_IRC2	–	4.82	41.13	38.54	40.86	42.98

Table S5. List of structures of the **4,6Piv** glycosyl cation reoptimized at the PBE0+D3/6-311+G(d,p) level of theory. Ring puckers, bond distances between the carbonyl oxygen of the C6-pivaloyl group and the anomeric carbon (C1), energies (ΔE, including zero-point-vibrational energy) and free energies (ΔF) at 90 K are assigned to each structure. Furthermore, single-point energies of these reoptimized structures at the DLPNO-CCSD(T)/Def2-TZVPP level of theory are indicated as such (ΔE, not including ZPVE) and with ZPVE plus free energy correction at 90 K (ΔF) derived from the PBE0 calculation. The infrared spectra of the structures labelled with an asterisk are represented in the manuscript. In the last six rows, the energetics of transition states connected to minima via intrinsic reaction coordinate (IRC) calculations are shown.

ID	Ring Pucker	d(C4=O—C1) [Å]	ΔE(PBE0+D3) [kJ mol^{-1}]	ΔF(PBE0+D3) [kJ mol^{-1}]	ΔE(DLPNO-CCSD(T)) [kJ mol^{-1}]	ΔF(DLPNO-CCSD(T)) [kJ mol^{-1}]
C4_dioxolenium/conf_00	1S5	1.52	0.00	0.00	0.00	0.14
C4_dioxolenium/conf_01 (*)	1S5	1.51	0.77	0.08	1.08	0.00
C4_dioxolenium/conf_02	1S5	1.51	4.30	5.62	4.57	6.15
C4_dioxolenium/conf_03	1S5	1.52	6.59	6.16	5.53	4.72
C4_dioxolenium/conf_04	1S5	1.52	5.10	5.95	6.72	7.46
C4_dioxolenium/conf_05 (*)	1S5	1.51	4.08	5.29	0.61	3.49
C4_dioxolenium/conf_06	1S5	1.52	4.78	5.76	2.91	5.17
C4_dioxolenium/conf_07	1S5	1.51	4.07	5.29	0.61	3.49
C4_dioxolenium/conf_08	1S3	1.51	18.71	18.07	21.40	20.51
C4_dioxolenium/conf_09	1S5	1.51	7.72	7.57	7.25	7.11
C6_dioxolenium/conf_00 (*)	1C4	5.21	21.42	21.83	23.13	23.37
C6_dioxolenium/conf_01	1C4	5.07	24.77	24.99	26.72	26.63
C6_dioxolenium/conf_02	1C4	5.22	23.66	23.17	26.08	25.98
C6_dioxolenium/conf_03	1C4	5.10	25.43	25.31	27.40	27.56
C6_dioxolenium/conf_04	BO,3	5.21	39.12	40.85	45.04	46.68
C4_rearranged/conf_00 (*)	–	4.34	18.22	17.29	15.93	8.07
C4_rearranged/conf_01	–	4.88	21.91	18.57	19.68	8.14
C4_rearranged/conf_02	–	4.92	23.34	19.40	22.89	10.45
C4_rearranged/conf_03	–	5.27	22.61	19.77	24.09	11.52
C4_rearranged/conf_04	–	5.28	25.41	22.42	25.10	13.18
C6_rearranged/conf_00 (*)	–	5.94	16.43	14.66	11.52	2.36
C6_rearranged/conf_01	–	4.56	15.64	13.01	13.44	3.03
C6_rearranged/conf_02	–	5.17	18.71	17.09	13.59	5.38
C6_rearranged/conf_03	–	5.34	23.84	21.50	19.48	9.94
C6_rearranged/conf_04	–	4.78	25.03	24.21	23.05	14.77
oxocarbenium/conf_00	4H3	5.21	57.76	55.21	71.09	57.95
oxocarbenium/conf_01	4H3	5.19	62.51	59.42	75.62	61.48
oxocarbenium/conf_02	4E	5.18	54.95	52.26	65.56	54.82
oxocarbenium/conf_03 (*)	3E	5.06	52.39	49.48	60.51	50.59
oxocarbenium/conf_04	4H3	5.17	59.40	56.25	71.51	57.66
oxocarbenium/conf_IRC1	4H3	5.21	57.76	55.21	71.11	57.98
TS1	4E	3.73	107.18	104.65	123.75	109.01
C4_dioxolenium/conf_IRC1	1,4B	1.52	13.92	14.87	17.66	17.94
oxocarbenium/conf_IRC2	4H3	5.16	60.75	57.85	73.74	60.24
TS2	–	5.34	91.32	89.40	109.91	93.14
C6_rearranged/conf_IRC2	–	5.32	18.84	15.51	17.02	5.17

Table S6. List of structures of the **4TFA** glycosyl cation reoptimized at the PBE0+D3/6-311+G(d,p) level of theory. Ring puckers, bond distances between the carbonyl oxygen of the C4-pivaloyl group and the anomeric carbon (C1), energies (ΔE, including zero-point-vibrational energy) and free energies (ΔF) at 90 K are assigned to each structure. Furthermore, single-point energies of these reoptimized structures at the DLPNO-CCSD(T)/Def2-TZVPP level of theory are indicated as such (ΔE, not including ZPVE) and with ZPVE plus free energy correction at 90 K (ΔF) derived from the PBE0 calculation.

ID	Ring Pucker	d(C4=O—C1) [Å]	ΔE(PBE0+D3) [kJ mol^{-1}]	ΔF(PBE0+D3) [kJ mol^{-1}]	ΔE(DLPNO-CCSD(T)) [kJ mol^{-1}]	ΔF(DLPNO-CCSD(T)) [kJ mol^{-1}]
C4_dioxolenium/conf_00	1S5	1.61	18.26	18.17	25.39	24.45
C4_dioxolenium/conf_01	1S5	1.60	26.09	26.40	33.13	32.16
C4_dioxolenium/conf_02	1S5	1.61	26.85	28.97	35.29	35.83
C4_dioxolenium/conf_03	1S5	1.59	23.64	22.54	30.85	28.76
C4_dioxolenium/conf_04	1S5	1.62	28.14	26.56	35.79	32.41
C4_dioxolenium/conf_05	1S5	1.59	31.73	30.83	37.88	35.00
C4_dioxolenium/conf_06	1S5	1.57	37.18	36.04	43.58	40.84
C4_dioxolenium/conf_07	1,4B	1.60	41.85	41.22	50.53	48.38
C4_dioxolenium/conf_08	1S5	1.59	39.80	37.76	47.30	43.28
C4_dioxolenium/conf_09	1S5	1.61	40.70	39.03	49.98	45.51
C4_dioxolenium/conf_10	1S5	1.95	23.48	21.90	36.95	31.40
C6_oxonium/conf_00	1C4	5.26	0.00	0.00	0.00	0.00
C6_oxonium/conf_01	1C4	5.10	7.03	5.60	7.06	5.64
C6_oxonium/conf_02	BO,3	4.99	14.34	12.27	15.12	12.34
C6_oxonium/conf_03	1C4	5.16	9.83	6.80	11.84	6.58
C6_oxonium/conf_04	1C4	5.09	12.66	10.85	12.23	9.48
oxocarbenium/conf_00	4E	5.24	15.85	14.57	33.58	26.58
oxocarbenium/conf_01	E3	5.20	17.94	16.88	37.46	29.12
oxocarbenium/conf_02	3H4	5.15	30.05	27.67	42.83	35.03
oxocarbenium/conf_03	4H3	5.22	24.73	22.55	43.66	35.00
oxocarbenium/conf_04	5H4	5.20	30.12	28.06	42.76	35.39
oxocarbenium/conf_IRC1	4H3	2.75	32.44	31.55	47.53	42.15
TS1	1,4B	1.71	51.89	52.52	63.52	61.01
C4_dioxolenium/conf_IRC1	1,4B	1.73	52.31	50.80	63.37	59.14
oxocarbenium/conf_IRC2	4H3	2.75	32.44	31.56	47.54	42.17
TS2	–	2.99	175.75	174.83	214.89	193.42
C4_rearranged/conf_IRC2	–	3.62	60.57	58.60	66.06	56.89

Table S7. List of structures of the **6TFA** glycosyl cation reoptimized at the PBE0+D3/6-311+G(d,p) level of theory. Ring puckers, bond distances between the carbonyl oxygen of the C4-pivaloyl group and the anomeric carbon (C1), energies (ΔE, including zero-point-vibrational energy) and free energies (ΔF) at 90 K are assigned to each structure. Furthermore, single-point energies of these reoptimized structures at the DLPNO-CCSD(T)/Def2-TZVPP level of theory are indicated as such (ΔE, not including ZPVE) and with ZPVE plus free energy correction at 90 K (ΔF) derived from the PBE0 calculation.

ID	Ring Pucker	d(C4=O—C1) [Å]	ΔE(PBE0+D3) [kJ mol^{-1}]	ΔF(PBE0+D3) [kJ mol^{-1}]	ΔE(DLPNO-CCSD(T)) [kJ mol^{-1}]	ΔF(DLPNO-CCSD(T)) [kJ mol^{-1}]
C6_dioxolenium/conf_00	1C4	1.53	10.22	12.21	5.12	11.77
C6_dioxolenium/conf_01	1C4	1.53	17.19	18.73	11.59	16.37
C6_dioxolenium/conf_02	1C4	1.53	35.29	33.10	29.99	29.96
C4_oxonium/conf_00	1,4B	5.35	42.08	36.48	34.48	25.62
C4_oxonium/conf_01	1,4B	5.23	50.67	49.18	41.62	38.59
C4_oxonium/conf_02	1,4B	3.91	48.98	44.00	37.28	31.12
C4_oxonium/conf_03	1,4B	5.15	47.40	43.90	35.53	31.27
C4_oxonium/conf_04	1,4B	5.99	70.53	67.87	60.97	57.00
oxocarbenium/conf_00	4E	5.11	0.00	0.00	0.00	0.00
oxocarbenium/conf_01	5H4	4.80	13.64	12.50	13.14	10.93
oxocarbenium/conf_02	3E	3.62	17.18	14.95	21.17	16.99
oxocarbenium/conf_03	3E	3.65	10.47	8.41	10.62	6.93
oxocarbenium/conf_04	3E	3.77	12.32	11.20	15.80	13.25

Table S8. List of structures of the **4,6TFA** glycosyl cation reoptimized at the PBE0+D3/6-311+G(d,p) level of theory. Ring puckers, bond distances between the carbonyl oxygen of the C4-pivaloyl group and the anomeric carbon (C1), energies (ΔE, including zero-point-vibrational energy) and free energies (ΔF) at 90 K are assigned to each structure. Furthermore, single-point energies of these reoptimized structures at the DLPNO-CCSD(T)/Def2-TZVPP level of theory are indicated as such (ΔE, not including ZPVE) and with ZPVE plus free energy correction at 90 K (ΔF) derived from the PBE0 calculation.

ID	Ring Pucker	d(C4=O—C1) [Å]	ΔE(PBE0+D3) [kJ mol^{-1}]	ΔF(PBE0+D3) [kJ mol^{-1}]	ΔE(DLPNO-CCSD(T)) [kJ mol^{-1}]	ΔF(DLPNO-CCSD(T)) [kJ mol^{-1}]
C4_dioxolenium/conf_00	1S5	1.59	1.82	1.00	0.01	0.01
C4_dioxolenium/conf_01	1S5	1.60	1.07	0.87	1.54	2.09
C4_dioxolenium/conf_02	1S5	1.59	0.00	0.00	1.06	1.70
C4_dioxolenium/conf_03	1S5	2.04	5.15	3.49	12.90	8.92
C4_dioxolenium/conf_04	1S5	2.09	5.34	3.16	12.61	8.07
C4_dioxolenium/conf_05	1S5	2.01	6.17	4.19	12.87	8.54
C4_dioxolenium/conf_06	1S5	1.61	6.00	3.98	4.30	2.71
C4_dioxolenium/conf_07	1S5	1.58	5.04	4.62	3.51	4.05
C4_dioxolenium/conf_08	1S5	2.01	6.17	4.20	12.87	8.54
C4_dioxolenium/conf_09	1S5	1.61	8.66	8.58	6.59	7.23
C6_dioxolenium/conf_00	1C4	5.25	8.25	8.61	12.19	14.45
C6_dioxolenium/conf_01	1C4	5.32	13.84	13.34	14.57	15.55
C6_dioxolenium/conf_02	1C4	5.29	22.38	20.84	24.71	24.03
oxocarbenium/conf_00	OS2	5.09	4.07	1.61	8.91	3.60
oxocarbenium/conf_01	OS2	5.09	4.07	1.61	8.90	3.60
oxocarbenium/conf_02	OS2	5.08	5.09	1.67	9.16	1.98
oxocarbenium/conf_03	OS2	5.09	8.04	5.25	14.76	8.62
oxocarbenium/conf_04	OS2	5.32	12.20	11.33	15.80	11.37
oxocarbenium/conf_05	3E	5.17	8.74	5.40	15.66	8.68
oxocarbenium/conf_IRC1	4H3	5.25	20.48	16.98	35.74	23.96
TS1	4H3	3.88	62.79	59.98	79.34	67.14
C4_dioxolenium/conf_IRC1	1S5	1.59	1.82	1.00	0.00	0.00
oxocarbenium/conf_IRC2	4H3	5.22	18.56	14.87	31.03	19.56
TS2	–	5.38	84.39	81.13	101.26	85.30
C6_rearranged/conf_IRC2	–	5.29	53.83	49.35	48.60	37.34

2.3 Energy Hierarchies

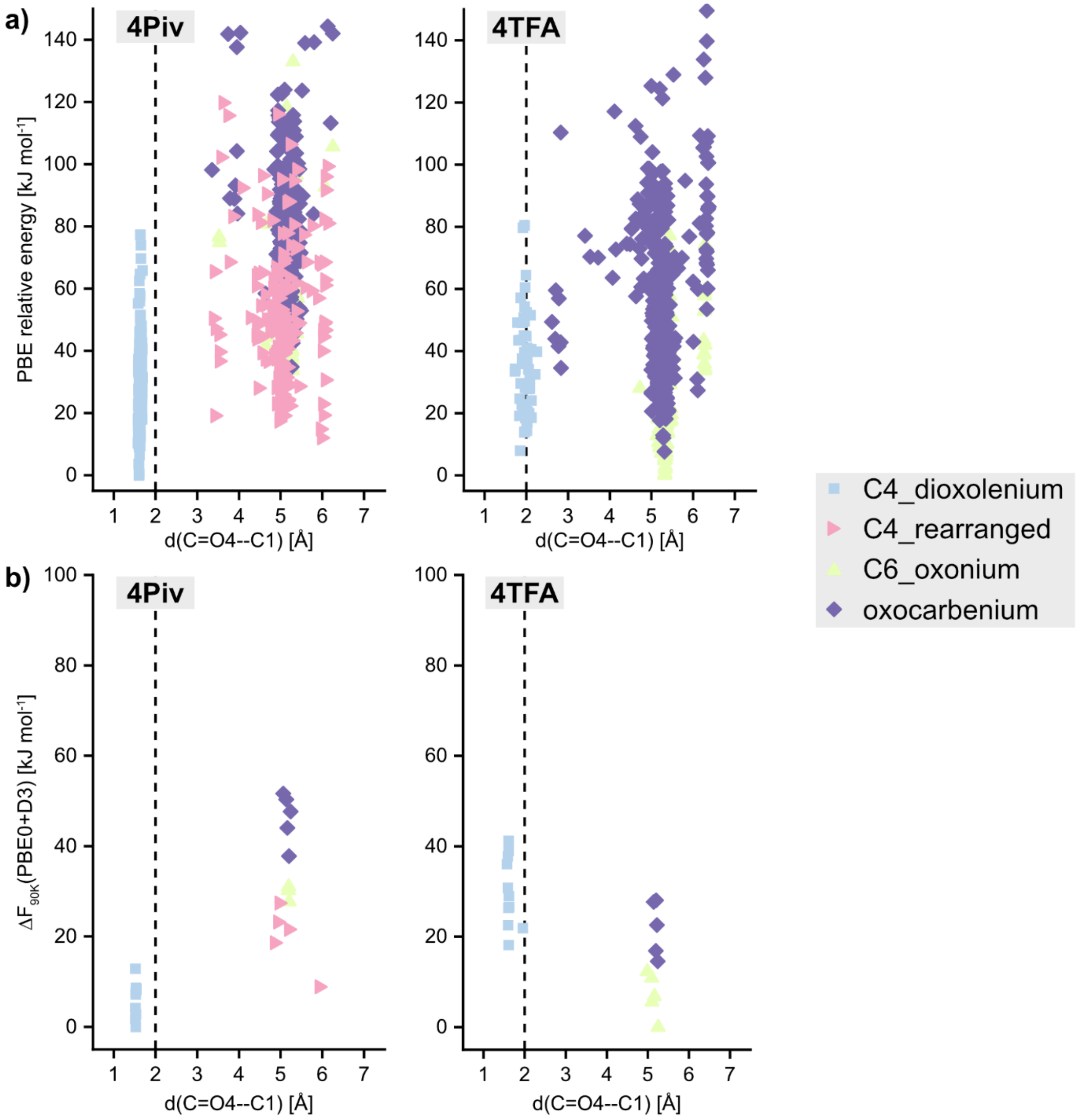

Figure S4. Energy hierarchies of (a) sampled and (b) reoptimized **4Piv** and **4TFA** galactosyl cations as a function of the distance between the carbonyl oxygen of the C4-acyl group and the anomeric carbon (C1). Blue squares indicate dioxolenium-type, red triangles rearranged, yellow triangles oxonium-type, and purple diamond oxocarbenium-type structures. The energetics of sampled and reoptimized galactosyl cations were computed at the PBE+vdW^{TS}/*light* and PBE0+D3/6-311+G(d,p) levels of theory respectively.

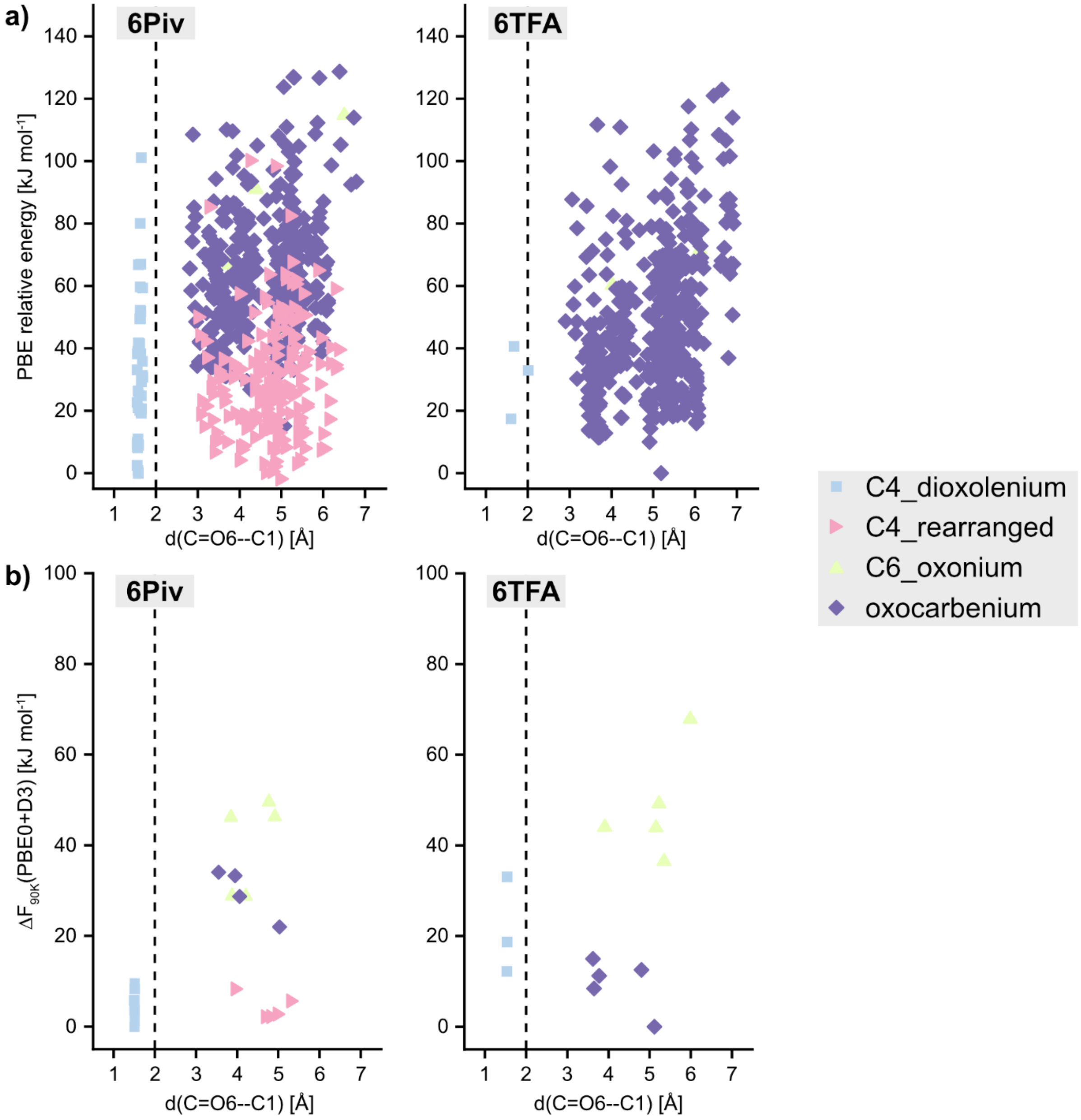

Figure S5. Energy hierarchies of (a) sampled and (b) reoptimized **6Piv** and **6TFA** galactosyl cations as a function of the distance between the carbonyl oxygen of the C6-acyl group and the anomeric carbon (C1). Blue squares indicate dioxolenium-type, red triangles rearranged, yellow triangles oxonium-type, and purple diamond oxocarbenium-type structures. The energetics of sampled and reoptimized galactosyl cations were computed at the PBE+vdW^{TS}/*light* and PBE0+D3/6-311+G(d,p) levels of theory respectively.

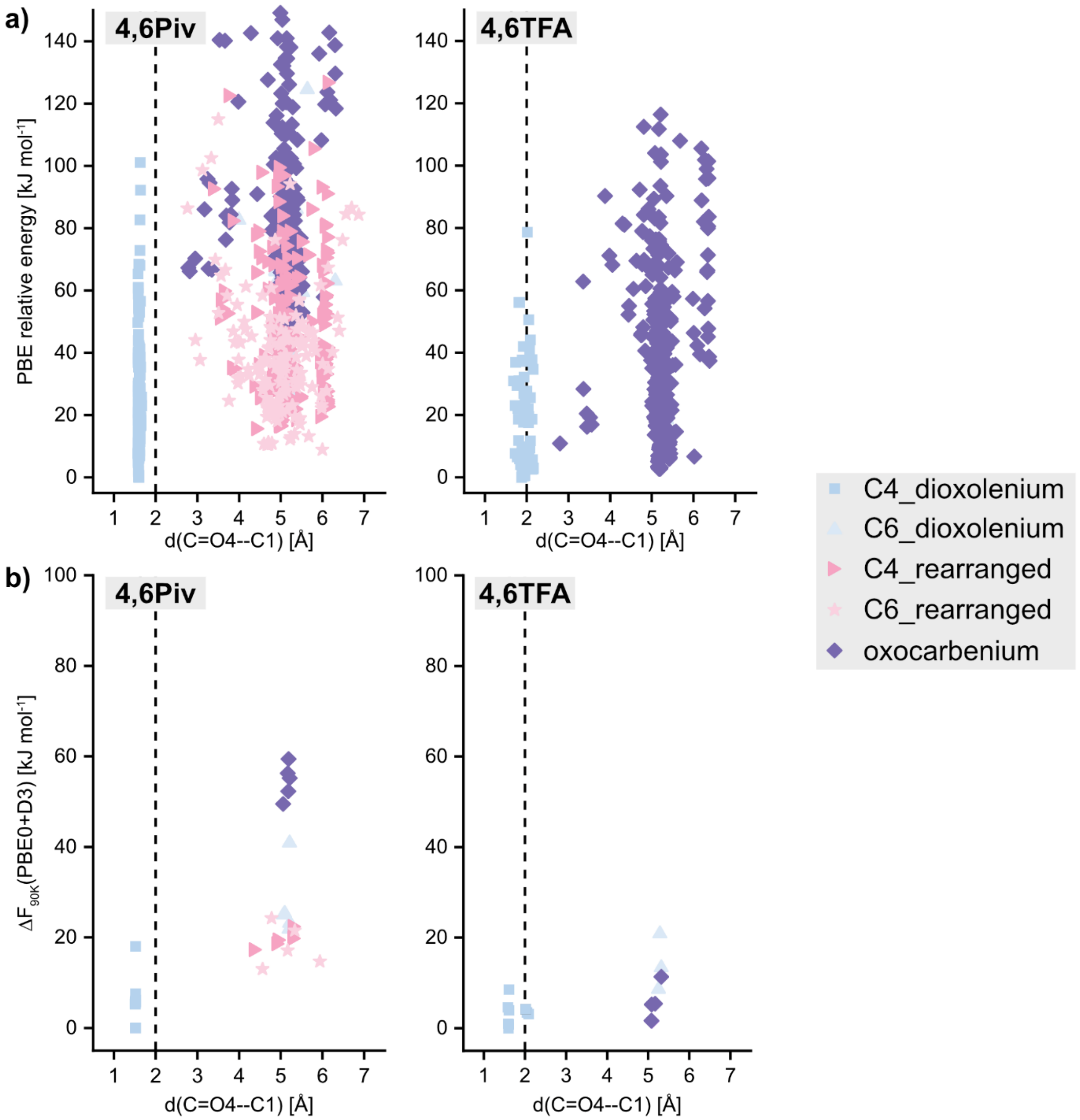

Figure S6. Energy hierarchies of (a) sampled and (b) reoptimized **4,6Piv** and **4,6TFA** galactosyl cations as a function of the distance between the carbonyl oxygen of the C4-acyl group and the anomeric carbon (C1). Blue squares indicate C4_dioxolenium-type, blue triangles C6_dioxolenium-type, red triangles C4_rearranged, red stars C6_rearranged, and purple diamond oxocarbenium-type structures. The energetics of sampled and reoptimized galactosyl cations were computed at the PBE+vdW^{TS}/*light* and PBE0+D3/6-311+G(d,p) levels of theory respectively.

2.4 Energy Diagrams

Transition states (TS) that connect oxocarbenium-type structures with rearranged and dioxolenium-type structures have been computed for **4,6Piv**, **4Piv**, **6Piv**, **4,6TFA**, and **4TFA** glycosyl cations. To find the transition states, relaxed scans of the bonds that form were performed in Gaussian 16. The saddle point of the obtained surfaces was optimized as a transition state and its existence confirmed by a single imaginary frequency. The transition states were connected to the structures in Tables S3-S6 and S8 by intrinsic reaction coordinate (IRC) calculations. The structures in most cases do not correspond to the lowest energy structures for each type of structure. As the conformational space of the probed ions is vast, the existence of multiple transition states that might be more or less favorable cannot be excluded. The structures generated by the IRC calculation can be transformed into their lowest-energy conformers by simple rotation of bonds, which usually requires only a low activation energy. Furthermore, we consider the single-point energy computed at DLPNO-CCSD(T)/Def2-TZVPP level of theory to construct energy diagrams including the ZPVE and free energy from the DFT calculation at PBE0 level of theory (Figures 2, 3 and S7). The energetics indicate that the rearrangement gets favored at higher temperatures, likely due to an entropic contribution. However, the exact temperature of the ions under in-source fragmentation conditions is not known.

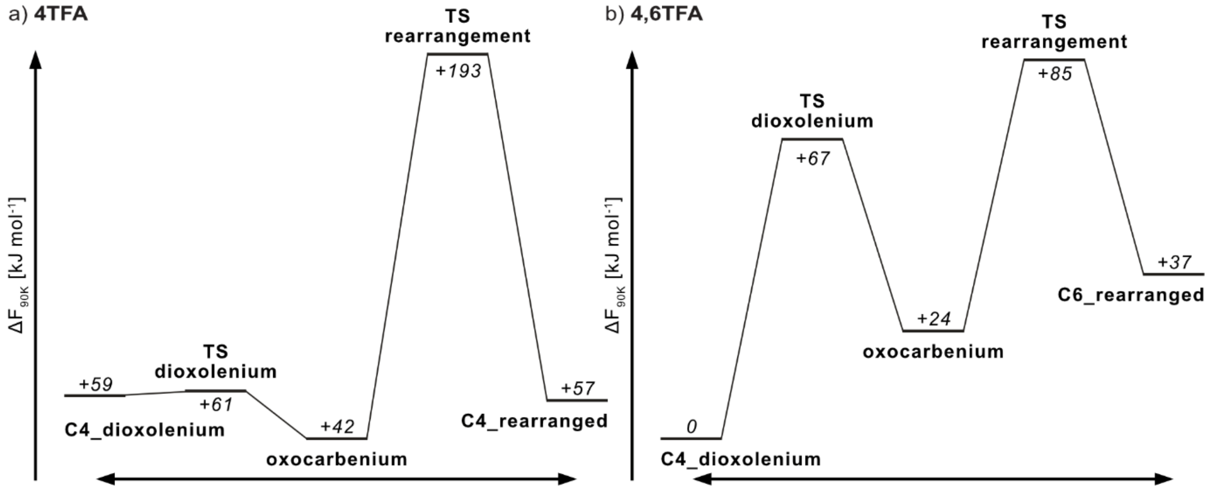

Figure S7. Energy diagrams of (a) **4TFA** and (b) **4,6TFA** galactosyl cations. Surfaces of formation of dioxolenium-type and rearranged structures from oxocarbenium-type structures are shown. As these galactosyl cations are not formed, the rearrangement and participation processes are purely hypothetical. Generally, the energetics indicate that these cations are less prone to engage in remote participation or rearrangement.

2.5 3D Structures

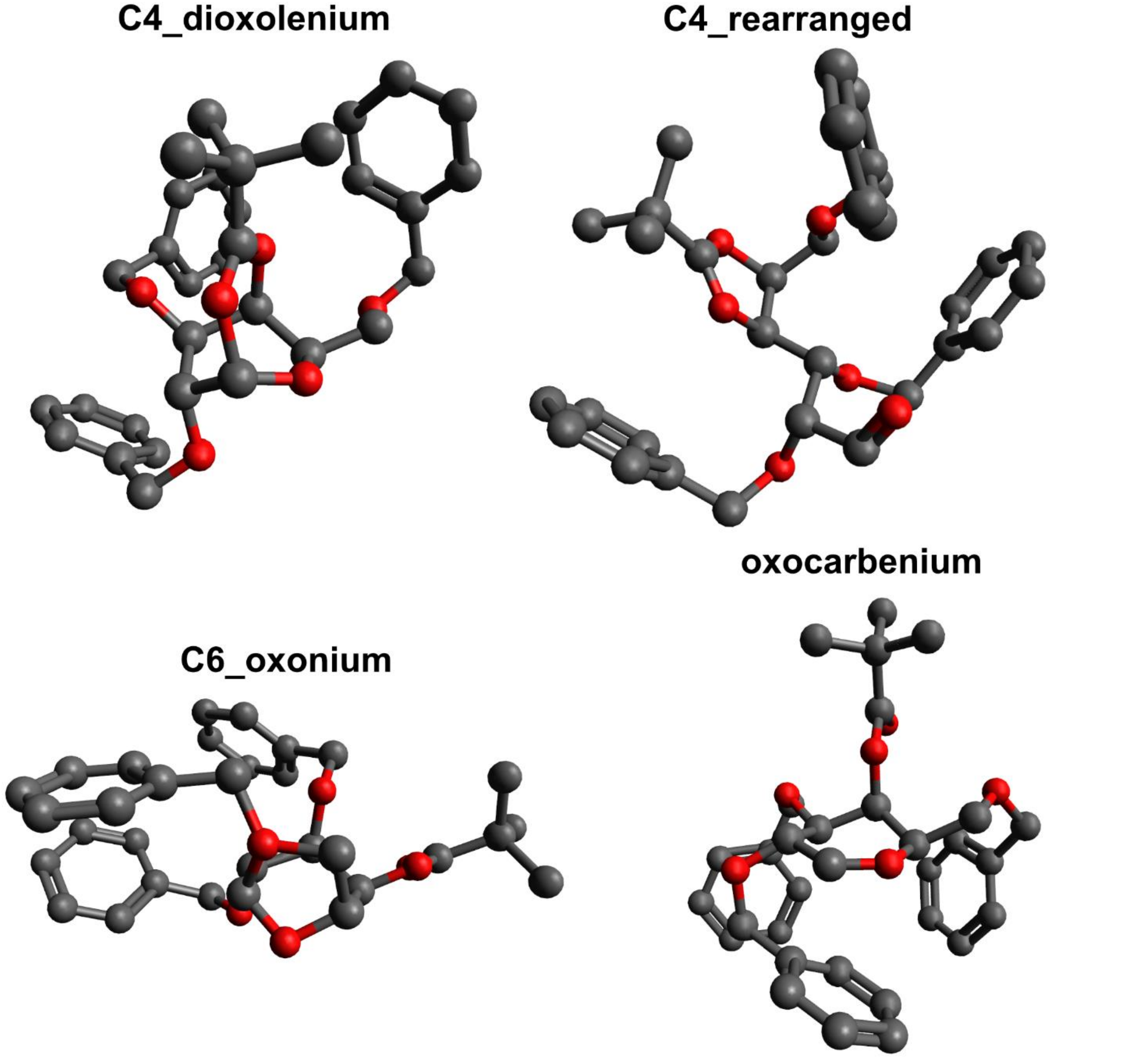

Figure S8. Reoptimized lowest-energy structures for intact and rearranged **4Piv** glycosyl cations for each structural motif. Hydrogen atoms are omitted for clarity.

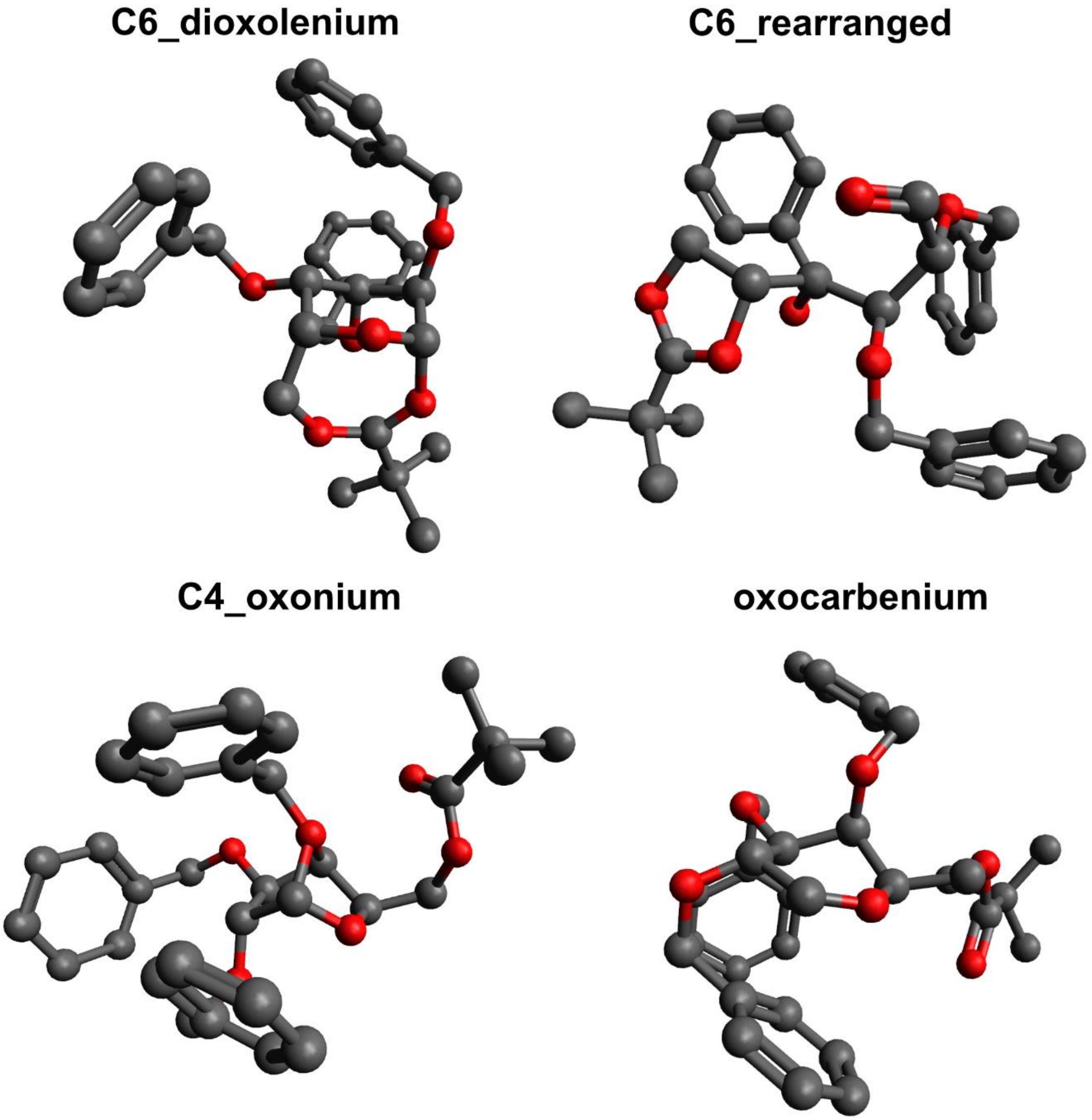

Figure S9. Reoptimized lowest-energy structures for intact and rearranged **6Piv** glycosyl cations for each structural motif. Hydrogen atoms are omitted for clarity.

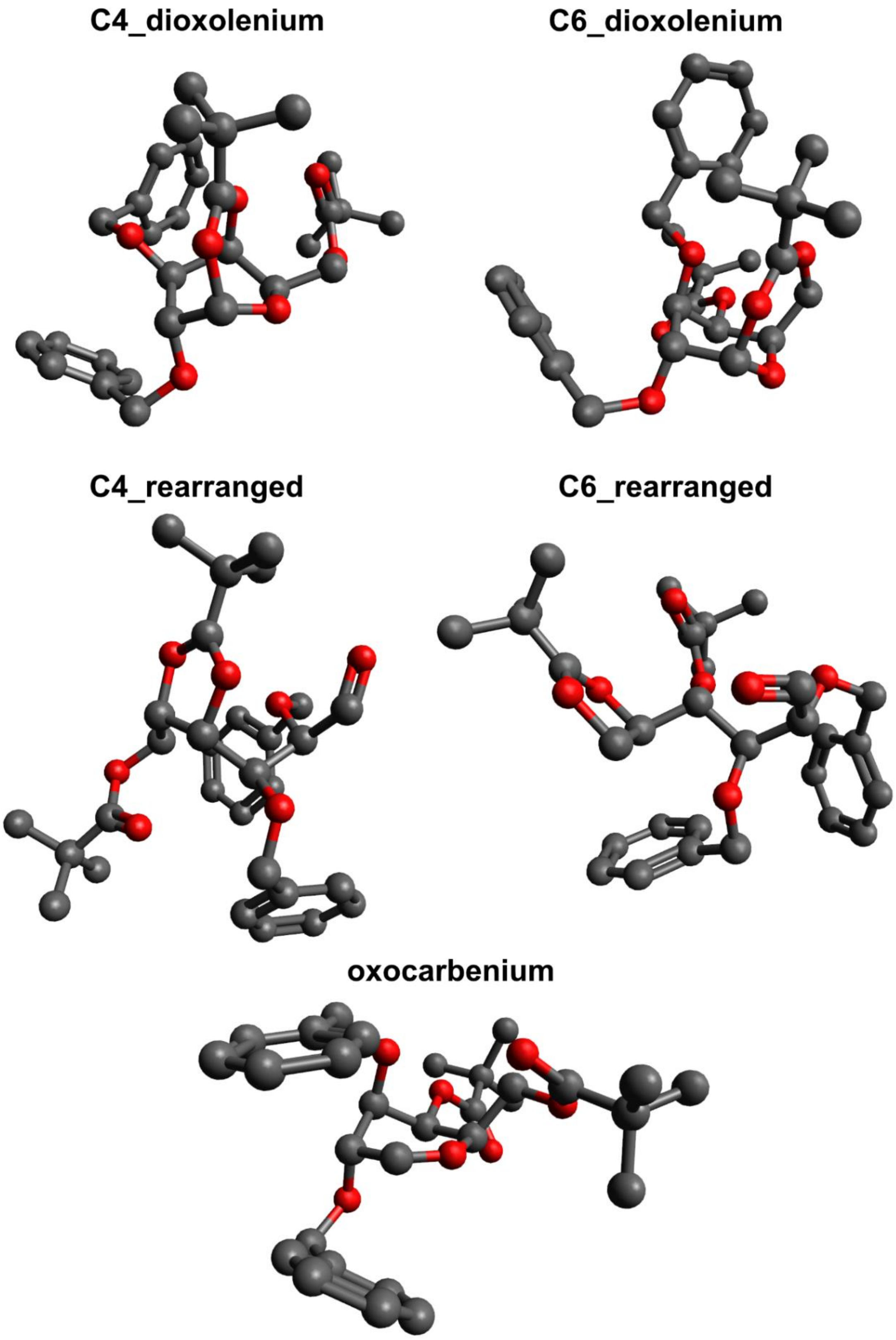

Figure S10. Reoptimized lowest-energy structures for intact and rearranged **4,6Piv** glycosyl cations for each structural motif. Hydrogen atoms are omitted for clarity.

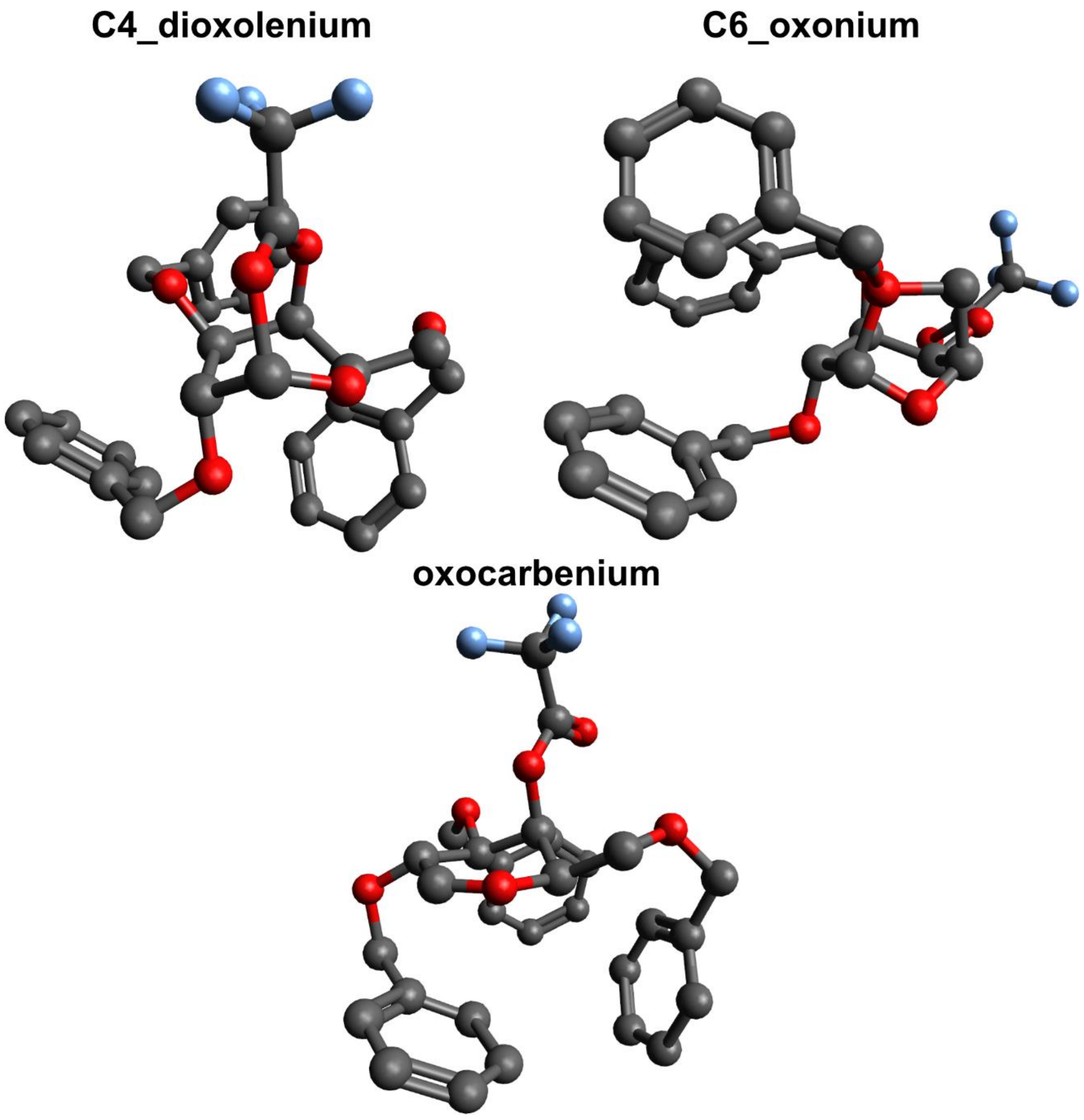

Figure S11. Reoptimized lowest-energy structures for intact **4TFA** glycosyl cations for each structural motif. Hydrogen atoms are omitted for clarity.

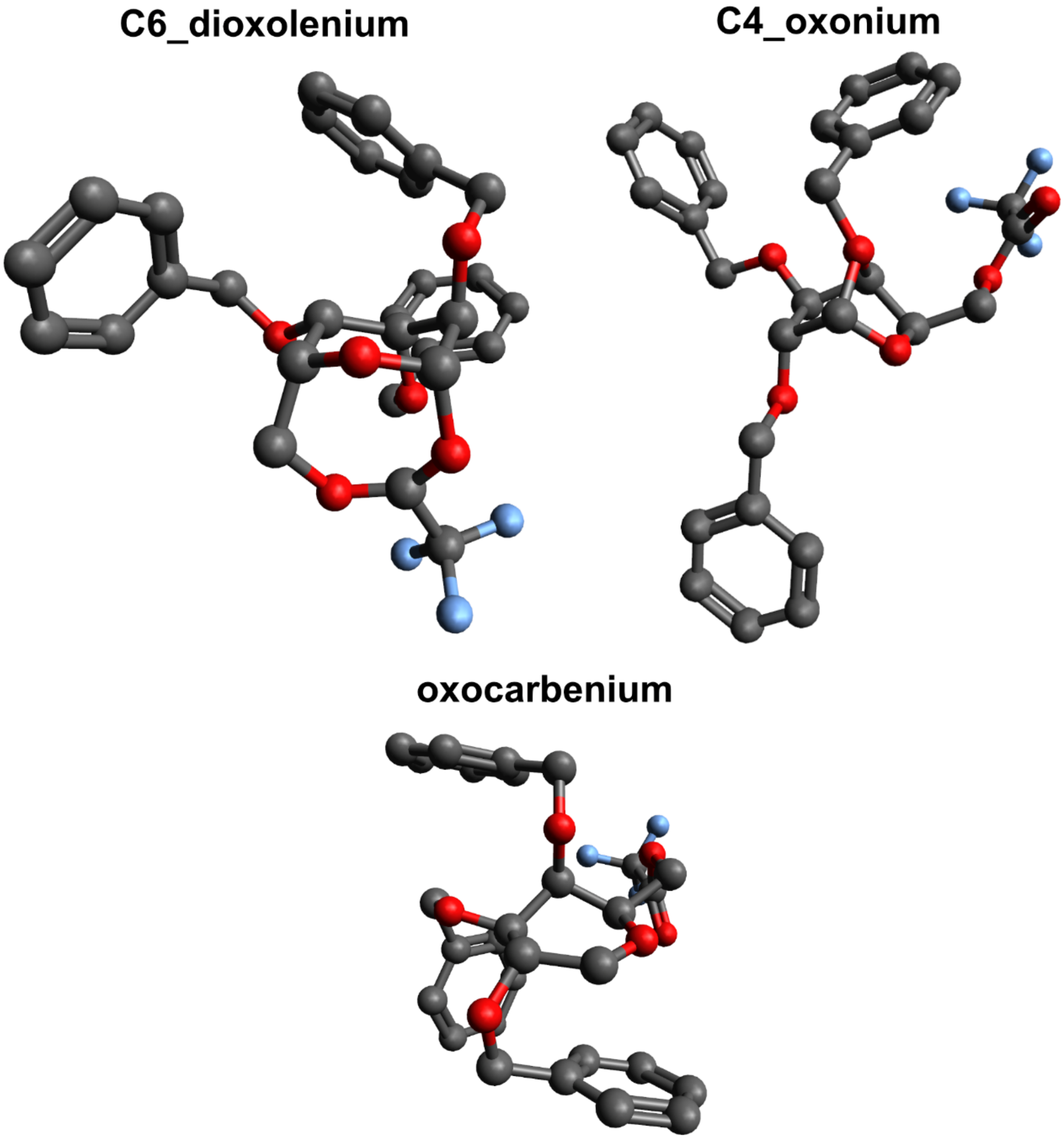

Figure S12. Reoptimized lowest-energy structures for intact **6TFA** glycosyl cations for each structural motif. Hydrogen atoms are omitted for clarity.

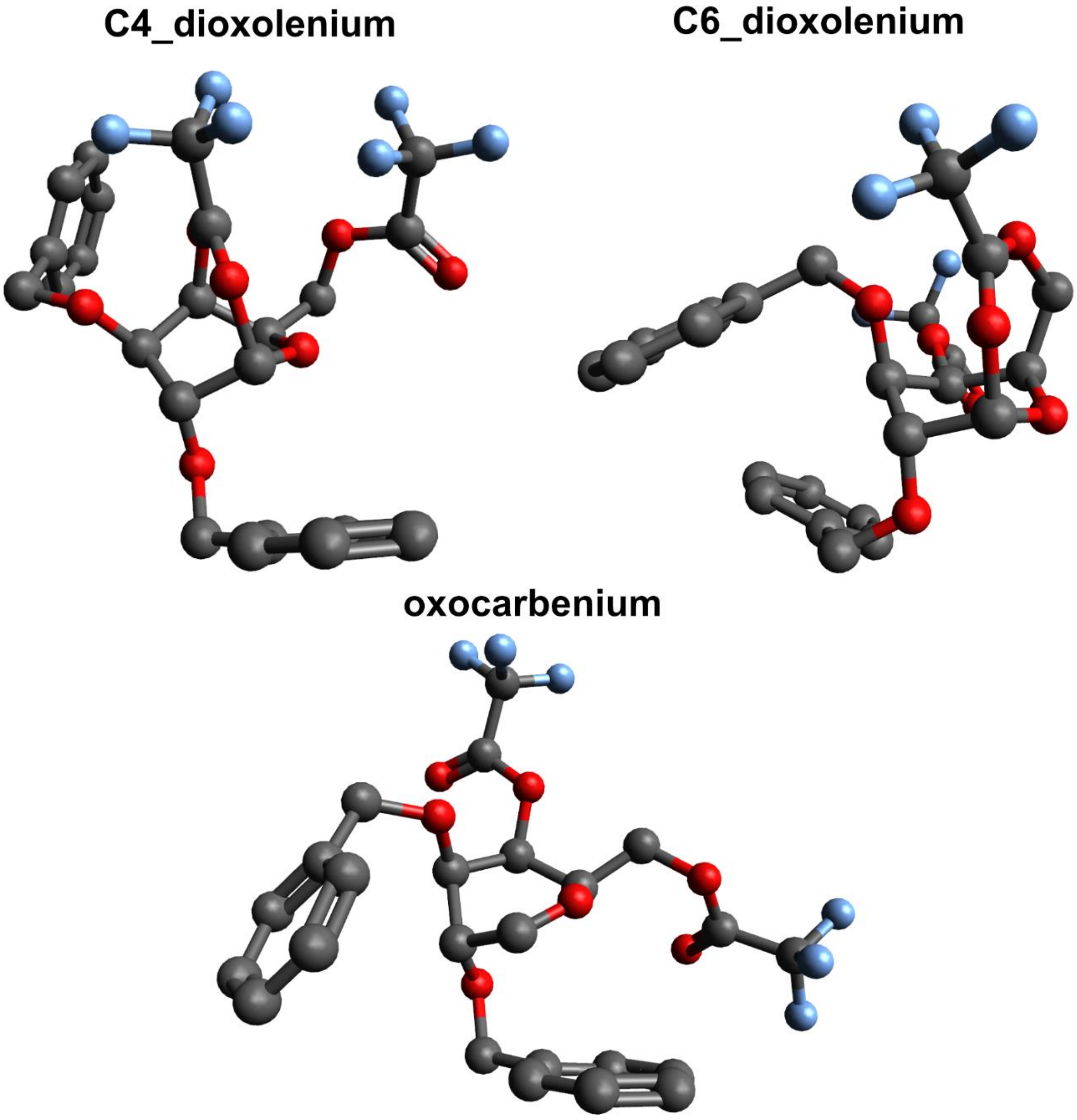

Figure S13. Reoptimized lowest-energy structures for intact **4,6TFA** glycosyl cations for each structural motif. Hydrogen atoms are omitted for clarity.

2.6 xyz-Coordinates of reoptimized structures

xyz-Coordinates of all reoptimized geometries can be found in a separate document “coordinates.xyz”.

3 General Information

All **chemicals** were reagent grade and used as supplied unless otherwise noted. All **solvents** for chemical reactions were commercially purchased in p.a. quality. If stated, they were dried in a Solvent Dispensing System (J.C. Meyer). For HPLC and MS spectrometry, solvents with corresponding quality were used. Water was used from a Milli Q-station from Millipore. **The automated syntheses** were performed on a home-built synthesizer developed at the Max Planck Institute of Colloids and Interfaces.

Reaction completion, identity, and purity of all compounds were determined by low resolution mass spectrometry (**ESI-LRMS**) or analytical thin-layer chromatography (**TLC**). TLC was performed on Merck silica gel 60 F_{254} plates (0.25 mm). Compounds were visualized by UV irradiation (254 nm) or stained (5% sulfuric acid in ethanol or Hanessian's Stain: 235 mL of distilled water, 12 g of ammonium molybdate, 0.5 g of ceric ammonium molybdate, and 15 mL sulfuric acid). **Flash column chromatography** was performed on Kieselgel 60 with 230-400 mesh (Sigma-Aldrich, St. Louis, USA). Analysis and purification by normal and reverse phase **HPLC** and ESI-LRMS was performed by using an Agilent 1200 series. ^{1}H, ^{13}C, COSY and HSQC **NMR spectra** were recorded in parts per million (δ) relative to the resonance of the solvent on a Varian 400-MR (400 MHz), Varian 600-MR (600 MHz), or Bruker Biospin AVANCE700 (700 MHz) spectrometer. Assignments were supported by COSY and HSQC experiments. High resolution mass spectra (**HRMS**) were obtained using 6210 ESI-TOF mass spectrometer (Agilent) and **MALDI-TOF** autoflexTM (Bruker) instruments.

4 Materials and Conditions for Automated Synthesis

4.1 Materials and Measurements

Solvents used for dissolving all building blocks and making of various solutions were taken from Solvent Dispensing System (J.C. Meyer). Wash solvents were HPLC grade. Prior to automated synthesis, the building blocks were weighed and co-evaporated three times with anhydrous toluene and dried for at least one hour under high vacuum prior to use. All solutions were freshly prepared in oven-dried, argon-flushed glassware and kept under argon during the automation process. Isolated product yields were calculated on the basis of resin loading. Functionalized resin **2** was synthesized as previously reported[13] and resin loading (0.40 mmol/g) was determined following a published protocol.[14] Resin was placed in the reaction vessel and was swollen in

dichloromethane for 20 min at room temperature before starting the first module. During this time, all reagent lines involved in the synthesis were washed and primed.

4.2 Preparation of Stock Solutions

Building Block Solution: Glycosyl phosphate building block (0.07 mmol, 4.7 equiv. per cycle) was dissolved in 1 mL (per cycle) of anhydrous CH_2Cl_2.

Activator Solution/Acidic Wash Solution: TMSOTf (0.9 mL, 0.62 mmol) was added to 40 mL of anhydrous CH_2Cl_2.

Pre-Capping Solution: Pyridine (10 mL) was added to 90 mL of DMF.

Capping Solution: Methanesulfonic acid (1.2 mL, 18.5 mmol), acetic anhydride (6 mL, 63.5 mmol) were added to 50 mL of anhydrous CH_2Cl_2.

Fmoc Deprotection Solution: Piperidine (20 mL) was added to 80 mL anhydrous DMF.

4.3 Modules for Automated Synthesis

Initiation: The resin **2** is loaded in the reaction vessel and washed with DMF, THF, and CH_2Cl_2 (3 x 3 mL for 15 s, respectively). The resin is then swollen in 2 mL CH_2Cl_2 for 20 minutes while the temperature of the reaction vessel is cooled to the lowest temperature required throughout the synthesis. During this time, all reagent lines needed for the synthesis are washed and primed.

Module I - Acidic Washing: Once the temperature of the reaction vessel has adjusted to the desired temperature of the subsequent glycosylation, 1 mL of the **Acidic Wash Solution** is delivered to the reaction vessel. After bubbling for three minutes, the solution is drained. Finally, the resin is washed with 3 mL CH_2Cl_2 for 25 s and drained.

Module II – Glycosylation (for glycosyl phosphate): Upon draining the CH_2Cl_2 in the reaction vessel, **Building Block Solution** (1 mL) containing the appropriate building block is delivered from the building block storing component to the reaction vessel. After the temperature again reaches the desired temperature (T_1), **Activator Solution** (1 mL) is delivered to the reaction vessel from the respective activator storing component to the reaction vessel. The glycosylation mixture is incubated for the selected duration (t_1) at the desired T_1, then the reaction temperature is ramped to T_2. Once T_2 is reached, it is maintained and the reaction mixture is incubated for an additional time (t_2). Once the incubation time is finished, the reaction mixture is

drained and the resin is washed with CH_2Cl_2 (2 x 2 mL for 25 s). The temperature of the reaction vessel is increased to 25 °C for the next module.

Module III - Capping: The resin is washed with DMF (2 x 2 mL for 25 s). Then **Pre-capping Solution** (2 mL) is delivered at 25 °C. After 1 min, the reaction solution is drained and the resin is washed with CH_2Cl_2 (3 x 3 mL for 25 s). Upon washing, **Capping Solution** (4 mL) is delivered and the temperature is maintained at 25 °C. The resin and the reagents are incubated for 20 min. The solution is then drained from the reactor vessel and the resin is washed with CH_2Cl_2 (3 x 3 mL for 25 s).

Module IV - Fmoc Deprotection: The resin is first washed with DMF (3 x 2 mL for 25 s), and then **Fmoc Deprotection Solution** (2 mL) is delivered to the reaction vessel at 25 °C. After 5 min, the reaction solution is drained and the resin is washed with DMF (3 x 3 mL for 25 s) and CH_2Cl_2 (5 x 2 mL for 25 s). Then, the temperature of the reaction vessel is decreased to T_1 for the next module.

4.4 Post-automated Synthesis Manipulations, Analysis and Purification

Cleavage from Solid Support (Method A)

After automated synthesis, the resin was removed from the reaction vessel, suspended in CH_2Cl_2 (20 mL), and photocleaved in a continuous-flow photoreactor. A Vapourtec E-Series easy-MedCHem, equipped with a UV-150 Photochemical reactor having a UV-150 Medium-Pressure Mercury Lamp (arc length 27.9 cm, 450 W) surrounded by a long-pass UV filter (Pyrex, 50% transmittance at 305 nm) was used. A Pump 11 Elite Series (Harvard Apparatus syringe pump at a flow rate of 0.8 mL/min was used to pump the mixture through a FEP tubing (i.d. 3.0 inch, volume: 12 mL) at 20 °C. The reactor was washed with 20 mL CH_2Cl_2 at a flow rate of 2.0 mL/min. The output solution was filtered to remove the resin and the solvent was evaporated *in vacuo*. Crude was then analyzed by MALDI.

Analytical NP-HPLC of Crude Material (Method B-1a)

Analytical NP-HPLC was conducted on an Agilent 1200 Series system. A YMC-Diol-300-NP column (150 mm x 4.60 mm I.D.) was used at a flow rate of 1.00 mL/min with hexane/EtOAc as eluent (20% EtOAc in hexane for 5 min, 20 → 55% EtOAc in hexane over 35 min, 55 → 100% EtOAc in hexane over 35 min, 100% EtOAc for 10 min).

Preparative NP-HPLC of Crude Material (Method B-1b)

Preparative NP-HPLC was conducted on an Agilent 1200 Series system. A YMC-Diol-300-NP column (150 mm x 20 mm I.D.) was used at a flow rate of 15.00 mL/min with hexane/EtOAc as eluent (20% EtOAc in hexane for 5 min, 20 → 55% EtOAc in hexane over 35 min, 100% EtOAc for 10 min).

4.5 General Procedure for Glycosylations

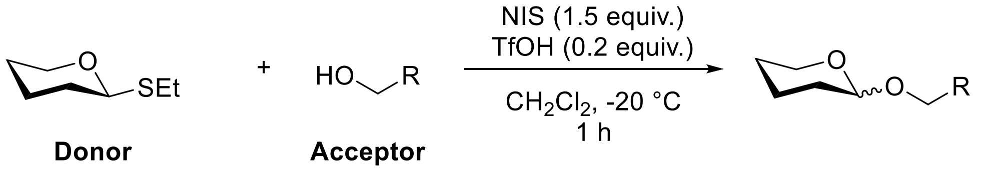

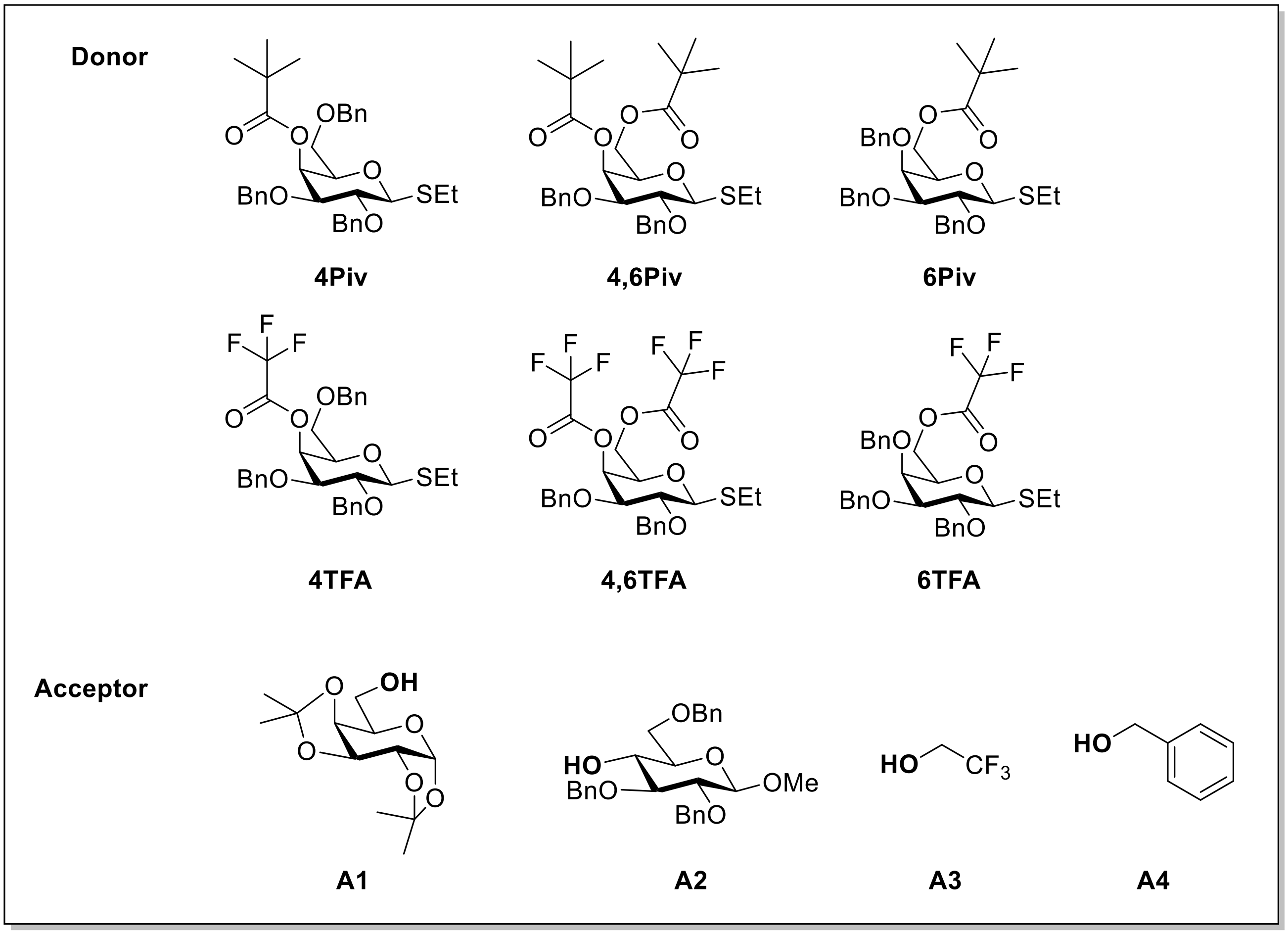

Donor (35 μmol, 1.0 equiv.) and the acceptor **A1** or **A2** (35 μmol, 1.0 equiv.) were co-evaporated with anhydrous toluene (3 x 2 mL) and kept under high vacuum for one hour. Anhydrous CH_2Cl_2 (2 mL) was added and if acceptor **A1** or **A2** were not used, acceptor **A3** or **A4** (175 μmol, 5 equiv.) was added and the mixture

was stirred over activated molecular sieves (3 Å-AW) for 30 minutes at room temperature. The solution was cooled to -20 °C and NIS (52 μmol, 1.5 equiv.) was added followed by TfOH (60 μL of a 1% solution in CH_2Cl_2, 7 μmol, 0.2 equiv.) and the mixture was stirred for 1 h at -20 °C. The reaction mixture was quenched with pyridine, diluted with CH_2Cl_2, filtered and was then washed with 10% $Na_2S_2O_3$ (10 mL). The aqueous phase was extracted with CH_2Cl_2 (3 x 10 mL), dried over Na_2SO_4 and concentrated. The residue was purified by HPLC using **Method B-2**.

4.6 Determination of Alpha/Beta Ratios and Purification

Alpha/beta ratios were determined using HPLC and $^1H/^{13}C$/HSQC NMR spectroscopy.

Analytical NP-HPLC for alpha/beta ratio determination (Method B-2a)

Analytical NP-HPLC was conducted on an Agilent 1200 Series system. A YMC-Diol-300-NP column (150 mm x 4.600 mm I.D.) was used with a flow rate of 1.00 mL/min and hexanes/EtOAc as eluent (16 min linear gradient 2 to 25% EtOAc in hexanes, 2 min linear gradient 25 to 70% EtOAc in hexanes, 2 min isocratic 70% EtOAc).

Preparative NP-HPLC of Crude Material (Method B-2b)

Preparative NP-HPLC was conducted on an Agilent 1200 Series system. A YMC-Diol-300-NP column (150 mm x 20 mm I.D.) was used at a flow rate of 15.00 mL/min with hexane/EtOAc as eluent (16 min linear gradient 2 to 25% EtOAc in hexanes, 2 min linear gradient 25 to 70% EtOAc in hexanes, 2 min isocratic 70% EtOAc).

5 Synthesis of building blocks

4Ac and **Bn** galactose building blocks were synthesized according to previously published procedures.[15-16]

Ethyl 2,3-bis-*O*-benzyl-4,6-O-[(S)-phenylmethylene]-1-thio-β-D-galactopyranoside[17] (S2)

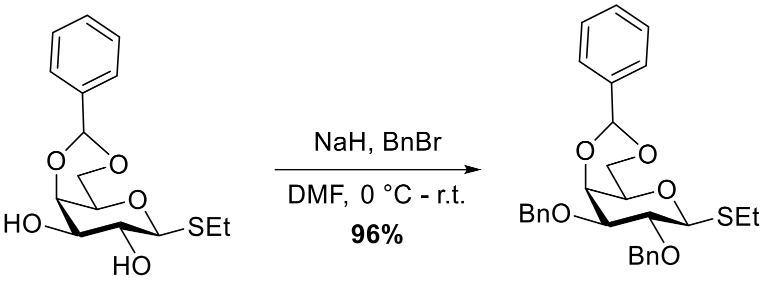

S1 **S2**

Galactose building block **S1** (2.5 g, 8.00 mmol, 1.0 eq.) was dissolved in anhydrous DMF (15 mL). The stirred solution was cooled to 0 °C and sodium hydride (1.5 g, 37.61 mmol; 60% dispersion in mineral oil, 4.7 eq.) was added in small portions. After 30 min, benzyl bromide (2.85 mL, 24.01 mmol, 3.0 eq.) was added dropwise. The reaction mixture was allowed to warm up to room temperature and was stirred overnight. Methanol (10 mL) was added, the reaction mixture was stirred for 10 min and afterwards diluted with EtOAc (50 mL). The organic layer was washed with water (2 x 30 mL). The aqueous phase was extracted with ethyl acetate (2 x 40 mL). The combined organic phase was washed with water (30 mL) followed by brine (30 mL), dried over Na_2SO_4 and concentrated. Product **S2** (3.8 g, 7.71 mmol, **96%**) was obtained as a colorless syrup after purification by column chromatography (SiO_2, Hex/EtOAc = 9:1).

^{1}H NMR (400 MHz, $CDCl_3$) δ 7.58 – 7.51 (m, 2H), 7.46 – 7.28 (m, 13H), 5.48 (s, 1H), 4.93 – 4.83 (m, 2H), 4.76 (d, *J* = 2.0 Hz, 2H), 4.44 (d, *J* = 9.6 Hz, 1H), 4.31 (dd, *J* = 12.4, 1.6 Hz, 1H), 4.16 (dd, *J* = 3.6, 1.1 Hz, 1H), 3.97 (dd, *J* = 12.4, 1.8 Hz, 1H), 3.90 (t, *J* = 9.4 Hz, 1H), 3.60 (dd, *J* = 9.2, 3.5 Hz, 1H), 3.36 (q, *J* = 1.5 Hz, 1H), 2.92 – 2.70 (m, 2H), 1.34 (t, *J* = 7.5 Hz, 3H) ppm.

^{13}C NMR (101 MHz, $CDCl_3$) δ 138.5, 138.4, 138.0, 129.2, 128.5, 128.5, 128.3, 127.9, 127.9, 126.7, 101.6, 84.5, 81.1, 77.0, 75.9, 74.1, 71.9, 69.9, 69.5, 23.9, 15.2 ppm.

^{1}H NMR (400 MHz, $CDCl_3$) of **S2**:

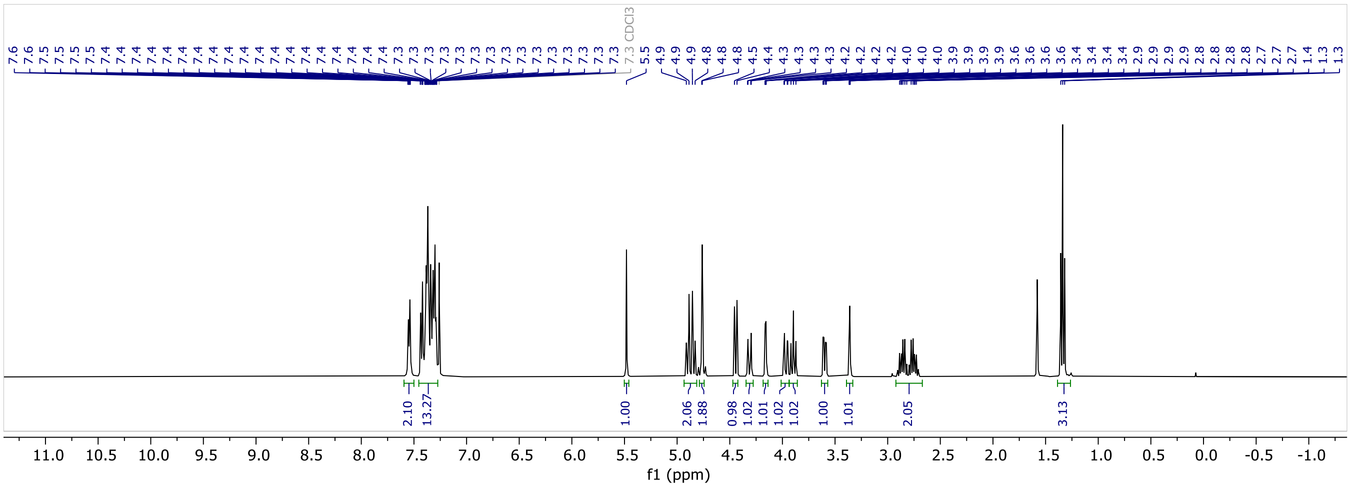

^{13}C NMR (101 MHz, $CDCl_3$) of **S2**:

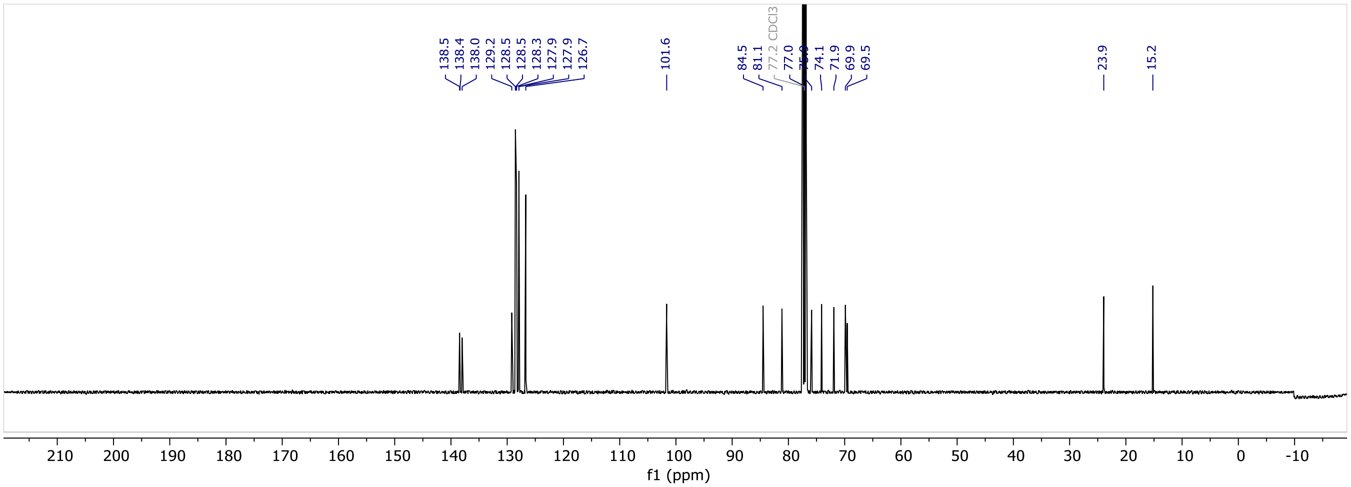

Ethyl 2,3-bis-*O*-benzyl-1-thio-β-D-galactopyranoside[18] (S3)

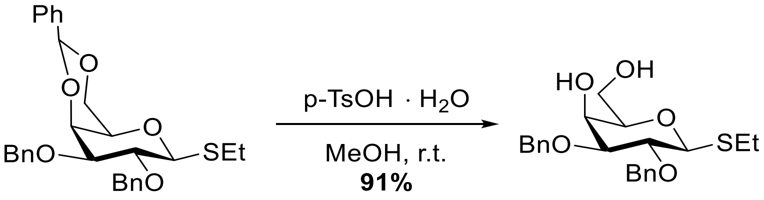

S2 **S3**

A mixture of **S2** (750 mg, 1.52 mmol, 1.0 equiv.) and *p*-TsOH·H_2O (290 mg, 1.52 mmol, 1.0 equiv.) in MeOH (30 mL) was stirred at room temperature for 5 h. The mixture was diluted with ethyl acetate (100 mL) and was washed with saturated aqueous $NaHCO_3$ (2 x 50 mL) and brine (50 mL). The organic layer was dried over

Na_2SO_4, filtered and concentrated. Product **S3** (560 mg, 1.38 mmol, 91%) was obtained as a white solid after purification by column chromatography (SiO_2, Hex/EtOAc = 8:2 to 1:1).

R*f* = 0.23 (Hex/EtOAc 8:2).

^{1}H NMR (400 MHz, $CDCl_3$) δ 7.44 – 7.27 (m, 10H), 4.92 – 4.74 (m, 2H), 4.73 (s, 2H), 4.44 (d, *J* = 9.7 Hz, 1H), 4.05 (dt, *J* = 3.3, 1.5 Hz, 1H), 4.00 – 3.74 (m, 2H), 3.67 (t, *J* = 9.3 Hz, 1H), 3.56 (dd, *J* = 9.0, 3.3 Hz, 1H), 3.52 – 3.45 (m, 1H), 2.86 – 2.69 (m, 2H), 2.66 (s, 1H), 2.19 (s, 1H), 1.32 (t, *J* = 7.4 Hz, 3H) ppm.

^{13}C NMR (101 MHz, $CDCl_3$) δ 138.17, 137.72, 128.72, 128.52, 128.49, 128.21, 128.02, 128.00, 85.30, 82.28, 77.94, 77.88, 75.98, 72.38, 67.58, 62.93, 25.00, 15.25 ppm.

^{1}H NMR (400 MHz, $CDCl_3$) of **S3**:

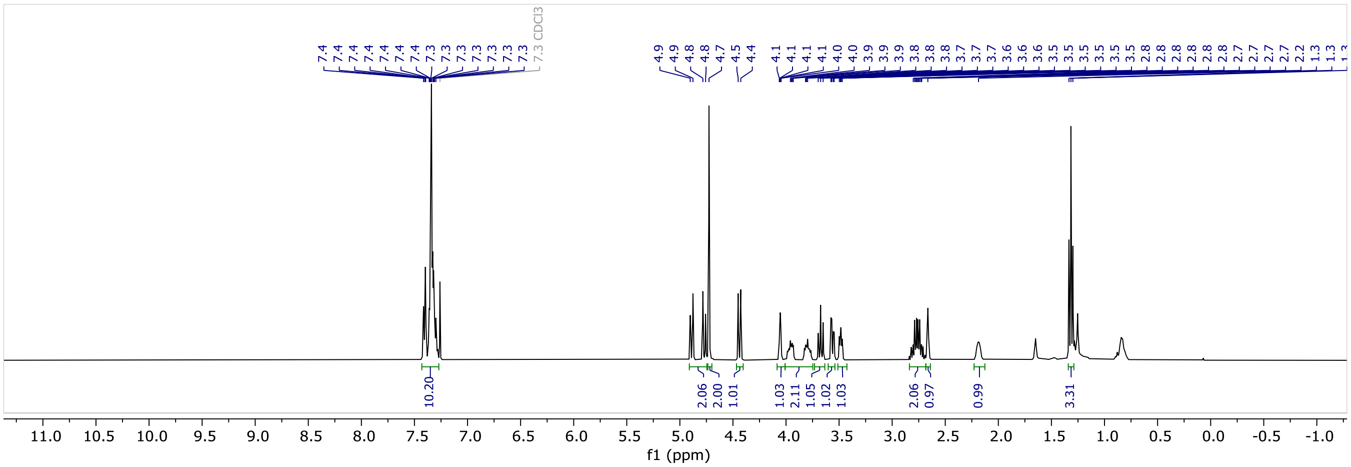

^{13}C NMR (101 MHz, $CDCl_3$) of **S3**:

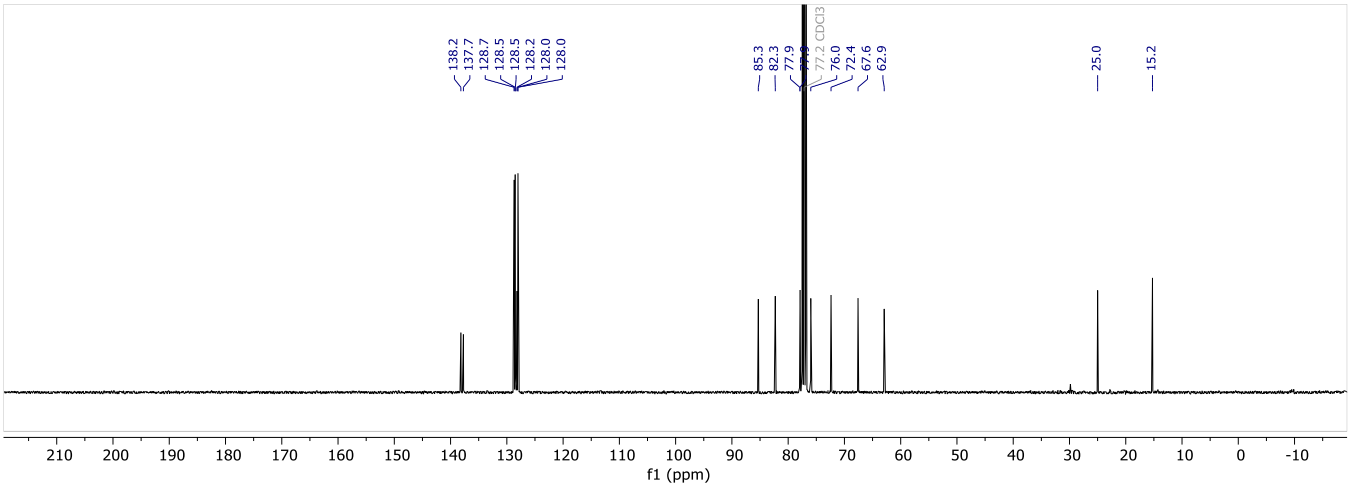

Ethyl 2,3,4-tris-*O*-benzyl-1-thio-β-D-galactopyranoside[19] (S4)

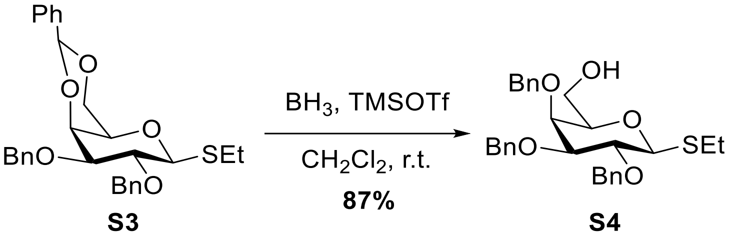

To a solution of **S3** (750 mg, 1.52 mmol, 1.0 equiv.) in anhydrous CH_2Cl_2 (10 mL) was added BH_3 (1 M solution in THF, 7.6 mL, 7.61 mmol, 5.0 equiv.) and TMSOTf (41 μL, 0.23 mmol, 0.15 equiv.). The mixture was stirred under argon atmosphere at room temperature for 4 h. Et_3N (1 mL) was added followed by MeOH until the evolution of H_2 ceased. The mixture was concentrated and coevaporated with MeOH (3 x 30 mL). Product **S4** (650 mg, 1.31 mmol, 87%) was obtained as a white solid after purification by column chromatography (Hex/EtOAc 3:1).

$\mathbf{R}_f$ = 0.19 (Hex/EtOAc 3:1).

^{1}H NMR (400 MHz, $CDCl_3$) δ 7.43 – 7.26 (m, 15H), 5.01 – 4.60 (m, 6H), 4.43 (d, *J* = 9.6 Hz, 1H), 3.89 – 3.80 (m, 2H), 3.81 – 3.74 (m, 1H), 3.58 (dd, *J* = 9.2, 2.8 Hz, 1H), 3.52 – 3.37 (m, 2H), 2.84 – 2.65 (m, 2H), 1.57 (s, 1H), 1.30 (t, *J* = 7.4 Hz, 3H) ppm.

^{13}C NMR (101 MHz, $CDCl_3$) δ 138.42, 138.37, 138.35, 128.65, 128.59, 128.55, 128.49, 128.08, 127.93, 127.77, 85.61, 84.31, 78.74, 78.66, 75.96, 74.21, 73.25, 73.08, 62.35, 25.06, 15.26 ppm.

^{1}H NMR (400 MHz, $CDCl_3$) of **S4**:

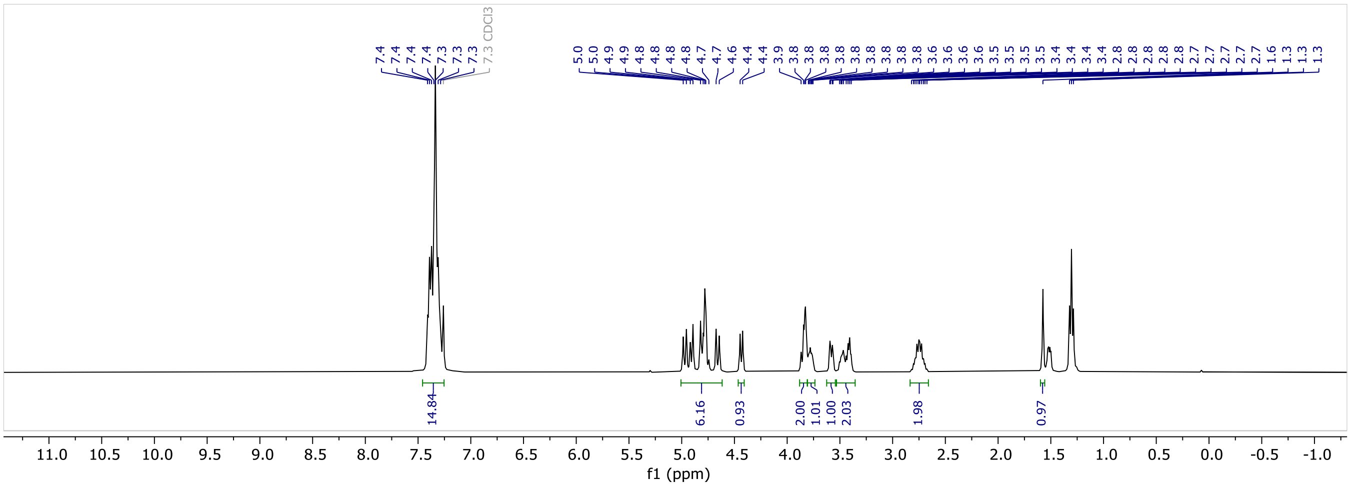

^{13}C NMR (101 MHz, $CDCl_3$) of **S4**:

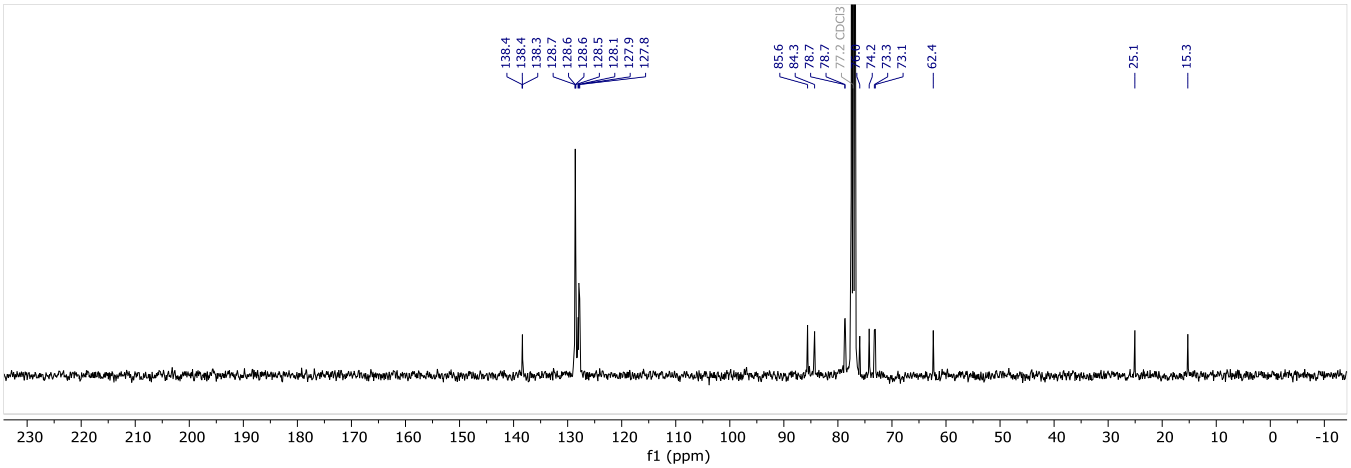

Ethyl 2,3,6-tris-*O*-benzyl-1-thio-β-D-galactopyranoside[20] (S5)

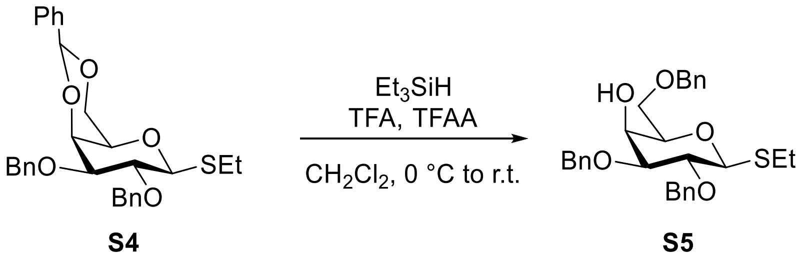

Compound **S4** (1.00 g, 2.03 mmol, 1.0 equiv.) was co-evaporated with anhydrous toluene (2 x 3 mL), and dissolved in anhydrous CH_2Cl_2 (10 mL). Triethylsilane (1.93 mL, 12.18 mmol, 6.0 equiv.) and trifluoroacetic anhydride (0.29 mL, 2.03 mmol, 1.00 equiv.) were added and the solution was cooled to 0 °C. Trifluoroacetic acid (0.93 mL, 12.18 mmol, 6.00 equiv.) was added dropwise. The mixture was allowed to warm up to room temperature and was stirred for 5 h. The solution was diluted with CH_2Cl_2 and quenched with saturated aqueous $NaHCO_3$ (20 mL). The aqueous phase was extracted with CH_2Cl_2 (2 x 30 mL) and the combined organic phase was washed with water (30 mL), dried over Na_2SO_4, filtered and concentrated. Product **S5** (800 mg, 1.62 mmol, 80%) was obtained as a colorless syrup after purification by column chromatography (SiO_2, Hex/EtOAc = 9:1 to 7:3).

$\mathbf{R}_f$ = 0.48 (Hex/EtOAc 7:3).

^{1}**H NMR** (400 MHz, $CDCl_3$) δ 7.43 – 7.28 (m, 15H), 4.90 – 4.67 (m, 4H), 4.58 (s, 2H), 4.43 (d, *J* = 9.7 Hz, 1H), 4.10 (s, 1H), 3.83 – 3.63 (m, 3H), 3.61 – 3.51 (m, 2H), 2.84 – 2.68 (m, 2H), 2.52 (s, 1H), 1.31 (t, *J* = 7.5 Hz, 3H) ppm.

^{13}C NMR (101 MHz, $CDCl_3$) δ 138.22, 138.00, 137.85, 128.79, 128.67, 128.58, 128.51, 128.50, 128.11, 128.00, 127.95, 127.93, 85.18, 82.46, 77.98, 76.96, 75.98, 73.85, 72.20, 69.41, 66.98, 24.91, 15.26 ppm.

^{1}H NMR (400 MHz, $CDCl_3$) of **S5**:

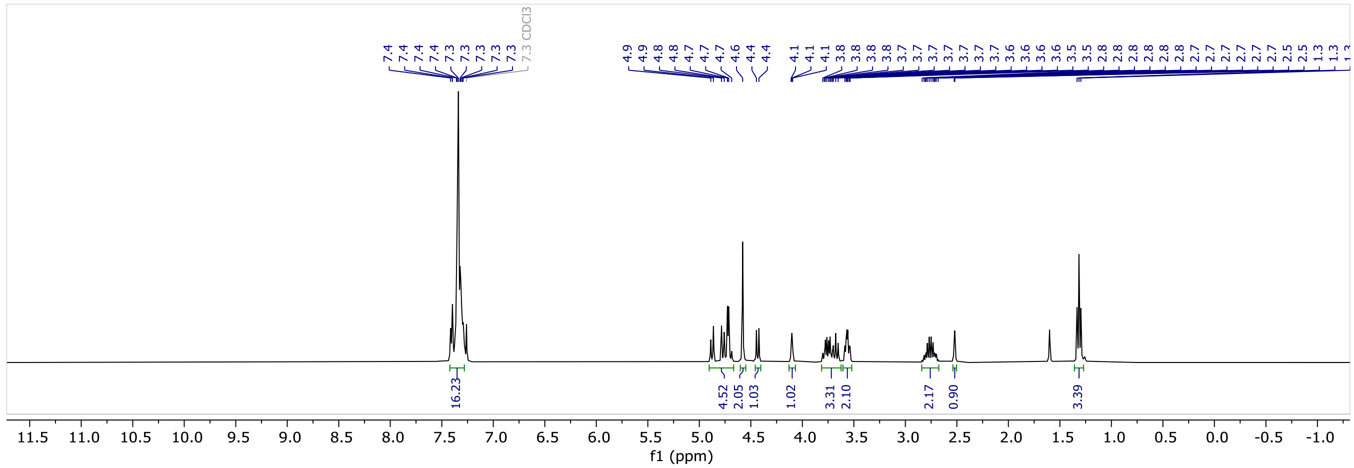

^{13}C NMR (101 MHz, $CDCl_3$) of **S5**:

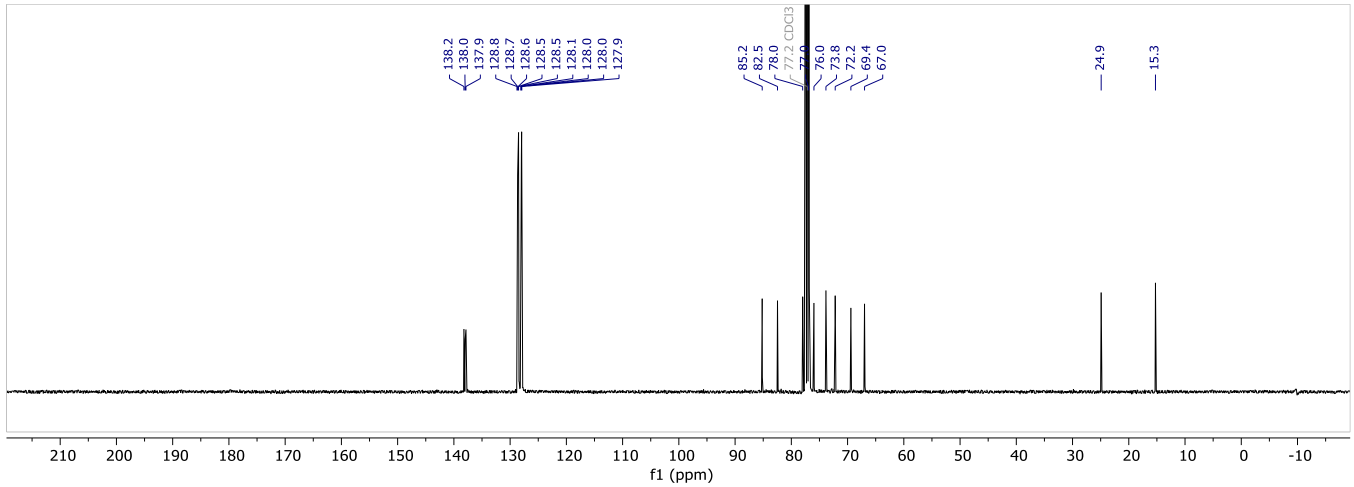

Ethyl 2,3-bis-*O*-benzyl-4,6-bis(trifluoroacetate)-1-thio-β-D-galactopyranoside (4,6TFA)

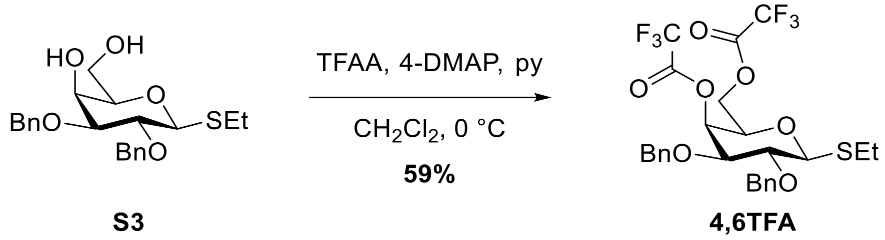

A stirred solution of **S3** (150 mg, 0.37 mmol, 1.0 equiv.) in anhydrous CH_2Cl_2 (3 mL) was cooled to 0 °C and anhydrous pyridine (60 μL, 0.74 mmol, 2.0 equiv.), trifluoroacetic anhydride (105 μL, 0.74 mmol, 2.0 equiv.) and 4-DMAP (5 mg, 0.04 mmol, 0.10 equiv.) were added. The solution was stirred at 0 °C for 30 minutes before it was quenched with water (10 mL). The mixture was extracted with CH_2Cl_2 (3 x 10 mL) and the combined organic phases were washed with 1 N HCl (10 mL), dried over Na_2SO_4, filtered and concentrated. Product **4,6TFA** (130 mg, 0.22 mmol, 59%) was obtained as a colorless oil after purification by column chromatography (SiO_2, Hex/EtOAc = 9:1 to 7:3).

R*f* = 0.90 (Hex/EtOAc 3:1).

^{1}H NMR (400 MHz, $CDCl_3$) δ 7.37 – 7.28 (m, 10H), 5.54 (d, *J* = 3.2 Hz, 1H), 4.88 – 4.80 (m, 1H), 4.77 – 4.70 (m, 2H), 4.63 – 4.56 (m, 1H), 4.54 – 4.43 (m, 2H), 4.35 (dd, *J* = 11.4, 5.6 Hz, 1H), 3.95 (t, *J* = 6.4 Hz, 1H), 3.75 – 3.67 (m, 1H), 3.59 (t, *J* = 9.4 Hz, 1H), 2.73 (qq, *J* = 13.7, 7.4 Hz, 2H), 1.31 (t, *J* = 7.5 Hz, 3H) ppm.

^{13}C NMR (101 MHz, $CDCl_3$) δ 137.69, 136.97, 128.66, 128.58, 128.54, 128.28, 128.16, 85.76, 79.86, 77.07, 76.18, 73.00, 72.98, 71.51, 64.93, 25.12, 15.14 ppm.

^{19}F NMR (376 MHz, $CDCl_3$) δ -74.60 (s), -74.86 (s) ppm.

HRMS (QToF): Calcd for $C_{26}H_{26}F_6O_7SNa$ $[M + Na]^+$ 619.1196; found 619.1201.

^{1}H NMR (400 MHz, $CDCl_3$) of **4,6TFA**:

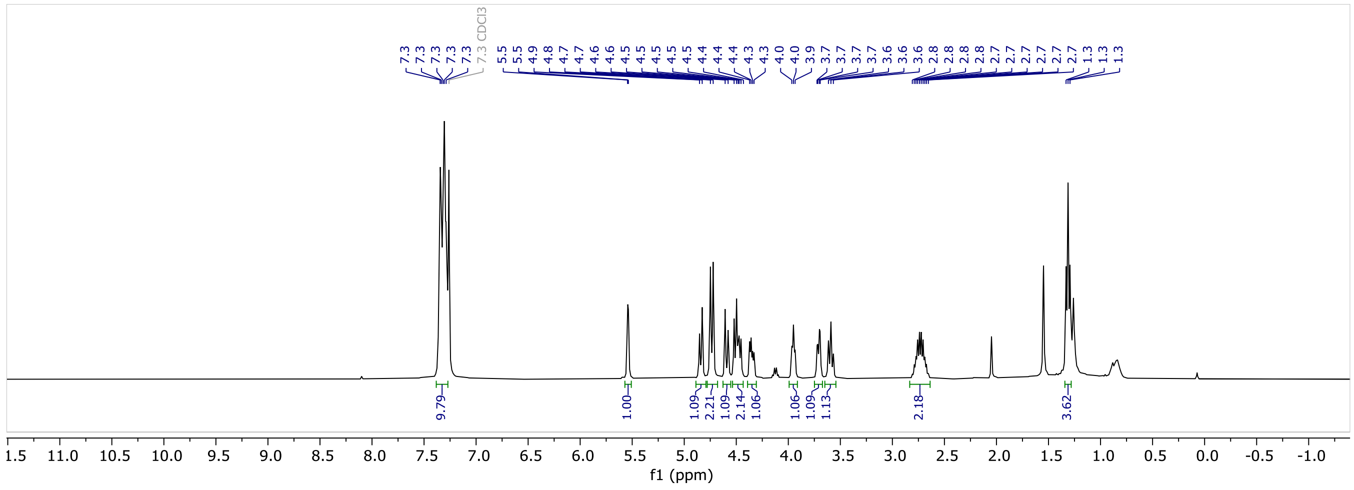

^{13}C NMR (101 MHz, $CDCl_3$) of **4,6TFA**:

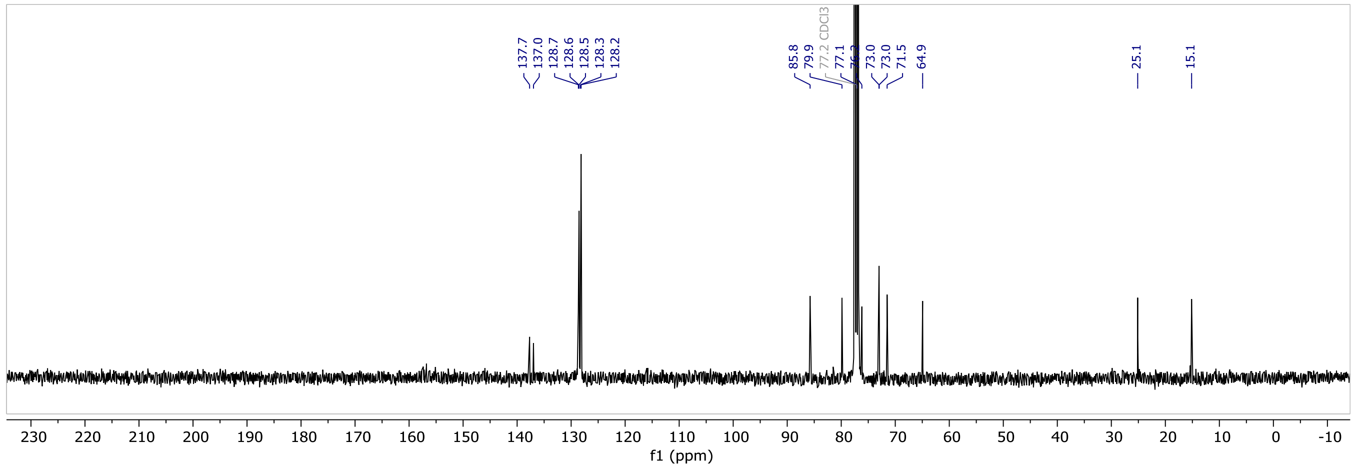

^{19}F NMR (376 MHz, $CDCl_3$) of **4,6TFA**:

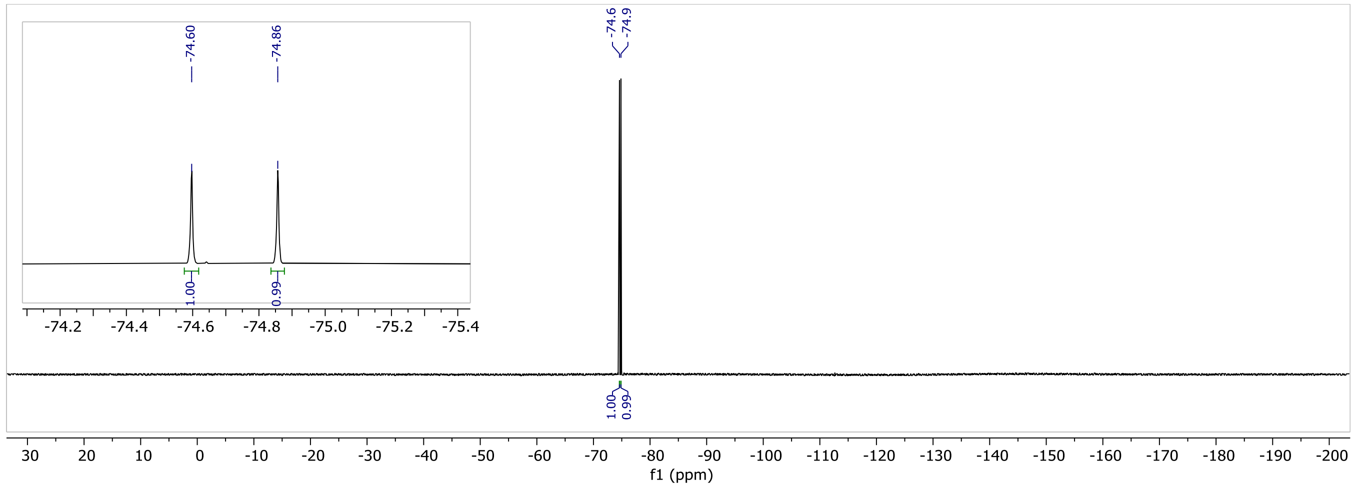

^{13}C,^{1}H HSQC of **4,6TFA**:

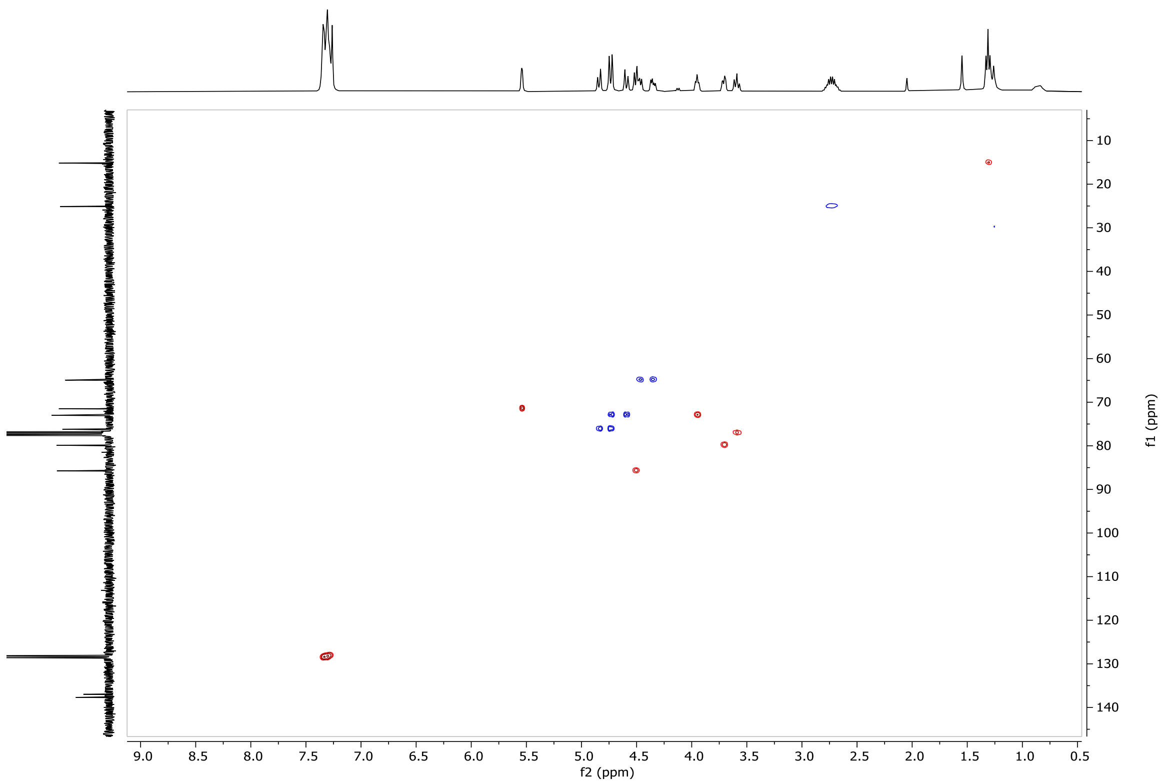

^{1}H,^{1}H COSY of **4,6TFA**:

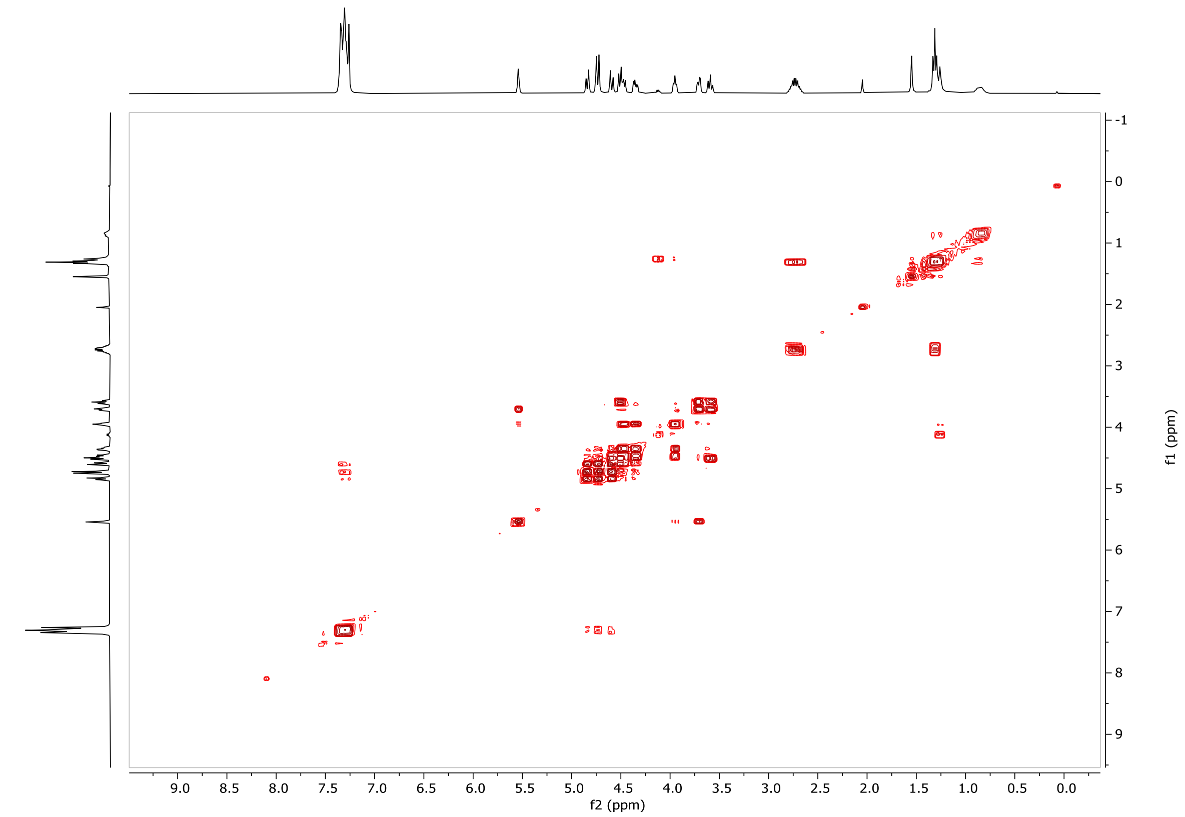

Ethyl 2,3,4-tris-*O*-benzyl-6-(trifluoroacetate)-1-thio-β-D-galactopyranoside (6TFA)

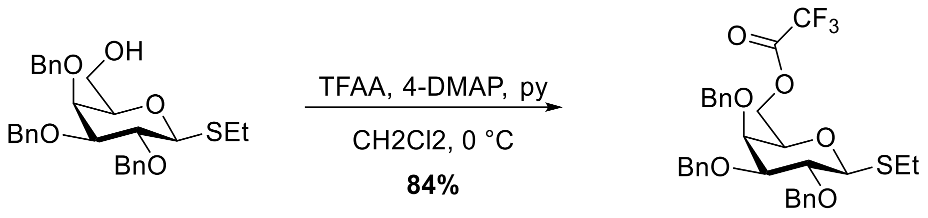

S4 **6TFA**

A stirred solution of **S4** (100 mg, 0.20 mmol, 1.0 equiv.) in anhydrous CH_2Cl_2 (3 mL) was cooled to 0 °C and anhydrous pyridine (20 μL, 0.24 mmol, 1.2 equiv.), trifluoroacetic anhydride (34 μL, 0.24 mmol, 1.2 equiv.) and 4-DMAP (2 mg, 0.02 mmol, 0.1 equiv.) were added. The solution was stirred at 0 °C for 30 minutes before it was quenched with water (10 mL). The mixture was extracted with CH_2Cl_2 (3 x 10 mL) and the combined organic phases were washed with 1 N HCl (10 mL), dried over Na_2SO_4, filtered and concentrated. Product **6TFA** (100 mg, 0.17 mmol, 84%) was obtained as a colorless solid after purification by column chromatography (SiO_2, Hex/EtOAc = 9:1 to 7:3).

$\mathbf{R}_f$ = 0.79 (Hex/EtOAc 3:1).

^{1}H NMR (400 MHz, $CDCl_3$) δ 7.43 – 7.26 (m, 15H), 5.03 – 4.58 (m, 6H), 4.45 (d, *J* = 9.6 Hz, 1H), 4.55 – 4.06 (m, 2H), 3.84 (t, *J* = 9.4 Hz, 1H), 3.81 – 3.77 (m, 1H), 3.66 – 3.55 (m, 2H), 2.82 – 2.62 (m, 2H), 1.29 (t, *J* = 7.4 Hz, 3H) ppm.

^{13}C NMR (101 MHz, $CDCl_3$) δ 138.19, 128.70, 128.53, 128.15, 128.04, 127.81, 85.61, 83.87, 78.42, 76.02, 75.22, 74.41, 73.59, 73.11, 66.92, 25.20, 15.18 ppm.

^{19}F NMR (376 MHz, $CDCl_3$) δ -74.94 (s) ppm.

HRMS (QToF): Calcd for $C_{31}H_{33}F_3O_6SNa$ $[M + Na]^+$ 613.1842; found 613.1844.

^{1}H NMR (400 MHz, $CDCl_3$) of **6TFA**:

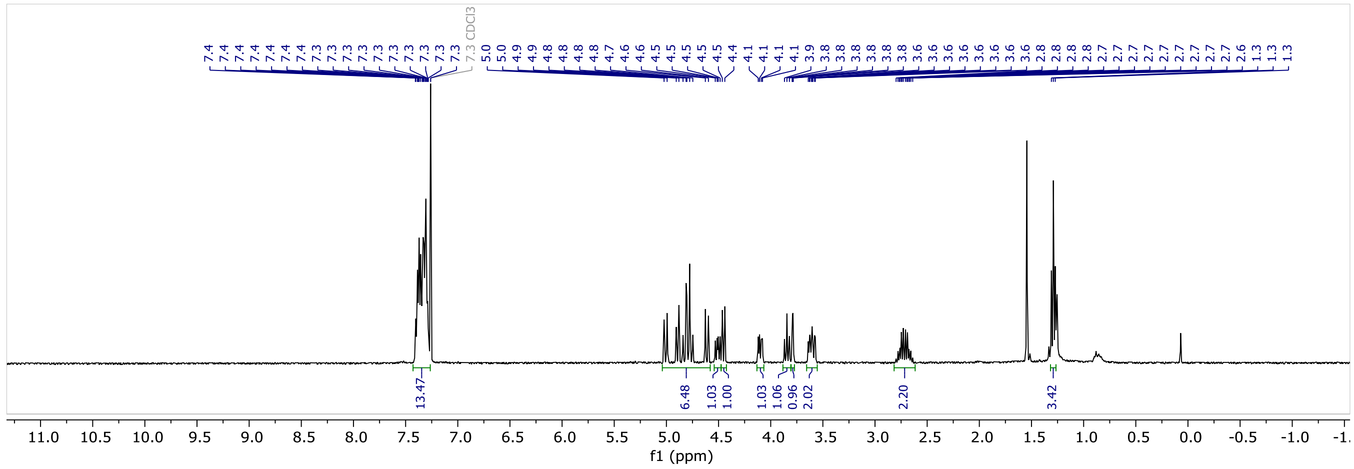

^{13}C NMR (101 MHz, $CDCl_3$) of **6TFA**:

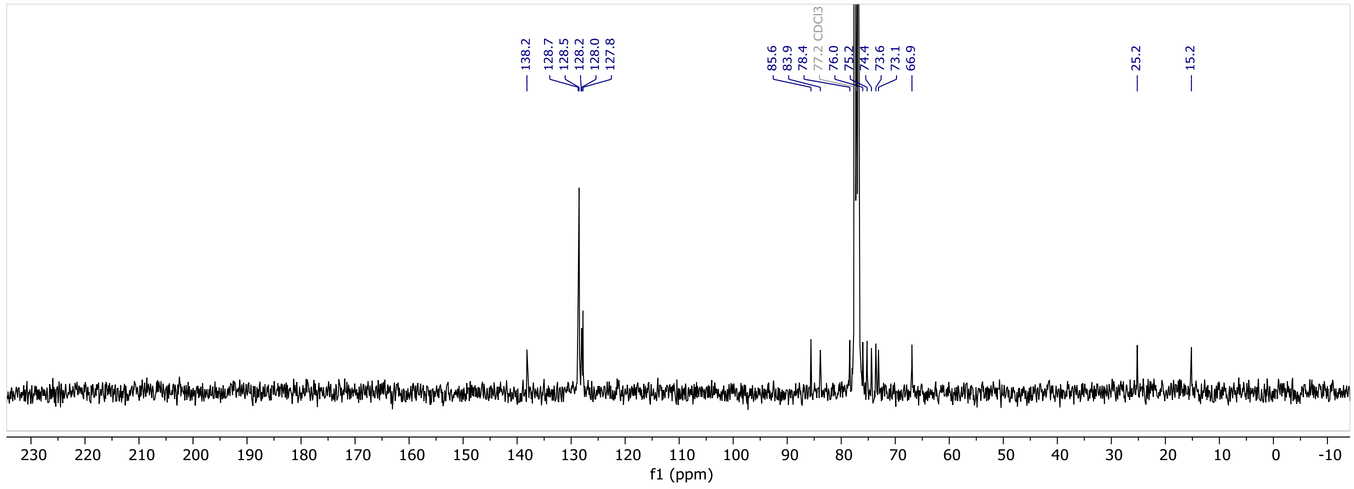

^{19}F NMR (376 MHz, $CDCl_3$) of **6TFA**:

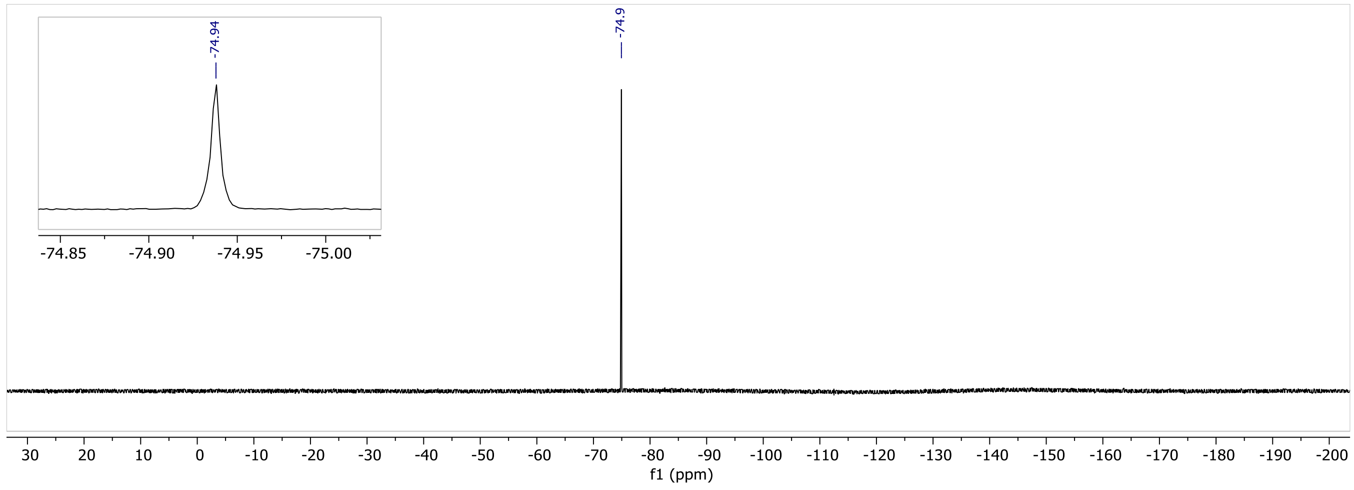

$^{13}C,^{1}H$ HSQC of **6TFA**:

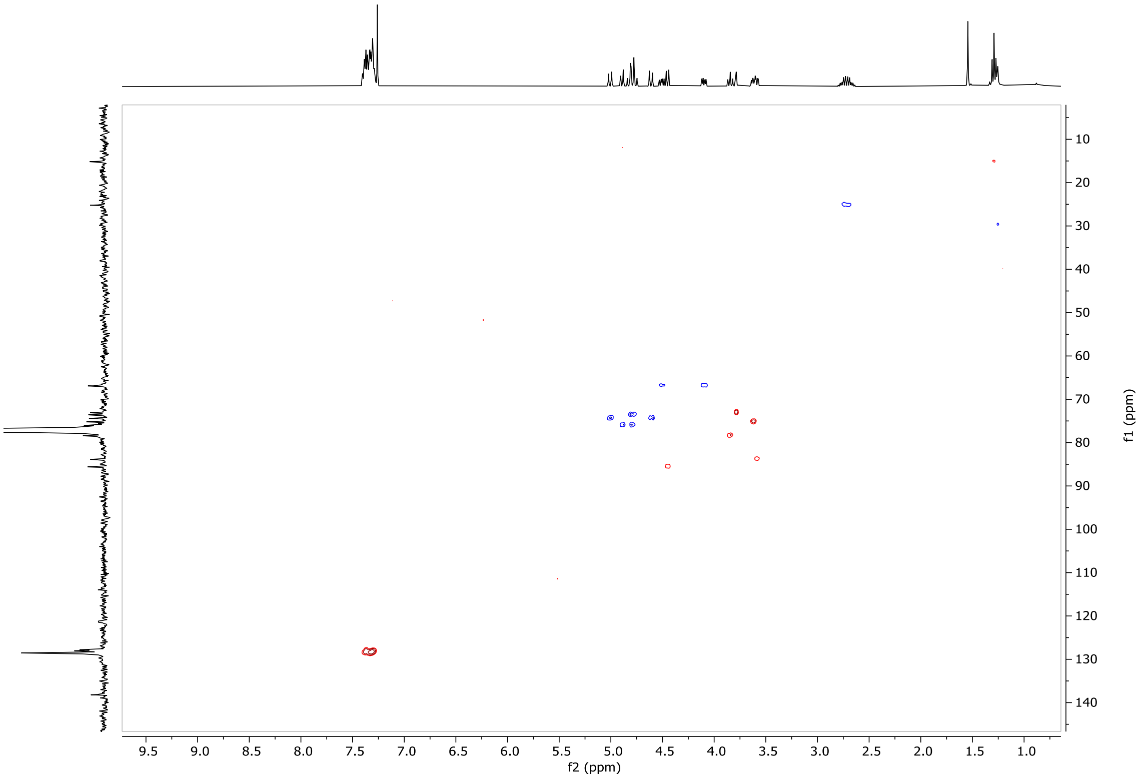

$^{1}H,^{1}H$ COSY of **6TFA**:

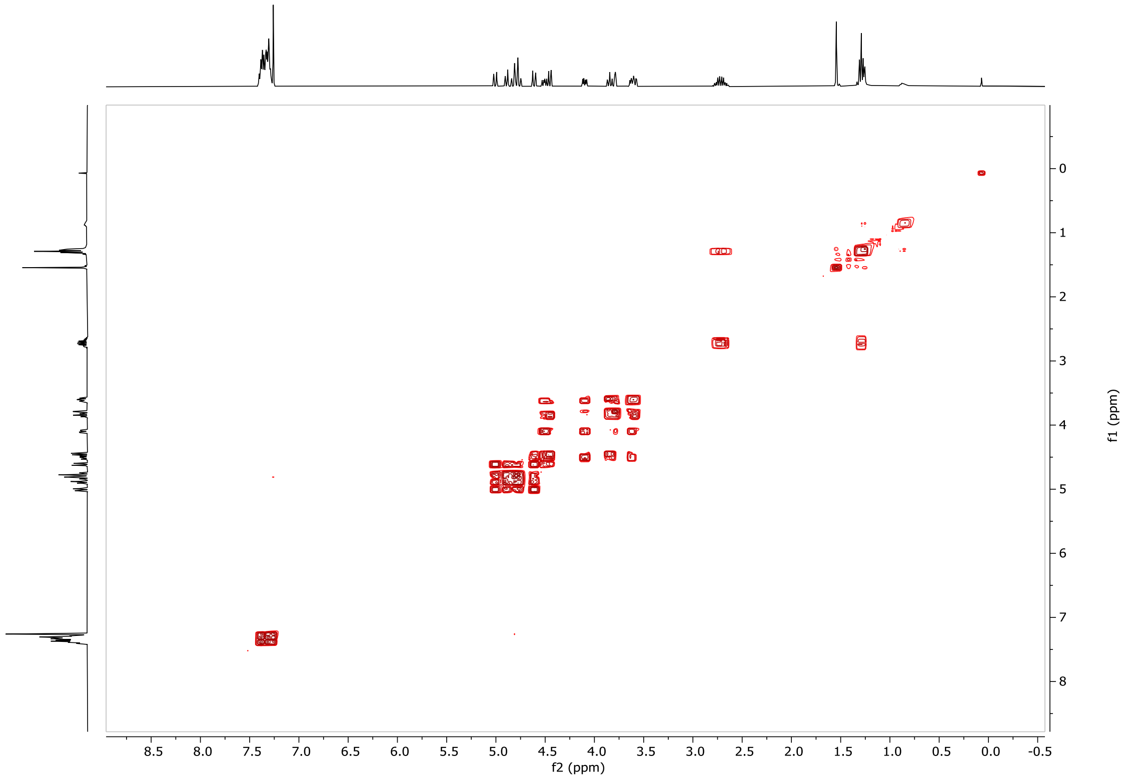

Ethyl 2,3,6-tris-*O*-benzyl-4-(trifluoroacetate)-1-thio-β-D-galactopyranoside (4TFA)

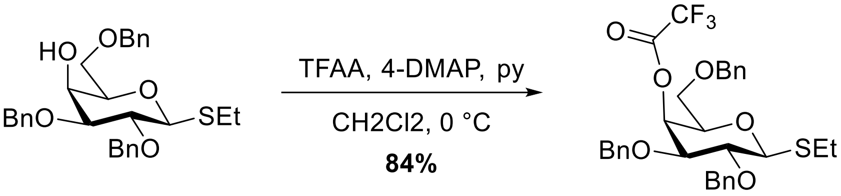

S5 **4TFA**

A stirred solution of **S5** (130 mg, 0.30 mmol, 1.0 equiv.) in anhydrous CH_2Cl_2 (3 mL) was cooled to 0 °C and anhydrous pyridine (29 μL, 0.36 mmol, 1.2 equiv.), trifluoroacetic anhydride (51 μL, 0.36 mmol, 1.2 equiv.) and 4-DMAP (4 mg, 0.03 mmol, 0.1 equiv.) were added. The solution was stirred at 0 °C for 30 min before it was quenched with water (10 mL). The mixture was extracted with CH_2Cl_2 (3 x 10 mL) and the combined organic phases were washed with 1 N HCl (10 mL), dried over Na_2SO_4, filtered and concentrated. Product **4TFA** (100 mg, 0.17 mmol, 84%) was obtained as a colorless oil after purification by column chromatography (SiO_2, Hex/EtOAc = 9:1 to 7:3).

$\mathbf{R}_f$ = 0.69 (Hex/EtOAc 3:1).

^{1}H NMR (400 MHz, $CDCl_3$) δ 7.36 – 7.27 (m, 15H), 5.74 (d, *J* = 3.1 Hz, 1H), 4.85 – 4.71 (m, 3H), 4.57 – 4.42 (m, 4H), 3.80 (dd, *J* = 8.3, 5.7 Hz, 1H), 3.71 – 3.60 (m, 2H), 3.55 (t, *J* = 9.4 Hz, 1H), 3.45 (t, *J* = 8.8 Hz, 1H), 2.82 – 2.62 (m, 2H), 1.30 (t, *J* = 7.5 Hz, 3H) ppm.

^{13}C NMR (101 MHz, $CDCl_3$) δ 137.94, 137.37, 137.26, 128.69, 128.56, 128.48, 128.24, 128.14, 128.05, 128.03, 85.46, 80.42, 77.32, 76.07, 74.73, 73.98, 72.47, 71.52, 67.14, 24.83, 15.15 ppm.

^{19}F NMR (376 MHz, $CDCl_3$) δ -74.64 (s) ppm.

HRMS (QToF): Calcd for $C_{31}H_{33}F_3O_6SNa$ $[M + Na]^+$ 613.1842; found 613.1852.

^{1}H NMR (400 MHz, $CDCl_3$) of **4TFA**:

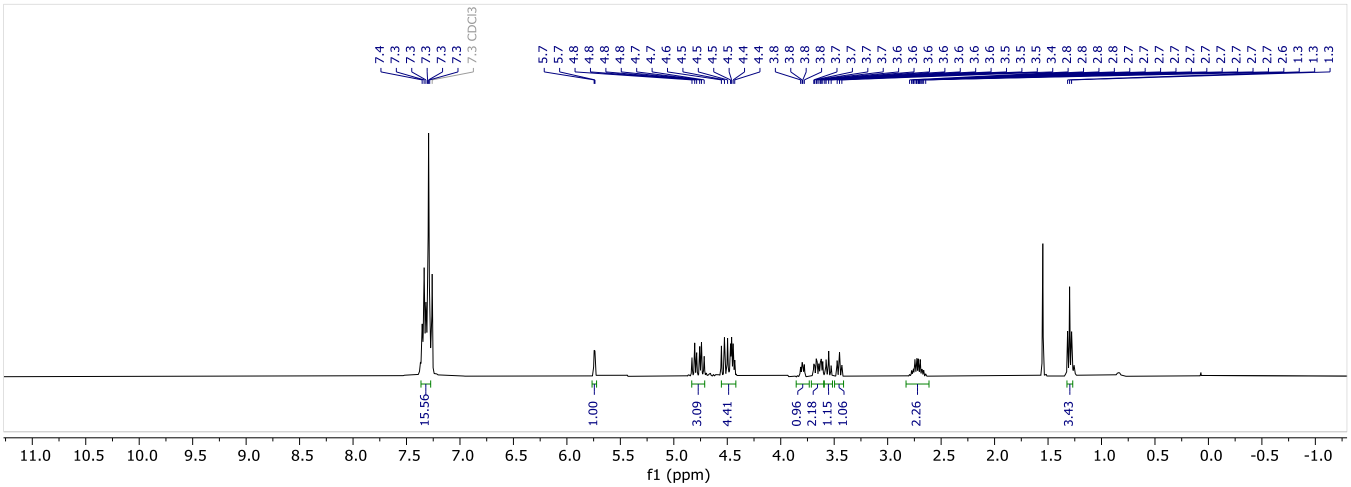

^{13}C NMR (101 MHz, $CDCl_3$) of **4TFA**:

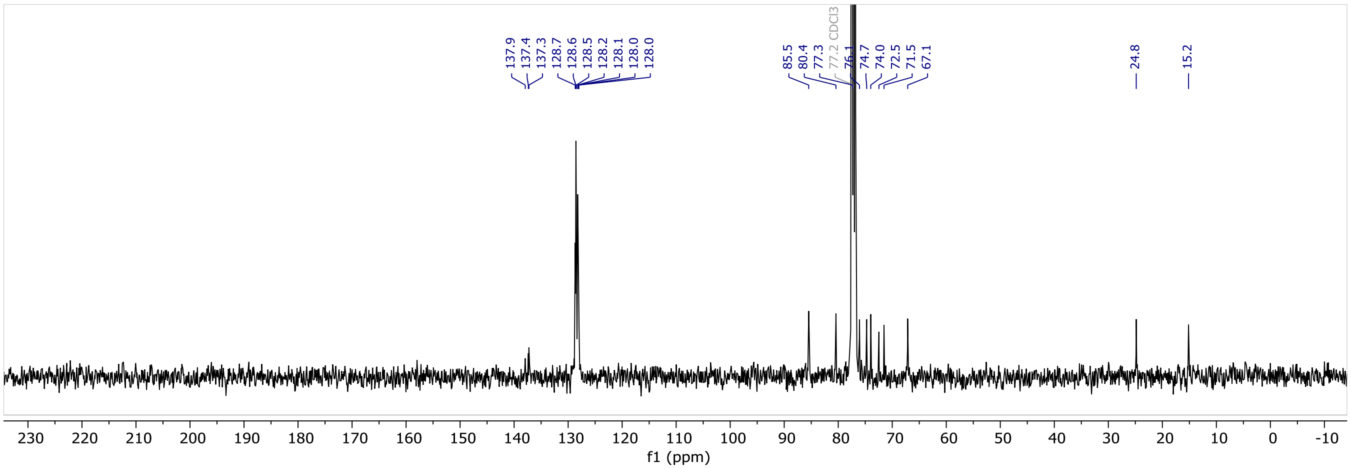

^{19}F NMR (376 MHz, $CDCl_3$) of **4TFA**:

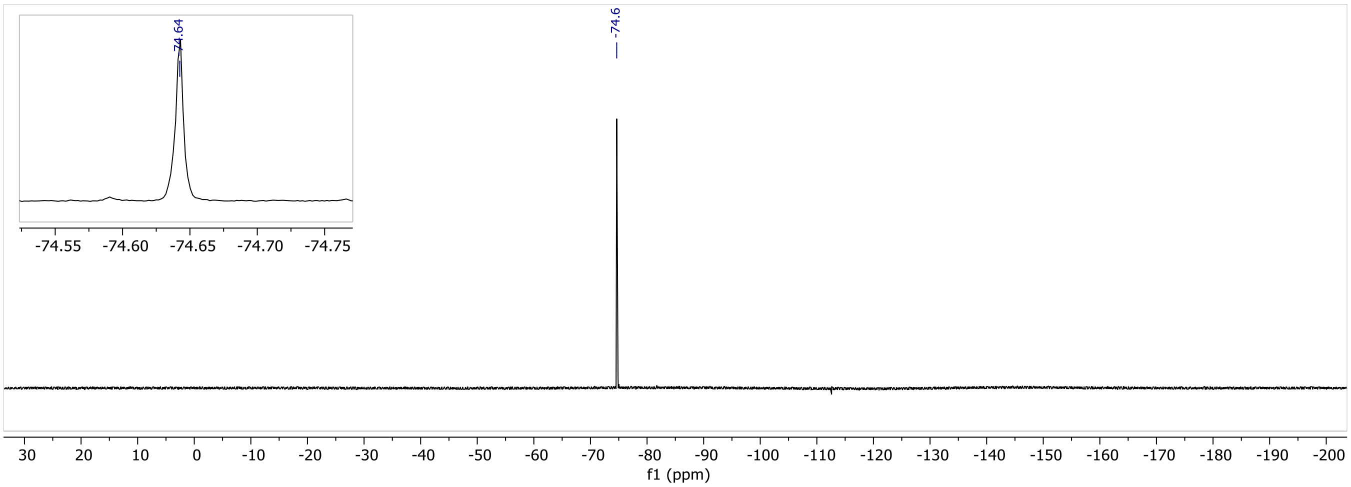

$^{13}C,^{1}H$ HSQC of **4TFA**:

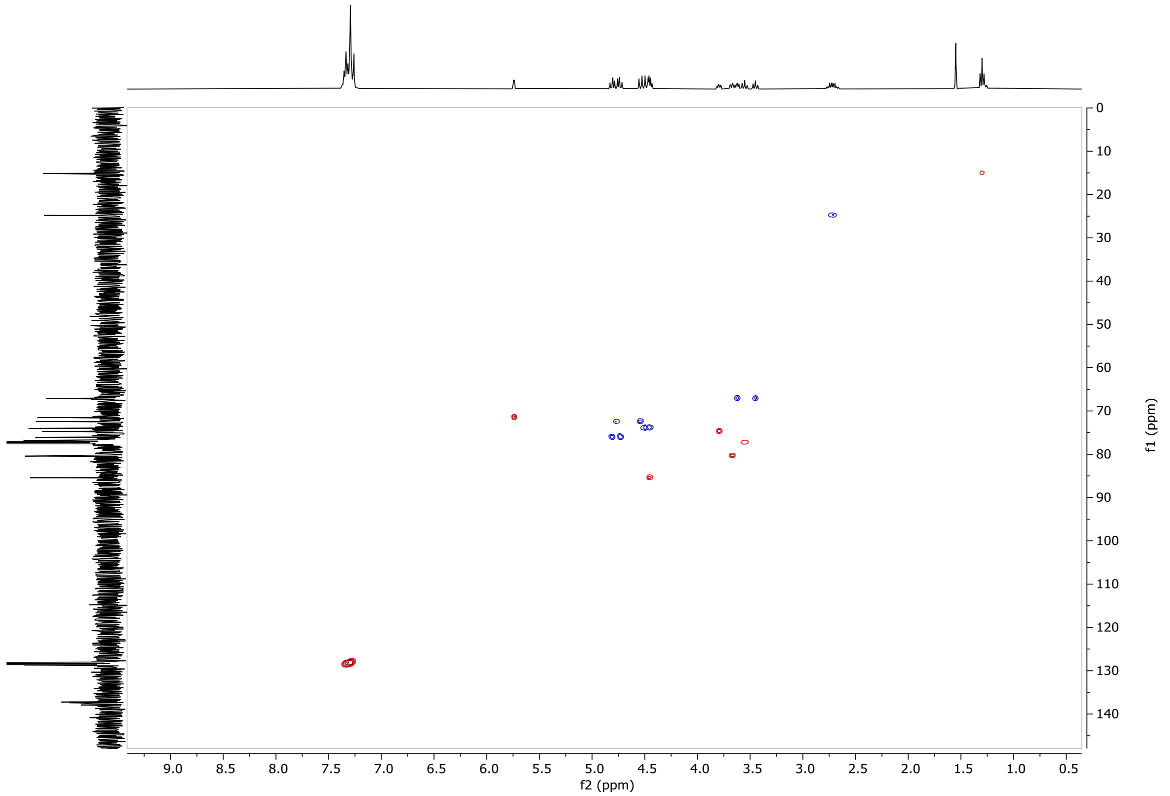

$^{1}H,^{1}H$ COSY of **4TFA**:

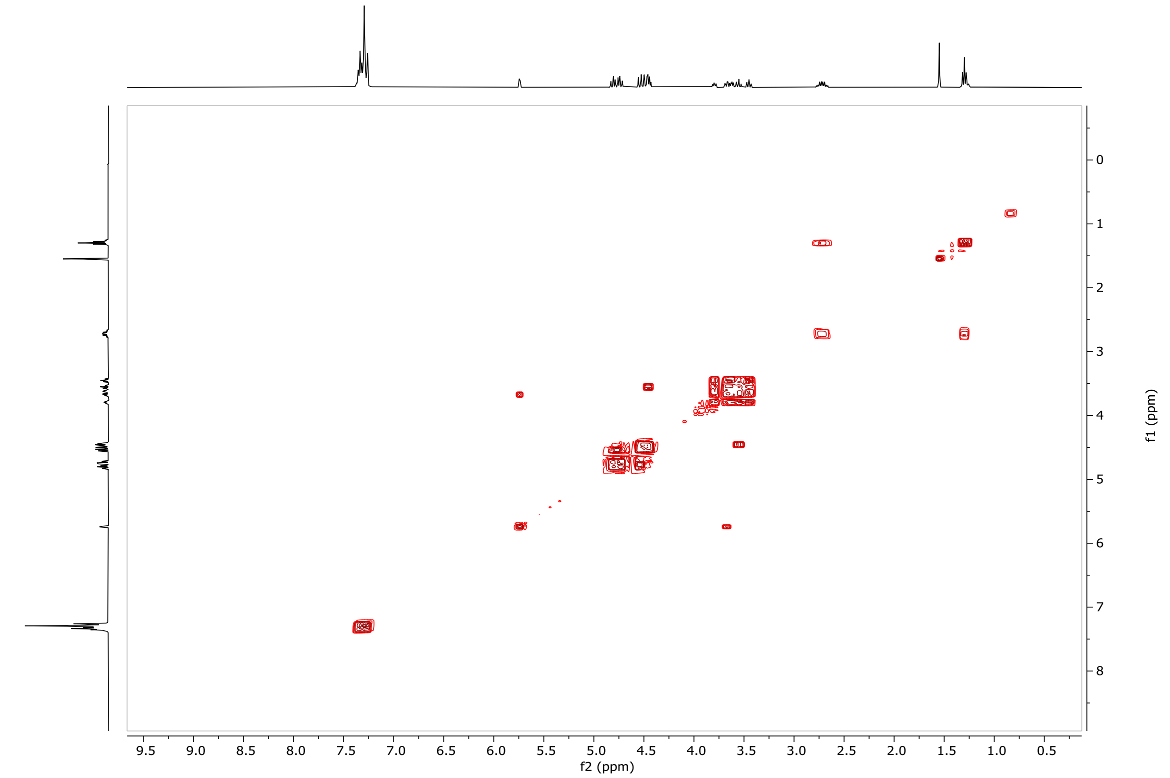

Ethyl 2,3-bis-*O*-benzyl-4,6-bis(2,2-dimethylpropanoate)-1-thio-β-D-galactopyranoside (4,6Piv)

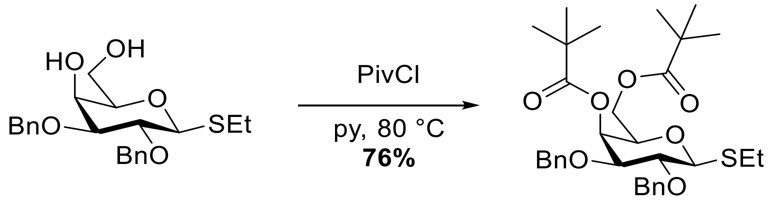

To a solution of **S3** (140 mg, 0.35 mmol, 1.0 equiv.) in anhydrous pyridine (3 mL) pivaloyl chloride (1.5 mL, 12.13 mmol, 35.0 equiv.) was added. The mixture was stirred at 80 °C for 2 hours. The volatiles were evaporated. Product **4,6Piv** (150 mg, 0.26 mmol, 76%) was obtained as a colorless oil after purification by column chromatography (SiO_2, Hex/EtOAc = 9:1).

$\mathbf{R}_f$ = 0.47 (Hex/EtOAc 9:1).

^{1}H NMR (400 MHz, $CDCl_3$) δ 7.39 – 7.27 (m, 10H), 5.53 (d, *J* = 3.2 Hz, 1H), 4.84 – 4.70 (m, 3H), 4.53 – 4.44 (m, 2H), 4.11 (qd, *J* = 11.3, 6.8 Hz, 2H), 3.82 (t, *J* = 6.7 Hz, 1H), 3.62 (dd, *J* = 9.3, 3.2 Hz, 1H), 3.54 (t, *J* = 9.4 Hz, 1H), 2.84 – 2.63 (m, 2H), 1.31 (t, *J* = 7.4 Hz, 3H), 1.22 (s, 9H), 1.20 (s, 9H) ppm.

^{13}C NMR (101 MHz, $CDCl_3$) δ 178.17, 177.60, 138.07, 137.89, 128.64, 128.43, 128.39, 128.20, 127.95, 127.81, 85.19, 81.10, 77.43, 75.90, 74.63, 71.96, 66.31, 62.24, 39.23, 38.87, 27.33, 27.23, 24.78, 15.16 ppm.

HRMS (QToF): Calcd for $C_{32}H_{44}O_7SNa$ $[M + Na]^+$ 595.2700; found 595.2701.

^{1}H NMR (400 MHz, $CDCl_3$) of **4,6Piv**:

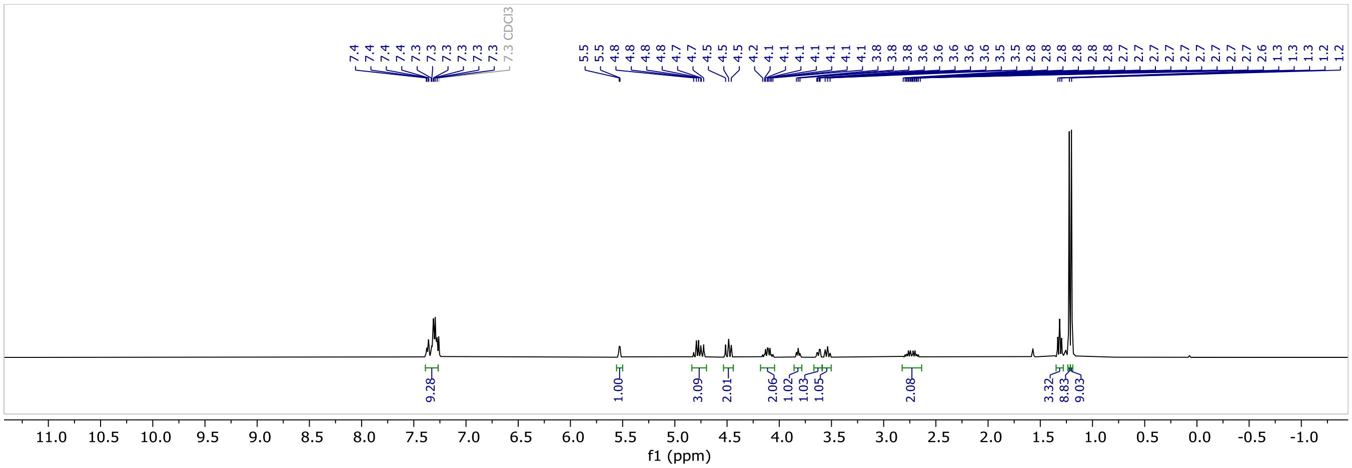

^{13}C NMR (101 MHz, $CDCl_3$) of **4,6Piv**:

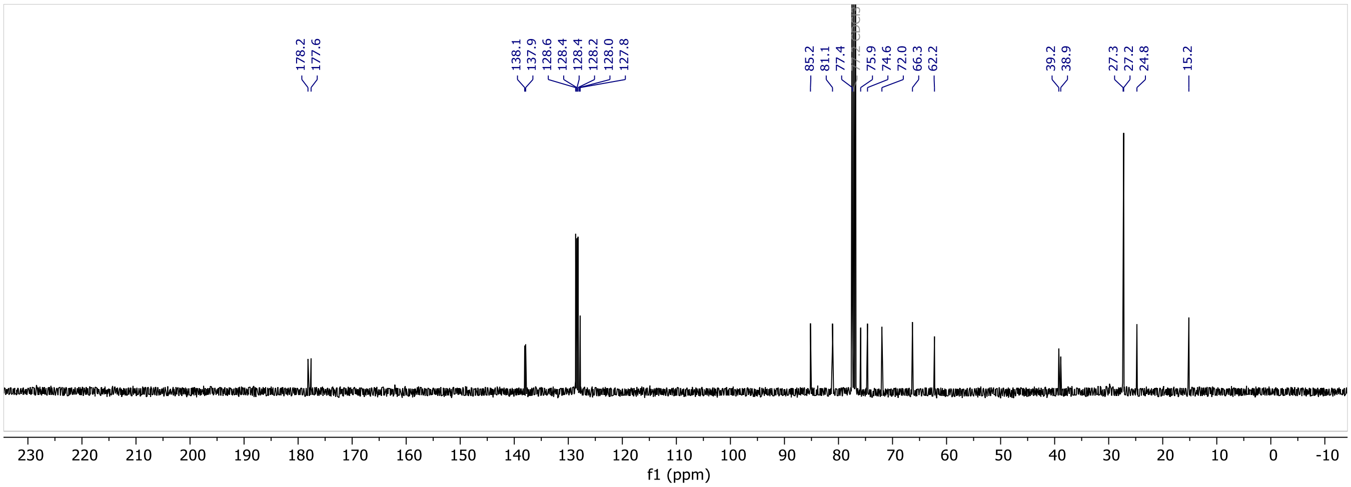

$^{13}C,^{1}H$ HSQC of **4,6Piv**:

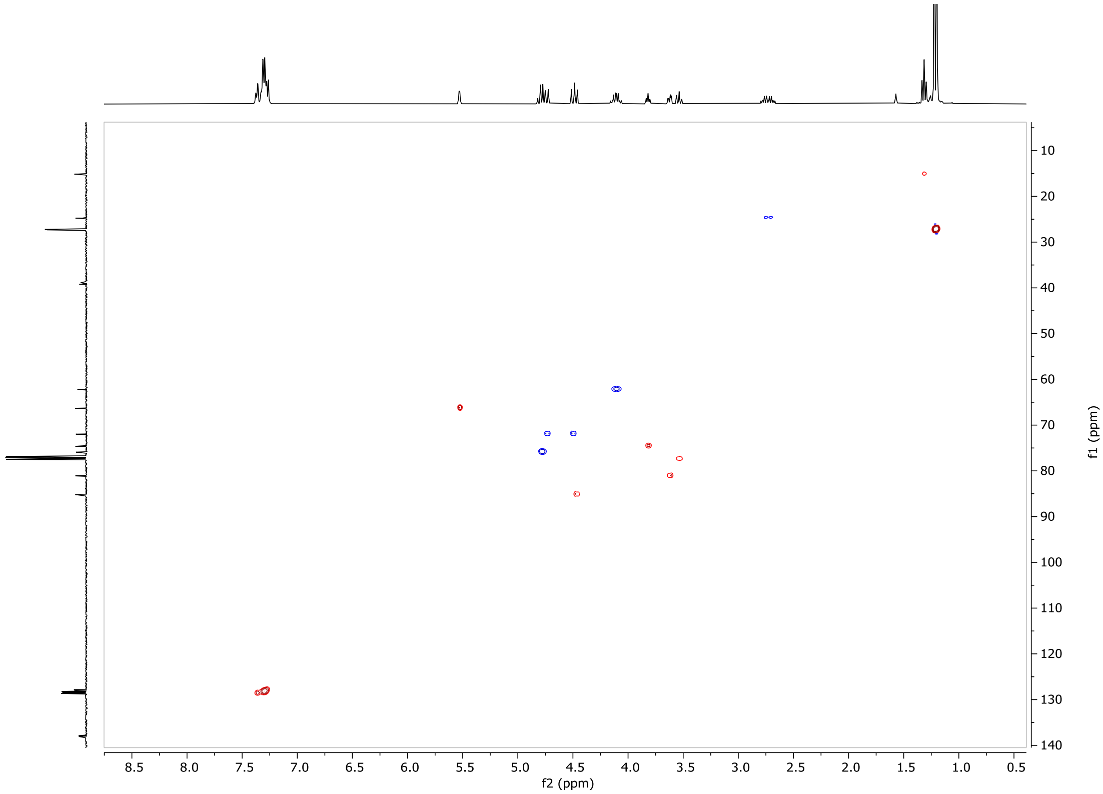

^{1}H,^{1}H COSY of **4,6Piv**:

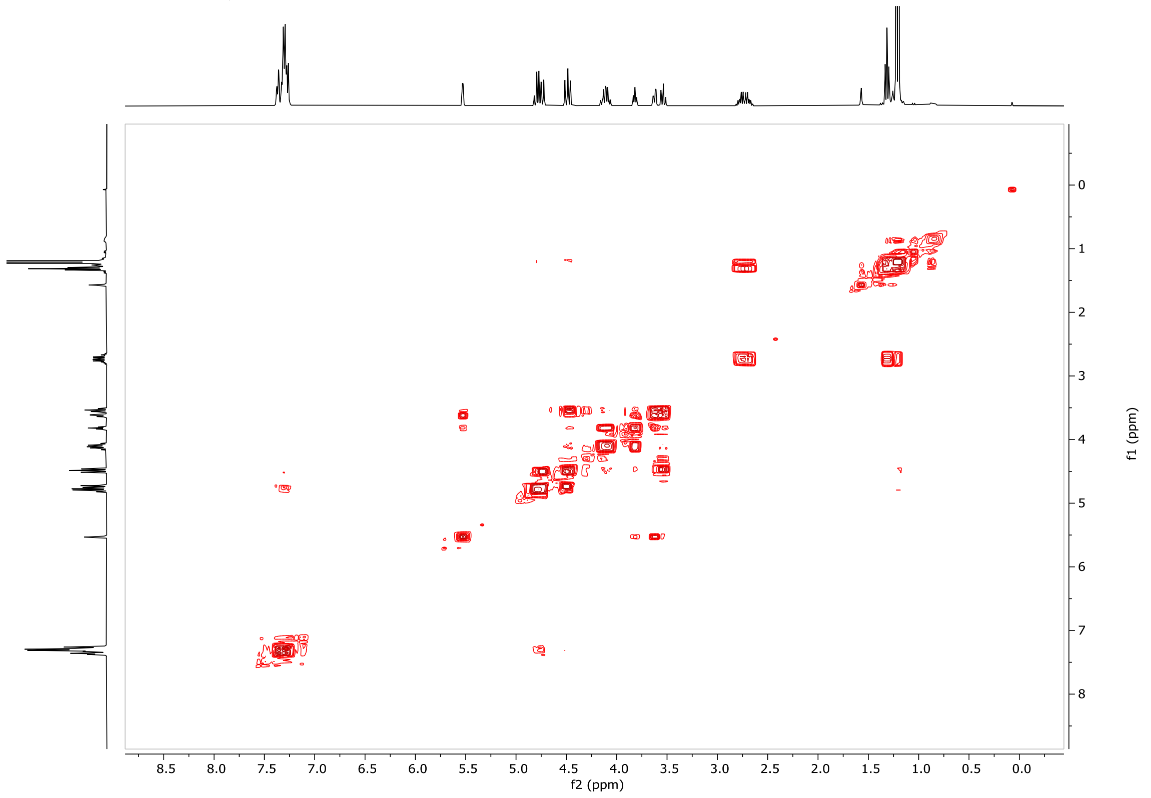

Ethyl 2,3,4-tris-*O*-benzyl-6-(2,2-dimethylpropanoate)-1-thio-β-D-galactopyranoside (6Piv)

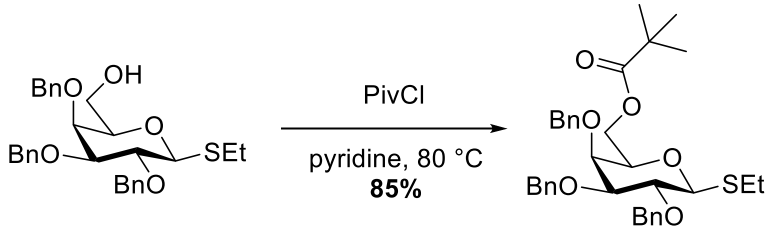

S4 **6Piv**

To a solution of **S4** (150 mg, 0.30 mmol, 1.0 equiv.) in anhydrous pyridine (3 mL) pivaloyl chloride (0.75 mL, 6.06 mmol, 20.0 equiv.) was added. The mixture was stirred at 80 °C for 2 hours. The volatiles were evaporated. Product **6Piv** (150 mg, 0.26 mmol, 85%) was obtained as a colorless oil after purification by column chromatography (SiO_2, Hex/EtOAc = 9:1).

R*f* = 0.53 (Hex/EtOAc 3:1).

^{1}H NMR (400 MHz, $CDCl_3$) δ 7.45 – 7.27 (m, 15H), 5.04 – 4.71 (m, 5H), 4.63 (d, *J* = 11.6 Hz, 1H), 4.44 (d, *J* = 9.7 Hz, 1H), 4.26 (dd, *J* = 11.2, 7.1 Hz, 1H), 4.06 (dd, *J* = 11.2, 5.6 Hz, 1H), 3.83 (t, *J* = 9.5 Hz, 1H), 3.79 – 3.76 (m, 1H), 3.60 – 3.50 (m, 2H), 2.84 – 2.63 (m, 2H), 1.30 (t, *J* = 7.4 Hz, 3H), 1.15 (s, 9H) ppm.

^{13}C NMR (151 MHz, $CDCl_3$) δ 178.27, 138.50, 138.41, 138.38, 128.62, 128.57, 128.47, 128.44, 128.20, 127.92, 127.91, 127.82, 127.79, 85.39, 84.12, 78.64, 76.18, 75.95, 74.60, 74.14, 73.33, 63.42, 38.83, 27.27, 25.01, 15.23 ppm.

HRMS (QToF): Calcd for $C_{34}H_{42}O_6SNa$ $[M + Na]^+$ 601.2594; found 601.2610.

^{1}H NMR (400 MHz, $CDCl_3$) of **6Piv**:

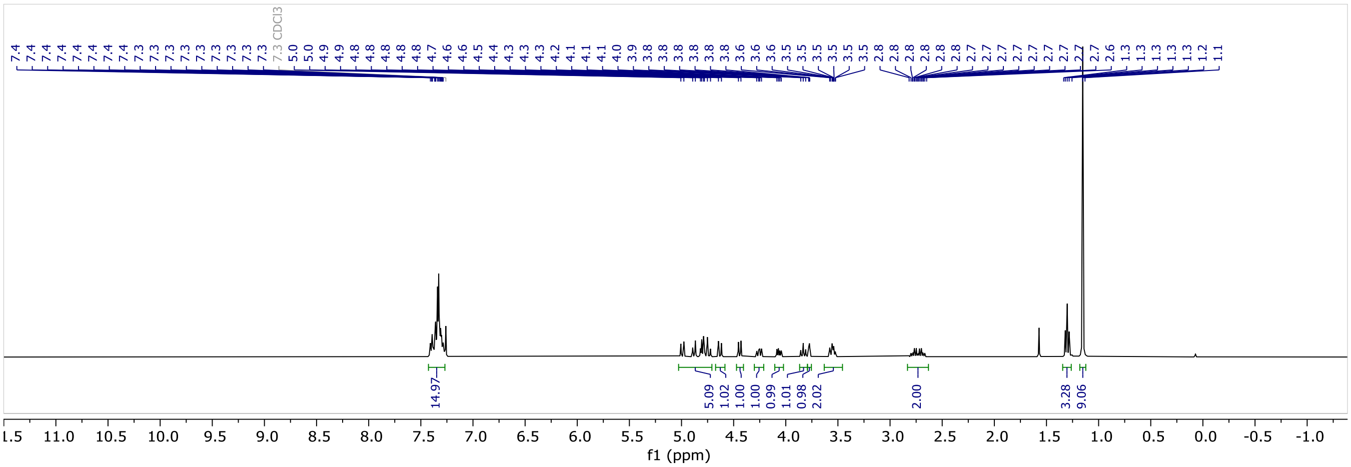

^{13}C NMR (101 MHz, $CDCl_3$) of **6Piv**:

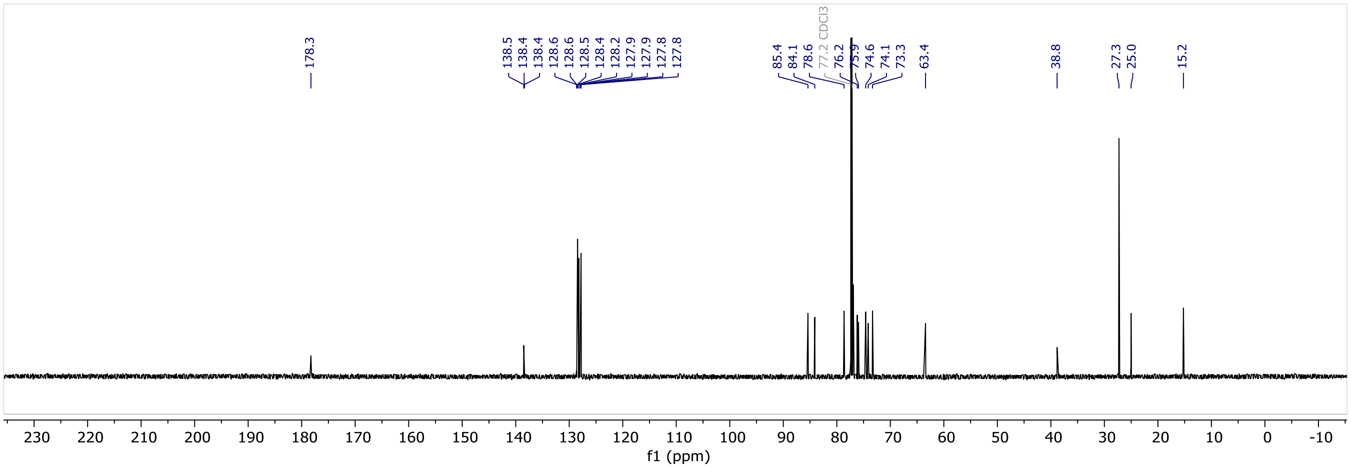

$^{13}C,^{1}H$ HSQC of **6Piv**:

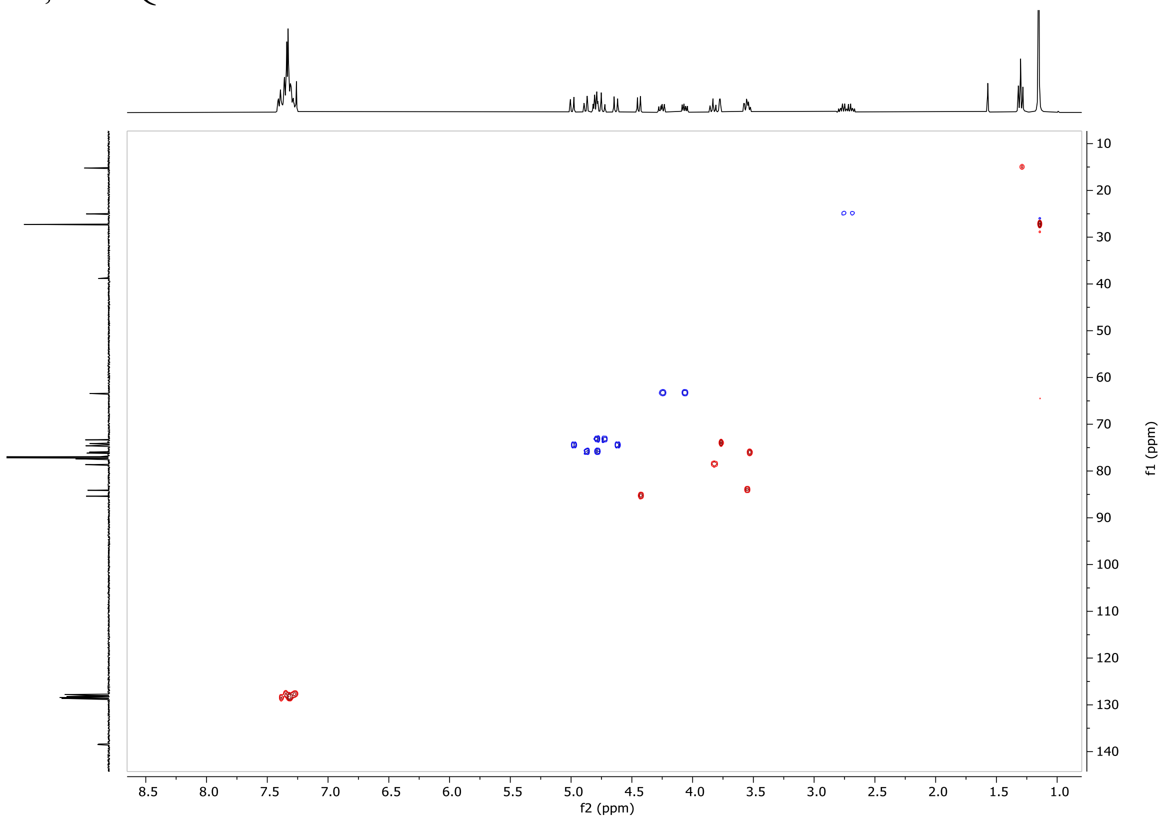

$^{1}H,^{1}H$ COSY of **6Piv**:

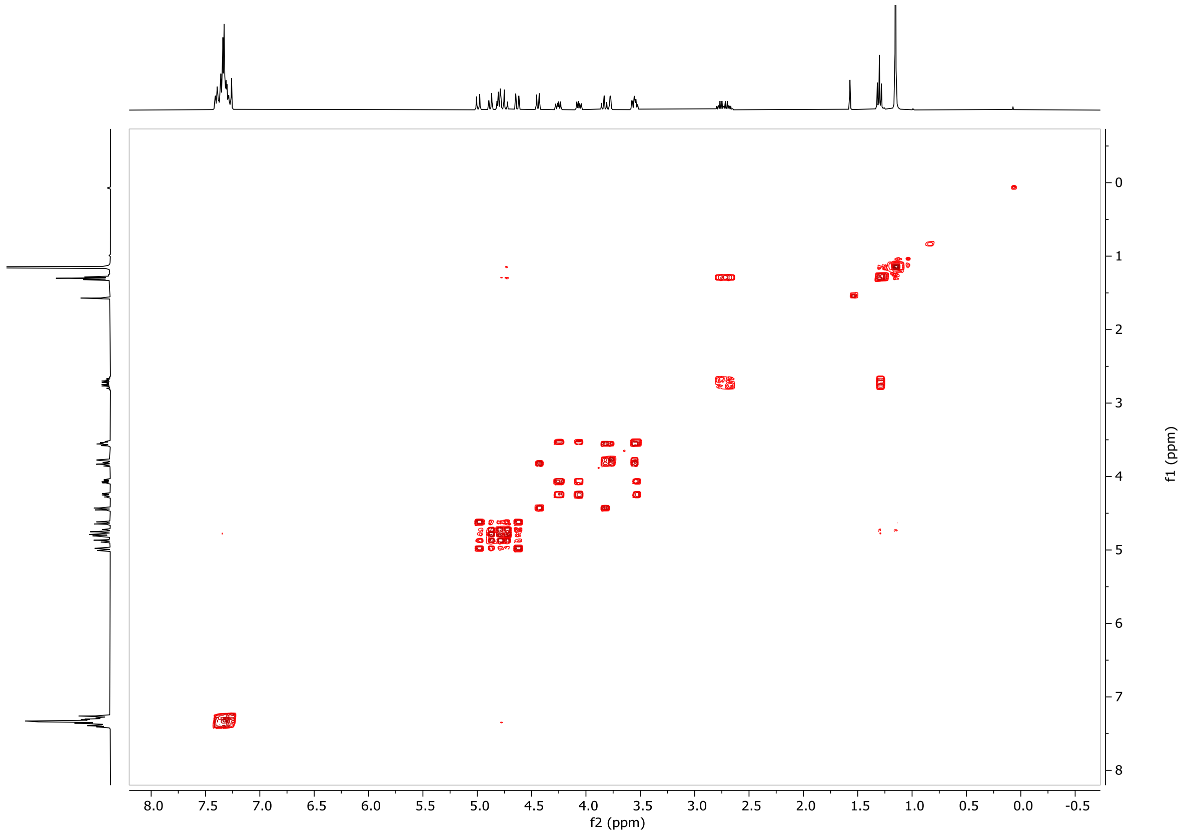

Ethyl 2,3,6-tris-*O*-benzyl-4-(2,2-dimethylpropanoate)-1-thio-β-D-galactopyranoside (4Piv)

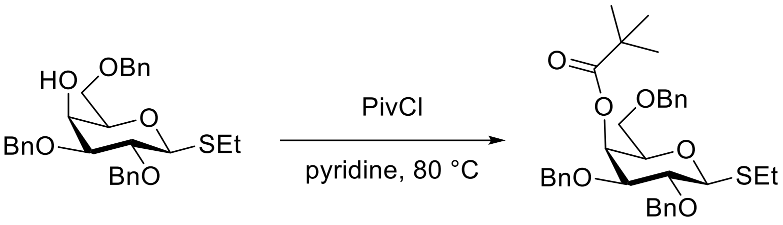

S5 **4Piv**

To a solution of **S5** (100 mg, 0.20 mmol, 1.0 equiv.) in anhydrous pyridine (1.5 mL) pivaloyl chloride (0.50 mL, 6.06 mmol, 20.0 equiv.) was added. The mixture was stirred at 80 °C for 2 hours. The volatiles were evaporated. Product **4Piv** (110 mg, 0.19 mmol, 94%) was obtained as a colorless oil after purification by column chromatography (SiO_2, Hex/EtOAc = 9:1).

R_f = 0.49 (Hex/EtOAc 3:1).

^{1}H NMR (600 MHz, $CDCl_3$) δ 7.37 – 7.26 (m, 14H), 5.64 (d, J = 2.3 Hz, 1H), 4.84 – 4.71 (m, 3H), 4.58 – 4.39 (m, 4H), 3.75 (d, J = 1.0 Hz, 1H), 3.64 – 3.50 (m, 3H), 3.46 (dd, J = 9.5, 6.9 Hz, 1H), 2.81 – 2.65 (m, 2H), 1.31 (t, J = 7.4 Hz, 3H), 1.19 (s, 9H) ppm.

^{13}C NMR (151 MHz, $CDCl_3$) δ 177.61, 138.20, 138.10, 137.82, 128.63, 128.61, 128.41, 128.36, 128.21, 128.14, 127.99, 127.90, 127.73, 85.24, 81.47, 77.52, 76.12, 75.87, 73.92, 71.80, 68.48, 66.61, 39.22, 27.38, 24.67, 15.20 ppm.

HRMS (QToF): Calcd for $C_{34}H_{42}O_6SNa$ $[M + Na]^+$ 601.2594; found 601.2614.

^{1}H NMR (400 MHz, $CDCl_3$) of **4Piv**:

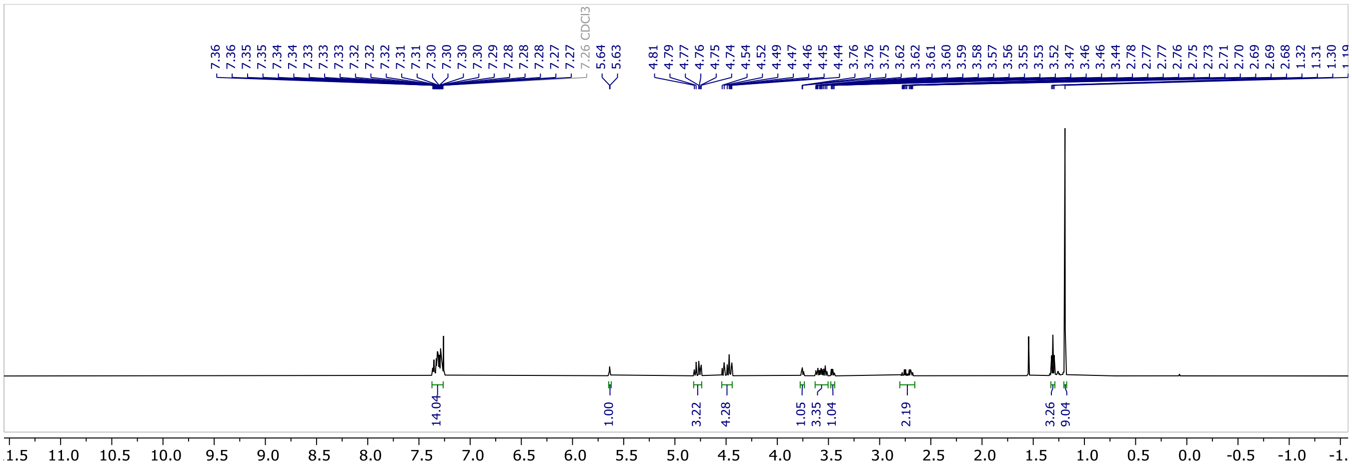

^{13}C NMR (101 MHz, $CDCl_3$) of **4Piv**:

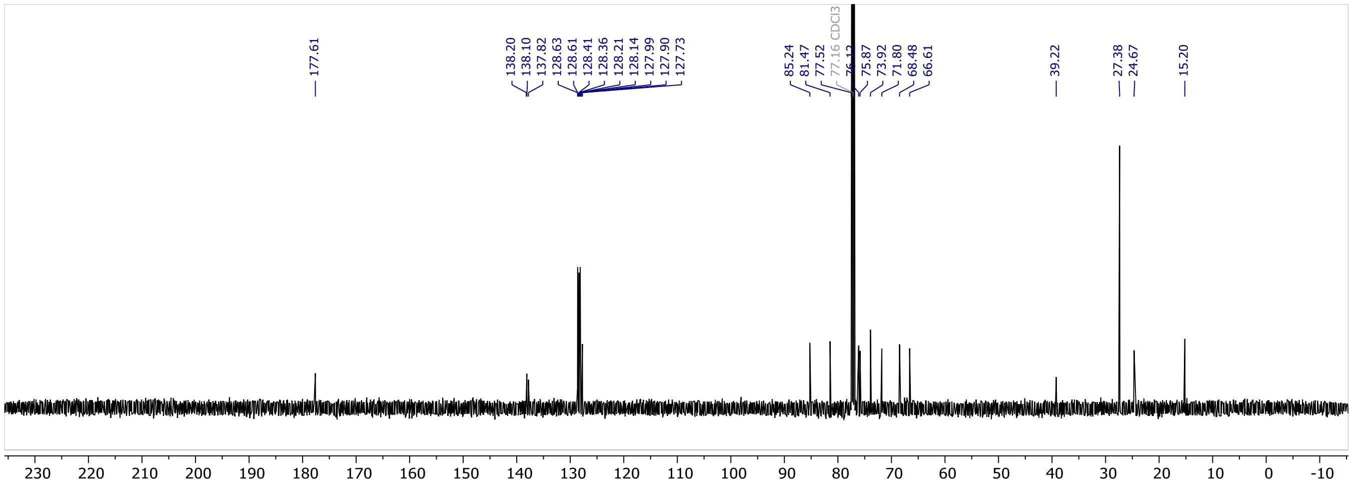

$^{13}C,^{1}H$ HSQC of **4Piv**:

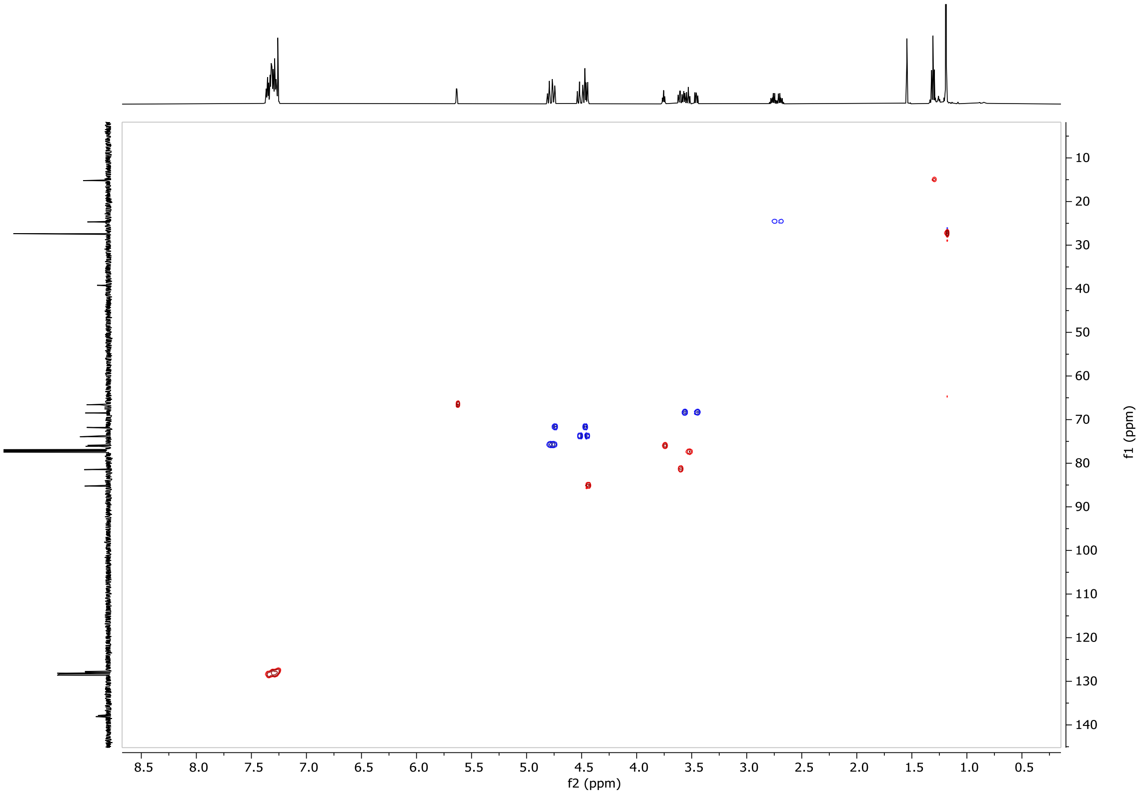

^{1}H,^{1}H COSY of **4Piv**:

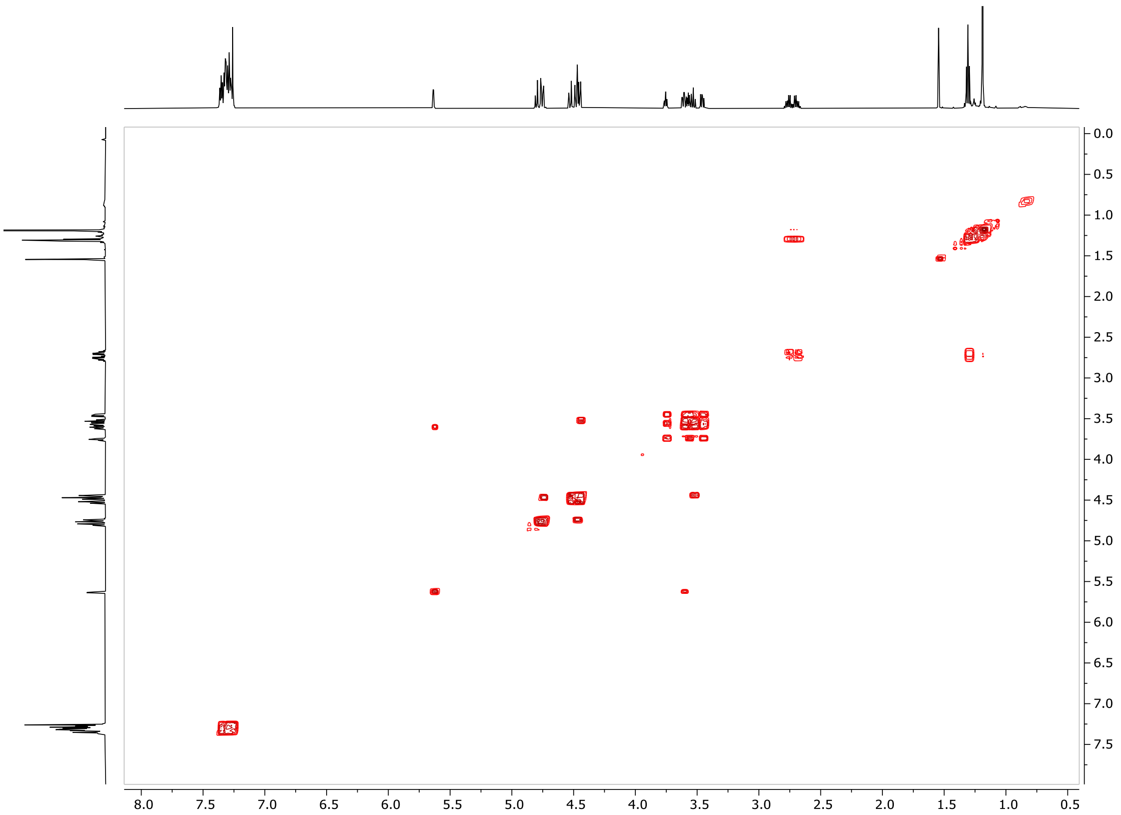

Ethyl 2-*O*-benzyl-3-*O*-(2-naphthalenylmethyl)-4,6-O-[(S)-phenylmethylene]-1-thio-β-D-galactopyranoside[21] (S7)

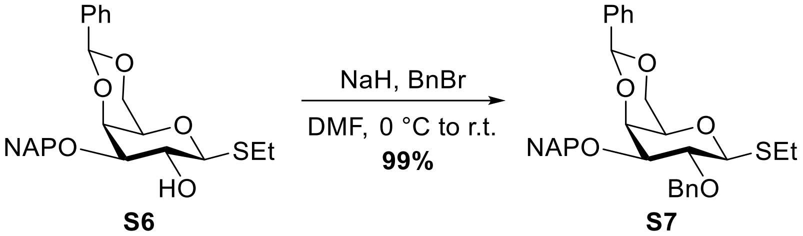

Compound **S6**[21] (7.4 g, 16.4 mmol, 1.0 equiv.) was dissolved in anhydrous DMF (75 mL). The stirred solution was cooled to 0 °C and sodium hydride (1.5 g, 24.6 mmol; 60% dispersion in mineral oil, 2.3 equiv.) was added in small portions. After 30 min, benzyl bromide (1.3 mL, 37.7 mmol, 1.5 equiv.) was added dropwise. The reaction mixture was allowed to warm up to room temperature and was stirred overnight. Methanol (10 mL) was added, the reaction mixture was stirred for 10 min and afterwards diluted with ethyl acetate (100 mL). The organic layer was washed with water (2 x 100 mL). The aqueous phase was extracted with ethyl acetate (2 x 100 mL). The combined organic phase was washed with water (100 mL), dried over Na_2SO_4 and concentrated.

Product **S7** (8.8 g, 16.2 mmol, **99%**) was obtained as a colorless solid after purification by column chromatography (SiO_2, Hex/EtOAc = 3:1).

$\mathbf{R_f}$ = 0.22 (Hex/EtOAc 3:1).

^{1}H NMR (700 MHz, $CDCl_3$) δ 7.87 – 7.78 (m, 3H), 7.74 – 7.69 (m, 1H), 7.57 – 7.29 (m, 13H), 5.48 (s, 1H), 4.96 – 4.87 (m, 4H), 4.44 (d, *J* = 9.7 Hz, 1H), 4.30 (dd, *J* = 12.3, 1.8 Hz, 1H), 4.17 (d, *J* = 3.7 Hz, 1H), 3.96 – 3.90 (m, 2H), 3.65 (dd, *J* = 9.2, 3.5 Hz, 1H), 3.35 – 3.32 (m, 1H), 2.90 – 2.73 (m, 2H), 1.34 (t, *J* = 7.5 Hz, 3H) ppm.

^{13}C NMR (176 MHz, $CDCl_3$) δ 138.6, 138.1, 135.9, 133.4, 133.2, 129.2, 128.5, 128.5, 128.4, 128.3, 128.0, 127.9, 127.8, 126.7, 126.7, 126.3, 126.1, 126.0, 101.7, 84.6, 81.1, 77.1, 75.9, 74.2, 72.1, 69.9, 69.5, 24.0, 15.2 ppm.

HRMS (QToF): Calcd for $C_{33}H_{34}O_5SNa$ $[M + Na]^+$ 565.2019; found 565.2018.

^{1}H NMR (400 MHz, $CDCl_3$) of **S7**:

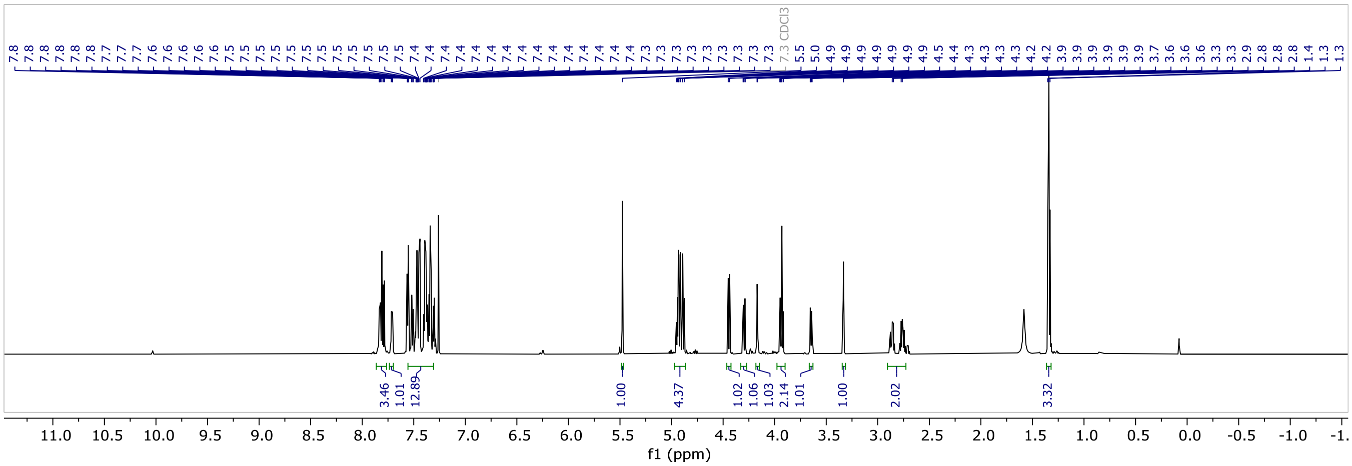

^{13}C NMR (101 MHz, $CDCl_3$) of **S7**:

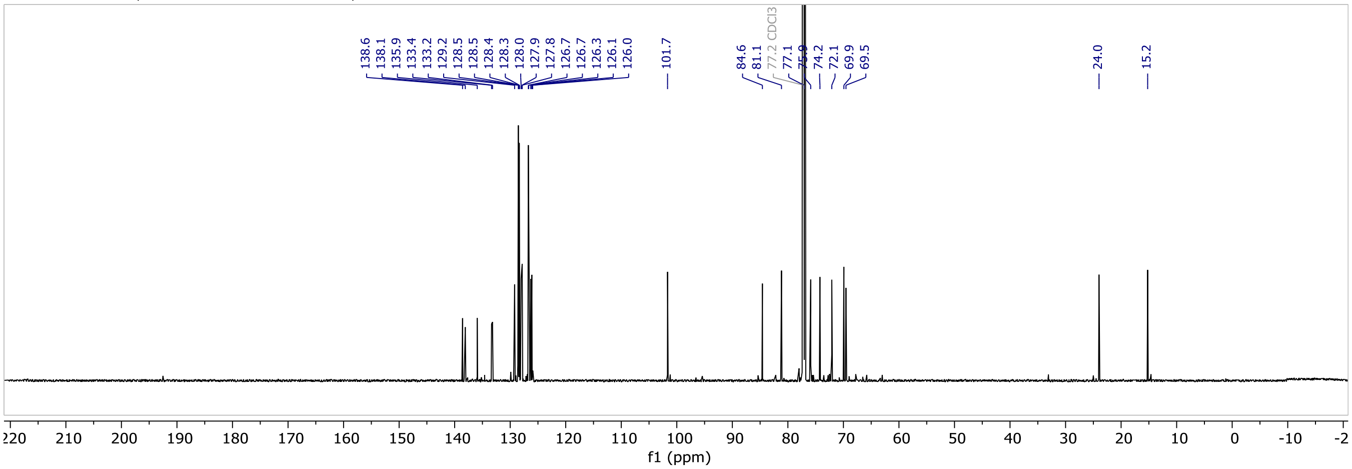

$^{13}C,^{1}H$ HSQC of **S7**:

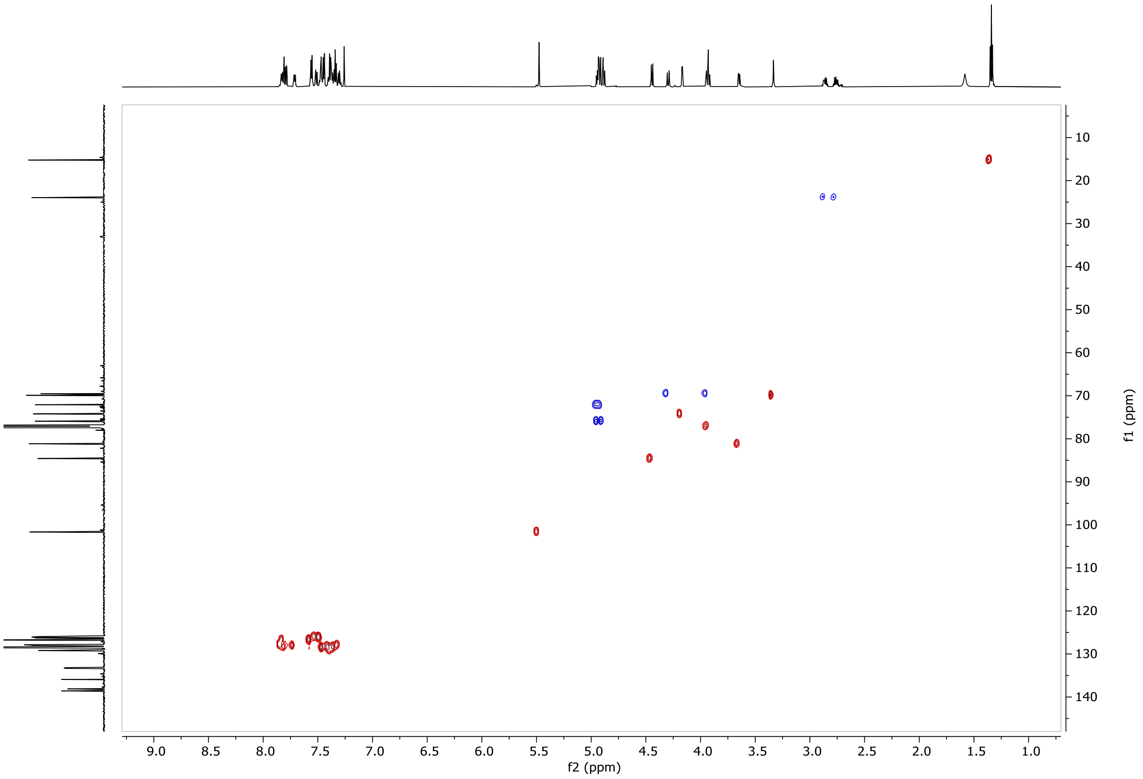

$^{1}H,^{1}H$ COSY of **S7**:

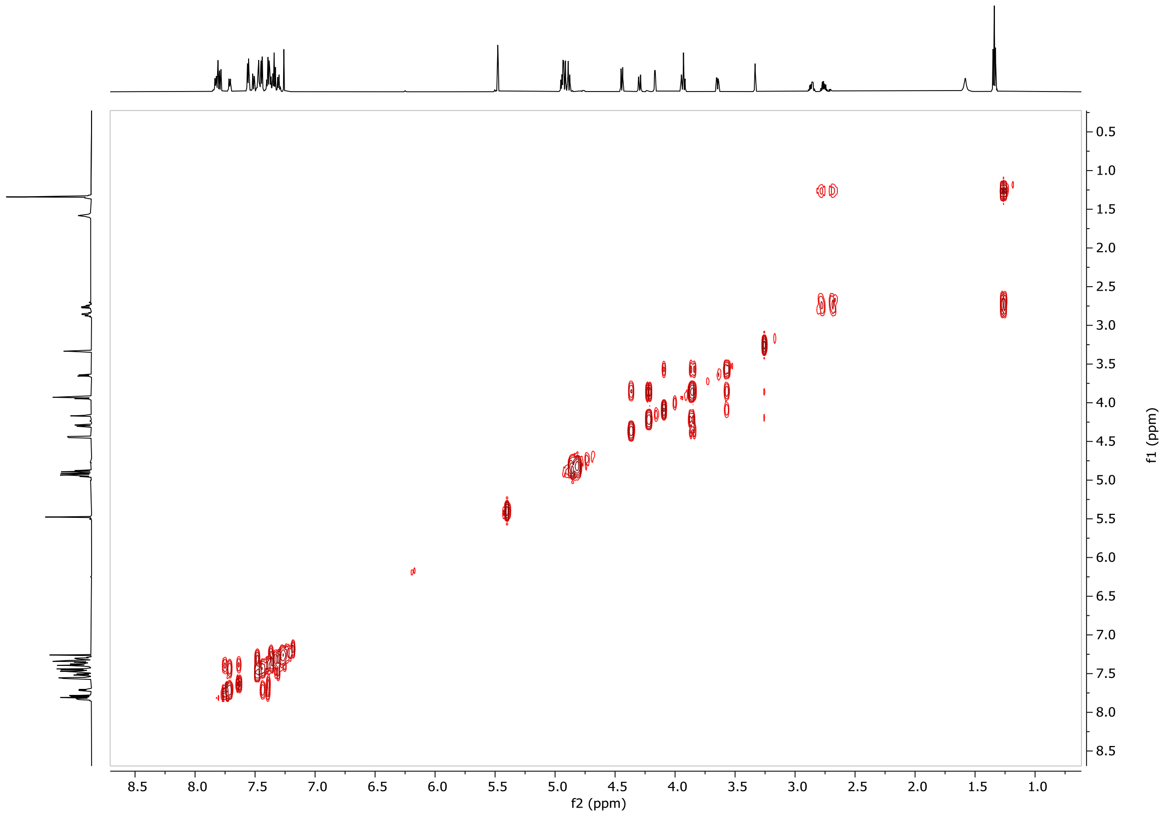

Ethyl 2,6-bis-*O*-benzyl-3-*O*-(2-naphthalenylmethyl)-1-thio-β-D-galactopyranoside[22] (S8)

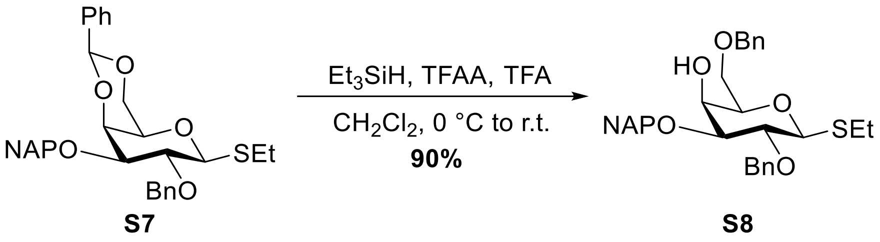

Compound **S7** (2.0 g, 3.7 mmol, 1.0 equiv.) was co-evaporated with anhydrous toluene (2 x 3 mL), and dissolved in anhydrous CH_2Cl_2 (20 mL). Triethylsilane (3.5 mL, 22.1 mmol, 6.0 equiv.) and trifluoroacetic anhydride (0.52 mL, 3.7 mmol, 1.0 equiv.) were added and the solution was cooled to 0 °C. Trifluoroacetic acid (1.7 mL, 22.1 mmol, 6.0 equiv.) was added dropwise. The mixture was allowed to warm up to room temperature and was stirred for 5 h. The solution was diluted with CH_2Cl_2 and quenched with saturated aqueous $NaHCO_3$ (40 mL). The aqueous phase was extracted with CH_2Cl_2 (2 x 60 mL) and the combined organic phase was washed with water (60 mL), dried over Na_2SO_4, filtered and concentrated. Product **S8** (1.8 g, 3.3 mmol, **90%**) was obtained as a colorless syrup after purification by column chromatography (SiO_2, Hex/EtOAc = 3:1 to 1:1).

$\mathbf{R_f}$ = 0.39 (Hex/EtOAc 1:1).

^{1}H NMR (400 MHz, $CDCl_3$) δ 7.88 – 7.71 (m, 4H), 7.53 – 7.27 (m, 13H), 4.95 – 4.77 (m, 4H), 4.58 (s, 2H), 4.44 (d, *J* = 9.8 Hz, 1H), 4.14 (dd, *J* = 3.3, 1.1 Hz, 1H), 3.83 – 3.68 (m, 3H), 3.64 – 3.52 (m, 2H), 2.87 – 2.66 (m, 2H), 1.32 (t, *J* = 7.5 Hz, 3H) ppm.

^{13}C NMR (101 MHz, $CDCl_3$) δ 138.3, 138.0, 135.3, 133.3, 133.2, 128.6, 128.5, 128.5, 128.0, 127.9, 127.9, 127.8, 126.8, 126.3, 126.2, 125.9, 85.2, 82.3, 78.0, 77.0, 76.0, 73.9, 72.3, 69.5, 67.1, 24.9, 15.3 ppm.

^{1}H NMR (400 MHz, $CDCl_3$) of **S8**:

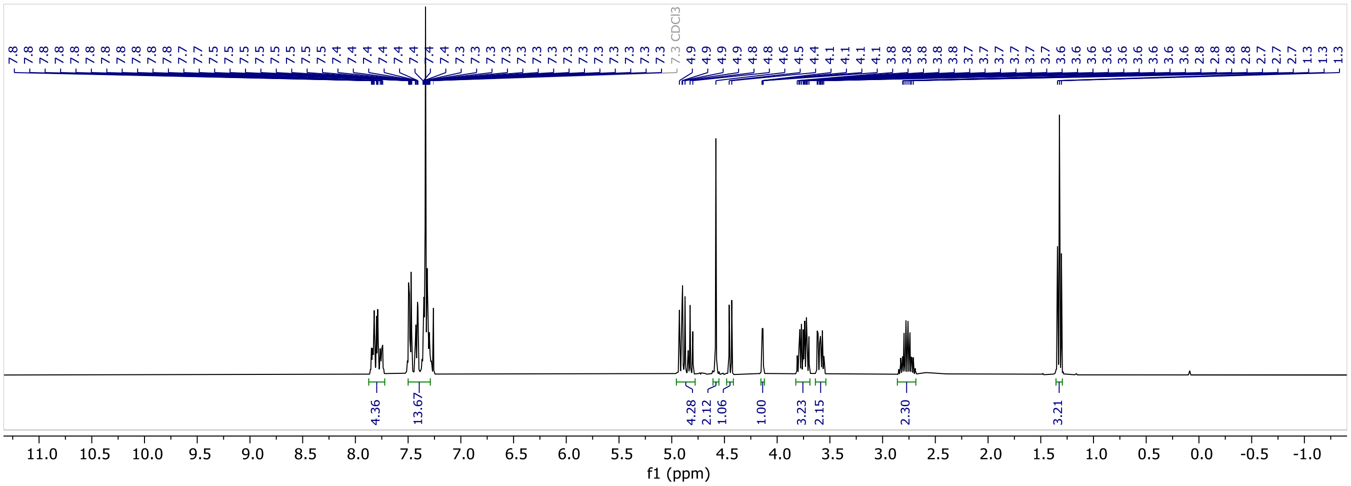

^{13}C NMR (101 MHz, $CDCl_3$) of **S8**:

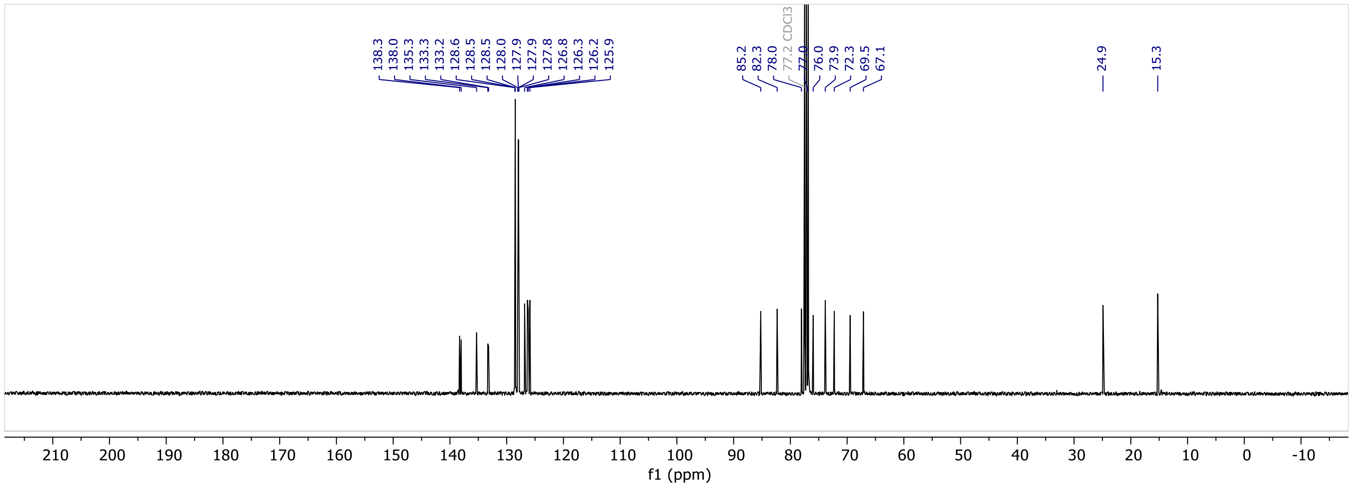

$^{13}C,^{1}H$ HSQC of **S8**:

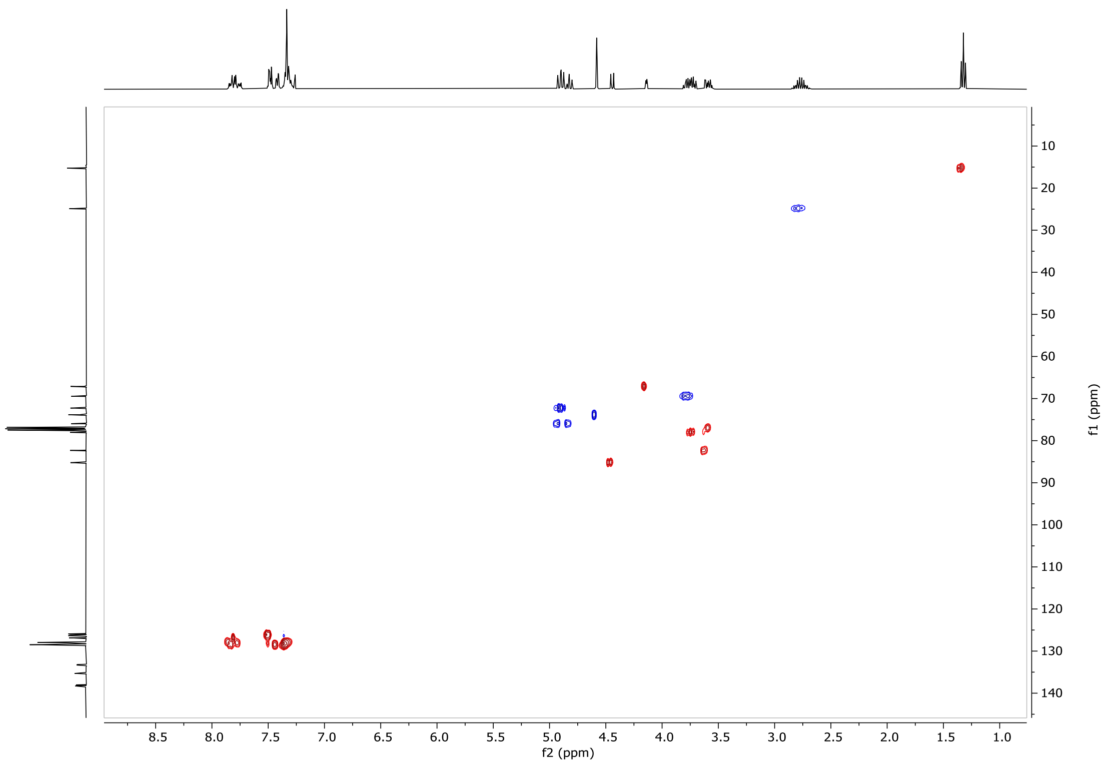

$^{1}H,^{1}H$ COSY of **S8**:

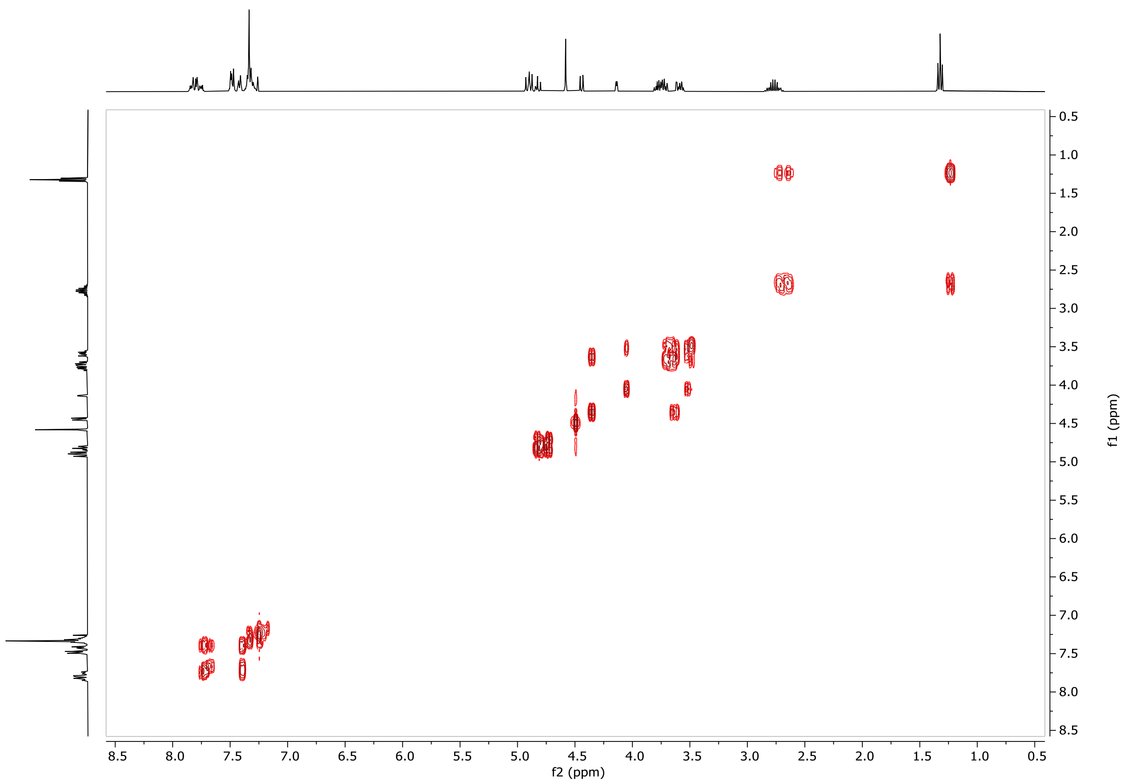

Ethyl 2,6-bis-*O*-benzyl-3-*O*-(2-naphthalenylmethyl)-4-(2,2-dimethylpropanoate)-1-thio-β-D-galactopyranoside (S9)

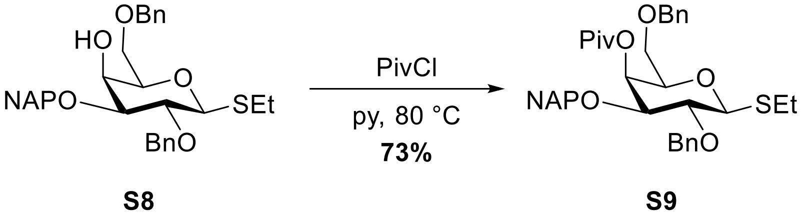

To a solution of **S8** (1.3 g, 2.4 mmol, 1.0 equiv.) in anhydrous pyridine (15 mL) pivaloyl chloride (1.5 mL, 12.0 mmol, 5.0 equiv.) was added. The mixture was stirred at 80 °C for 2 hours. The volatiles were evaporated. Product **S9** (1.1 g, 1.7 mmol, **73%**) was obtained as a colorless oil after purification by column chromatography (SiO_2, Hex/EtOAc = 9:1 to 3:1).

R*f* = 0.43 (Hex/EtOAc 3:1).

^{1}H NMR (400 MHz, $CDCl_3$) δ 7.84 – 7.68 (m, 4H), 7.50 – 7.41 (m, 3H), 7.39 – 7.27 (m, 10H), 5.68 (dd, *J* = 3.3, 1.1 Hz, 1H), 4.91 (d, *J* = 11.2 Hz, 1H), 4.80 (q, *J* = 10.4 Hz, 2H), 4.63 (d, *J* = 11.2 Hz, 1H), 4.57 – 4.44 (m, 4H), 3.77 (ddd, *J* = 7.2, 5.8, 1.1 Hz, 1H), 3.67 (dd, *J* = 9.1, 3.3 Hz, 1H), 3.61 – 3.52 (m, 2H), 3.47 (dd, *J* = 9.5, 7.1 Hz, 1H), 1.31 (t, *J* = 7.5 Hz, 3H), 1.21 (s, 9H) ppm.

^{13}C NMR (101 MHz, $CDCl_3$) δ 177.7, 138.2, 135.6, 133.4, 133.1, 128.6, 128.6, 128.4, 128.2, 128.1, 127.9, 127.8, 127.0, 126.4, 126.1, 126.0, 85.2, 81.4, 77.0, 76.1, 75.9, 73.9, 71.9, 68.4, 66.6, 27.4, 24.7, 15.2 ppm.

HRMS (QToF): Calcd for $C_{38}H_{44}O_6SNa$ $[M + Na]^+$ 651.2751; found 651.2740.

^{1}H NMR (400 MHz, $CDCl_3$) of **S9**:

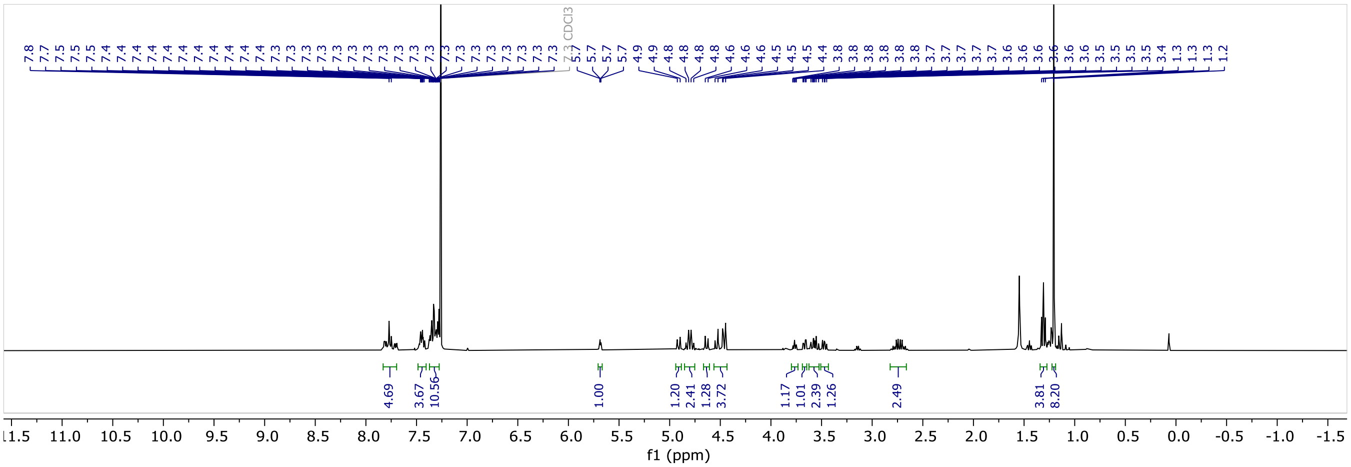

^{13}C NMR (101 MHz, $CDCl_3$) of **S9**:

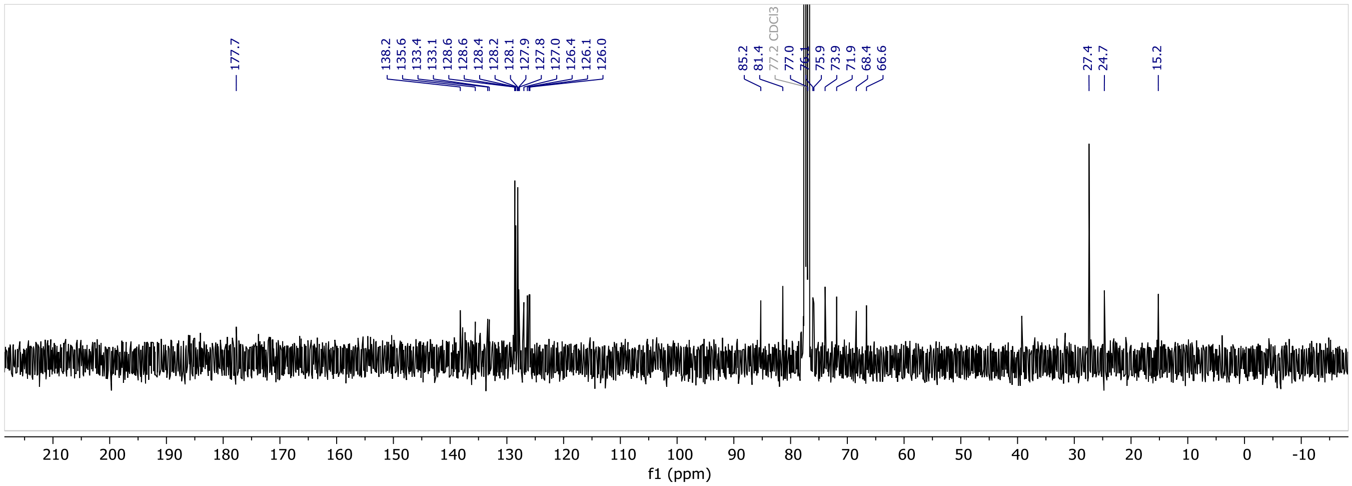

^{13}C,^{1}H HSQC of **S9**:

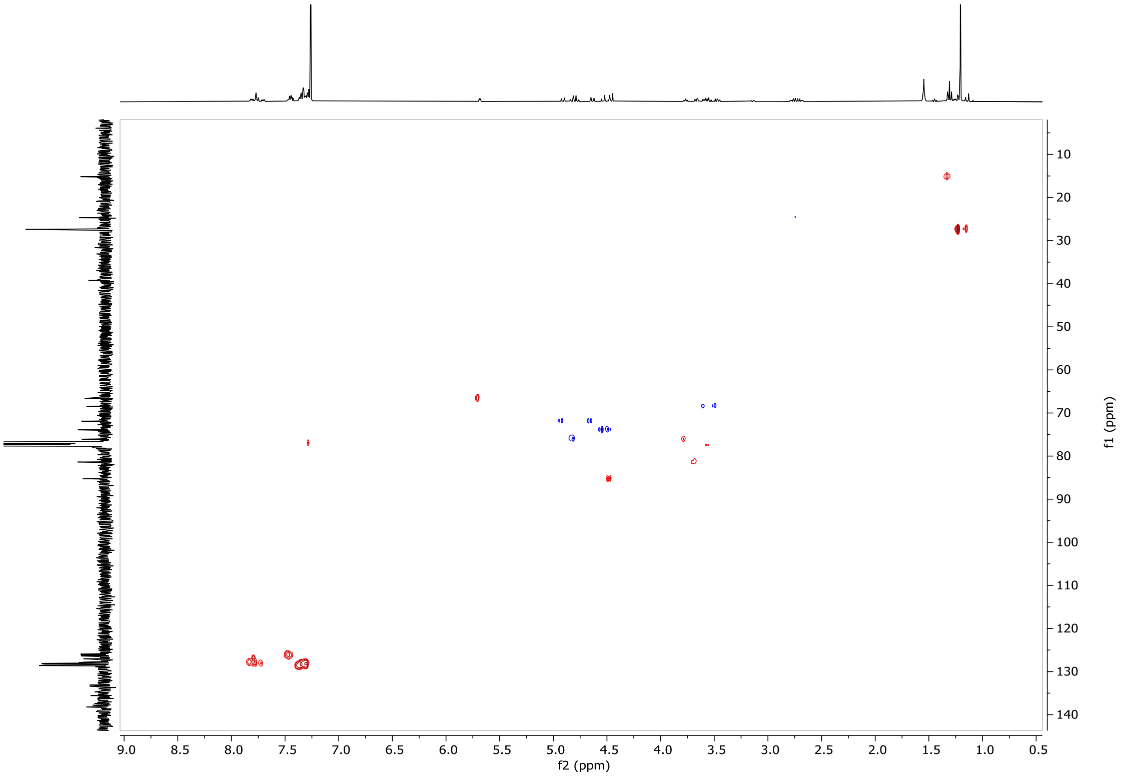

$^{1}H,^{1}H$ COSY of **S9**:

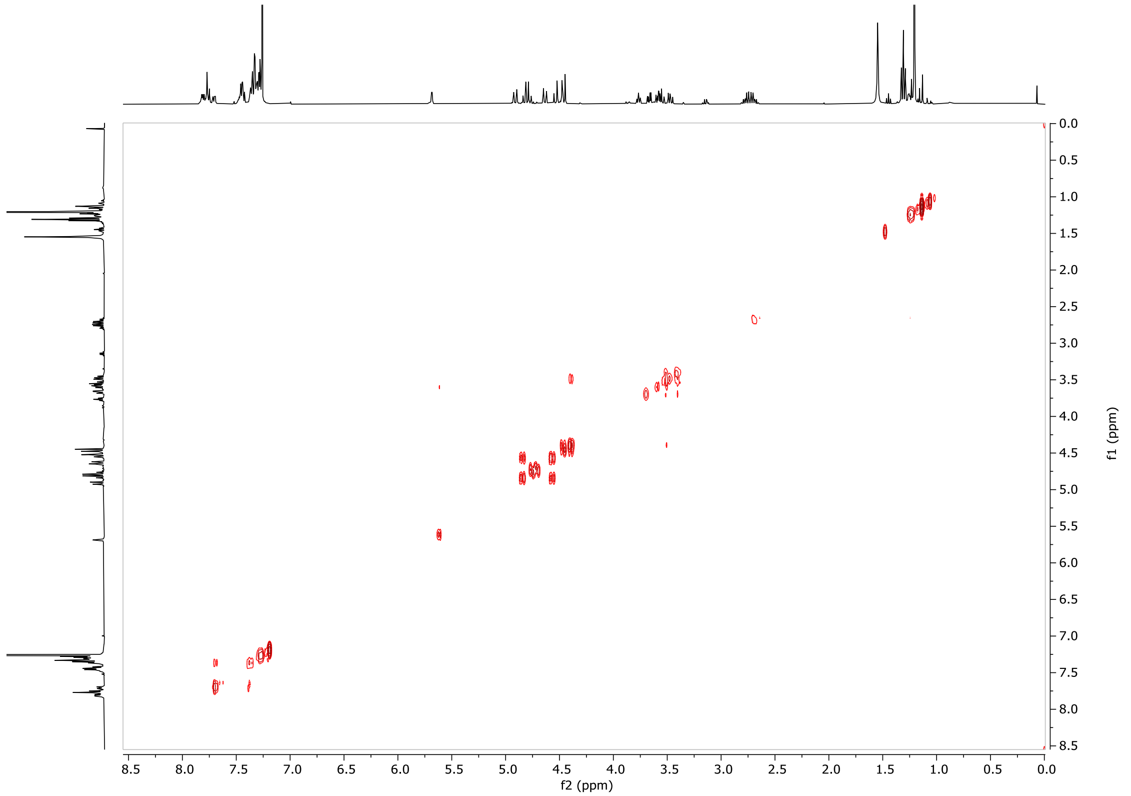

Ethyl 2,6-bis-*O*-benzyl-4-(2,2-dimethylpropanoate)-1-thio-β-D-galactopyranoside (S10)

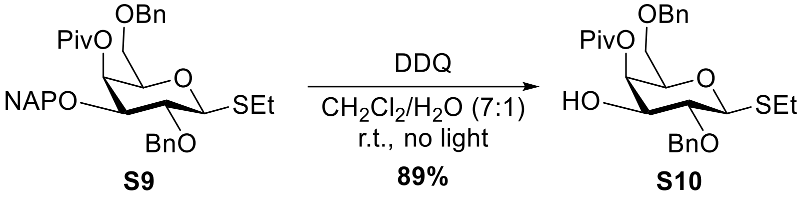

To a well stirred emulsion of **S9** (1.3 g, 2.1 mmol, 1.0 equiv.) in CH_2Cl_2/water (7:1, 24 mL), was added DDQ (517 mg, 2.3 mmol, 1.1 equiv.) and the suspension was stirred at room temperature for 1.5 h protected from light. The mixture was diluted with CH_2Cl_2, washed with 10% $Na_2S_2O_3$ and saturated aqueous $NaHCO_3$ solution. The organic layer was dried over Na_2SO_4, filtered, concentrated and the residue was purified by column chromatography (SiO_2, Hex/EtOAc = 9:1 to 3:1) to obtain the title compound **S10** (900 mg, 1.8 mmol, 89%) as a colorless solid.

^{1}H NMR (600 MHz, $CDCl_3$) δ 7.41 – 7.27 (m, 10H), 5.39 (dd, *J* = 3.4, 1.2 Hz, 1H), 4.93 (d, *J* = 11.0 Hz, 1H), 4.69 (d, *J* = 11.0 Hz, 1H), 4.53 – 4.43 (m, 3H), 3.82 – 3.76 (m, 2H), 3.55 (dd, *J* = 9.6, 6.2 Hz, 1H), 3.49 – 3.41 (m, 2H), 2.85 – 2.67 (m, 2H), 1.34 (t, *J* = 7.5 Hz, 3H), 1.18 (s, 9H) ppm.

^{13}C NMR (151 MHz, $CDCl_3$) δ 178.5, 137.9, 137.8, 128.8, 128.7, 128.6, 128.3, 128.0, 127.9, 85.1, 78.2, 76.3, 75.4, 74.1, 73.8, 69.8, 68.5, 39.3, 27.3, 25.0, 15.2 ppm.

HRMS (QToF): Calcd for $C_{27}H_{36}O_6SNa$ $[M + Na]^+$ 511.2125; found 511.2217.

^{1}H NMR (400 MHz, $CDCl_3$) of **S10**:

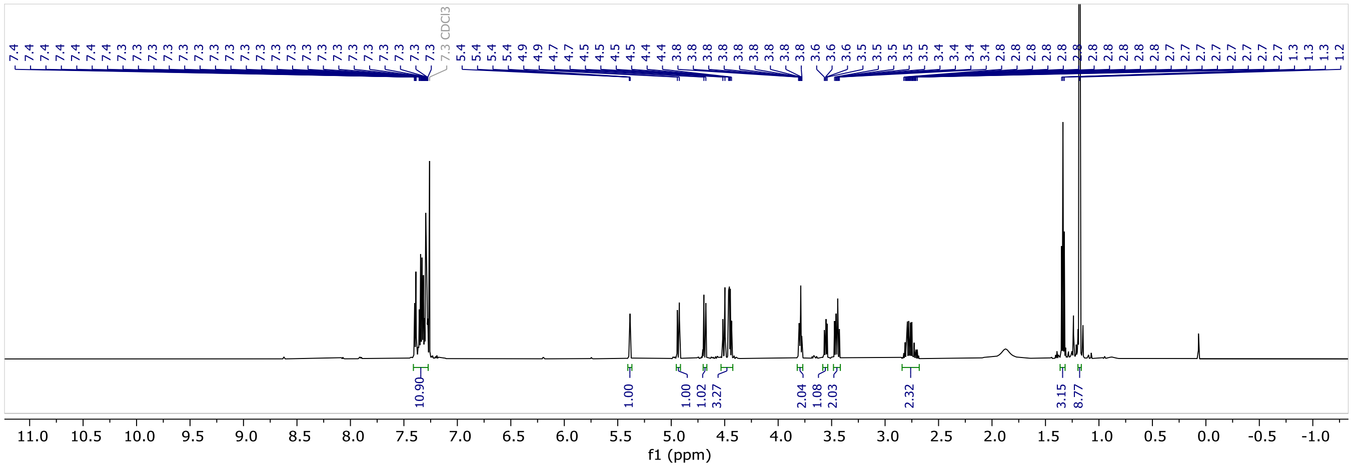

^{13}C NMR (101 MHz, $CDCl_3$) of **S10**:

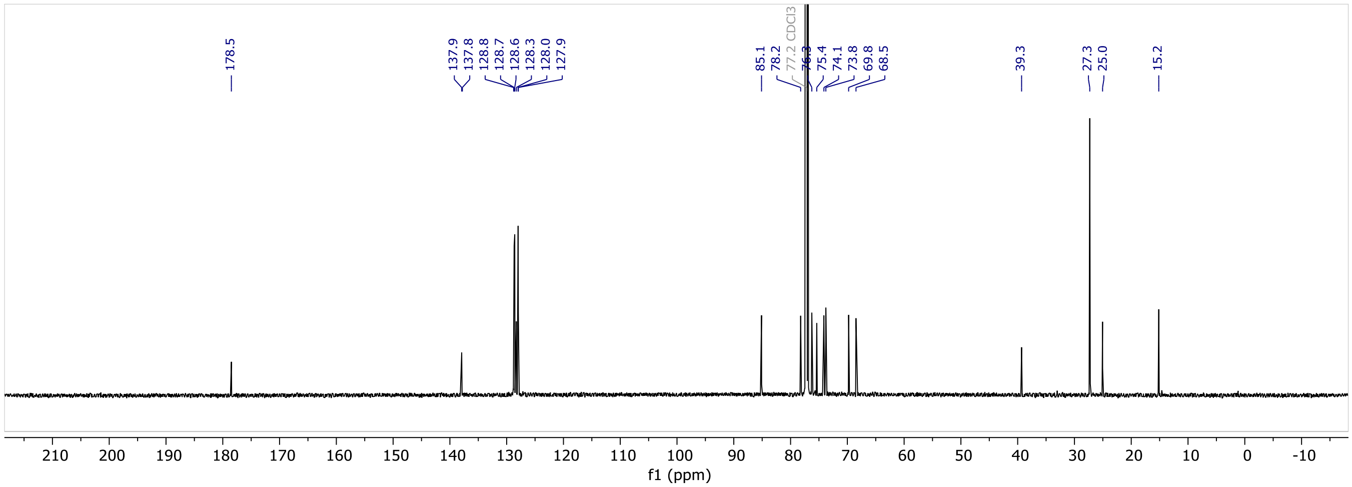

$^{13}C,^{1}H$ HSQC of **S10**:

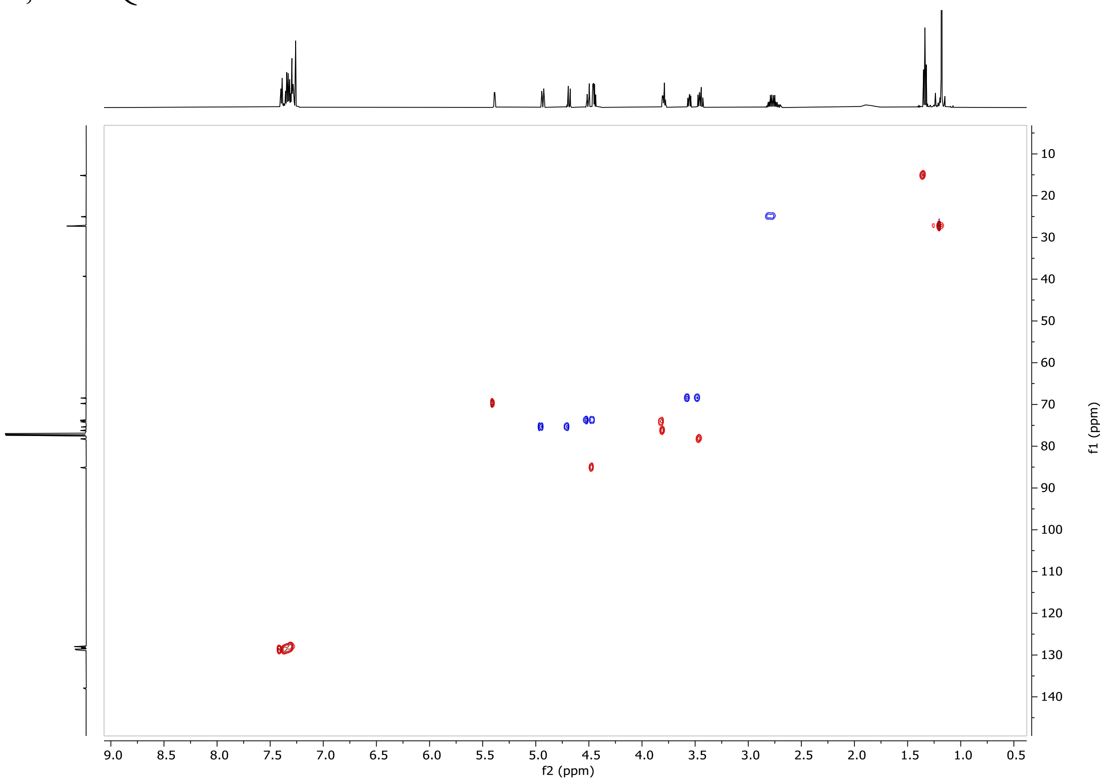

$^{1}H,^{1}H$ COSY of **S10**:

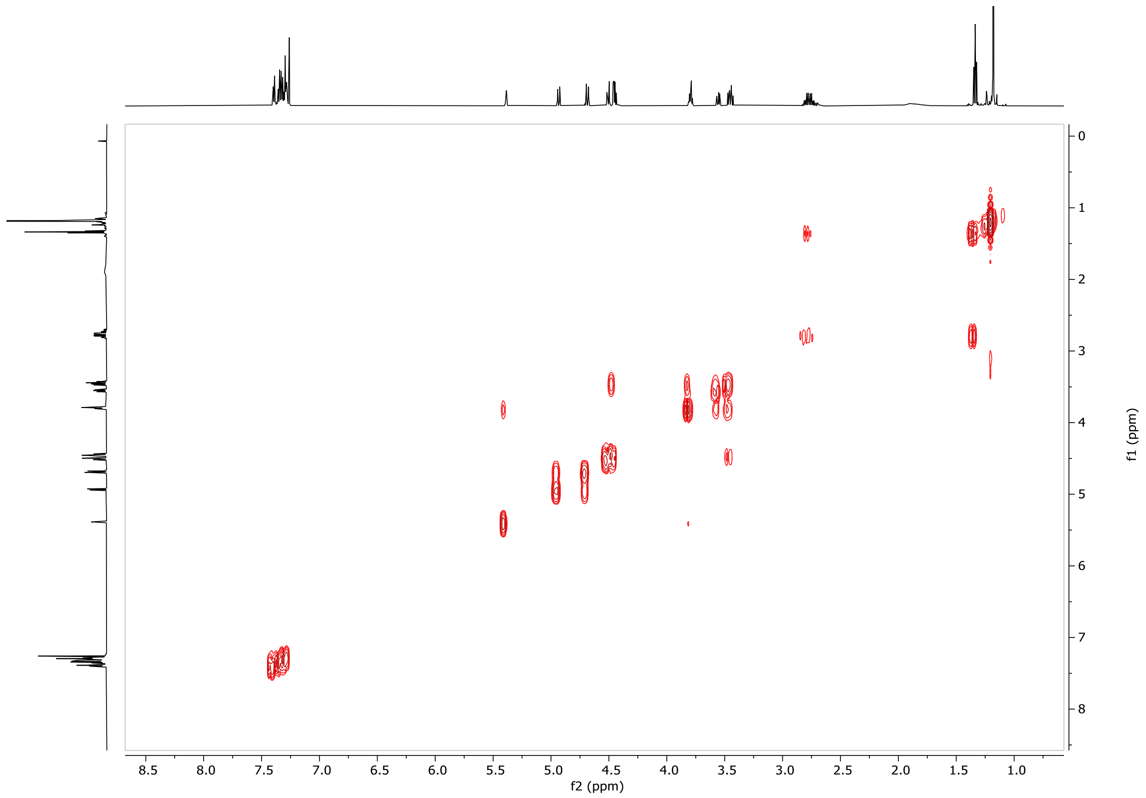

Ethyl 2,6-bis-*O*-benzyl-4-(2,2-dimethylpropanoate)-4-*O*-fluorenylmethoxycarbonyl-1-thio-β-D-galactopyranoside (S11)

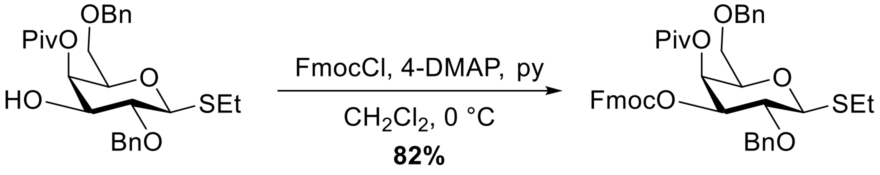

S10 was dissolved in anhydrous CH_2Cl_2 (20 mL) and anhydrous pyridine (0.41 mL, 5.12 mmol, 5.0 equiv.) was added followed by FmocCl (0.4 g, 1.54 mmol, 1.5 equiv.) at 0 °C. 4-DMAP (6 mg, 0.05 mmol, 0.05 equiv.) was added and the reaction mixture was stirred for one hour at 0 °C. Aqueous citric acid solution (10 mL) was added and the mixture was allowed to warm up to room temperature. The aqueous phase was extracted with CH_2Cl_2 and the combined organic phase was dried over Na_2SO_4, filtered and concentrated. The title compound **S11** (600 mg, 0.84 mmol, **82%**) was obtained as a white solid after purification by column chromatography (SiO_2, Hex/EtOAc = 9:1 to 3:1).

^{1}H NMR (600 MHz, $CDCl_3$) δ 7.78 (ddt, *J* = 7.5, 2.0, 0.9 Hz, 2H), 7.63 (ddd, *J* = 9.2, 7.4, 0.9 Hz, 2H), 7.42 (tt, *J* = 7.5, 1.5 Hz, 2H), 7.38 – 7.26 (m, 12H), 5.62 (dd, *J* = 3.4, 1.1 Hz, 1H), 4.92 – 4.85 (m, 2H), 4.72 (d, *J* = 10.8 Hz, 1H), 4.57 – 4.43 (m, 4H), 4.34 – 4.25 (m, 2H), 3.88 (td, *J* = 6.4, 1.2 Hz, 1H), 3.67 (t, *J* = 9.6 Hz, 1H), 3.58 (dd, *J* = 9.6, 6.2 Hz, 1H), 3.46 (dd, *J* = 9.6, 6.5 Hz, 1H), 2.86 – 2.70 (m, 2H), 1.35 (t, *J* = 7.4 Hz, 3H), 1.22 (s, 9H) ppm.

^{13}C NMR (151 MHz, $CDCl_3$) δ 177.5, 154.3, 143.8, 143.4, 141.4, 137.7, 137.6, 128.6, 128.5, 128.5, 128.0, 128.0, 127.9, 127.3, 127.3, 125.4, 125.3, 120.1, 85.2, 78.7, 75.9, 75.6, 75.5, 73.7, 70.3, 68.1, 67.7, 46.8, 39.3, 27.3, 25.0, 15.1 ppm.

HRMS (QToF): Calcd for $C_{42}H_{46}O_8SNa$ $[M + Na]^+$ 733.2806; found 733.2787.

^{1}H NMR (400 MHz, $CDCl_3$) of **S11**:

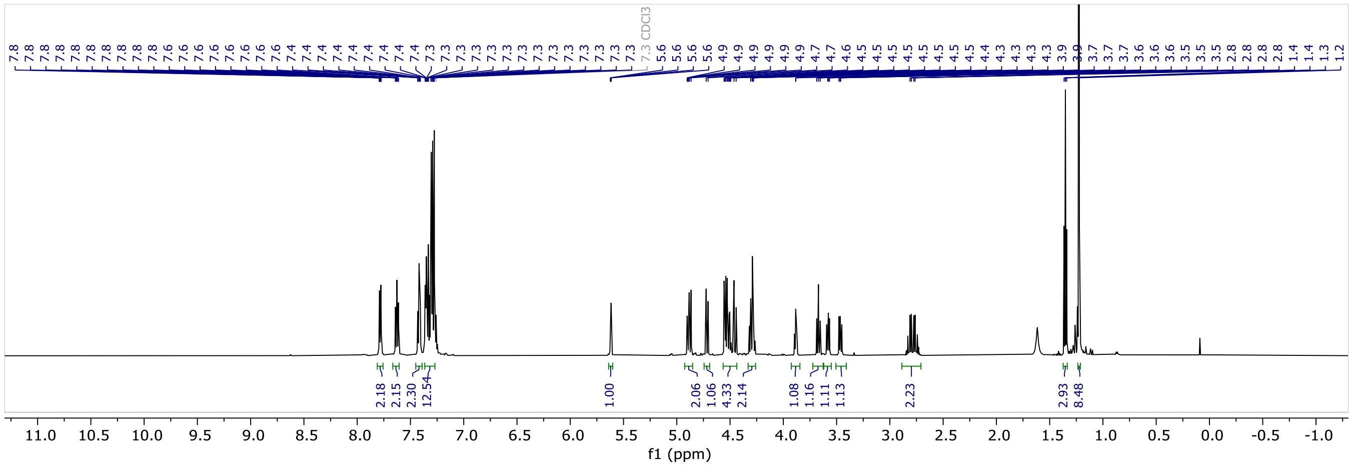

^{13}C NMR (101 MHz, $CDCl_3$) of **S11**:

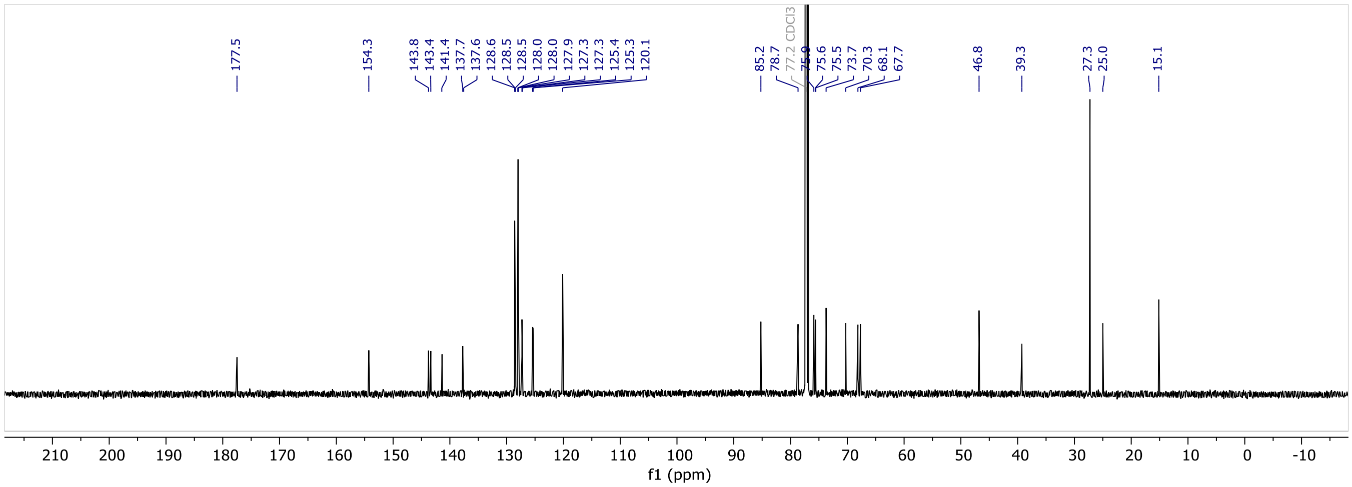

$^{13}C,^{1}H$ HSQC of **S11**:

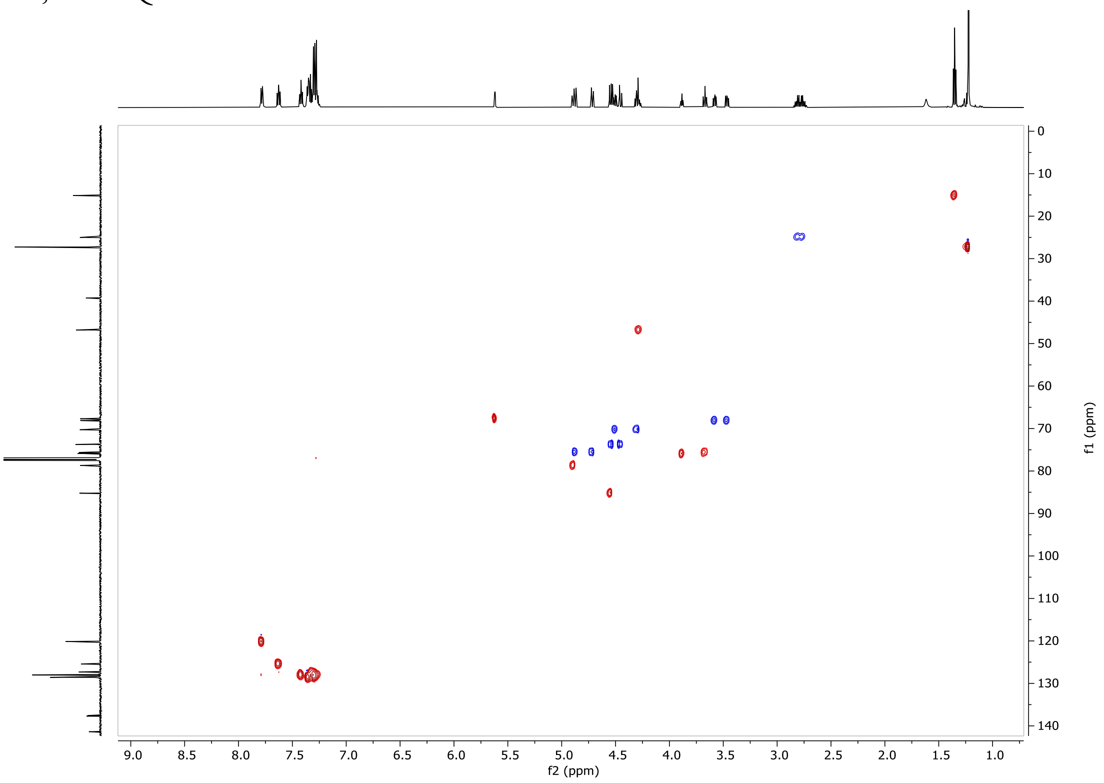

Dibutoxyphosphoryloxy 2,6-bis-*O*-benzyl-4-(2,2-dimethylpropanoate)-4-*O*-fluorenylmethoxycarbonyl-α-D-galactopyranoside (S12)

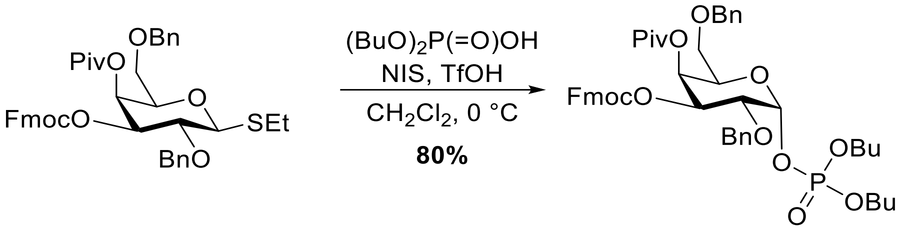

S11 **S12**

To a solution of **S11** (300 mg, 0.4 mmol, 1.0 equiv.) and dibutyl phosphate (168 μL, 0.8 mmol, 2.0 equiv.) in anhydrous CH_2Cl_2 (4 mL) was added NIS (171 mg, 0.8 mmol, 1.5 equiv.) and TfOH (11 μL, 0.1 mmol, 0.3 equiv.) at 0 °C. The reaction was stirred for 1 h. To the reaction mixture was added 10% sodium thiosulfate solution. The bilayer mixture was extracted with CH_2Cl_2, dried with Na_2SO_4, filtered, concentrated and purified by flash column chromatography (SiO_2, Hex/EtOAc = 3:1 to 2:3) to give title compound **S12** (290 mg, 0.3 mmol, **80%**) as a colorless foam.

R*f* = 0.16 (Hex/EtOAc 3:1).

^{1}**H NMR** (600 MHz, $CDCl_3$) δ 7.77 (ddt, *J* = 7.6, 1.8, 0.9 Hz, 2H), 7.63 (tt, *J* = 7.5, 1.0 Hz, 2H), 7.41 (t, *J* = 7.1 Hz, 2H), 7.37 – 7.24 (m, 12H), 5.96 (dd, *J* = 7.3, 3.4 Hz, 1H), 5.63 (dd, *J* = 3.4, 1.5 Hz, 1H), 5.16 (dd, *J* = 10.4, 3.3 Hz, 1H), 4.81 – 4.62 (m, 2H), 4.56 – 4.36 (m, 4H), 4.35 – 4.24 (m, 2H), 4.09 – 3.97 (m, 4H), 3.88 (dt, *J* = 10.4, 2.9 Hz, 1H), 3.54 – 3.39 (m, 2H), 1.61 – 1.53 (m, 4H), 1.40 – 1.23 (m, 4H), 1.14 (s, 9H), 0.86 (dt, *J* = 15.0, 7.4 Hz, 6H) ppm.

^{13}**C NMR** (151 MHz, $CDCl_3$) δ 177.4, 154.3, 143.8, 143.4, 141.4, 137.7, 137.3, 128.5, 128.5, 128.2, 128.1, 128.0, 128.0, 127.9, 127.3, 127.2, 125.4, 125.4, 120.2, 95.2, 73.7, 73.7, 72.7, 70.3, 69.8, 68.0, 67.8, 46.8, 39.2, 32.2, 27.2, 18.7, 13.7 ppm.

HRMS (QToF): Calcd for $C_{48}H_{59}O_{12}PNa$ $[M + Na]^+$ 881.3636; found 881.3759.

^{1}H NMR (400 MHz, $CDCl_3$) of **S12**:

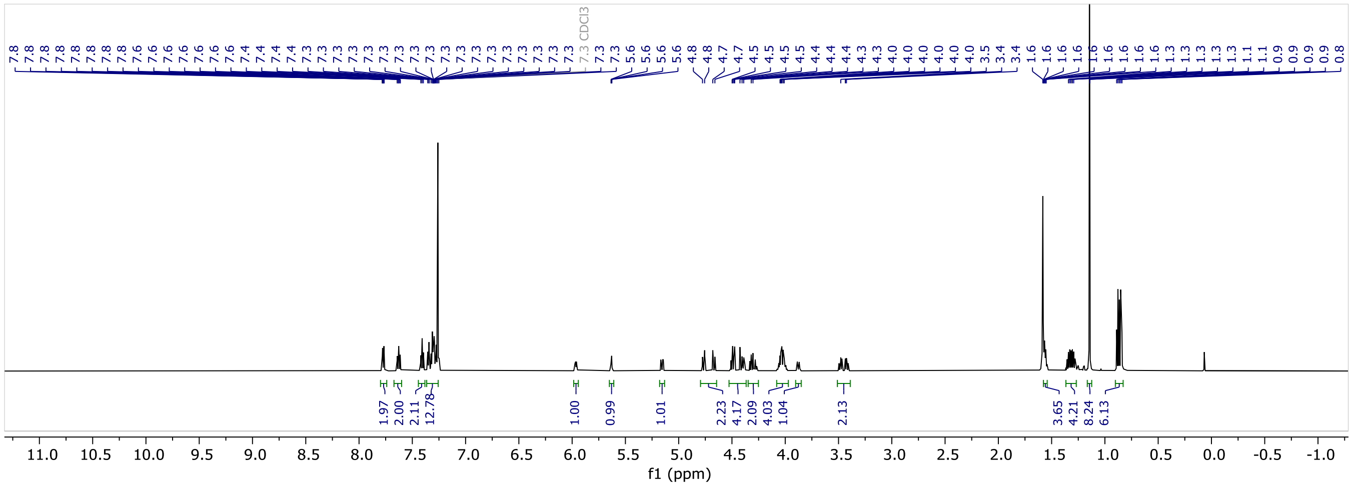

^{13}C NMR (101 MHz, $CDCl_3$) of **S12**:

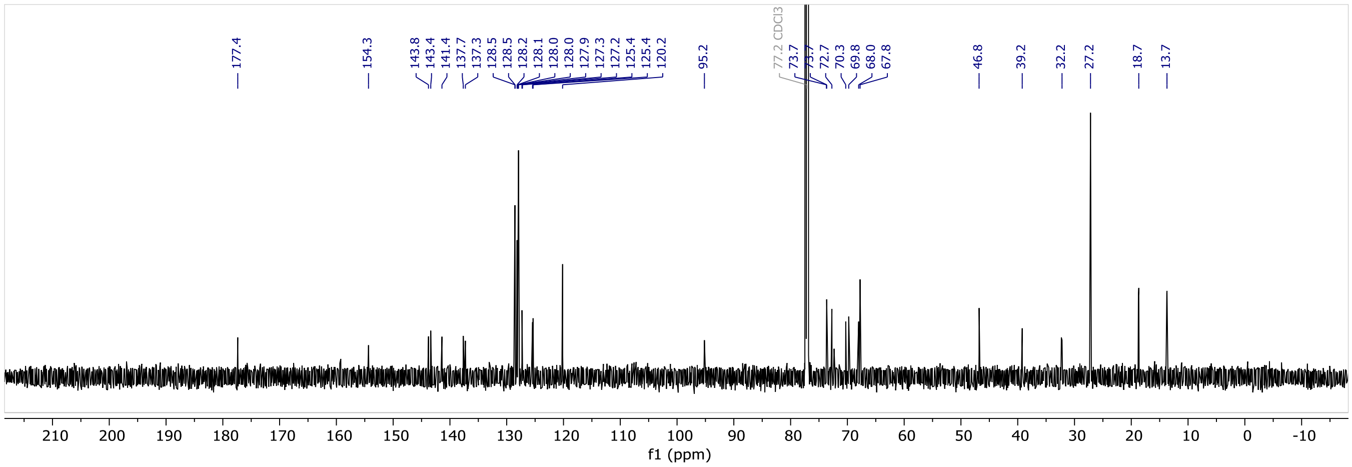

Coupled ^{13}C,^{1}H HSQC of **S12**:

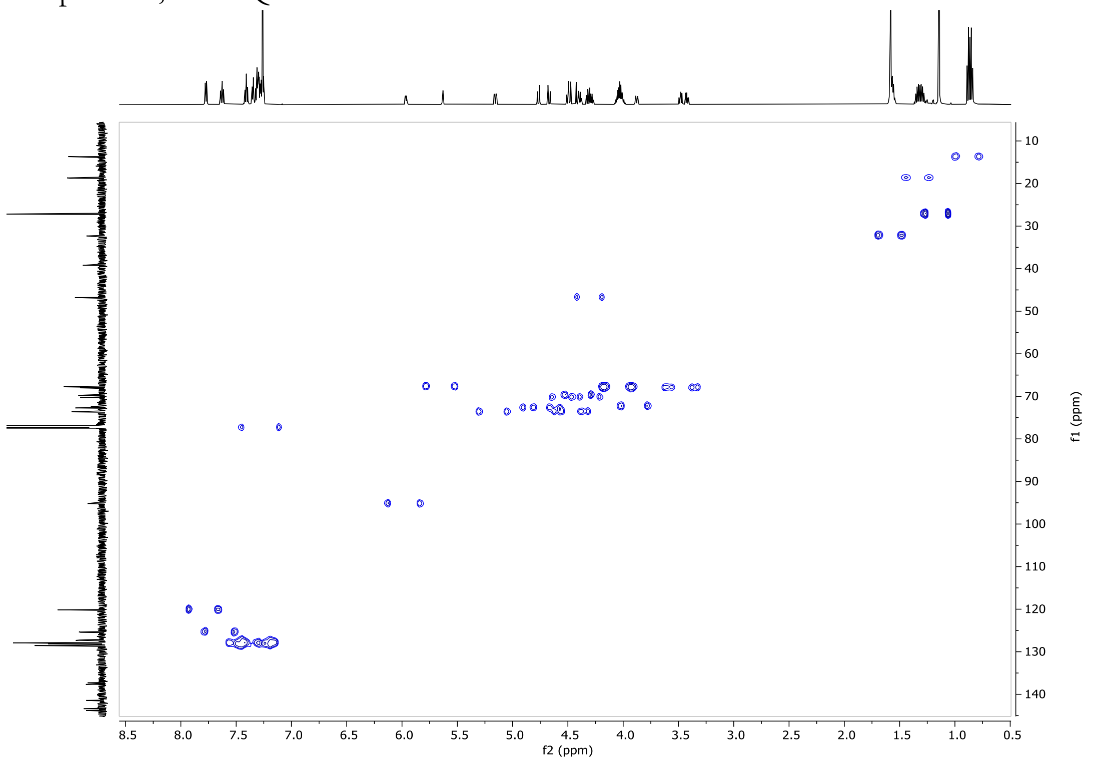

^{13}C,^{1}H HSQC of **S12**:

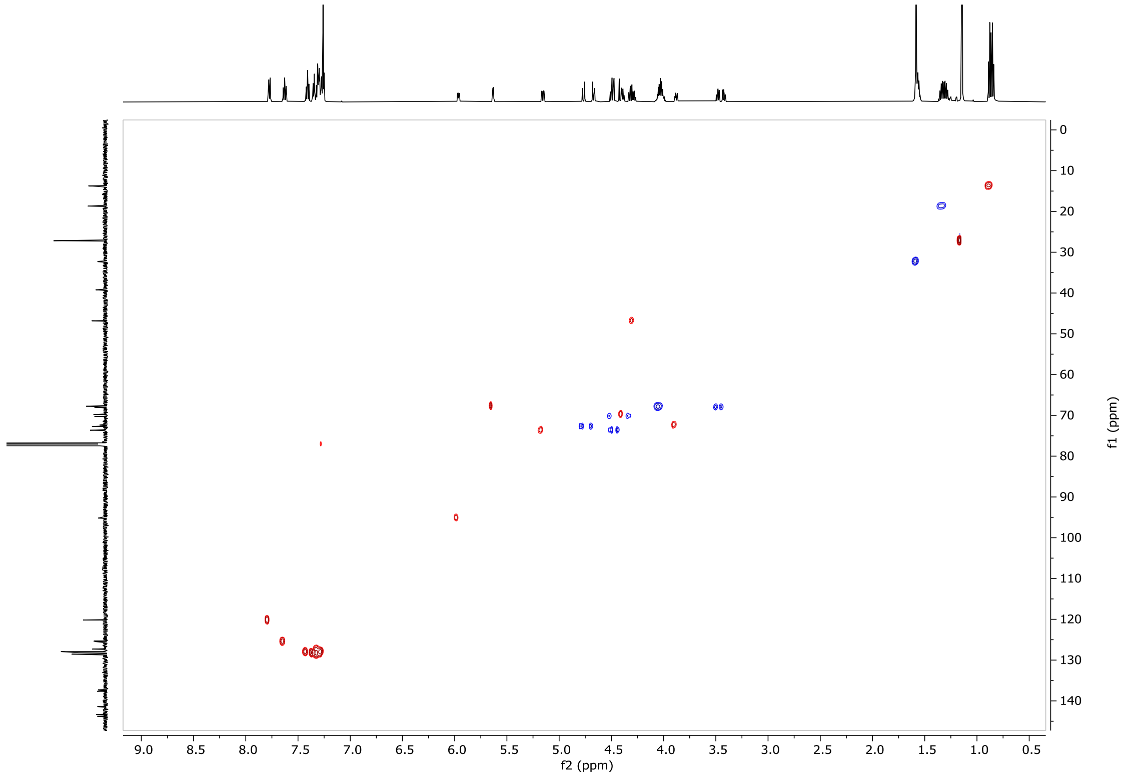

6 Determination of alpha/beta-ratios

1,2:3,4-Bis-*O*-(1-methylethylidene)-6-*O*-[2,3,4-tris-*O*-benzyl-6-(2,2-dimethylpropanoate)-α/β-D-galactopyranosyl]-α-D-galactopyranoside (6Piv-A1)

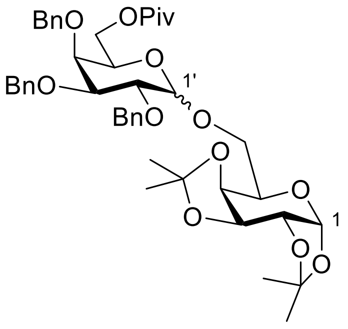

The title compound was prepared according to general procedure for glycosylations. Product **6Piv-A1** (20 mg, 26 µmol, 75%, **α/β 53:47**) was obtained as a colorless oil after purification using **Method-2b** (t_R (α) = 9.8 min, t_R (β) = 9.1 min).

Data of the anomeric mixture:

^{1}H NMR (600 MHz, $CDCl_3$) δ 7.47 – 7.27 (m, 30H), 5.56 (d, *J* = 5.0 Hz, 1H, **H_1(β)**), 5.50 (d, *J* = 5.0 Hz, 1H, **H_1(α)**), 5.05 (d, *J* = 11.1 Hz, 1H), 5.00 (d, *J* = 3.7 Hz, 1H, **H_1'(α)**), 4.97 (dd, *J* = 11.3, 5.1 Hz, 2H), 4.87 (dd, *J* = 18.6, 11.9 Hz, 2H), 4.79 – 4.70 (m, 5H), 4.62 (d, *J* = 11.5 Hz, 1H), 4.60 – 4.56 (m, 3H), 4.41 (d, *J* = 7.7 Hz, 1H, **H_1'(β)**), 4.32 – 4.28 (m, 3H), 4.28 – 4.24 (m, 1H), 4.23 – 4.16 (m, 2H), 4.13 – 4.00 (m, 8H), 3.97 (dd, *J* = 10.0, 2.8 Hz, 1H), 3.88 – 3.82 (m, 2H), 3.76 (d, *J* = 6.6 Hz, 1H), 3.73 – 3.66 (m, 3H), 3.55 – 3.49 (m, 2H), 1.51 (s, 3H), 1.48 (s, 3H), 1.43 (s, 4H), 1.32 (s, 3H), 1.31 (s, 6H), 1.30 (s, 3H), 1.17 (s, 9H), 1.15 (s, 9H) ppm.

^{13}C NMR (151 MHz, $CDCl_3$) δ 178.2, 139.1, 139.0, 138.8, 138.7, 138.6, 138.5, 137.3, 128.7, 128.6, 128.5, 128.5, 128.4, 128.4, 128.3, 128.3, 127.9, 127.8, 127.8, 127.8, 127.7, 127.7, 127.7, 127.6, 127.5, 109.5, 109.4, 108.7, 108.6, 104.8 (**C_1'(β)**), 97.5 (**C_1'(α)**), 96.5 (**C_1(β)**), 96.4 (**C_1(α)**), 82.0, 79.2, 79.0, 76.5, 75.5, 74.9, 74.9, 74.7, 74.0, 73.7, 73.5, 72.9, 72.1, 71.6, 71.1, 70.9, 70.8, 70.8, 70.7, 69.9, 69.7, 68.7, 67.8, 67.6, 66.6, 66.2, 63.3, 62.7, 38.8, 27.4, 27.3, 26.3, 26.2, 26.1, 25.2, 25.1, 24.6, 24.6 ppm.

HRMS (QToF): Calcd for $C_{44}H_{56}O_{12}Na$ $[M + Na]^+$ 799.3664; found 799.3670.

NP-HPLC of **P1-SL101** (ELSD trace, t_R (α) = 9.8 min, t_R (β) = 9.1 min):

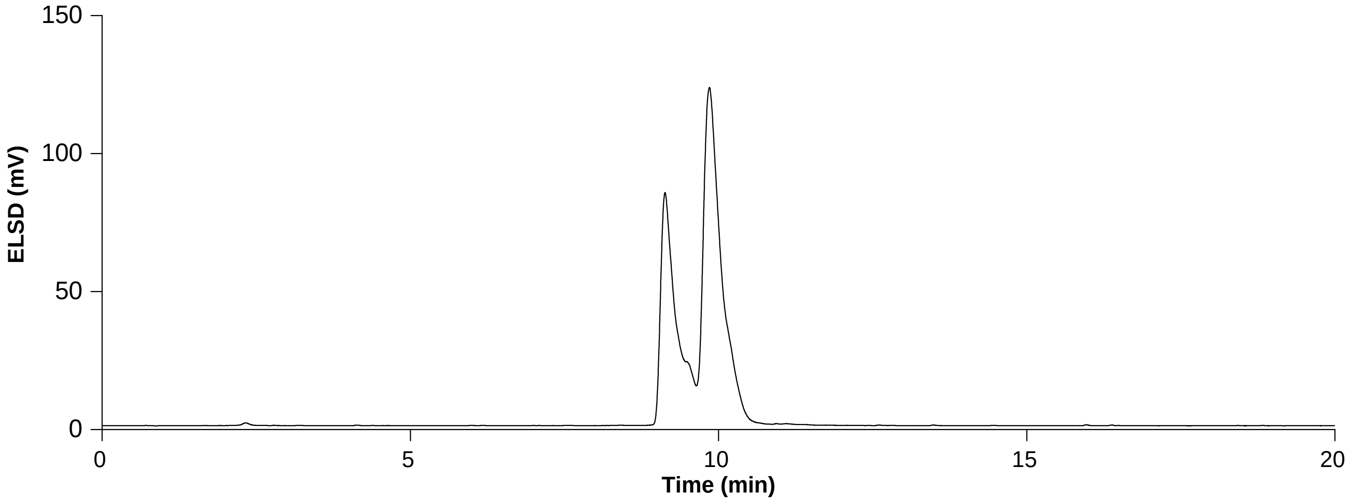

^{1}H NMR (600 MHz, $CDCl_3$) of **P1-SL101**:

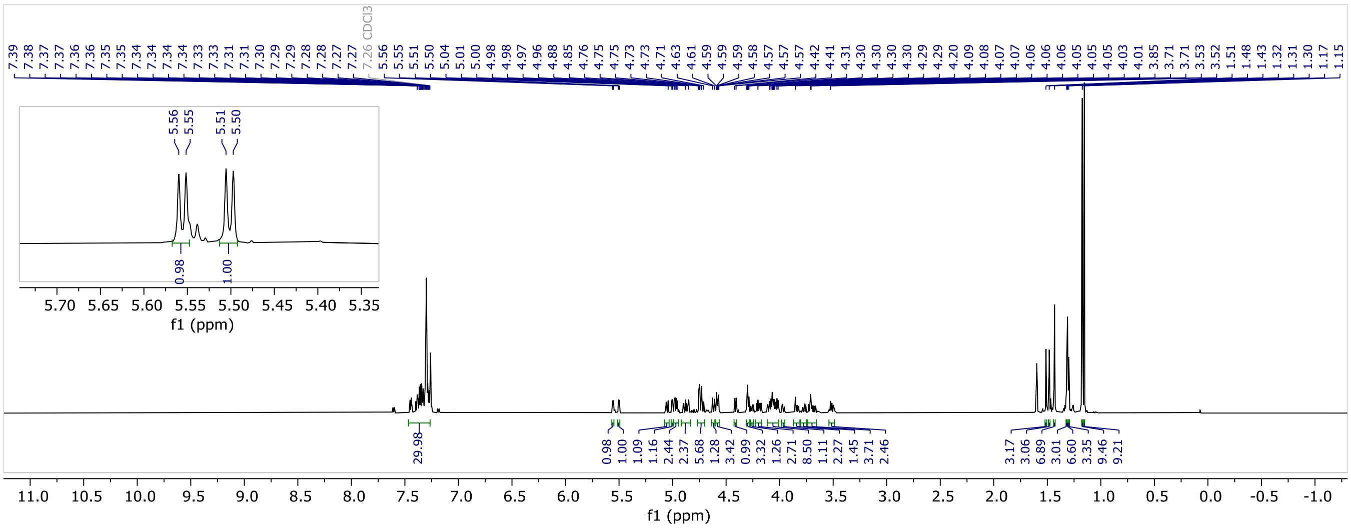

^{13}C NMR (151 MHz, $CDCl_3$) of **P1-SL101**:

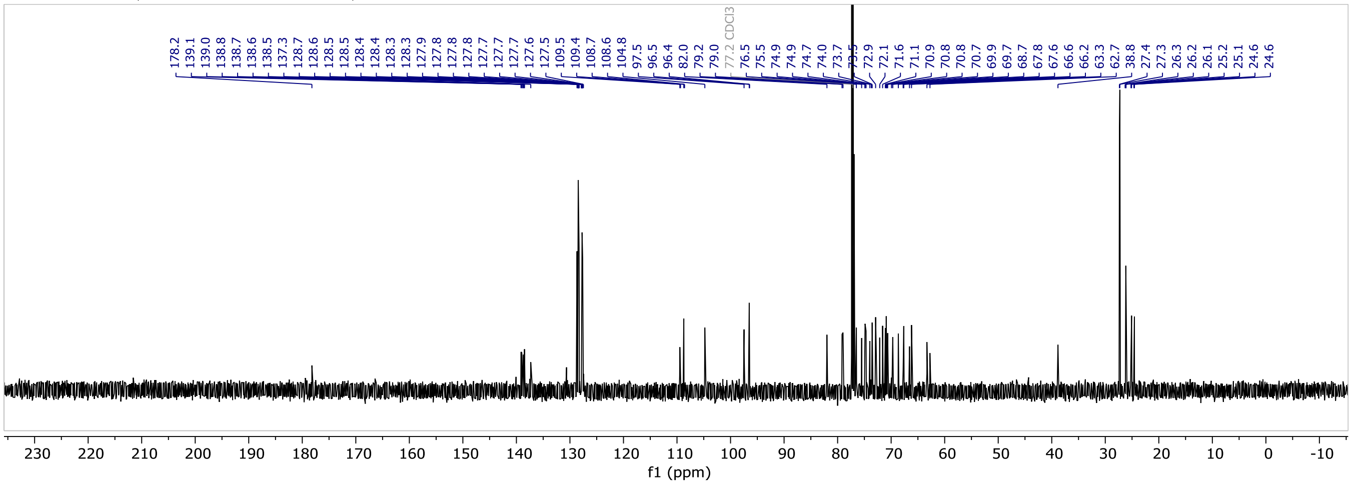

Coupled ^{13}C,^{1}H HSQC of **P1-SL101**:

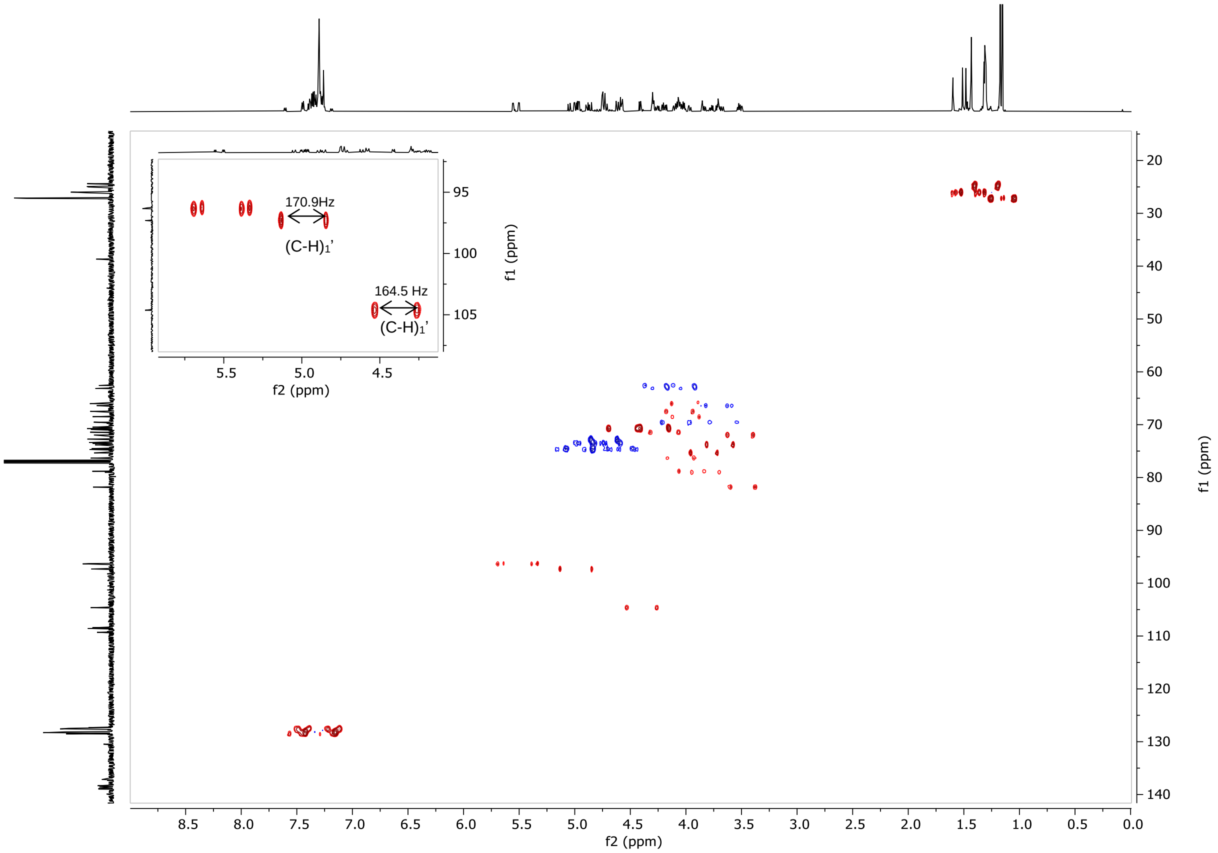

1,2:3,4-Bis-*O*-(1-methylethylidene)-6-*O*-[2,3-bis-*O*-benzyl-4,6-bis-(2,2-dimethylpropanoate)-α/β-D-galactopyranosyl]-α-D-galactopyranoside (4,6Piv-A1)

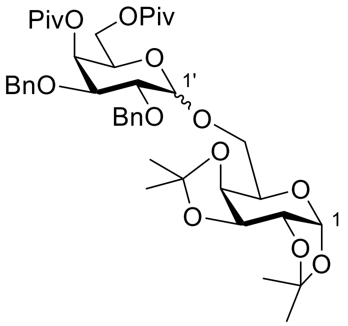

The title compound was prepared according to general procedure for glycosylations. Product **4,6Piv-A1** (19 mg, 24 µmol, 69%, **α/β 80:20**) was obtained as a colorless oil after purification using **Method-2b** (t_R (α) = 7.6 min, t_R (β) = 8.6 min).

Data of the major isomer (α):

^{1}H NMR (600 MHz, $CDCl_3$) δ 7.35 – 7.26 (m, 10H), 5.56 (dd, *J* = 3.5, 1.4 Hz, 1H), 5.51 (d, *J* = 5.0 Hz, 1H, **H$_1$**), 4.97 (d, *J* = 3.5 Hz, 1H, **H$_{1'}$**), 4.80 – 4.67 (m, 3H), 4.59 (ddd, *J* = 7.7, 5.3, 2.4 Hz, 1H), 4.54 (d, *J* = 10.8 Hz, 1H), 4.34 – 4.26 (m, 3H), 4.08 (dd, *J* = 11.0, 7.1 Hz, 1H), 4.05 – 3.97 (m, 3H), 3.82 – 3.72 (m, 3H), 1.52 (s, 3H), 1.44 (s, 3H), 1.33 – 1.32 (m, 6H), 1.21 (s, 9H), 1.15 (s, 9H) ppm.

^{13}C NMR (151 MHz, $CDCl_3$) δ 178.1, 176.9, 138.5, 138.4, 128.4, 128.3, 128.0, 128.0, 127.8, 127.5, 109.4, 108.7, 98.1 (**C$_{1'}$**), 96.4 (**C$_1$**), 76.1, 74.9, 73.2, 72.0, 71.1, 70.8, 70.7, 67.3, 67.1, 67.0, 66.5, 62.2, 39.2, 38.9, 27.3, 27.3, 26.3, 26.2, 25.1, 24.7 ppm.

HRMS (QToF): Calcd for $C_{42}H_{58}O_{13}Na$ $[M + Na]^+$ 793.3796; found 793.3786.

NP-HPLC of **P1-SL103** (ELSD trace, t_R (α) = 7.6 min, t_R (β) = 8.6 min):

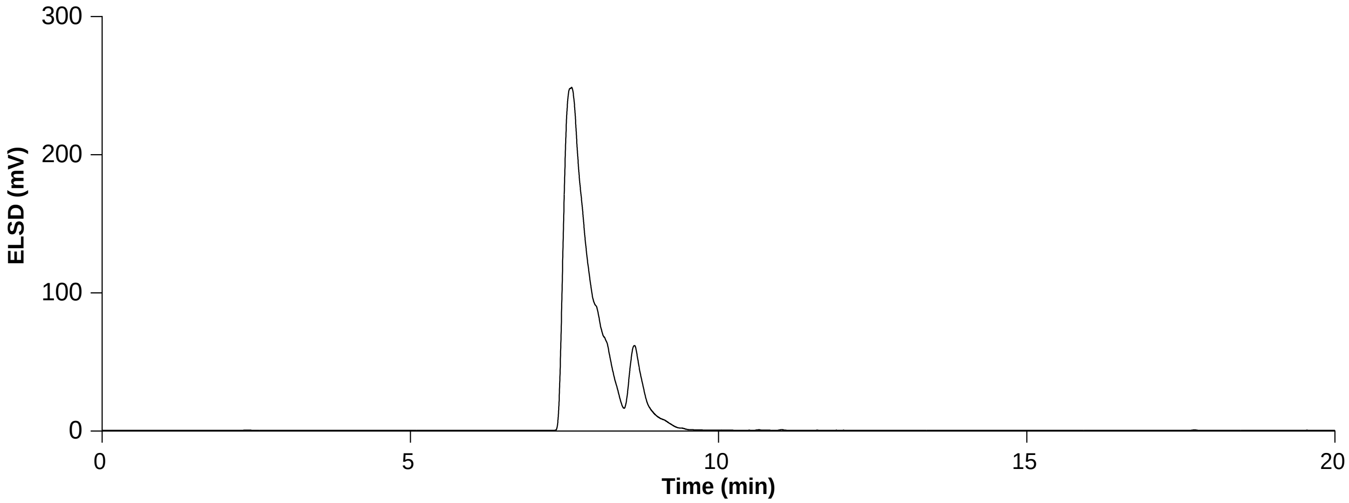

^{1}H NMR (600 MHz, $CDCl_3$) of **4,6Piv-A1**:

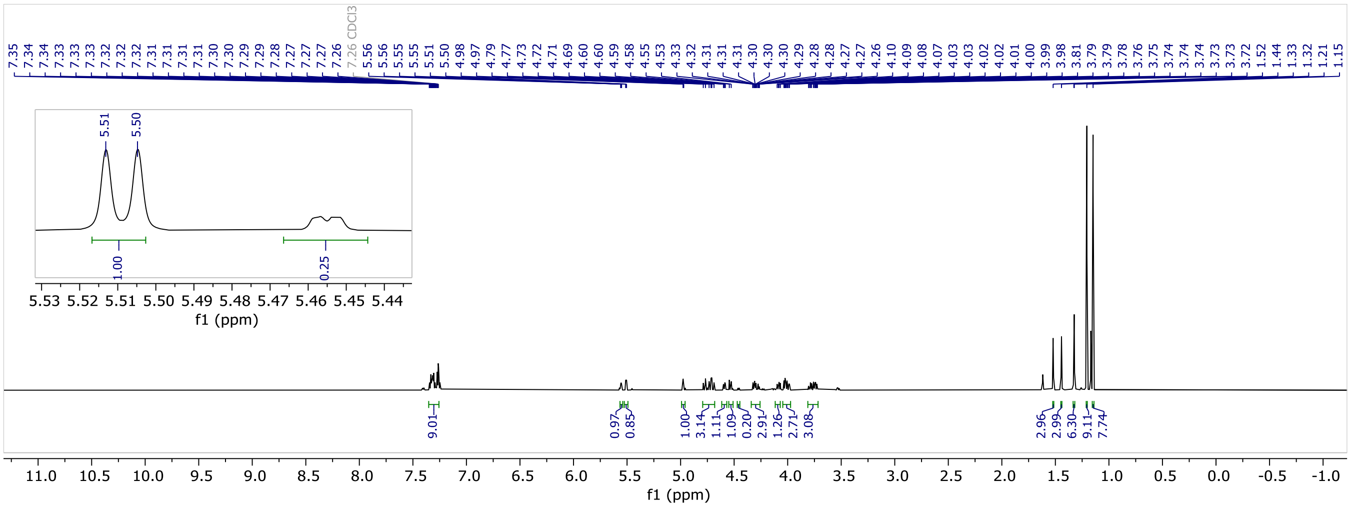

^{13}C NMR (151 MHz, $CDCl_3$) of **4,6Piv-A1**:

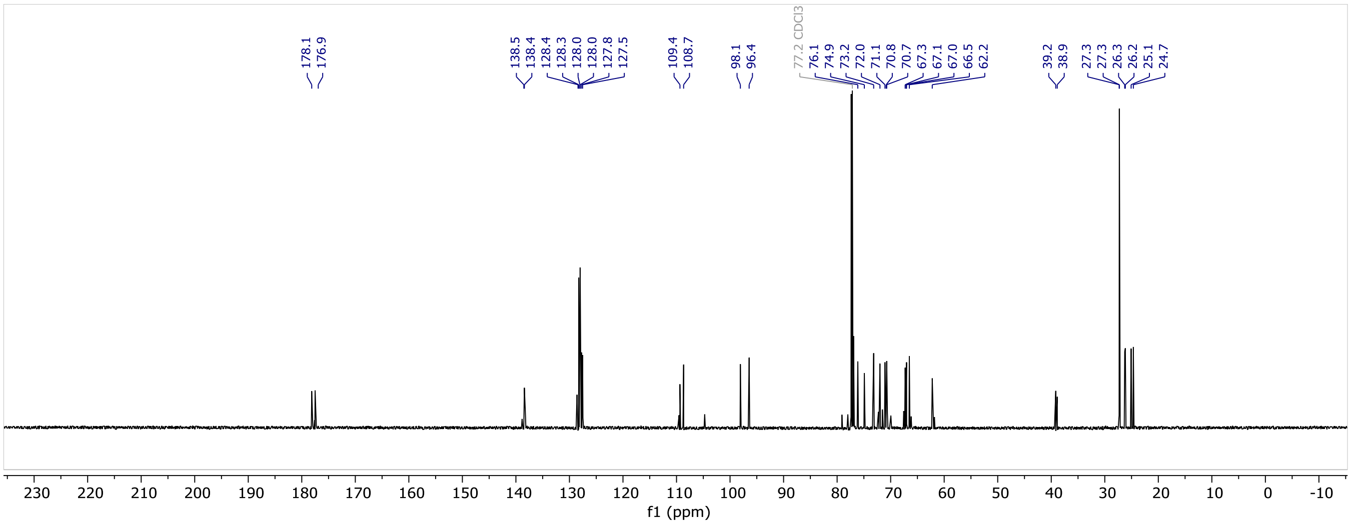

Coupled ^{13}C,^{1}H HSQC of **4,6Piv-A1**:

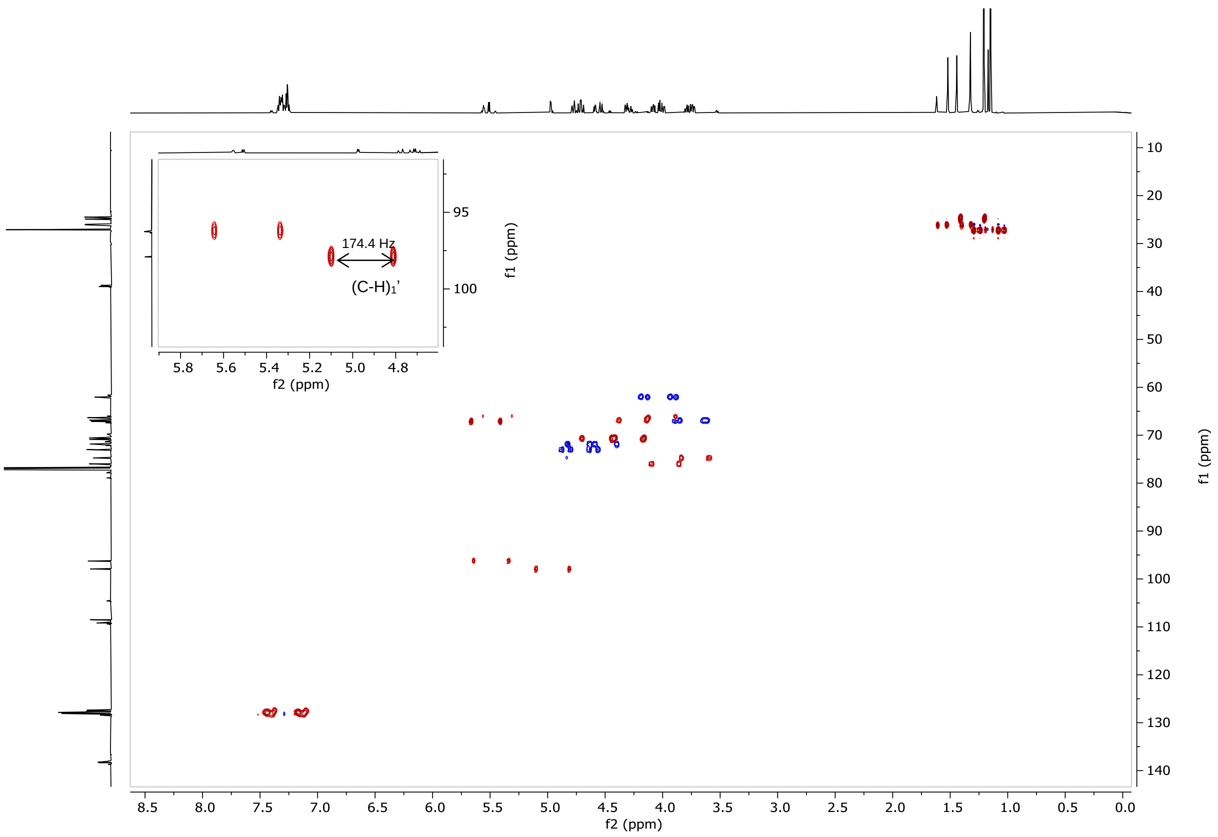

1,2:3,4-Bis-*O*-(1-methylethylidene)-6-*O*-[2,3,6-tris-*O*-benzyl-4-(2,2-dimethylpropanoate)-α/β-D-galactopyranosyl]-α-D-galactopyranoside (4Piv-A1)

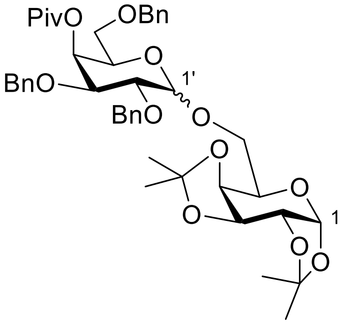

The title compound was prepared according to general procedure for glycosylations. Product **4Piv-A1** (22 mg, 28 μmol, 82%, **α/β 96:4**) was obtained as a colorless oil after purification using **Method-2b** (t_R (α) = 7.9 min).

Data of the major isomer (α):

^{1}H NMR (600 MHz, $CDCl_3$) δ 7.35 – 7.24 (m, 15H), 5.63 (dd, *J* = 3.4, 1.3 Hz, 1H), 5.51 (d, *J* = 5.1 Hz, 1H, **H_1**), 4.98 (d, *J* = 3.7 Hz, 1H, **$H_{1'}$**), 4.79 – 4.67 (m, 3H), 4.58 (dd, *J* = 7.9, 2.4 Hz, 1H), 4.52 (t, *J* = 10.7 Hz, 2H), 4.48 – 4.43 (m, 1H), 4.34 (dd, *J* = 7.9, 1.9 Hz, 1H), 4.30 (dd, *J* = 5.0, 2.4 Hz, 1H), 4.23 (td, *J* = 6.5, 1.3 Hz, 1H), 4.04 (td, *J* = 6.7, 1.9 Hz, 1H), 3.98 (dd, *J* = 10.0, 3.3 Hz, 1H), 3.85 – 3.80 (m, 1H), 3.80 – 3.70 (m, 2H), 3.46 (ddd, *J* = 42.0, 9.6, 6.5 Hz, 2H), 1.53 (s, 3H), 1.43 (s, 3H), 1.33 (s, 3H), 1.32 (s, 3H), 1.12 (s, 9H) ppm.

^{13}C NMR (151 MHz, $CDCl_3$) δ 177.5, 138.7, 138.6, 138.0, 128.5, 128.4, 128.2, 128.1, 128.0, 128.0, 127.8, 127.7, 127.5, 109.3, 108.7, 98.1 (**$C_{1'}$**), 96.4 (**C_1**), 76.5, 75.1, 73.6, 73.1, 71.8, 71.0, 70.8, 70.8, 68.5, 67.9, 67.6, 66.8, 66.5, 66.2, 39.1, 29.8, 27.3, 26.3, 26.2, 25.1, 24.8 ppm.

HRMS (QToF): Calcd for $C_{44}H_{56}O_{12}Na$ $[M + Na]^+$ 799.3664; found 799.3693.

NP-HPLC of **4Piv-A1** (ELSD trace, t_R (α) = 7.9 min):

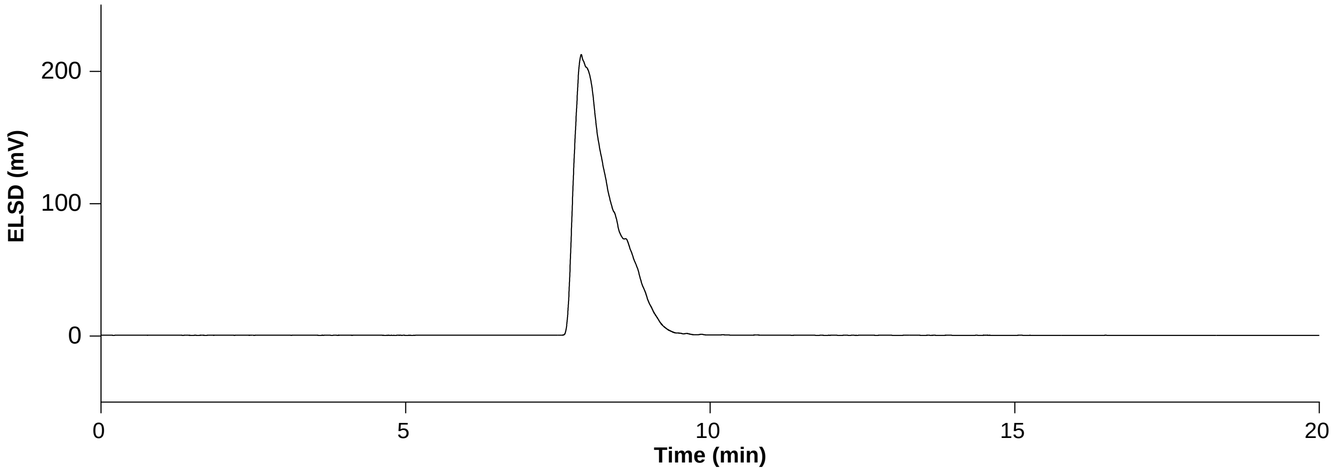

^{1}H NMR (600 MHz, $CDCl_3$) of **4Piv-A1**:

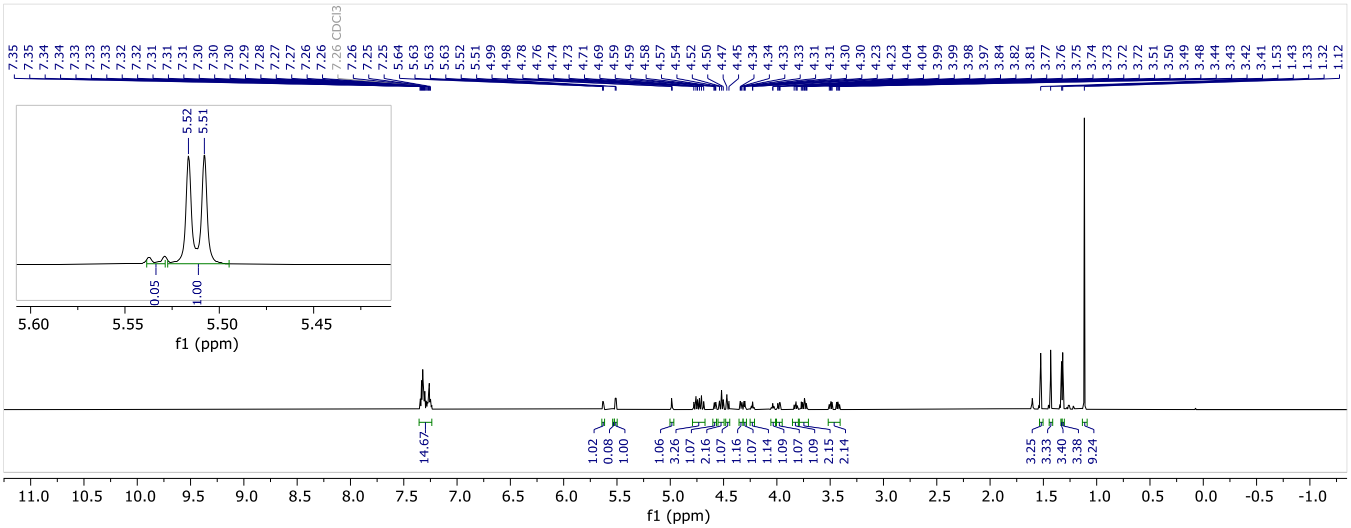

^{13}C NMR (151 MHz, $CDCl_3$) of **4Piv-A1**:

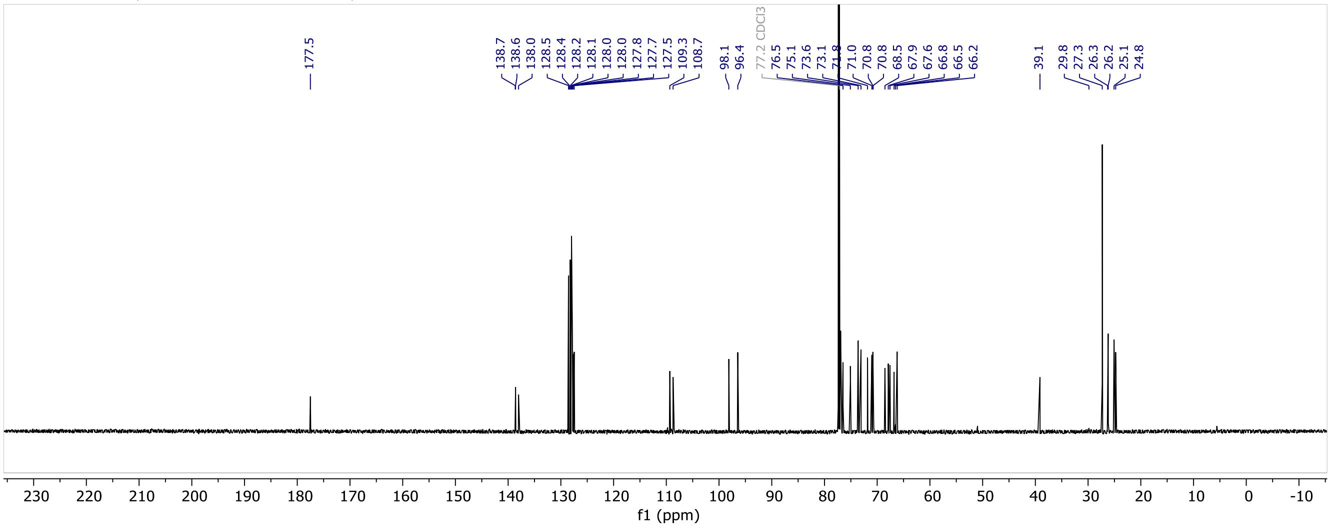

Coupled $^{13}C,^{1}H$ HSQC of **4Piv-A1**:

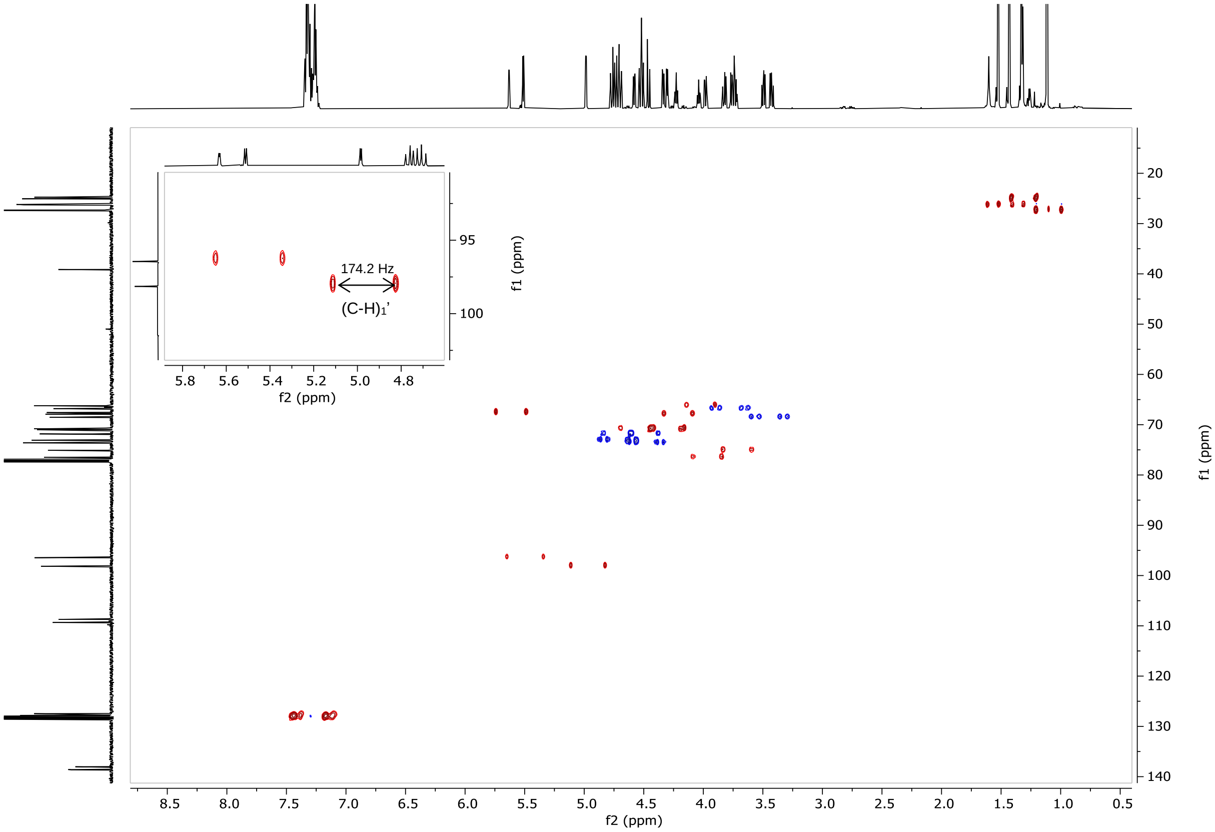

1,2:3,4-Bis-*O*-(1-methylethylidene)-6-*O*-[2,3,6-tris-*O*-benzyl-4-(trifluoroacetate)-α/β-D-galactopyranosyl]-α-D-galactopyranoside (4TFA-A1)

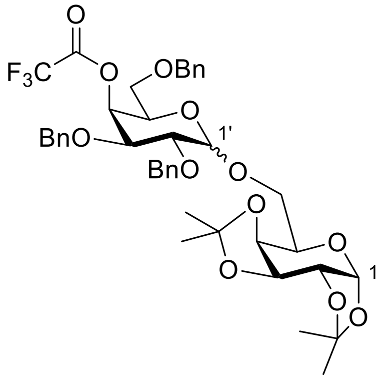

The title compound was prepared according to general procedure for glycosylations. Product **4TFA-A1** (18 mg, 23 μmol, 67%, **α/β 85:15**) was obtained as a colorless oil after purification using **Method-2b** (t_R (α) = 6.0 min, t_R (β) = 9.1 min).

Data of the major isomer (α):

^{1}H NMR (600 MHz, $CDCl_3$) δ 7.37 – 7.26 (m, 15H), 5.74 (dd, *J* = 3.4, 1.3 Hz, 1H), 5.49 (d, *J* = 5.0 Hz, 1H, **H_1**), 4.94 (d, *J* = 3.7 Hz, 1H, **$H_{1'}$**), 4.75 (dt, *J* = 11.6, 3.4 Hz, 2H), 4.67 (d, *J* = 12.0 Hz, 1H), 4.61 – 4.54 (m, 2H), 4.53 – 4.43 (m, 2H), 4.33 – 4.25 (m, 3H), 4.06 – 3.98 (m, 2H), 3.80 – 3.68 (m, 3H), 3.54 (dd, *J* = 9.3, 5.6 Hz, 1H), 3.41 (dd, *J* = 9.2, 8.3 Hz, 1H), 1.51 (s, 3H), 1.43 (s, 3H), 1.33 (s, 3H), 1.30 (s, 3H) ppm.

^{13}C NMR (151 MHz, $CDCl_3$) δ 138.4, 138.0, 137.6, 128.6, 128.4, 128.1, 128.0, 127.9, 127.8, 127.8, 109.5, 108.8, 98.0 (**$C_{1'}$**), 96.5 (**C_1**), 75.7, 75.2, 73.8, 73.4, 72.9, 72.6, 71.1, 70.8, 70.7, 67.4, 67.3, 66.6, 66.4, 29.9, 26.3, 26.2, 25.1, 24.7 ppm.

^{19}F NMR (564 MHz, $CDCl_3$) δ -74.85 (s) ppm.

HRMS (QToF): Calcd for $C_{41}H_{47}F_3O_{12}Na$ $[M + Na]^+$ 811.2912; found 811.2944.

NP-HPLC of **4TFA-A1** (ELSD trace, t_R (α) = 6.0 min, t_R (β) = 9.1 min):

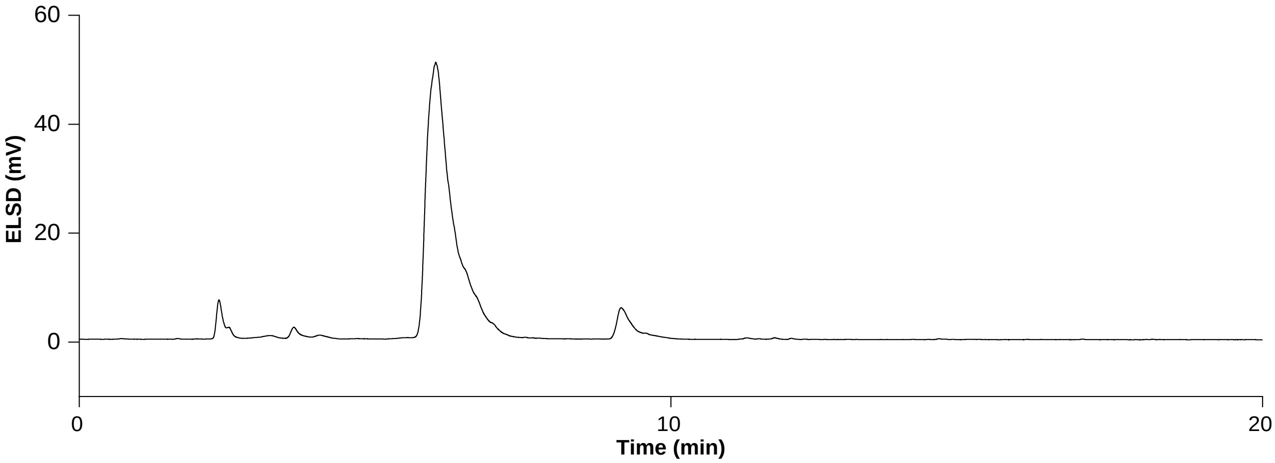

^{1}H NMR (600 MHz, $CDCl_3$) of **4TFA-A1**:

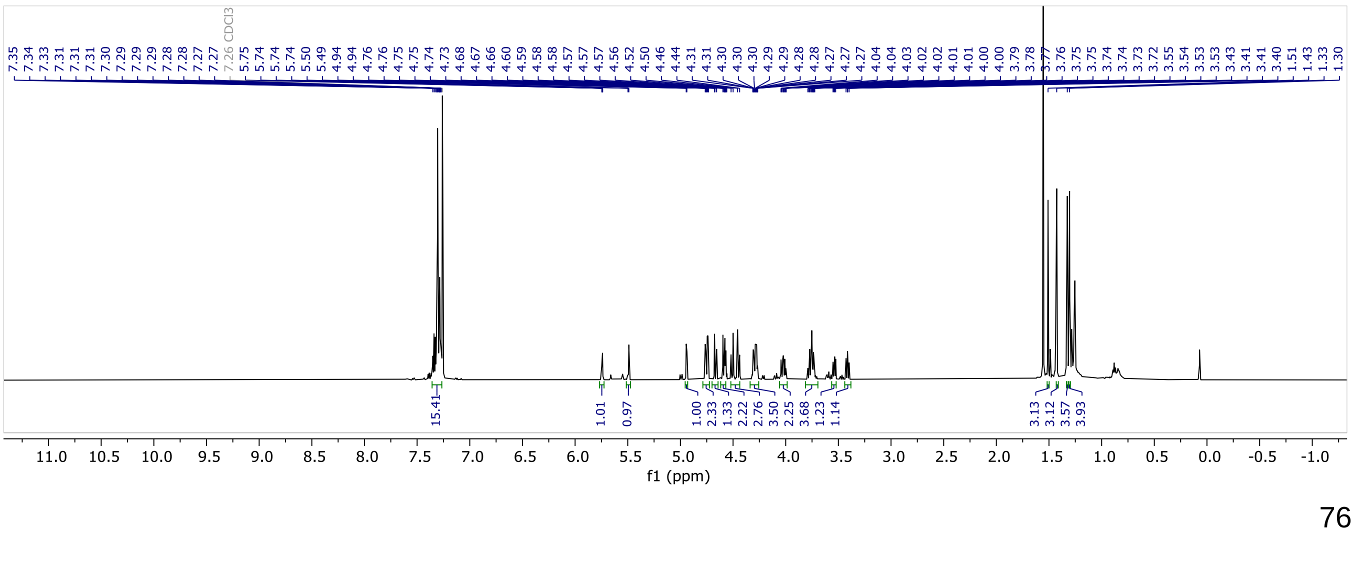

^{13}C NMR (151 MHz, $CDCl_3$) of **4TFA-A1**:

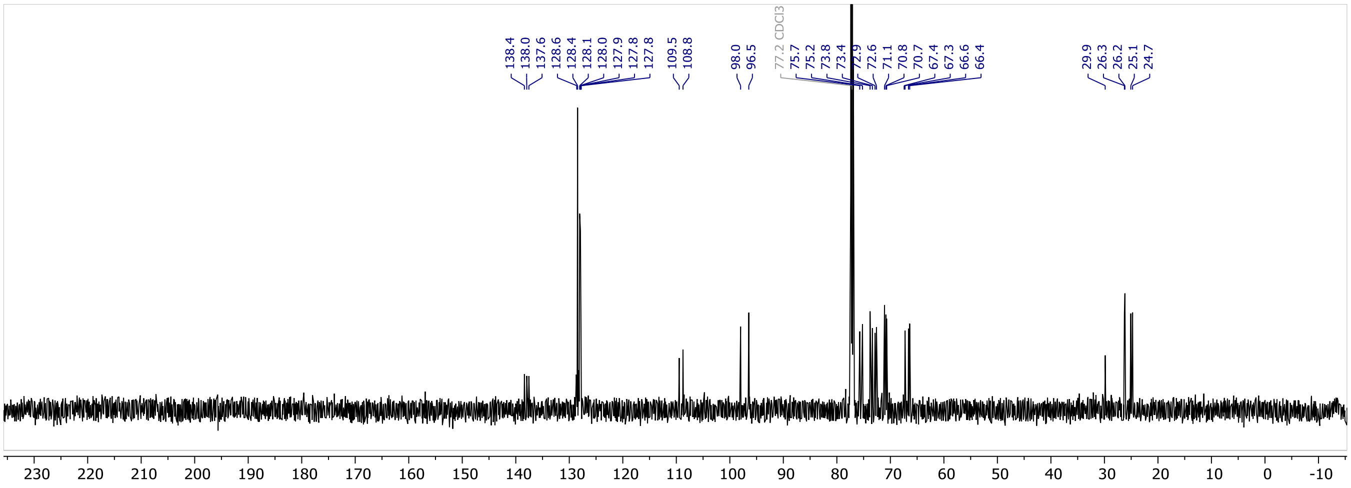

^{19}F NMR (376 MHz, $CDCl_3$) of **4TFA-A1**:

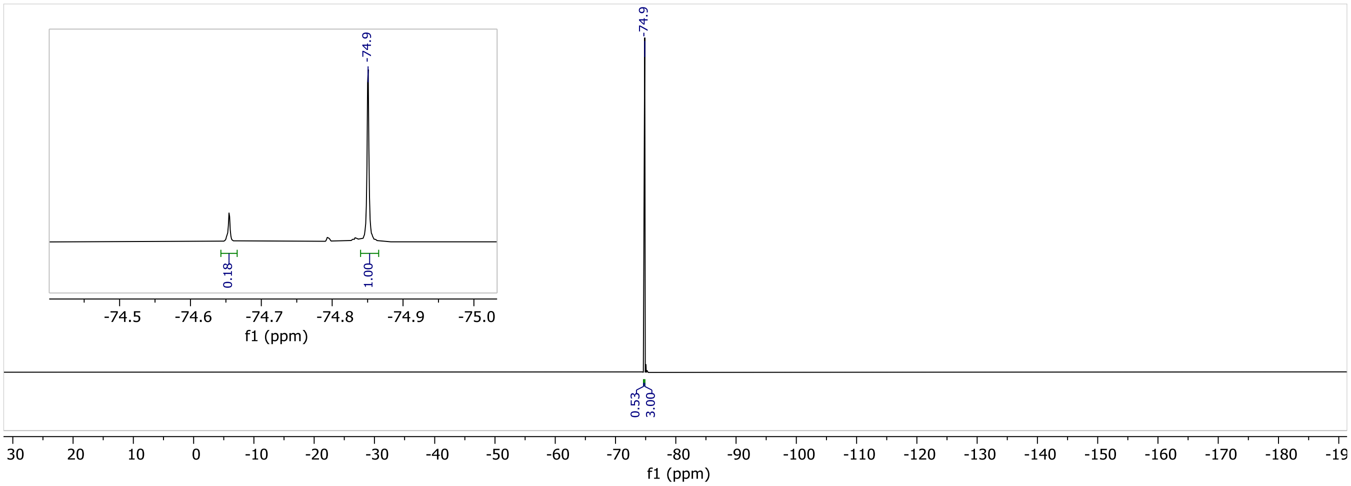

Coupled ^{13}C,^{1}H HSQC of **4TFA-A1**:

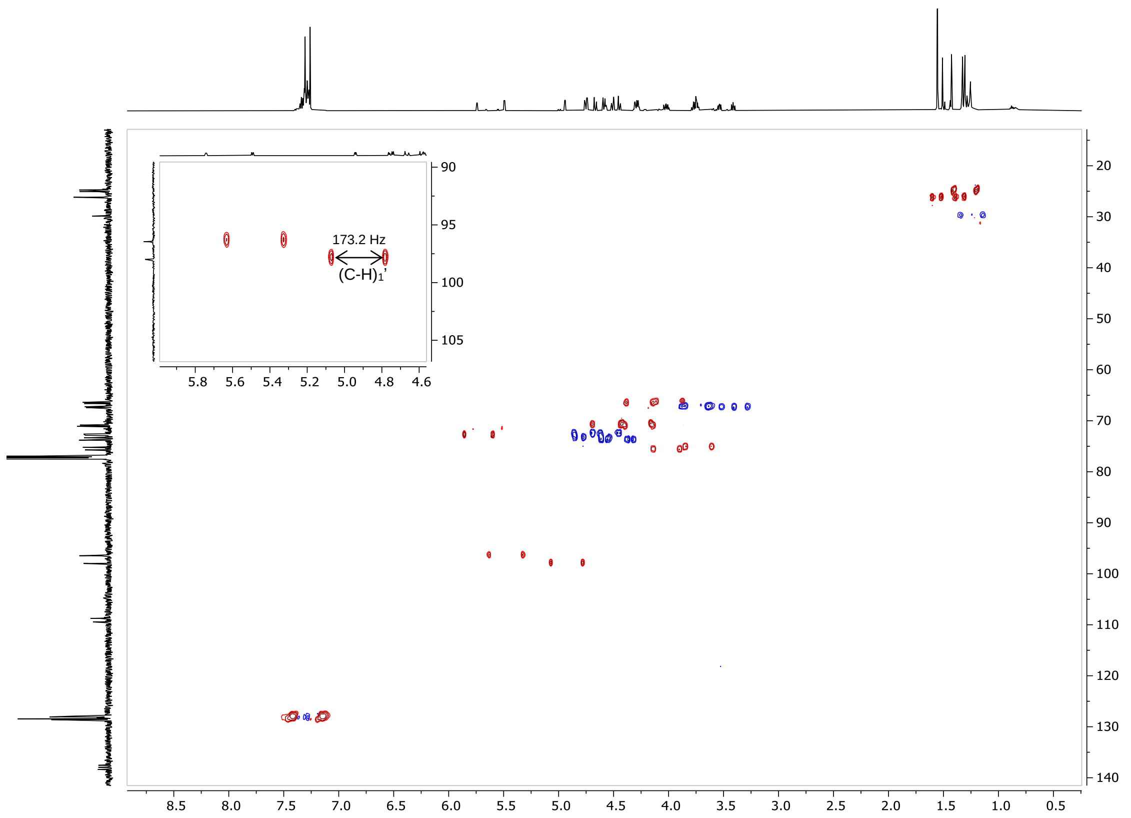

1,2:3,4-Bis-*O*-(1-methylethylidene)-6-*O*-[2,3,4-tris-*O*-benzyl-6-(trifluoroacetate)-α/β-D-galactopyranosyl]-α-D-galactopyranoside (6TFA-A1)

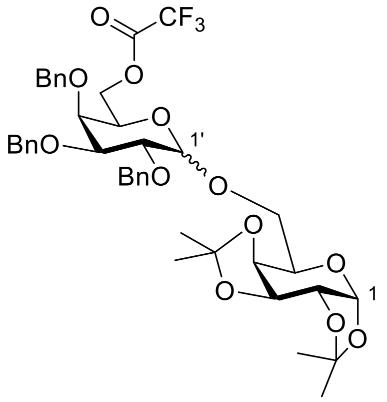

The title compound was prepared according to general procedure for glycosylations. Product **6TFA-A1** (15 mg, 19 µmol, 56%, **α/β 53:47**) was obtained as a colorless oil after purification using **Method-2b** (t_R (α) = 8.3 min, t_R (β) = 9.0 min).

Data of the anomeric mixture:

^{1}H NMR (600 MHz, $CDCl_3$) δ 7.46 – 6.98 (m, 30H), 5.56 (d, *J* = 5.0 Hz, 1H. **H_1(β)**), 5.46 (d, *J* = 5.0 Hz, 1H, **H_1(α)**), 5.05 (d, *J* = 11.0 Hz, 1H, **H_1'(α)**), 4.98 (dd, *J* = 11.6, 7.3 Hz, 2H), 4.93 (d, *J* = 3.6 Hz, 1H), 4.88 (dd, *J* = 14.2, 11.7 Hz, 2H), 4.81 – 4.71 (m, 5H), 4.63 – 4.55 (m, 4H), 4.51 – 4.42 (m, 3H, **H_1'(β)**), 4.29 (ddd, *J* = 15.8, 5.0, 2.4 Hz, 2H), 4.24 – 4.19 (m, 2H), 4.18 – 4.03 (m, 5H), 3.98 (dd, *J* = 12.7, 9.8 Hz, 2H), 3.88 – 3.82 (m, 2H), 3.78 – 3.66 (m, 4H), 3.61 (t, *J* = 6.2 Hz, 1H), 3.54 (dd, *J* = 9.7, 2.9 Hz, 1H), 1.48 (s, 6H), 1.44 (s, 3H), 1.42 (s, 3H), 1.31 (s, 3H), 1.31 (s, 3H), 1.30 (s, 3H), 1.30 (s, 4H) ppm.

^{13}C NMR (151 MHz, $CDCl_3$) δ 139.0, 138.8, 138.6, 138.5, 138.2, 138.0, 128.7, 128.6, 128.6, 128.5, 128.3, 128.1, 128.1, 128.0, 127.9, 127.8, 127.8, 127.8, 127.7, 127.6, 109.5, 109.5, 108.8, 108.6, 104.8 (**C_1'(β)**), 97.7 (**C_1'(α)**), 96.5 (**C_1(β)**), 96.4 (**C_1(α)**), 81.7, 79.0, 78.8, 76.4, 74.9, 74.7, 74.5, 74.5, 73.9, 73.8, 73.3, 73.0, 71.6, 71.5, 71.3, 70.9, 70.9, 70.6, 69.8, 67.8, 67.7, 67.6, 66.9, 66.5, 66.4, 26.2, 26.2, 26.1, 26.0, 25.2, 25.0, 24.6, 24.5 ppm.

^{19}F NMR (564 MHz, $CDCl_3$) δ -74.9 (s), -74.9 (s) ppm.

HRMS (QToF): Calcd for $C_{41}H_{47}F_3O_{12}Na$ $[M + Na]^+$ 811.2939; found 811.2912.

NP-HPLC of **6TFA-A1** (ELSD trace, t_R (α) = 8.3 min, t_R (β) = 9.0 min):

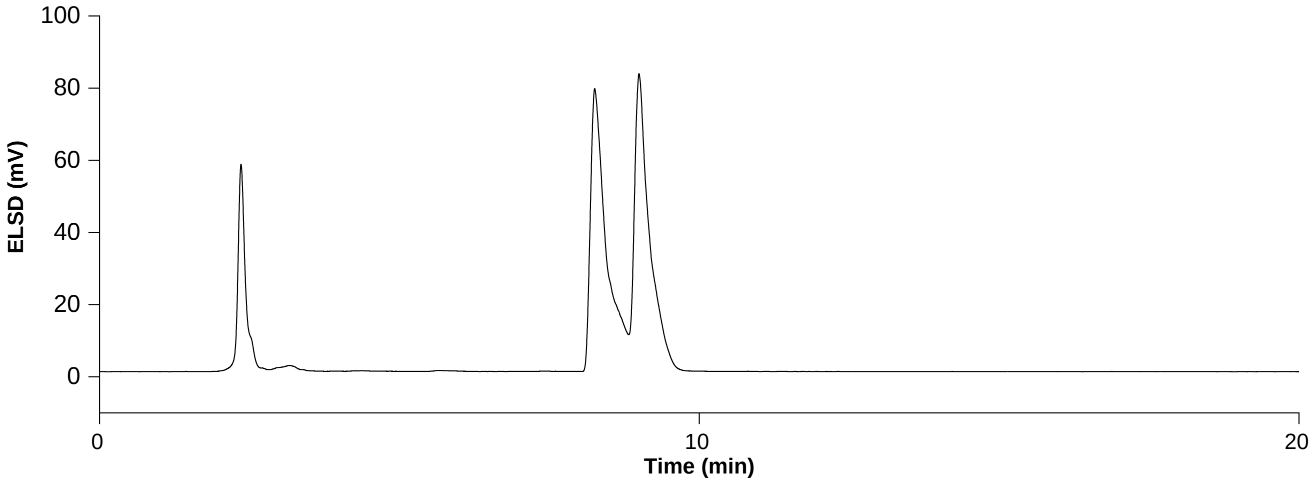

^{1}H NMR (600 MHz, $CDCl_3$) of **6TFA-A1**:

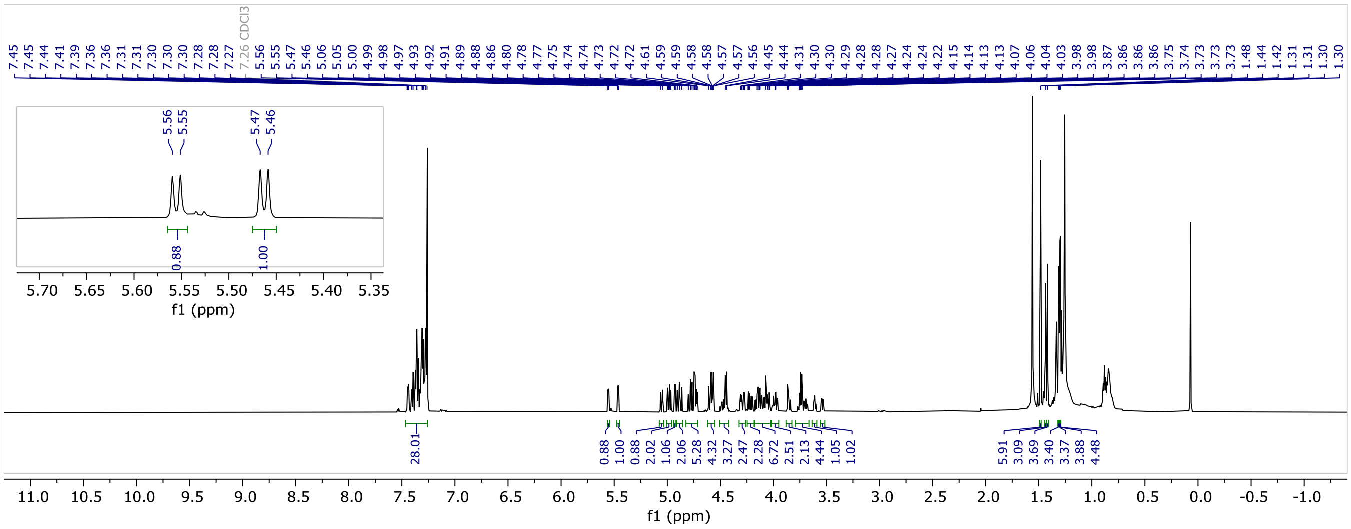

^{19}F NMR (376 MHz, $CDCl_3$) of **6TFA-A1**:

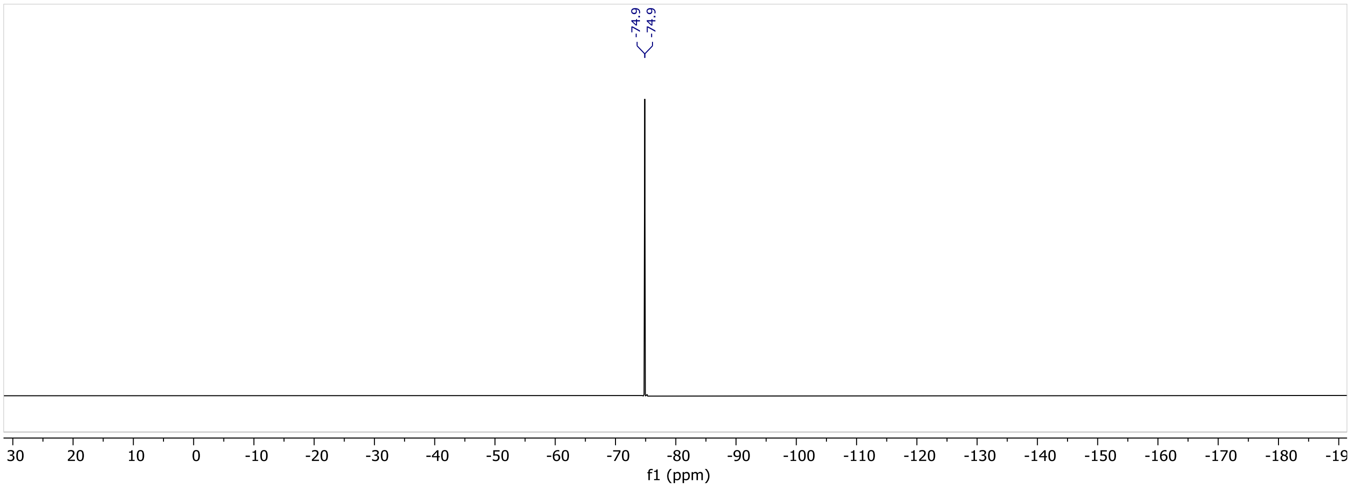

^{13}C NMR (151 MHz, $CDCl_3$) of **6TFA-A1**:

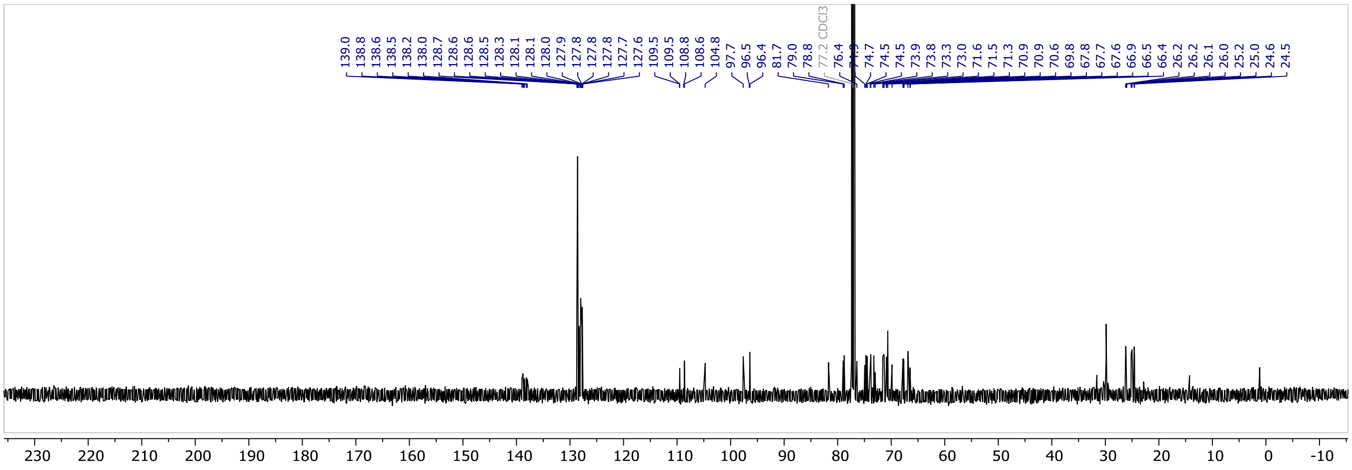

Coupled ^{13}C,^{1}H HSQC of **6TFA-A1**:

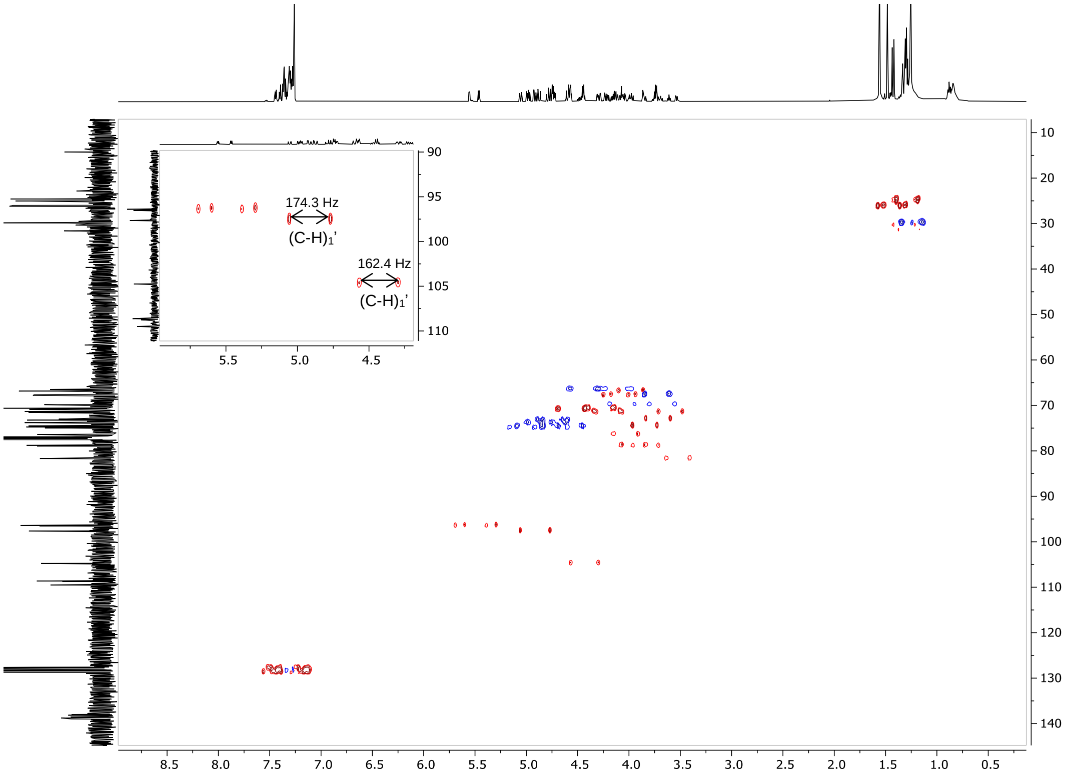

2,2,2-Trifluoroethyl 2,3,4-tris-*O*-benzyl-6-(2,2-dimethylpropanoate)-α/β-D-galactopyranoside (6Piv-A3)

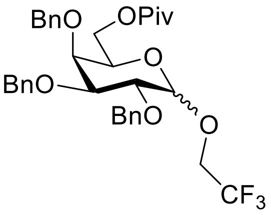

The title compound was prepared according to general procedure for glycosylations. Product **6Piv-A3** (18 mg, 29 µmol, 85%, **α/β 82:18**) was obtained as a colorless oil after purification using **Method-2b** (t_R (α) = 6.7 min).

Data of the major isomer (α):

^{1}H NMR (600 MHz, $CDCl_3$) δ 7.42 – 7.27 (m, 15H), 4.99 (d, *J* = 11.2 Hz, 1H), 4.93 – 4.81 (m, 3H), 4.75 (d, *J* = 11.6 Hz, 1H, **H_1**), 4.66 (d, *J* = 12.2 Hz, 1H), 4.59 (d, *J* = 11.3 Hz, 1H), 4.17 (dd, *J* = 11.4, 7.5 Hz, 1H), 4.11 – 4.01 (m, 2H), 3.98 – 3.81 (m, 5H), 1.17 (s, 9H) ppm.

^{13}C NMR (151 MHz, $CDCl_3$) δ 178.2, 138.7, 138.5, 138.2, 128.6, 128.5, 128.4, 128.1, 128.0, 127.8, 127.7, 98.2 (**C_1**), 78.6, 76.2, 75.3, 74.9, 73.8, 73.6, 69.6, 63.7, 38.8, 27.2 ppm.

^{19}F NMR (564 MHz, $CDCl_3$) δ -73.61 (t, J = 8.7 Hz) ppm.

HRMS (QToF): Calcd for $C_{34}H_{39}F_3O_7Na$ $[M + Na]^+$ 639.2540; 639.2535.

NP-HPLC of **6Piv-A3** (ELSD trace, t_R (α) = 6.7 min):

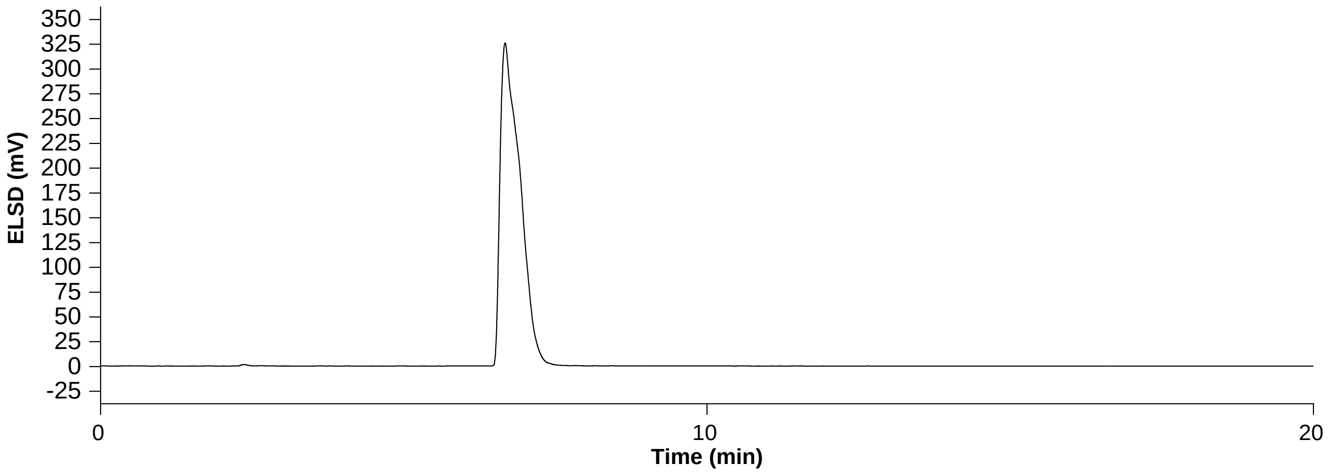

^{1}H NMR (600 MHz, $CDCl_3$) of **6Piv-A3**:

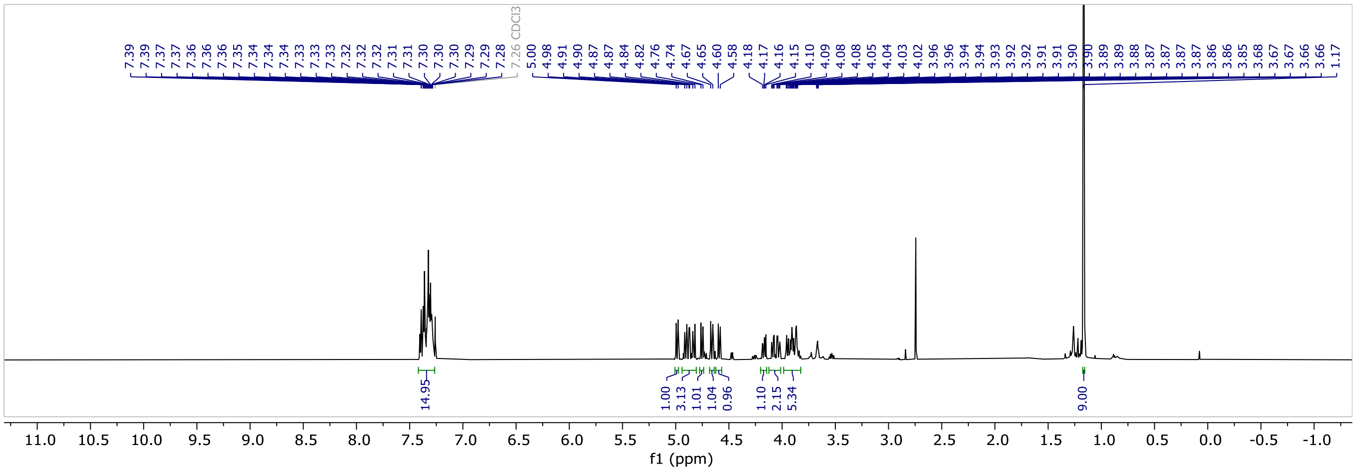

^{19}F NMR (376 MHz, $CDCl_3$) of **6Piv-A3**:

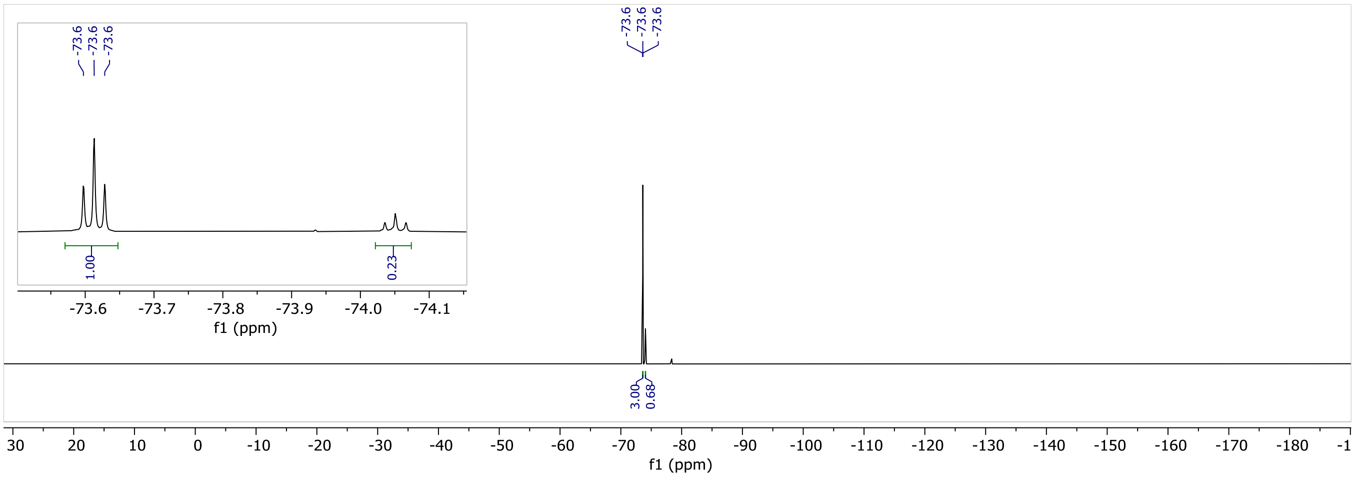

^{13}C NMR (151 MHz, $CDCl_3$) of **6Piv-A3**:

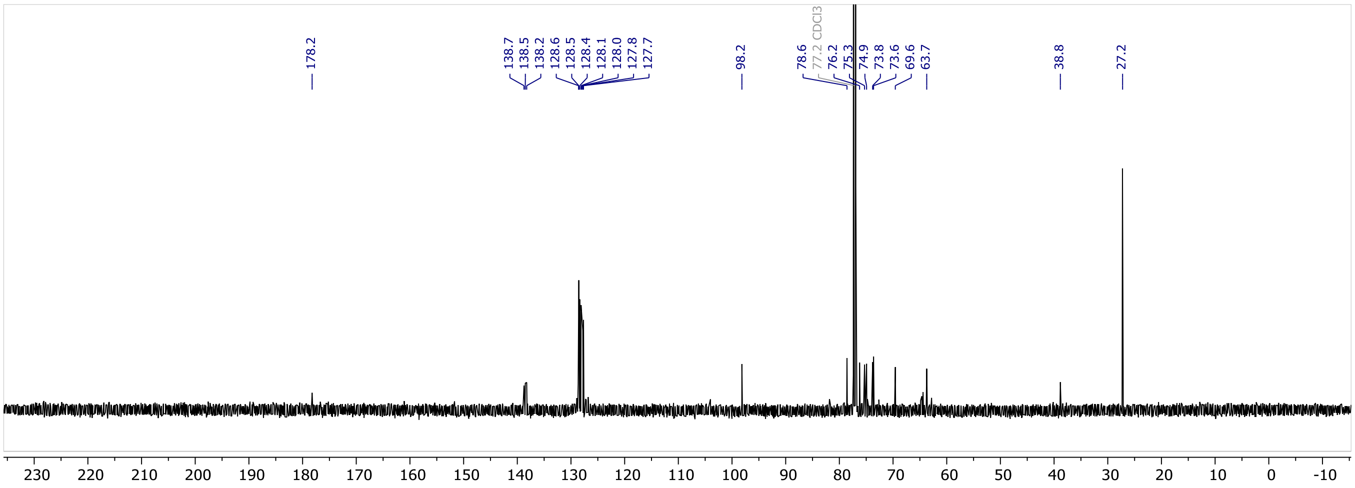

Coupled $^{13}C,^{1}H$ HSQC of **6Piv-A3**:

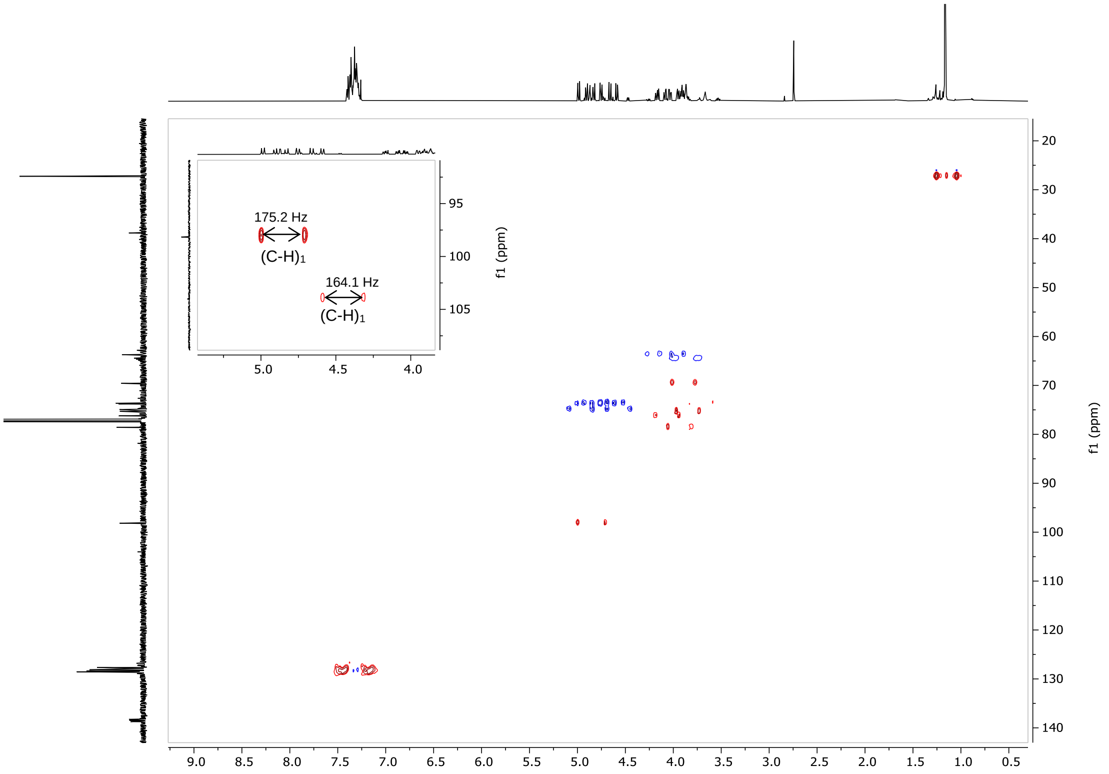

2,2,2-Trifluoroethyl 2,3-bis-*O*-benzyl-4,6-bis-(2,2-dimethylpropanoate)-α/β-D-galactopyranoside (4,6Piv-A3)

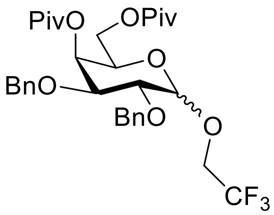

The title compound was prepared according to general procedure for glycosylations. Product **4,6Piv-A3** (13 mg, 21 μmol, 61%, **α/β 96:4**) was obtained as a colorless oil after purification using **Method-2b** (t_R (α) = 4.5 min).

Data of the major isomer (α):

^{1}**H NMR** (600 MHz, $CDCl_3$) δ 7.33 – 7.27 (m, 10H), 5.56 (dd, *J* = 3.5, 1.4 Hz, 1H), 4.88 (s, 1H, **H**$_1$), 4.82 (d, *J* = 12.2 Hz, 1H), 4.72 (d, *J* = 10.8 Hz, 1H), 4.64 (d, *J* = 12.2 Hz, 1H), 4.55 (d, *J* = 10.7 Hz, 1H), 4.16 (td, *J* =

6.5, 1.4 Hz, 1H), 4.10 – 4.02 (m, 2H), 3.99 (dd, *J* = 10.1, 3.4 Hz, 1H), 3.93 (q, *J* = 8.6 Hz, 2H), 3.76 (dd, *J* = 10.0, 3.7 Hz, 1H), 1.19 (s, 9H), 1.16 (s, 9H) ppm.

^{13}C NMR (151 MHz, $CDCl_3$) δ 178.1, 177.4, 138.2, 138.1, 128.5, 128.3, 128.2, 128.1, 128.0, 127.7, 98.6 (**C_1**), 75.8, 74.4, 73.7, 72.2, 68.0, 67.1, 62.5, 39.2, 38.9, 27.3, 27.2 ppm.

^{19}F NMR (564 MHz, $CDCl_3$) δ -73.63 (t, *J* = 8.7 Hz) ppm.

HRMS (QToF): Calcd for $C_{32}H_{41}F_3O_8Na$ $[M + Na]^+$ 633.2646; found 633.2651.

NP-HPLC of **4,6Piv-A3** (ELSD trace, t_R (α) = 4.5 min):

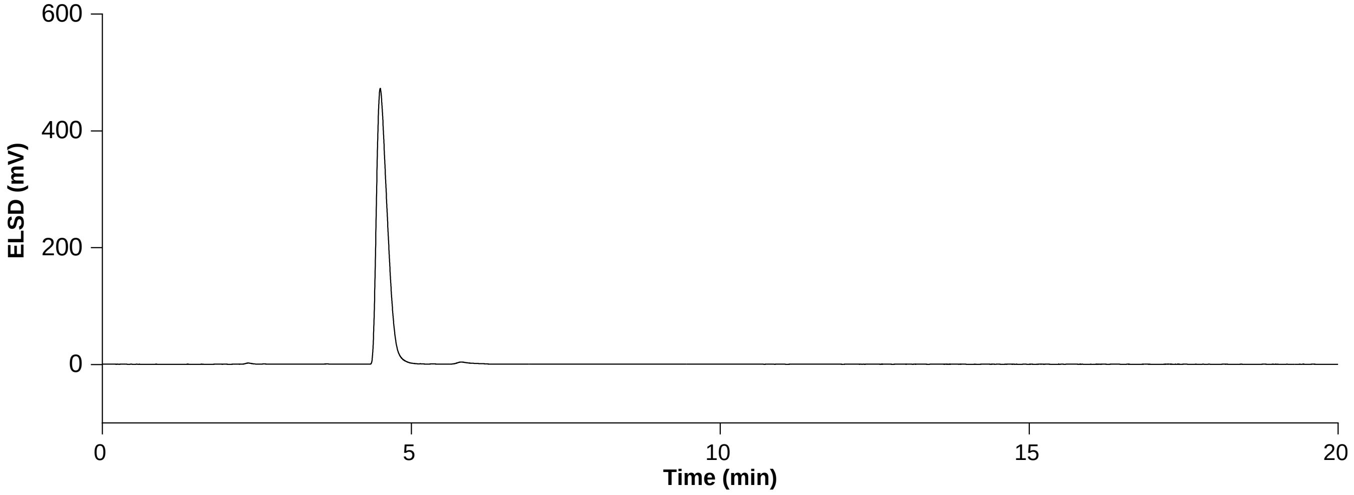

^{1}H NMR (600 MHz, $CDCl_3$) of **4,6Piv-A3**:

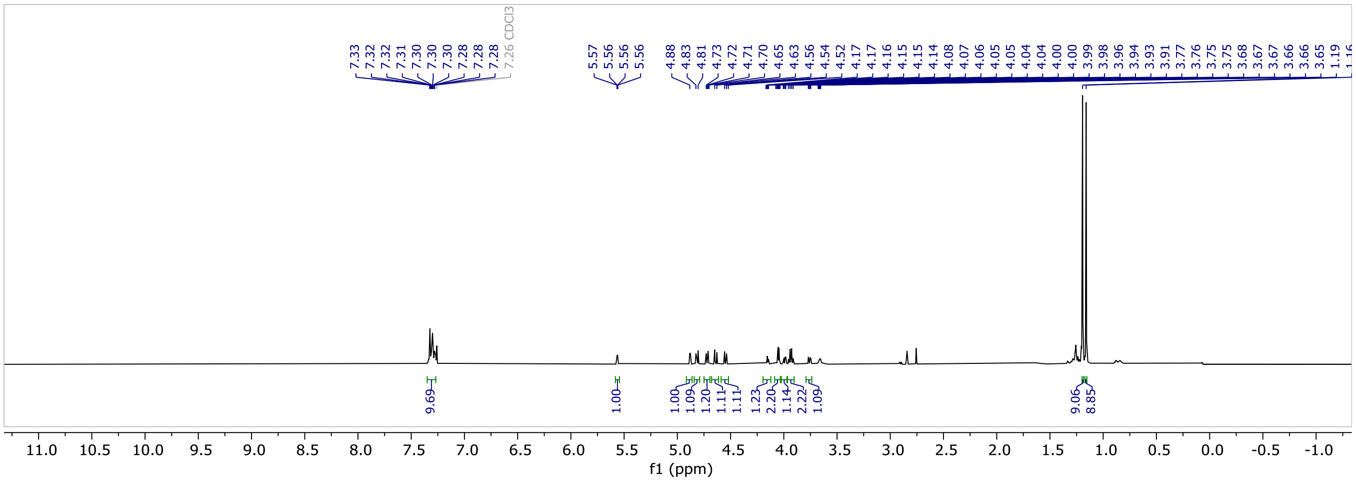

^{13}C NMR (151 MHz, $CDCl_3$) of **4,6Piv-A3**:

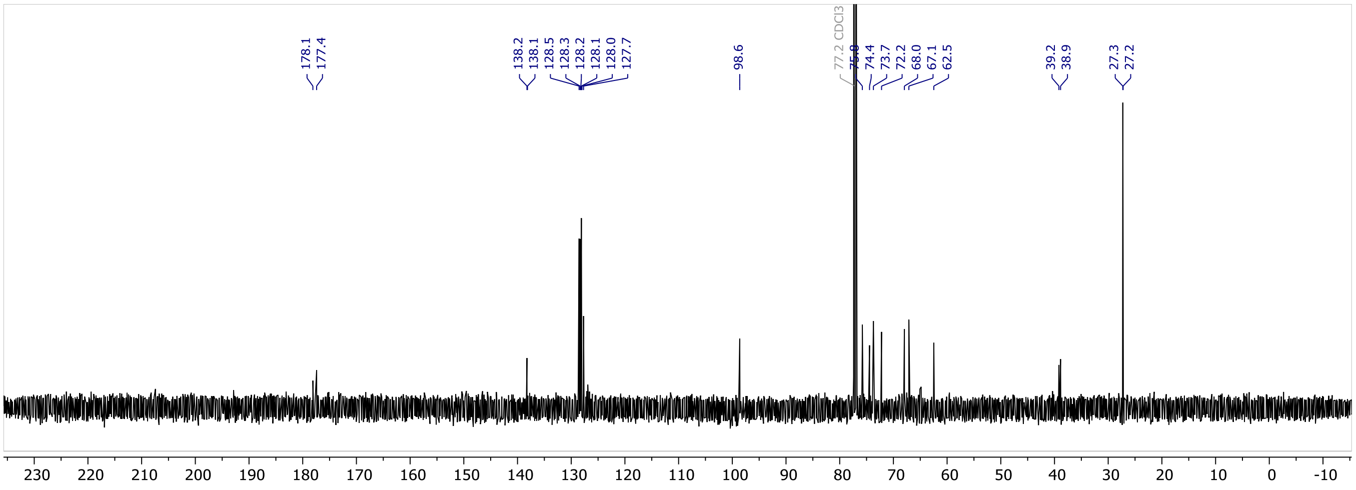

^{19}F NMR (376 MHz, $CDCl_3$) of **4,6Piv-A3**:

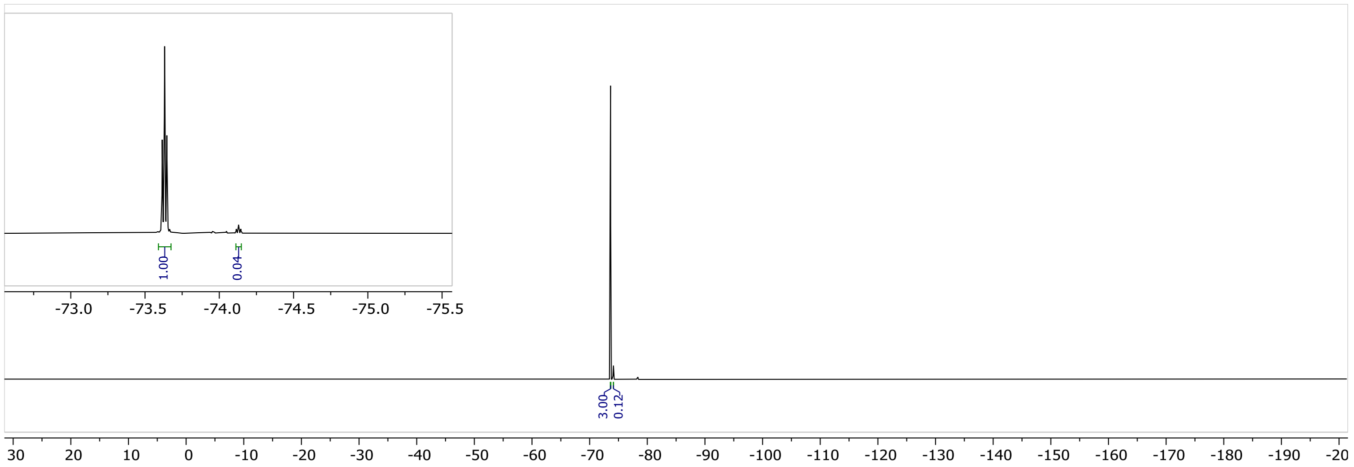

Coupled $^{13}C,^{1}H$ HSQC of **4,6Piv-A3**:

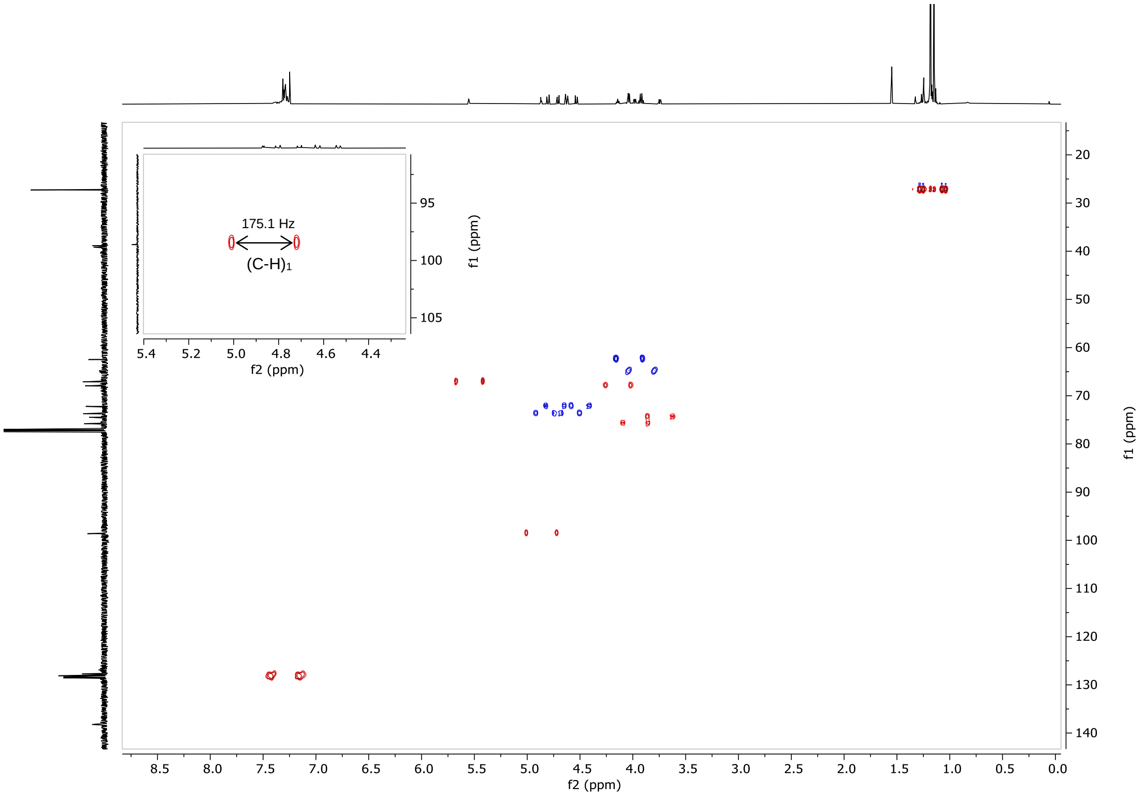

2,2,2-Trifluoroethyl 2,3,6-tris-*O*-benzyl-4-(2,2-dimethylpropanoate)-α/β-D-galactopyranoside (4Piv-A3)

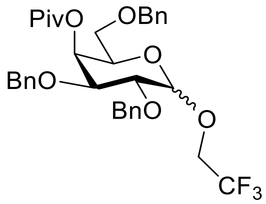

The title compound was prepared according to general procedure for glycosylations. Product **4Piv-A3** (15 mg, 24 μmol, 71%, **α/β 97:3**) was obtained as a colorless oil after purification using **Method-2b** (t_R (α) = 5.5 min).

Data of the major isomer (α):

^{1}H NMR (600 MHz, $CDCl_3$) δ 7.37 – 7.27 (m, 15H), 5.62 (dd, *J* = 3.4, 1.3 Hz, 1H), 4.90 (d, *J* = 3.7 Hz, 1H, **H$_1$**), 4.81 (d, *J* = 12.2 Hz, 1H), 4.73 (d, *J* = 10.8 Hz, 1H), 4.64 (d, *J* = 12.2 Hz, 1H), 4.55 – 4.50 (m, 2H), 4.45 (d, *J* = 11.8 Hz, 1H), 4.11 (td, *J* = 6.3, 1.3 Hz, 1H), 4.00 – 3.91 (m, 3H), 3.76 (dd, *J* = 10.0, 3.7 Hz, 1H), 3.50 – 3.41 (m, 2H), 1.13 (s, 9H) ppm.

^{13}**C NMR** (151 MHz, $CDCl_3$) δ 177.4, 138.4, 138.3, 137.8, 128.6, 128.5, 128.3, 128.1, 128.1, 128.0, 127.9, 127.6, 98.6 (**C_1**), 76.1, 74.6, 73.8, 73.6, 72.0, 68.8, 68.6, 67.4, 39.1, 27.3 ppm.

^{19}**F NMR** (564 MHz, $CDCl_3$) δ -73.54 (t, J = 8.7 Hz).

HRMS (QToF): Calcd for $C_{34}H_{39}F_3O_7Na$ $[M + Na]^+$ 639.2540; found 639.2535.

NP-HPLC of **4Piv-A3** (ELSD trace, t_R (α) = 5.5 min):

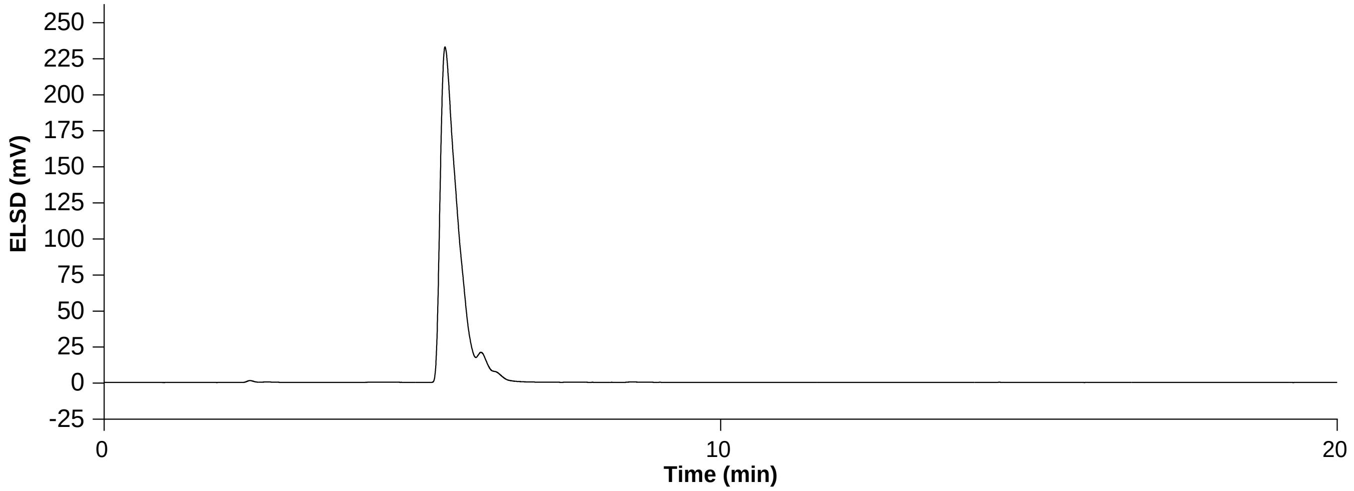

^{1}H NMR (600 MHz, $CDCl_3$) of **4Piv-A3**:

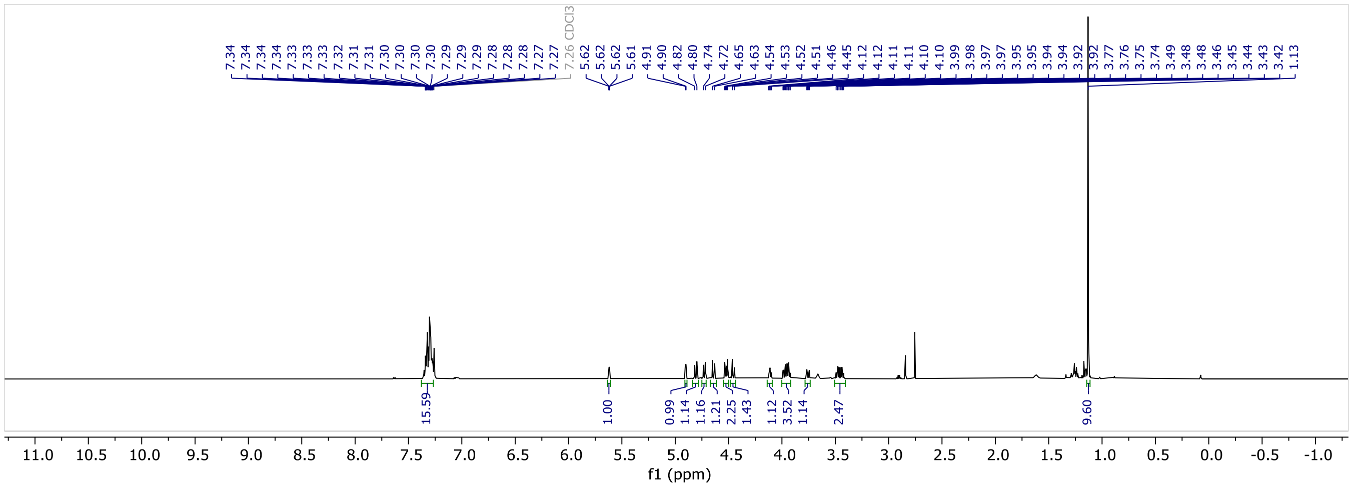

^{13}C NMR (151 MHz, $CDCl_3$) of **4Piv-A3**:

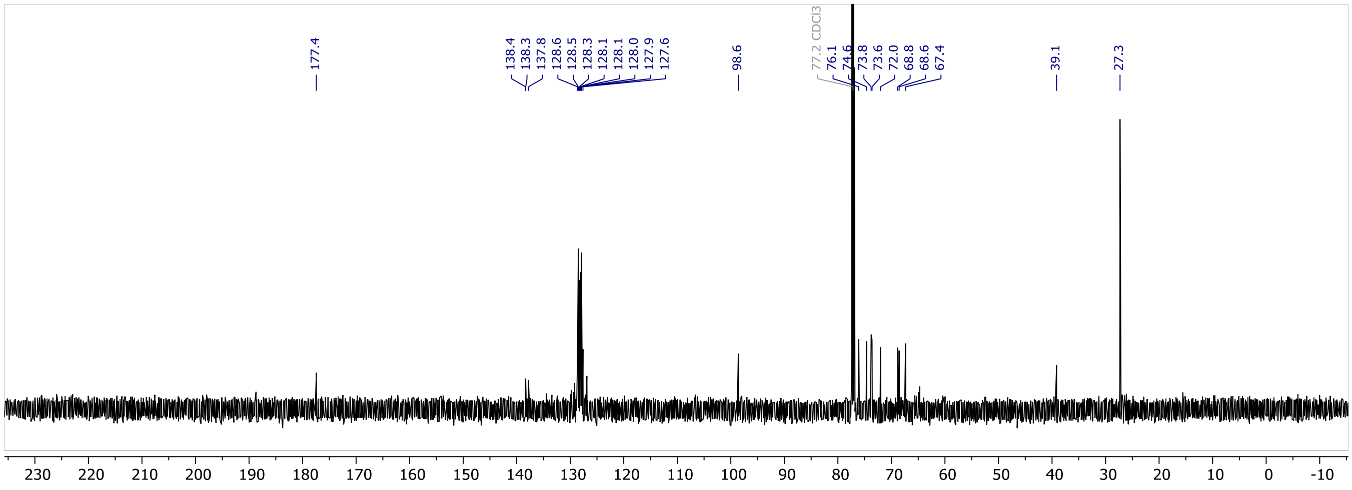

^{19}F NMR (376 MHz, $CDCl_3$) of **4Piv-A3**:

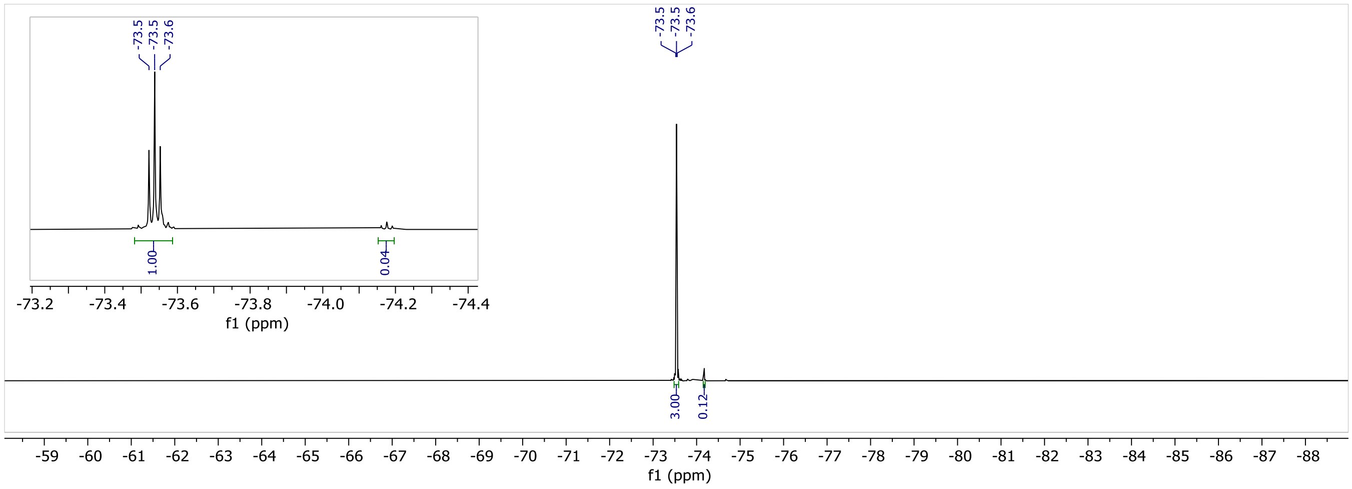

Coupled $^{13}C,^{1}H$ HSQC of **4Piv-A3**:

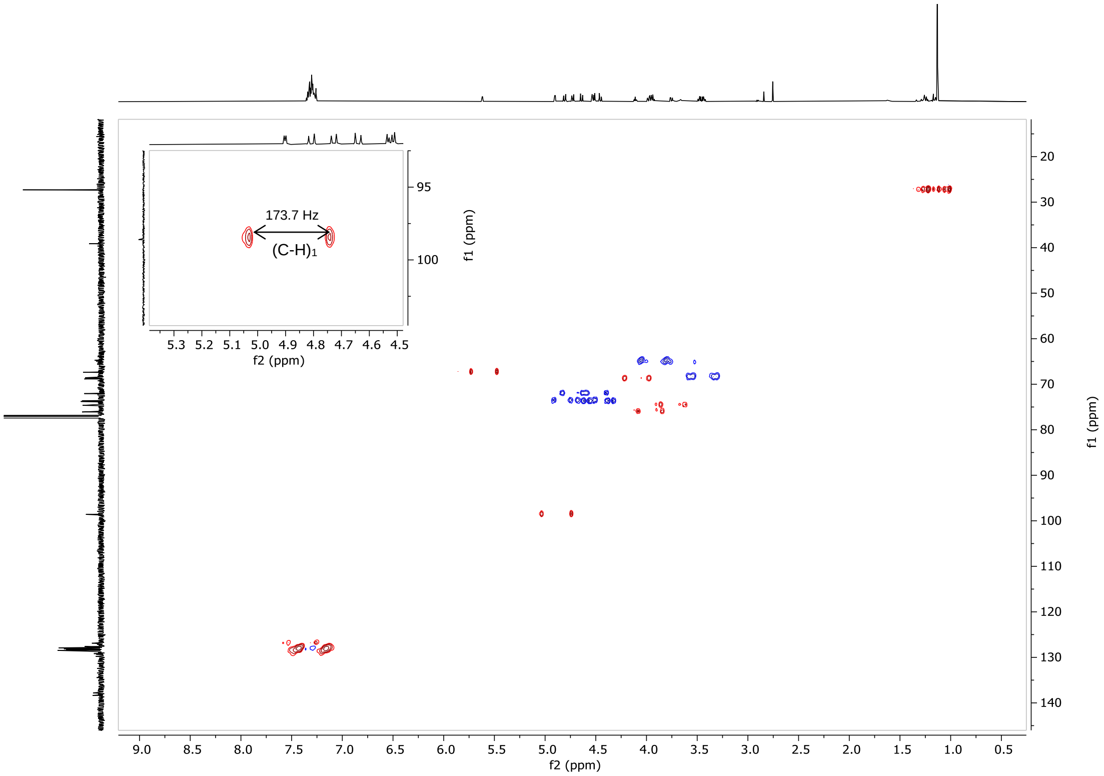

2,2,2-Trifluoroethyl 2,3,4-tris-*O*-benzyl-6-(trifluoroacetate)-α/β-D-galactopyranoside (6TFA-A3)

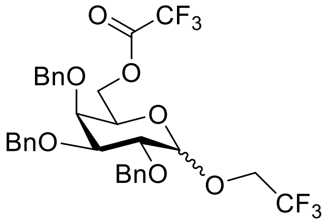

The title compound was prepared according to general procedure for glycosylations. Product **6TFA-A3** (14 mg, 22 μmol, 66%, **α/β 96:4**) was obtained as a colorless oil.

Data of the major isomer (α):

^{1}H NMR (600 MHz, $CDCl_3$) δ 7.45 – 7.26 (m, 15H), 5.00 (d, *J* = 11.6 Hz, 1H), 4.92 (d, *J* = 11.6 Hz, 1H), 4.86 – 4.75 (m, 3H, **H_1**), 4.69 – 4.57 (m, 2H), 4.44 (dd, *J* = 11.3, 7.9 Hz, 1H), 4.07 (ddd, *J* = 11.4, 6.9, 3.9 Hz, 2H), 4.01 – 3.92 (m, 2H), 3.90 – 3.83 (m, 3H) ppm.

^{13}C NMR (151 MHz, $CDCl_3$) δ 157.1, 156.8, 138.4, 138.2, 137.7, 128.5, 128.5, 128.4, 128.1, 128.0, 127.9, 127.8, 127.6, 115.3, 113.4, 98.3 (**C_1**), 78.1, 76.0, 74.6, 74.2, 73.9, 73.6, 68.7, 66.8, 64.8 (q, *J* = 35.0 Hz) ppm.

^{19}F NMR (564 MHz, $CDCl_3$) δ -73.87 (t, *J* = 8.7 Hz), -75.11 ppm.

^{1}H NMR (600 MHz, $CDCl_3$) of **6TFA-A3**:

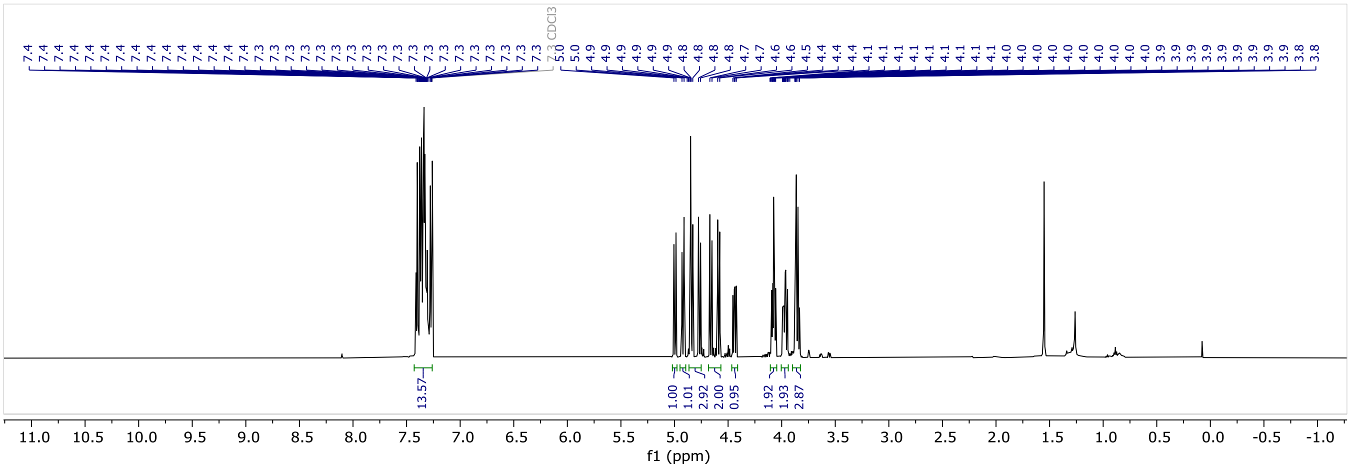

^{13}C NMR (151 MHz, $CDCl_3$) of **6TFA-A3**:

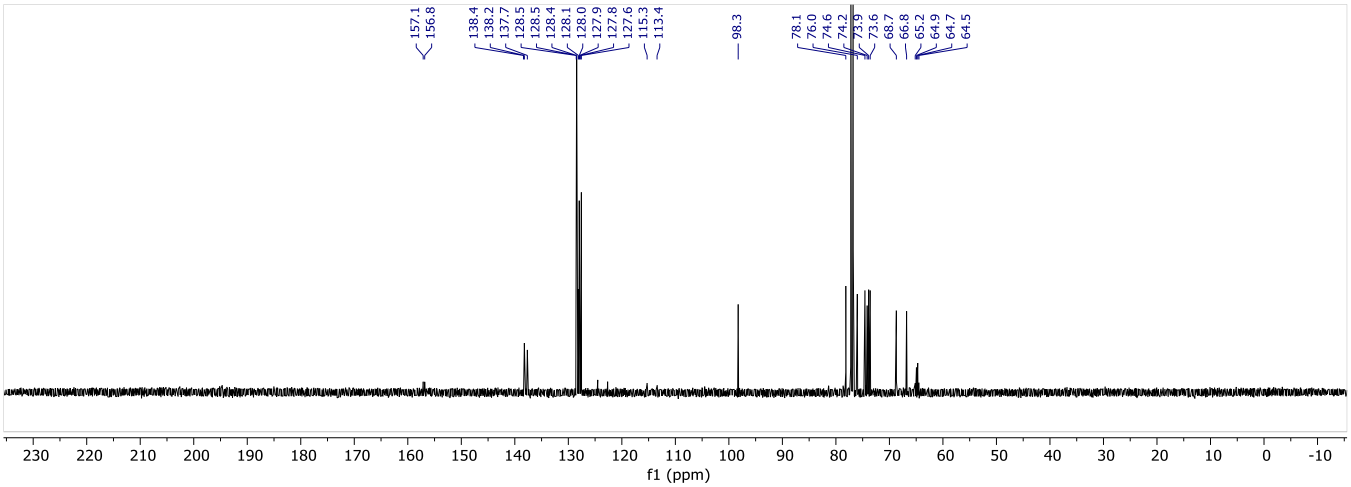

^{19}F NMR (376 MHz, $CDCl_3$) of **6TFA-A3**:

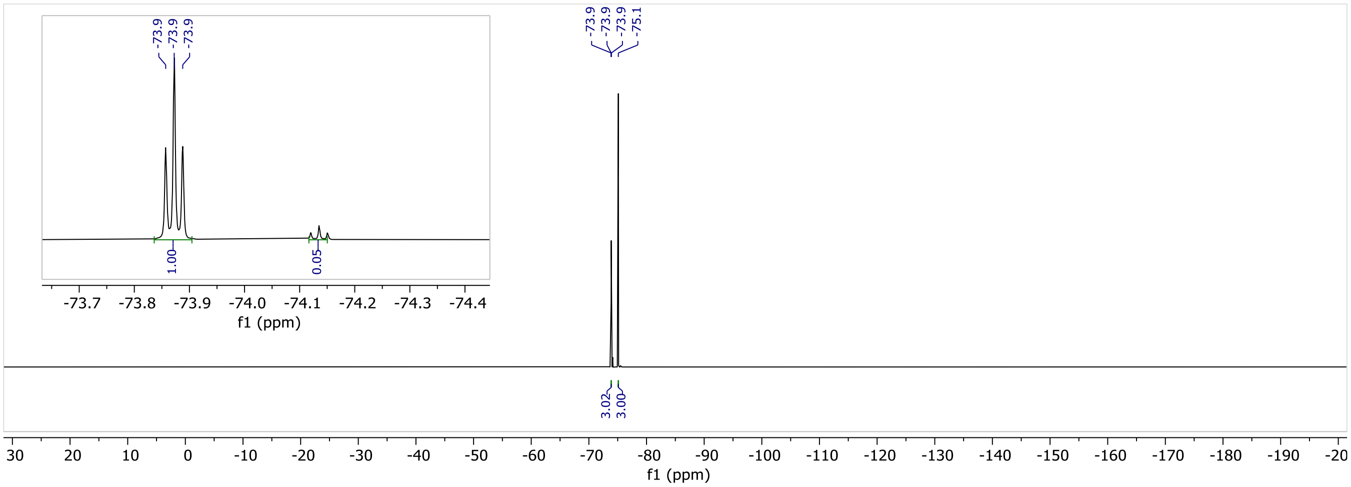

Coupled $^{13}C,^{1}H$ HSQC of **6TFA-A3**:

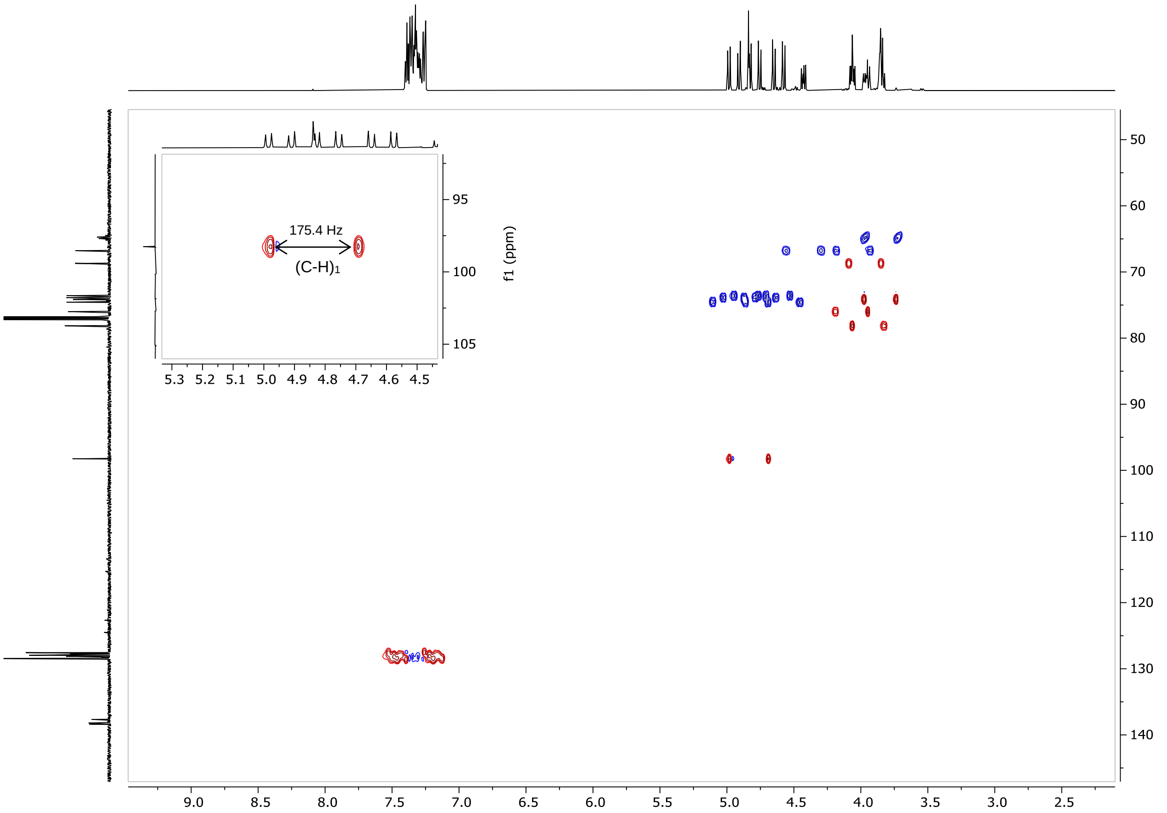

2,2,2-Trifluoroethyl 2,3,6-tris-*O*-benzyl-4-(trifluoroacetate)-α-D-galactopyranoside (4TFA-A3)

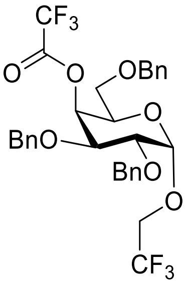

The title compound was prepared according to general procedure for glycosylations. Product **4TFA-A3** (15 mg, 24 µmol, 71%, **α-only**) was obtained as a colorless oil.

Data of the major isomer (α):

^{1}H NMR (600 MHz, $CDCl_3$) δ 7.38 – 7.26 (m, 15H), 5.73 (dd, *J* = 3.2, 1.3 Hz, 1H), 4.85 (d, *J* = 3.7 Hz, 1H, **H_1**), 4.83 – 4.72 (m, 2H), 4.61 (dd, *J* = 11.5, 2.2 Hz, 2H), 4.53 – 4.41 (m, 2H), 4.14 (ddd, *J* = 7.3, 5.7, 1.3 Hz,

1H), 4.03 (dd, J = 10.0, 3.3 Hz, 1H), 3.91 (qd, J = 8.6, 2.8 Hz, 2H), 3.77 (dd, J = 10.0, 3.7 Hz, 1H), 3.52 (dd, J = 9.4, 5.8 Hz, 1H), 3.42 (dd, J = 9.3, 7.9 Hz, 1H) ppm.

^{13}C NMR (151 MHz, $CDCl_3$) δ 156.92 (q, J = 42.4 Hz), 138.1, 137.7, 137.3, 128.7, 128.6, 128.5, 128.2, 128.2, 128.1, 127.9, 127.9, 113.8, 98.7 (**C_1**), 75.3, 74.8, 73.9, 73.9, 72.8, 72.5, 67.5, 67.3, 65.3 (q, J = 35.0 Hz) ppm.

^{19}F NMR (564 MHz, $CDCl_3$) δ -73.69 (t, J = 8.7 Hz), -74.82 ppm.

HRMS (QToF): Calcd for $C_{31}H_{30}F_6O_7Na$ $[M + Na]^+$ 651.1788; found 651.1815.

^{1}H NMR (600 MHz, $CDCl_3$) of **4TFA-A3**:

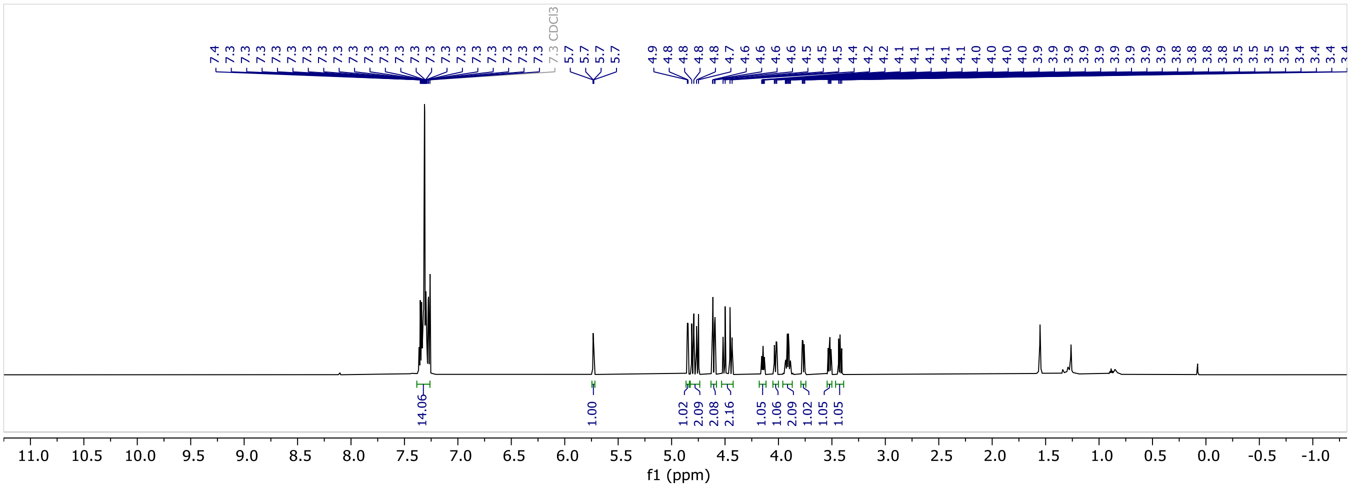

^{13}C NMR (151 MHz, $CDCl_3$) of **4TFA-A3**:

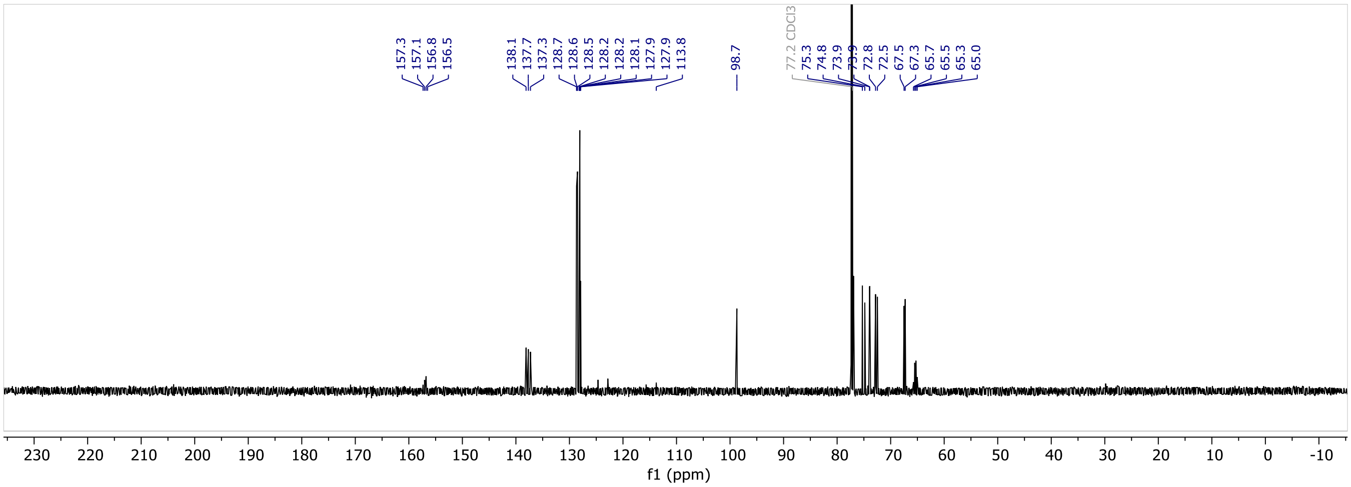

^{19}F NMR (376 MHz, $CDCl_3$) of **4TFA-A3**:

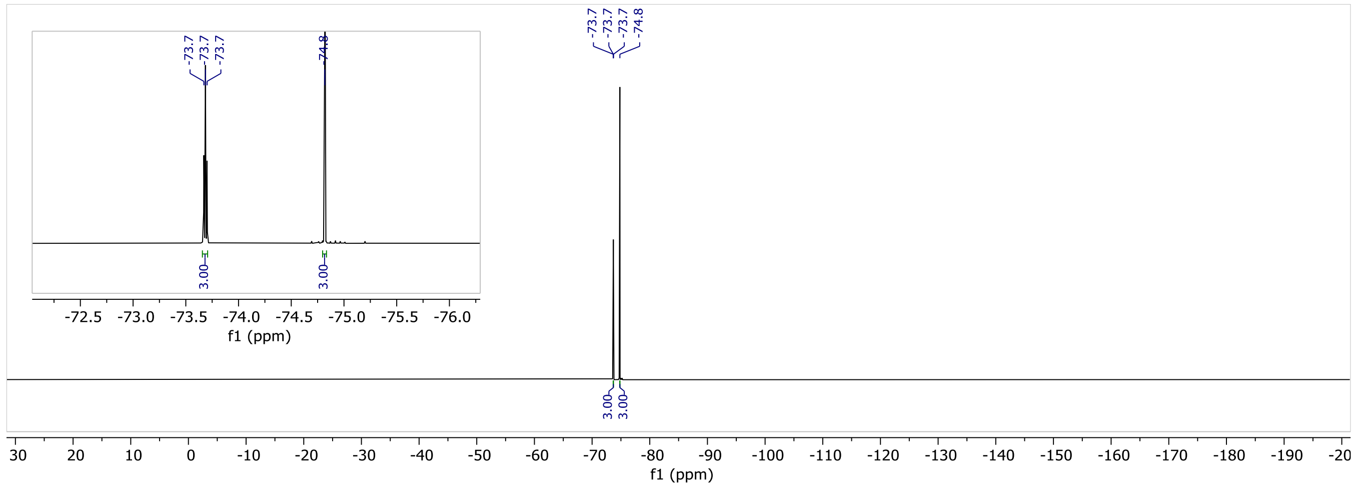

Coupled $^{13}C,^{1}H$ HSQC of **4TFA-A3**:

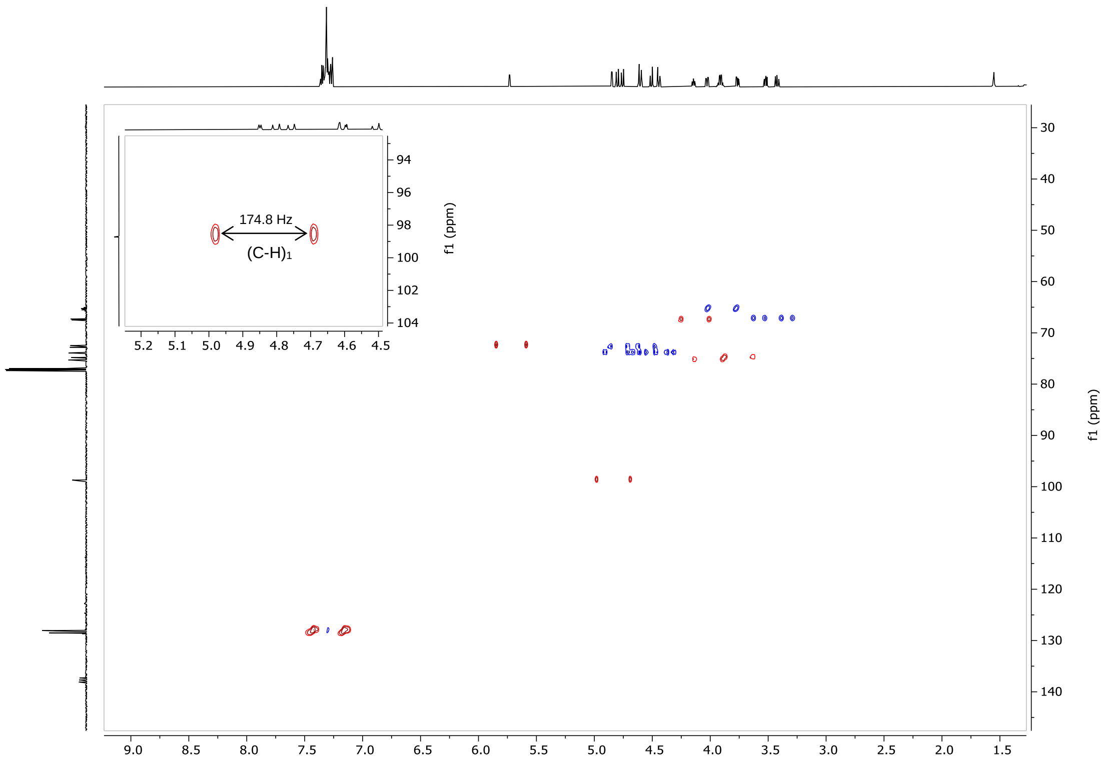

Benzyl 2,3,4-tris-*O*-benzyl-6-(2,2-dimethylpropanoate)-α/β-D-galactopyranoside (6Piv-A4)

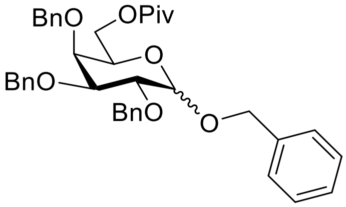

The title compound was prepared according to general procedure for glycosylations. Product **6Piv-A4** (18 mg, 29 μmol, 83%, **α/β 36:64**) was obtained as a colorless oil after purification using **Method-2b** (t_R (α/ β) = 6.7 min).

Data of the anomeric mixture:

^{1}H NMR (600 MHz, $CDCl_3$) δ 7.44 – 7.27 (m, 26H), 5.03 – 4.88 (m, 4H, **H_1(α)**), 4.85 – 4.69 (m, 5H), 4.68 – 4.52 (m, 4H), 4.45 (d, *J* = 7.7 Hz, 1H, **H_1(β)**), 4.31 (dd, *J* = 11.1, 7.0 Hz, 1H), 4.18 (dd, *J* = 11.2, 7.4 Hz, 1H), 4.13 – 3.95 (m, 3H), 3.91 (dd, *J* = 9.7, 7.7 Hz, 1H), 3.88 – 3.85 (m, 1H), 3.74 (d, *J* = 2.8 Hz, 1H), 3.57 – 3.49 (m, 2H), 1.19 (s, 4H), 1.19 (s, 9H) ppm.

^{13}C NMR (151 MHz, $CDCl_3$) δ 178.2, 138.9, 138.8, 138.6, 138.6, 138.5, 138.4, 137.6, 137.2, 128.5, 128.5, 128.5, 128.4, 128.4, 128.3, 128.3, 128.1, 128.0, 127.9, 127.8, 127.7, 102.7 **C_1(β)**, 95.7 **C_1(α)**, 82.3, 79.6, 79.2, 76.5, 75.5, 75.3, 74.9, 74.7, 73.9, 73.7, 73.6, 73.3, 72.3, 70.9, 68.9, 68.7, 63.8, 63.2, 38.9, 27.3 ppm.

HRMS (QToF): Calcd for $C_{39}H_{44}O_7Na$ $[M + Na]^+$ 647.2979; found 647.2974.

NP-HPLC of **6Piv-A4** (ELSD trace, t_R (α/ β) = 6.7 min):

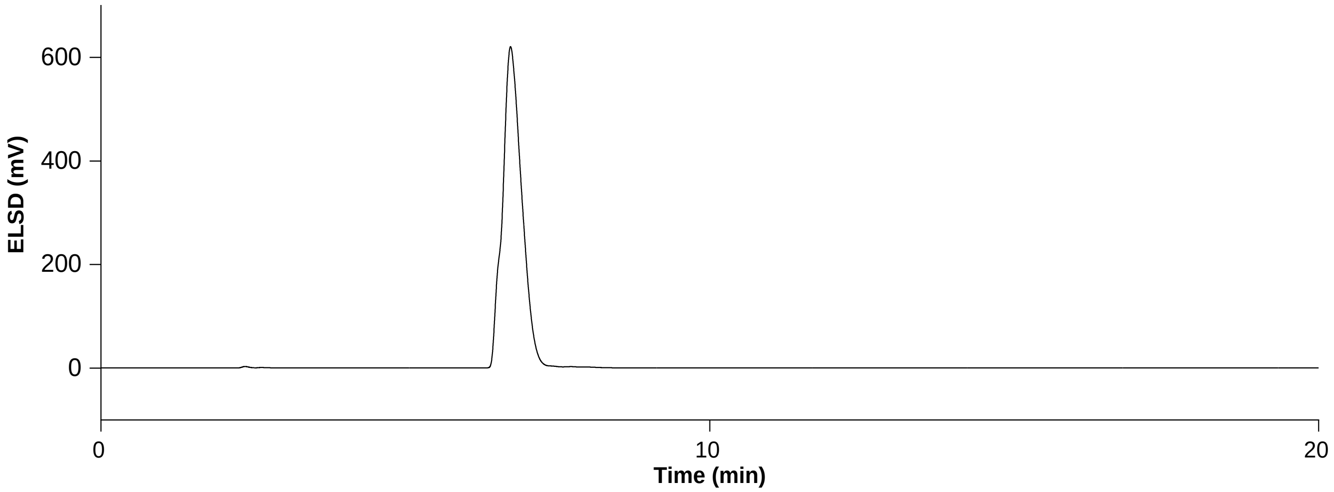

^{1}H NMR (600 MHz, $CDCl_3$) of **6Piv-A4**:

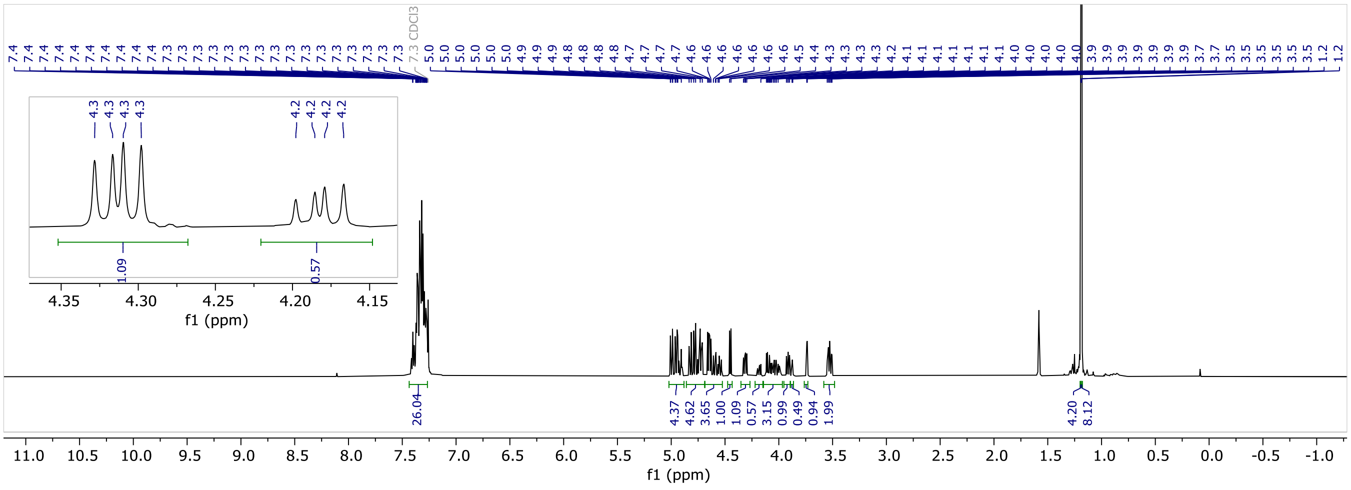

^{13}C NMR (151 MHz, $CDCl_3$) of **6Piv-A4**:

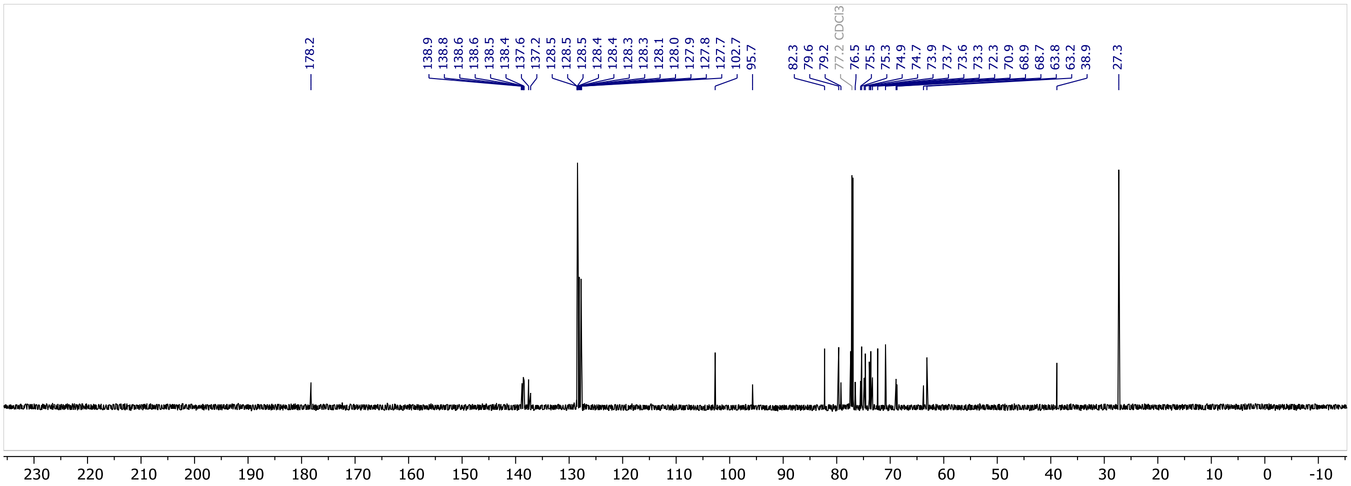

Coupled $^{13}C,^{1}H$ HSQC of **6Piv-A4**:

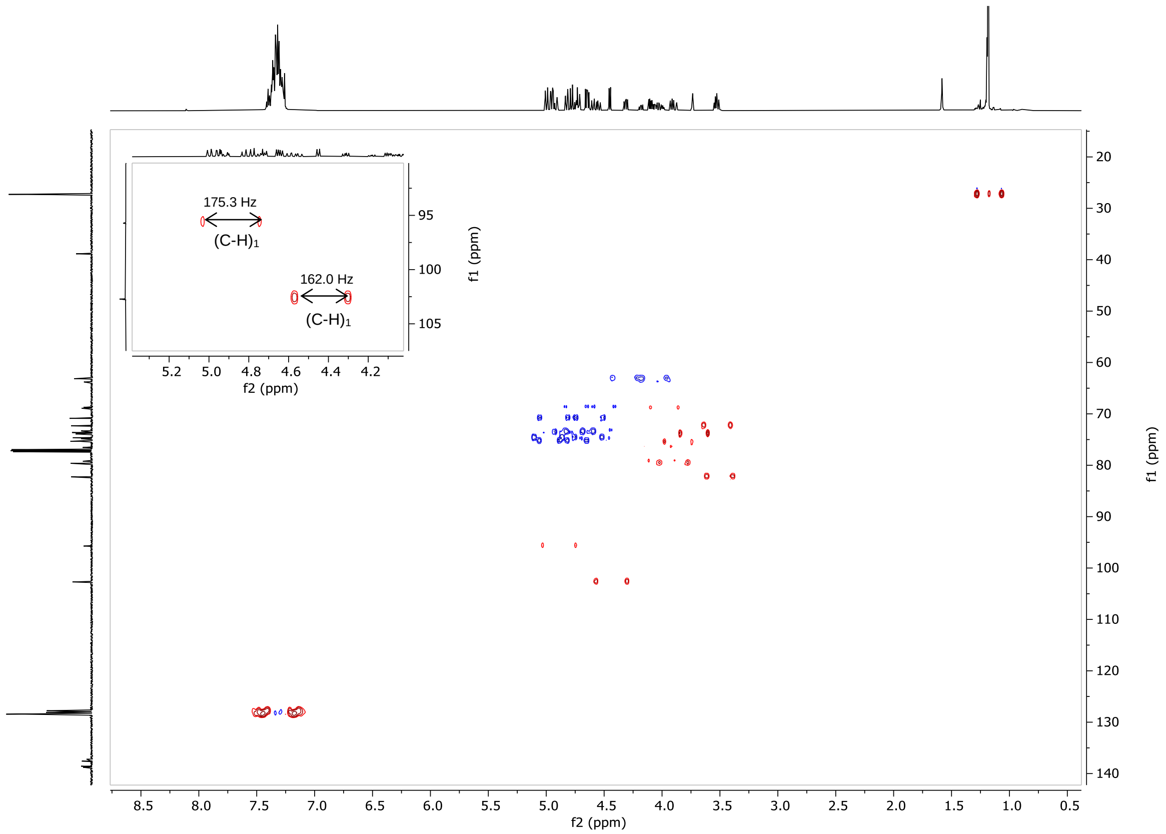

Benzyl 2,3-bis-*O*-benzyl-4,6-bis-(2,2-dimethylpropanoate)-α/β-D-galactopyranoside (4,6Piv-A4)

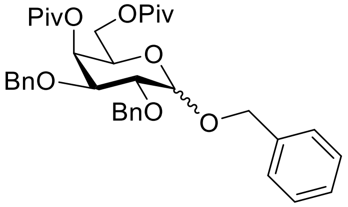

The title compound was prepared according to general procedure for glycosylations. Product **4,6Piv-A4** (18 mg, 29 µmol, 83%, **α/β 66:34**) was obtained as a colorless oil after purification using **Method-2b** (t_R (α) = 5.0 min, t_R (β) = 5.6 min).

Data of the anomeric mixture:

^{1}H NMR (600 MHz, $CDCl_3$) δ 7.46 – 7.27 (m, 22H), 5.59 (d, *J* = 2.9 Hz, 1H), 5.50 (d, *J* = 3.4 Hz, 1H), 5.01 – 4.87 (m, 2H, **H_1(α)**), 4.83 – 4.67 (m, 5H), 4.64 – 4.49 (m, 4H, **H_1(β)**), 4.29 – 4.12 (m, 2H), 4.12 – 4.04 (m, 3H), 3.83 (t, *J* = 6.8 Hz, 1H), 3.75 (dd, *J* = 10.0, 3.7 Hz, 1H), 3.66 – 3.55 (m, 1H), 1.25 (s, 4H), 1.25 (s, 9H), 1.23 (s, 4H), 1.18 (d, *J* = 0.8 Hz, 9H) ppm.

^{13}C NMR (151 MHz, $CDCl_3$) δ 178.2, 178.1, 177.7, 177.5, 138.6, 138.4, 138.4, 138.1, 137.4, 137.0, 128.6, 128.6, 128.4, 128.4, 128.3, 128.3, 128.3, 128.1, 128.1, 128.0, 127.8, 127.7, 127.6, 102.5 **C_1(β)**, 96.1 **C_1(α)**, 79.4, 78.7, 76.4, 75.4, 74.9, 73.5, 72.2, 72.2, 71.2, 71.1, 69.1, 67.4, 67.3, 66.2, 62.6, 62.1, 39.2, 39.1, 38.9, 27.3, 27.3, 27.3 ppm.

HRMS (QToF): Calcd for $C_{37}H_{46}O_8Na$ $[M + Na]^+$ 641.3085; found 641.3082.

NP-HPLC of **4,6Piv-A4** (ELSD trace, t_R (α) = 5.0 min, t_R (β) = 5.6 min):

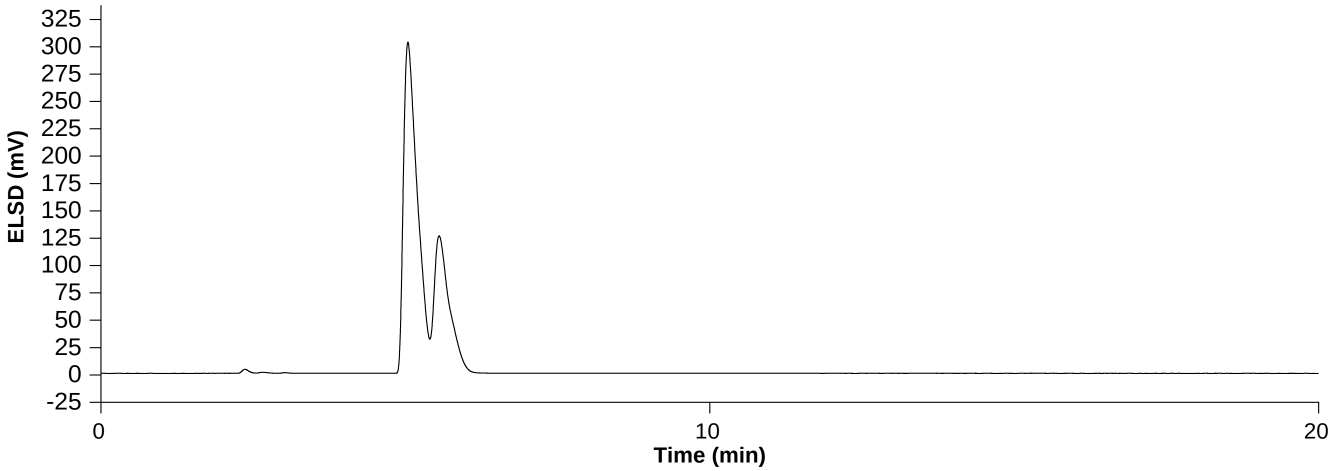

^{1}H NMR (600 MHz, $CDCl_3$) of **4,6Piv-A4**:

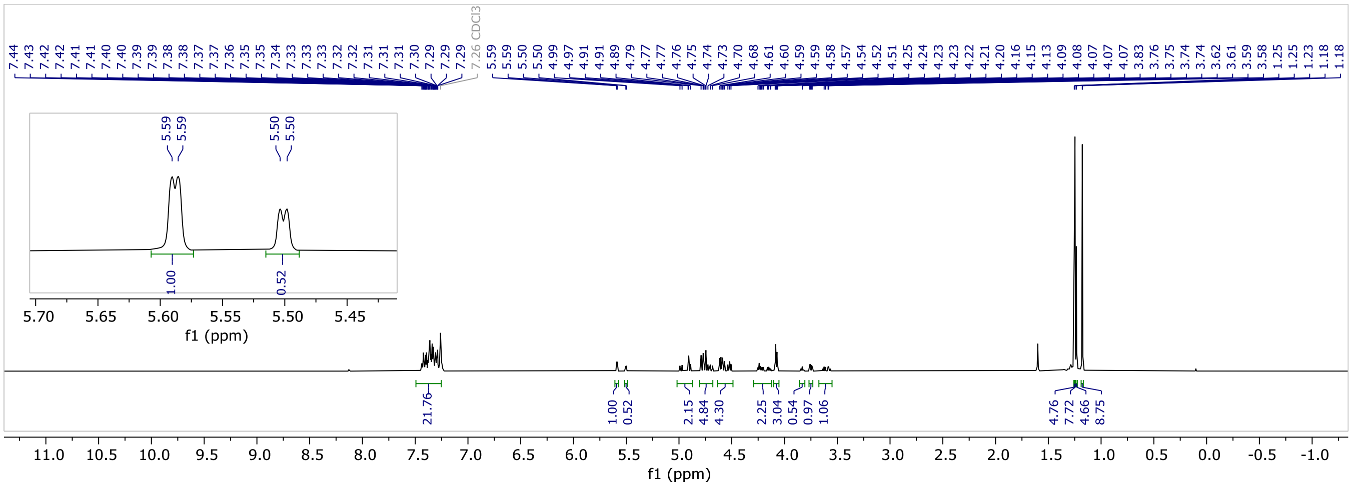

^{13}C NMR (151 MHz, $CDCl_3$) of **4,6Piv-A4**:

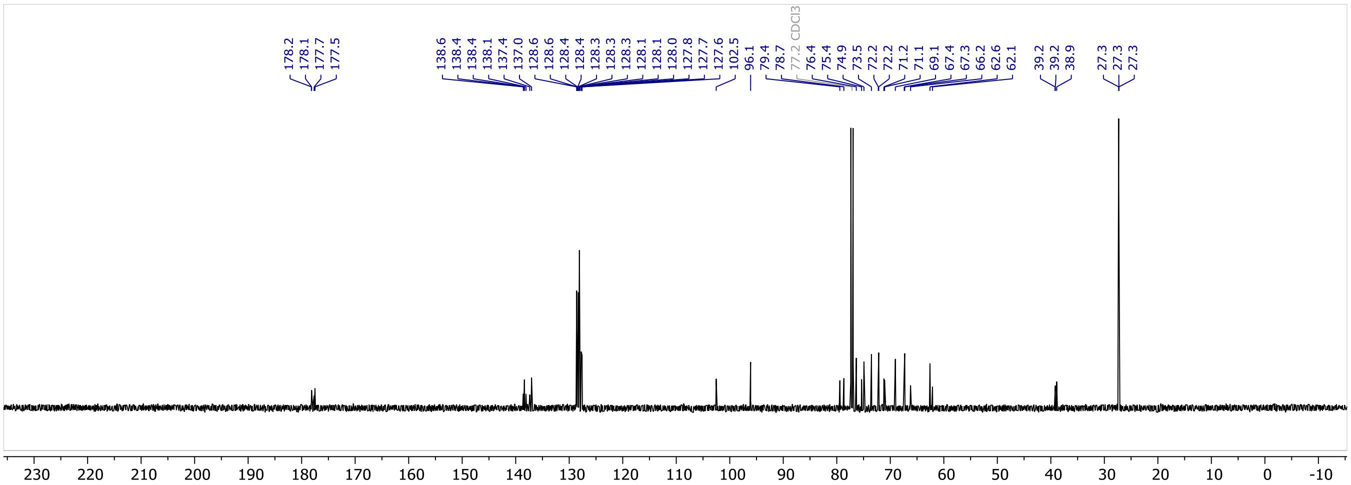

Coupled ^{13}C,^{1}H HSQC of **4,6Piv-A4**:

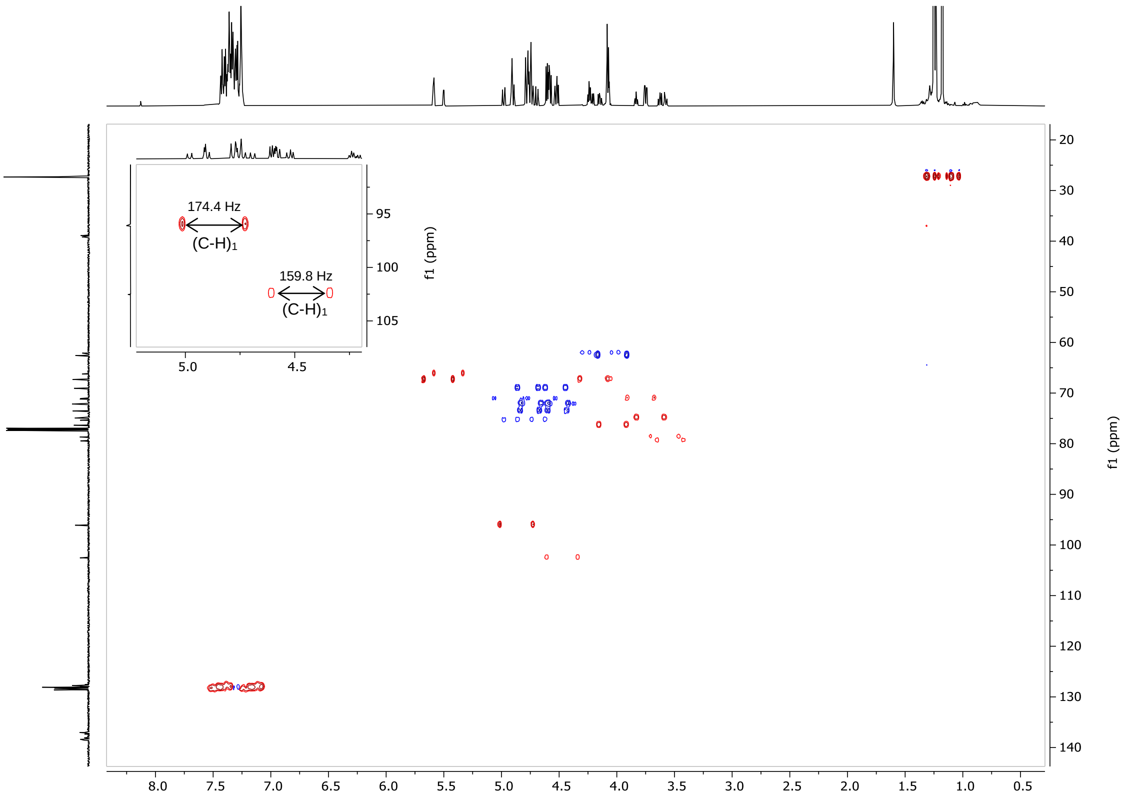

Benzyl 2,3,6-tris-*O*-benzyl-4-(2,2-dimethylpropanoate)-α/β-D-galactopyranoside (4Piv-A4)

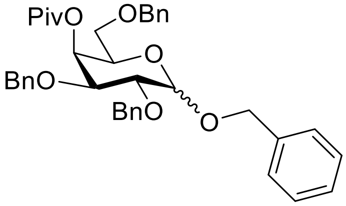

The title compound was prepared according to general procedure for glycosylations. Product **4Piv-A4** (16 mg, 26 μmol, 74%, **α/β 68:32**) was obtained as a colorless oil after purification using **Method-2b** (t_R (α) = 6.8 min, t_R (β) = 8.1 min).

Data of the major isomer (α):

^{1}H NMR (600 MHz, $CDCl_3$) δ 7.43 – 7.25 (m, 15H), 5.61 (d, *J* = 3.0 Hz, 1H), 4.99 – 4.83 (m, 2H, **H_1**), 4.77 – 4.66 (m, 4H), 4.63 – 4.42 (m, 6H), 4.16 (t, *J* = 6.4 Hz, 1H), 4.03 (dd, *J* = 10.0, 3.3 Hz, 1H), 3.77 – 3.69 (m, 1H), 3.64 – 3.50 (m, 2H), 3.43 (dd, *J* = 6.3, 1.2 Hz, 2H), 1.12 (s, 9H) ppm.

^{13}C NMR (151 MHz, $CDCl_3$) δ 177.5, 138.5, 138.0, 137.3, 128.6, 128.5, 128.5, 128.3, 128.3, 128.1, 127.9, 127.7, 127.6, 96.4 (**C_1**), 76.7, 75.1, 73.8, 73.5, 72.0, 69.3, 68.8, 68.3, 67.7, 39.1, 27.3 ppm.

HRMS (QToF): Calcd for $C_{39}H_{44}O_7Na$ $[M + Na]^+$ 647.2979; found 647.2978.

NP-HPLC of **4Piv-A4** (ELSD trace, t_R (α) = 6.8 min, t_R (β) = 8.1 min):

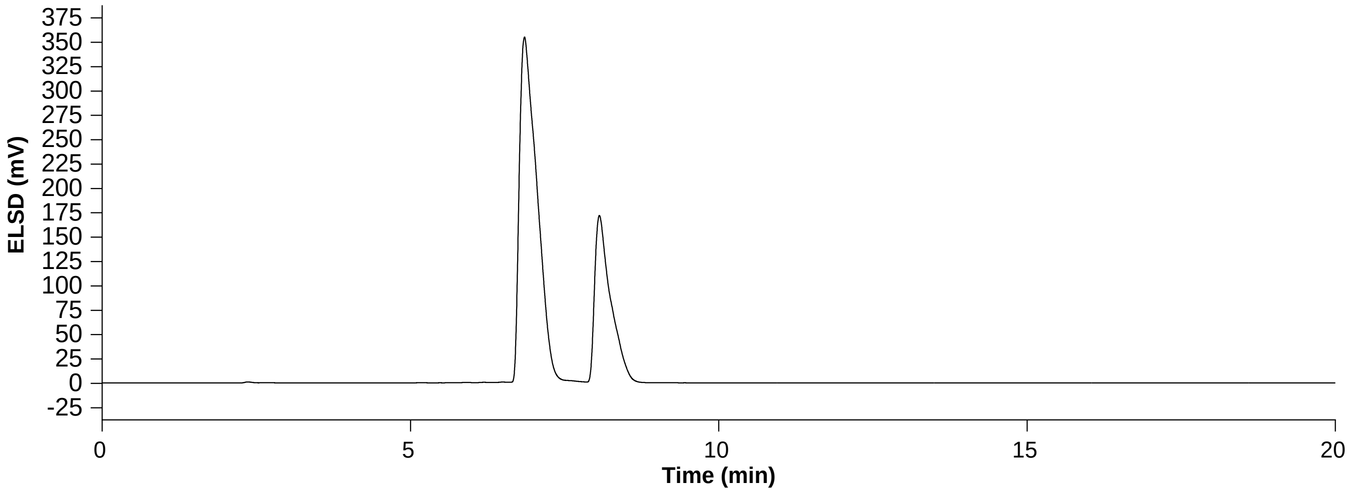

^{1}H NMR (600 MHz, $CDCl_3$) of **4Piv-A4**:

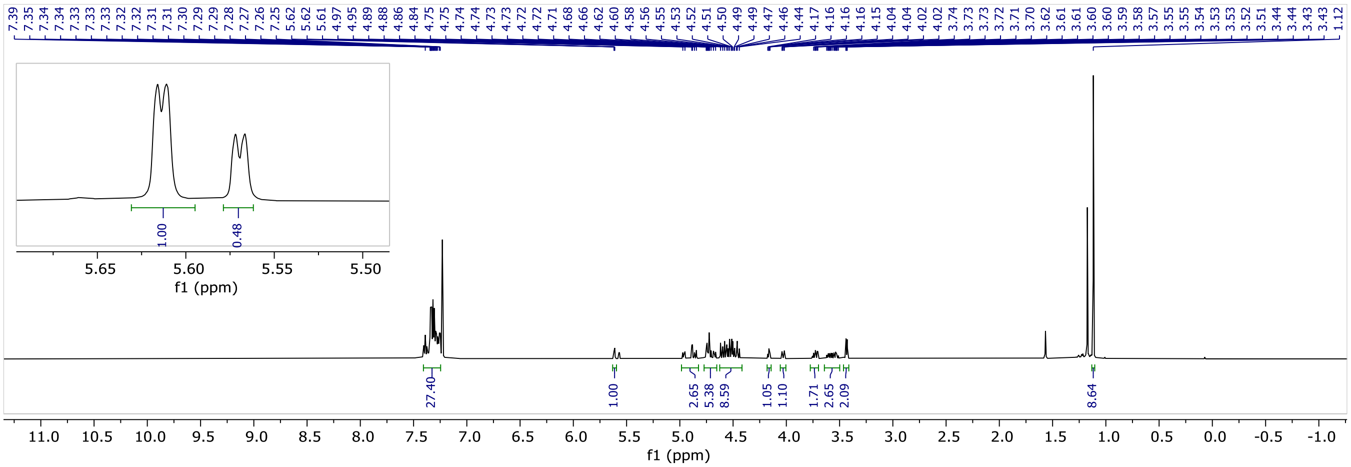

^{13}C NMR (151 MHz, $CDCl_3$) of **4Piv-A4**:

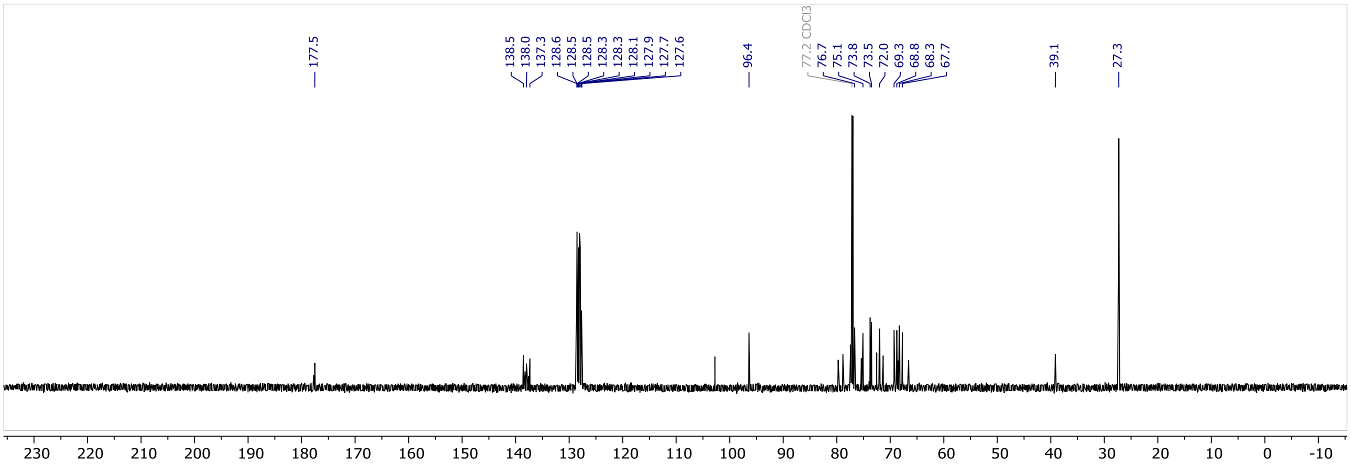

Coupled $^{13}C,^{1}H$ HSQC of **4Piv-A4**:

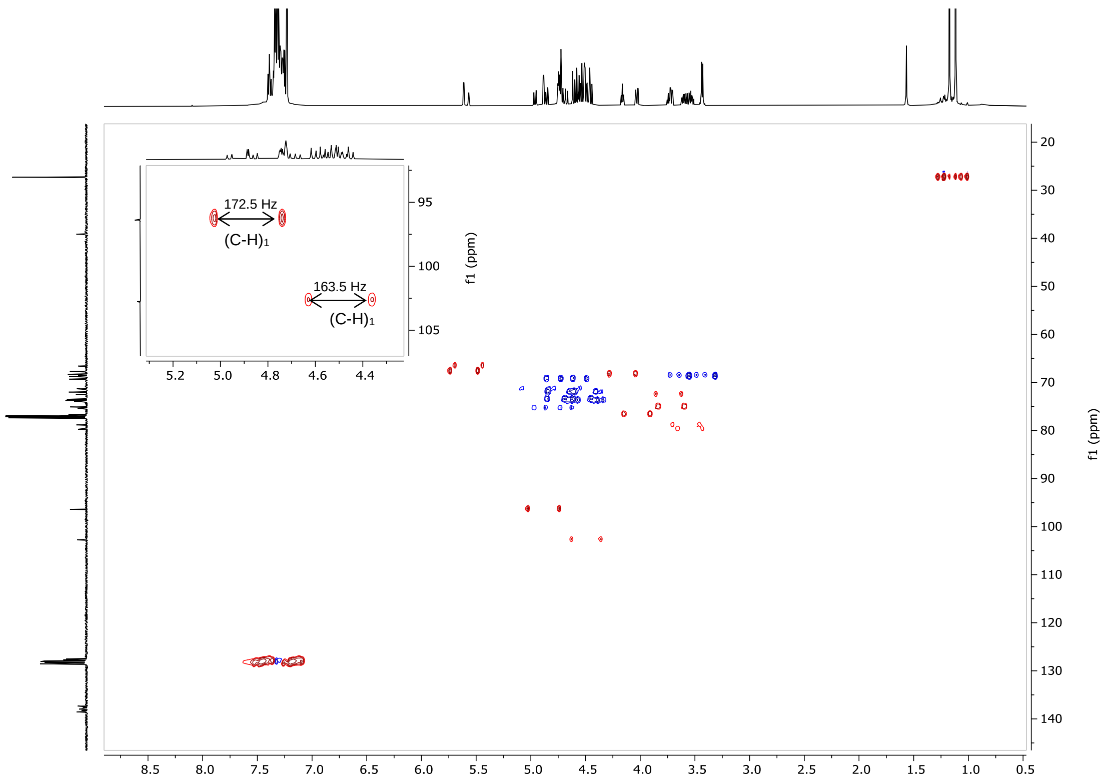

Benzyl 2,3,6-tris-*O*-benzyl-4-(trifluoroacetate)-α/β-D-galactopyranoside (4TFA-A4)

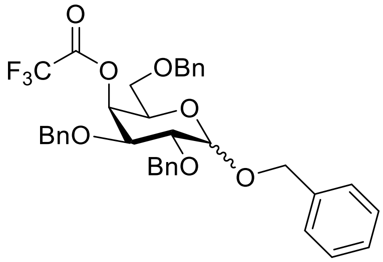

The title compound was prepared according to general procedure for glycosylations. Product **4TFA-A4** (10 mg, 16 µmol, 47%, **α/β 54:46**) was obtained as a colorless oil after purification using **Method-2b** (t_R (α/ β) = 6.1 min).

Data of the anomeric mixture:

^{1}H NMR (600 MHz, $CDCl_3$) δ 7.38 – 7.19 (m, 39H), 5.70 (dd, *J* = 3.4, 1.4 Hz, 1H), 5.66 (q, *J* = 1.3 Hz, 1H), 4.92 (d, *J* = 11.9 Hz, 1H), 4.88 – 4.82 (m, 2H), 4.77 – 4.39 (m, 13H), 4.13 (ddd, *J* = 7.4, 5.7, 1.4 Hz, 1H), 4.06

(dd, J = 10.0, 3.3 Hz, 1H), 3.76 (ddd, J = 8.3, 5.5, 1.1 Hz, 1H), 3.70 (dd, J = 10.0, 3.7 Hz, 1H), 3.64 (dd, J = 9.2, 5.6 Hz, 1H), 3.60 (dd, J = 4.5, 1.6 Hz, 2H), 3.50 (dd, J = 9.3, 8.2 Hz, 1H), 3.42 (dd, J = 9.2, 5.8 Hz, 1H), 3.37 (dd, J = 9.3, 7.9 Hz, 1H) ppm.

^{13}C NMR (151 MHz, $CDCl_3$) δ 170.2, 170.1, 157.3, 157.2, 157.0, 156.9, 138.4, 138.3, 137.9, 137.5, 137.4, 137.3, 137.2, 137.1, 132.6, 130.1, 128.7, 128.6, 128.6, 128.5, 128.4, 128.3, 128.2, 128.1, 128.0, 127.9, 127.8, 127.8, 102.6, 96.5, 78.7, 75.8, 75.6, 75.2, 74.0, 73.7, 72.9, 72.7, 71.5, 71.2, 69.8, 67.5, 67.2, 67.0 ppm.

^{19}F NMR (564 MHz, $CDCl_3$) δ -74.58, -74.80 ppm.

HRMS (QToF): Calcd for $C_{36}H_{35}F_3O_7Na$ $[M + Na]^+$ 659.2227; found 659.2225.

NP-HPLC of **4TFA-A4** (ELSD trace, t_R (α) = 7.9 min, t_R (β) = 6.0 min):

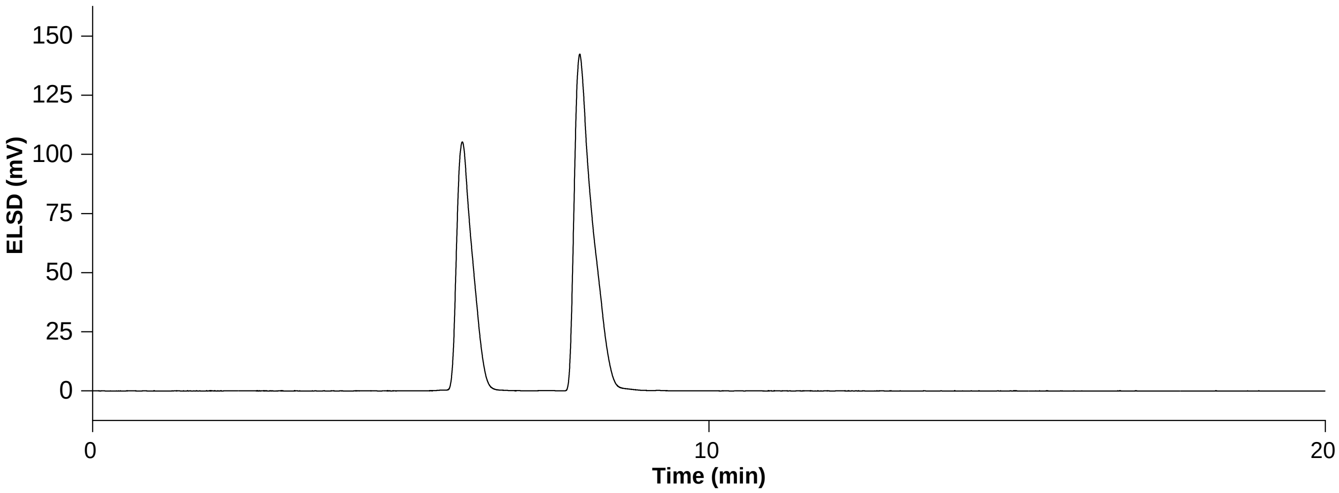

^{1}H NMR (600 MHz, $CDCl_3$) of **4TFA-A4**:

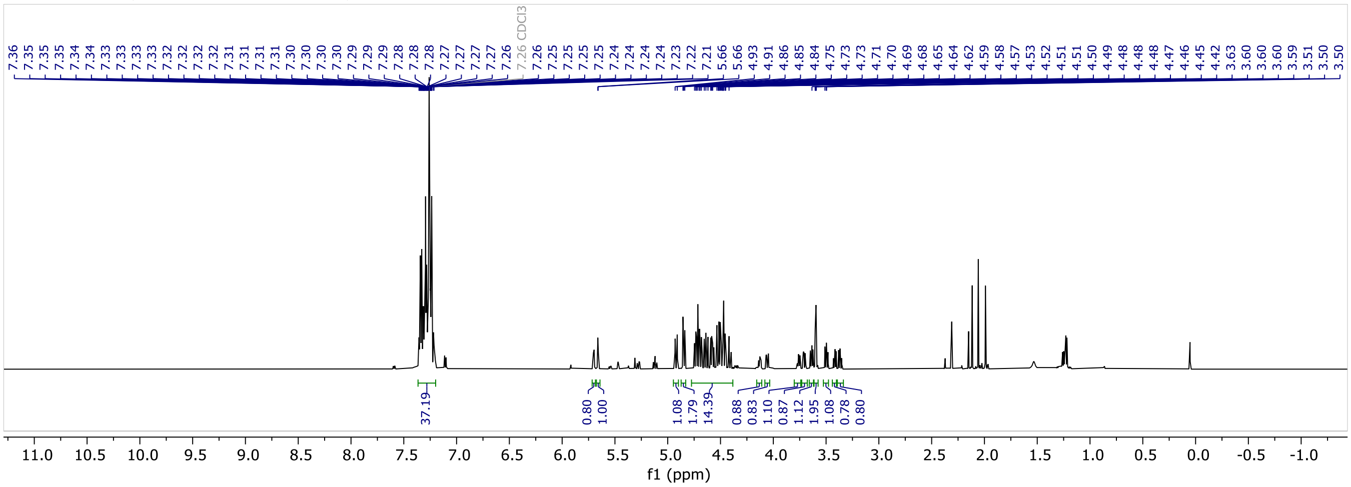

^{13}C NMR (151 MHz, $CDCl_3$) of **4TFA-A4**:

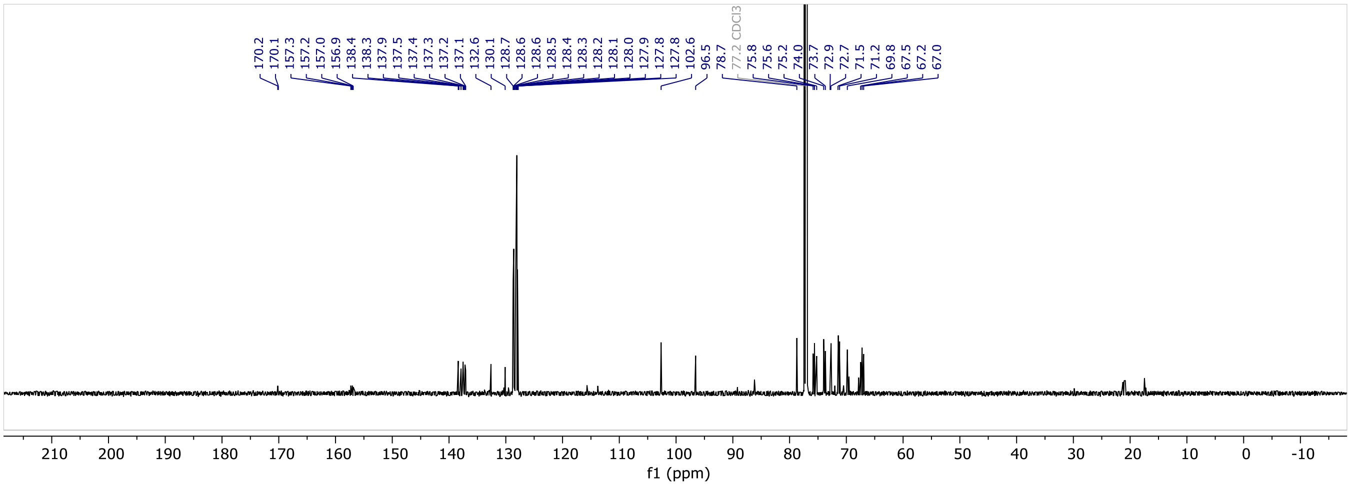

^{19}F NMR (564 MHz, $CDCl_3$) of **4TFA-A4**:

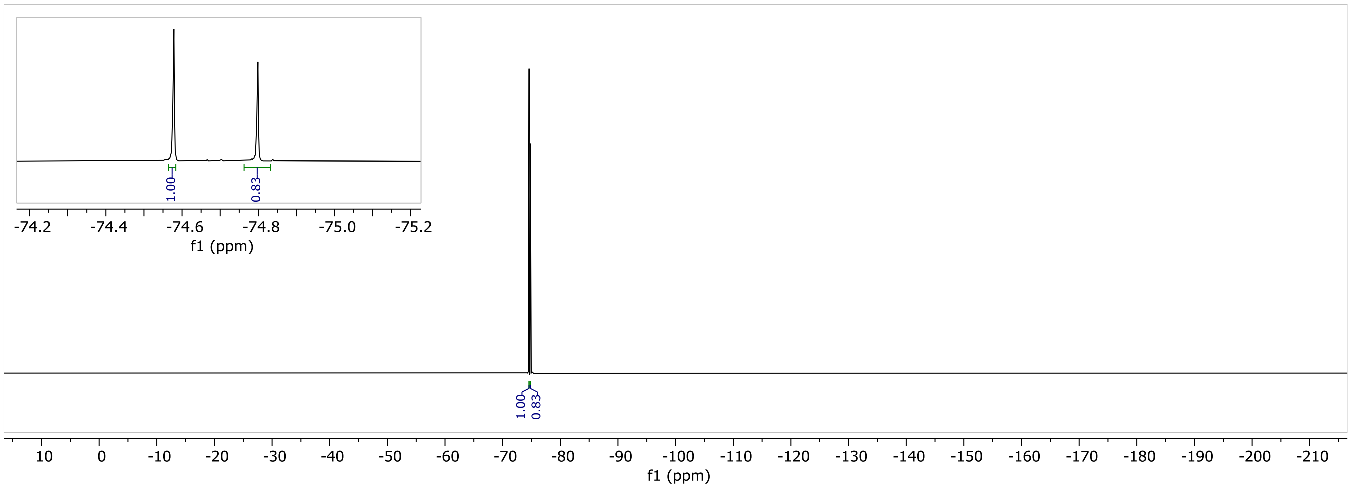

$^{13}C,^{1}H$ HSQC of **4TFA-A4**:

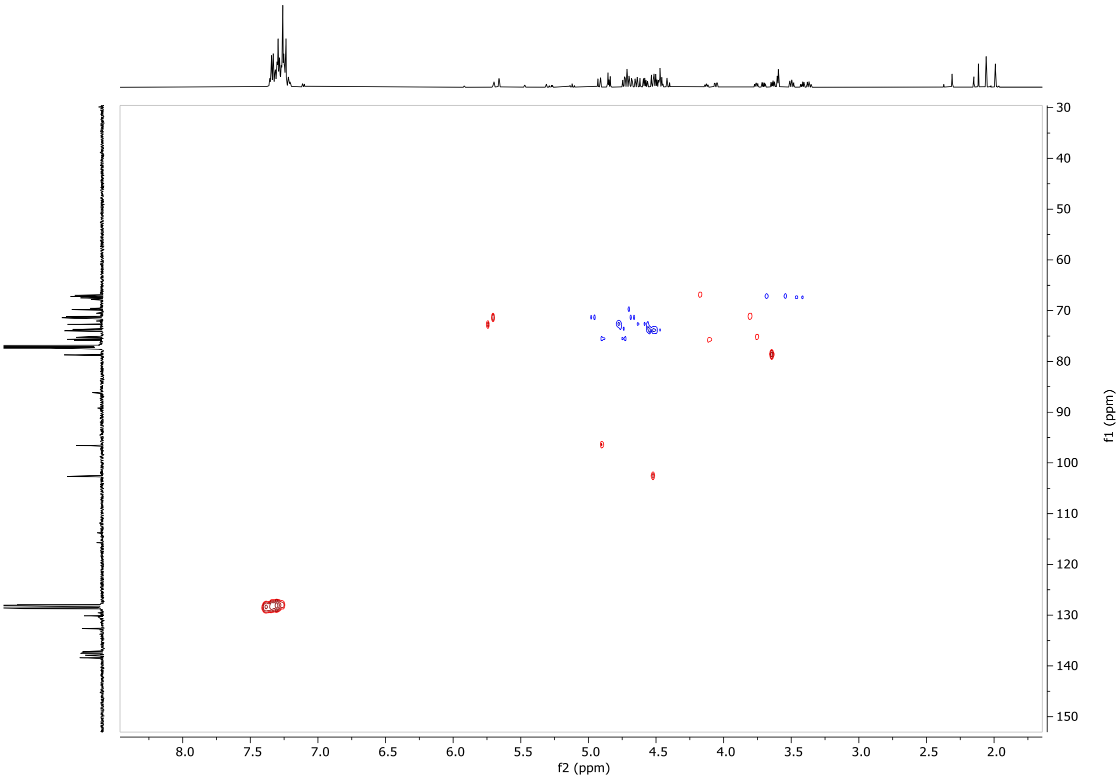

Coupled $^{13}C,^{1}H$ HSQC of **4TFA-A4**:

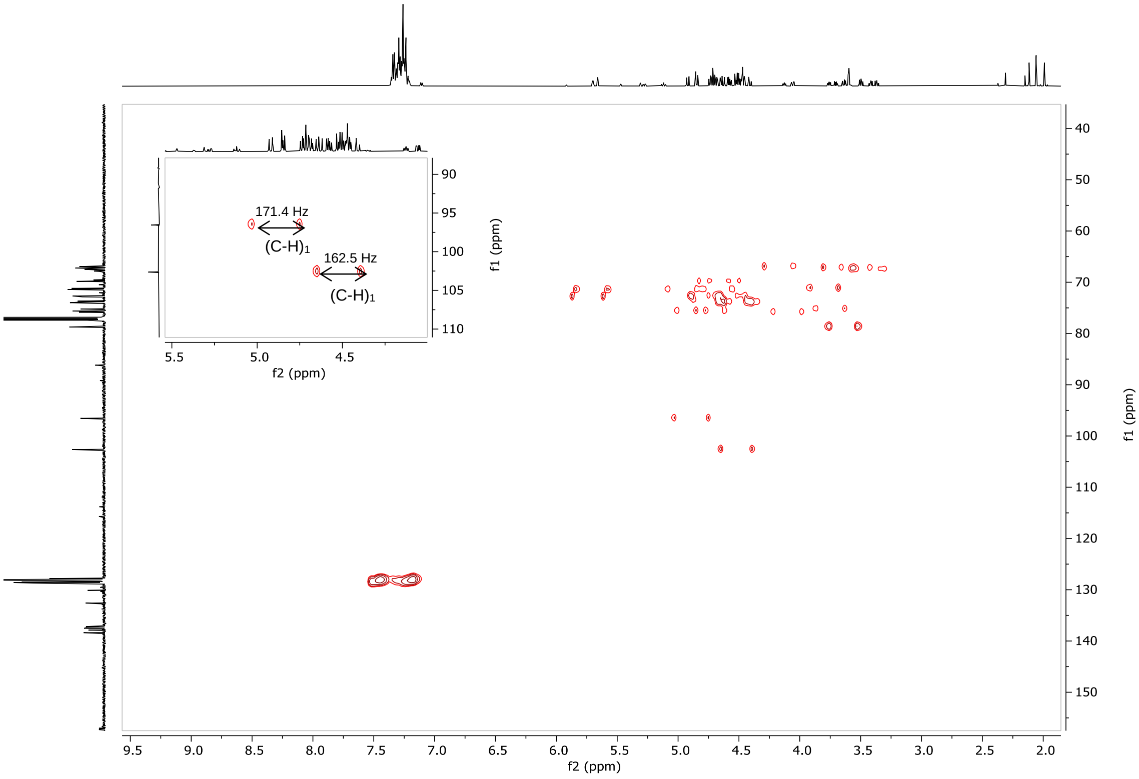

Benzyl 2,3,4-tris-*O*-benzyl-6-(trifluoroacetate)-α/β-D-galactopyranoside (6TFA-A4)

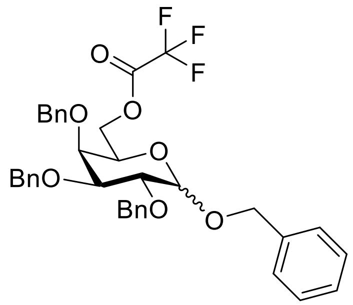

The title compound was prepared according to general procedure for glycosylations. Product **6TFA-A4** (11 mg, 17 μmol, 50%, **α/β 50:50**) was obtained as a colorless oil after purification using **Method-2b** (t_R (α) = 5.7 min, t_R (β) = 6.0 min).

Data of the anomeric mixture:

^{1}H NMR (600 MHz, $CDCl_3$) δ 7.44 – 7.26 (m, 40H), 5.00 (dd, *J* = 11.7, 6.3 Hz, 2H), 4.98 – 4.89 (m, 4H), 4.84 (d, *J* = 11.7 Hz, 1H), 4.76 (ddd, *J* = 20.7, 11.3, 2.1 Hz, 4H), 4.70 – 4.61 (m, 4H), 4.61 – 4.50 (m, 4H), 4.45 (d, *J* = 7.6 Hz, 1H), 4.41 (dd, *J* = 11.1, 7.5 Hz, 1H), 4.14 (dd, *J* = 11.2, 5.1 Hz, 1H), 4.09 – 4.05 (m, 1H), 4.04 – 3.99 (m, 2H), 3.92 (dd, *J* = 9.7, 7.6 Hz, 1H), 3.86 (dd, *J* = 2.6, 1.4 Hz, 1H), 3.75 (dd, *J* = 3.0, 1.3 Hz, 1H), 3.60 (ddd, *J* = 7.4, 5.1, 1.3 Hz, 1H), 3.53 (dd, *J* = 9.7, 2.9 Hz, 1H) ppm.

^{13}C NMR (151 MHz, $CDCl_3$) δ 157.1 (q, *J* = 42.5 Hz), 138.7, 138.6, 138.5, 138.4, 138.0, 137.9, 137.2, 137.0, 128.7, 128.6, 128.6, 128.6, 128.6, 128.5, 128.5, 128.4, 128.3, 128.2, 128.2, 128.2, 128.1, 128.0, 128.0, 127.9, 127.8, 127.8, 127.8, 127.7, 115.5, 115.5, 113.6, 113.6, 102.4, 95.9, 82.0, 79.4, 79.0, 76.5, 75.4, 74.7, 74.5, 74.0, 73.9, 73.4, 72.9, 71.6, 70.9, 69.1, 68.1, 67.1, 66.8 ppm.

^{19}F NMR (564 MHz, $CDCl_3$) δ -74.87, -74.87 ppm.

HRMS (QToF): Calcd for $C_{36}H_{35}F_3O_7Na$ $[M + Na]^+$ 659.2227; found 659.2225.

NP-HPLC of **4TFA-A4** (ELSD trace, t_R (α) = 5.7 min, t_R (β) = 6.0 min):

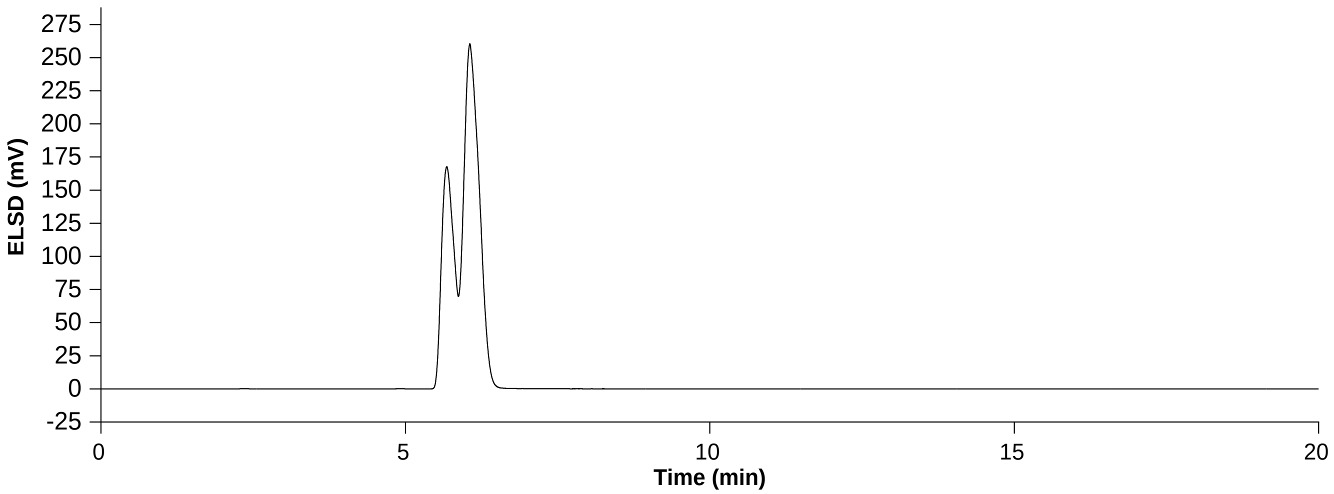

^{1}H NMR (600 MHz, $CDCl_3$) of **6TFA-A4**:

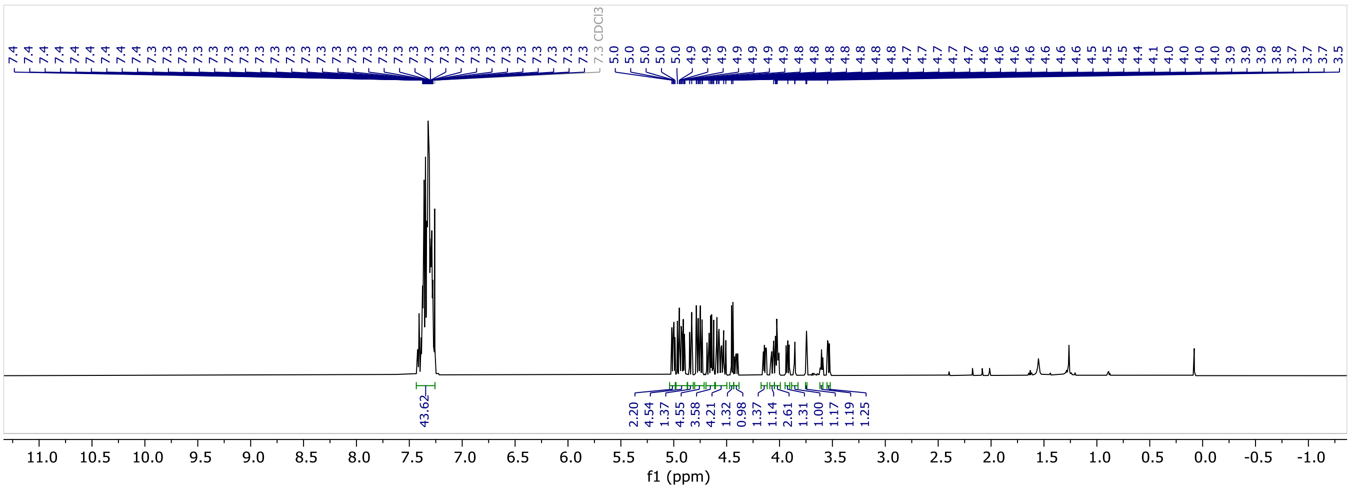

^{19}F NMR (564 MHz, $CDCl_3$) of **4TFA-A4**:

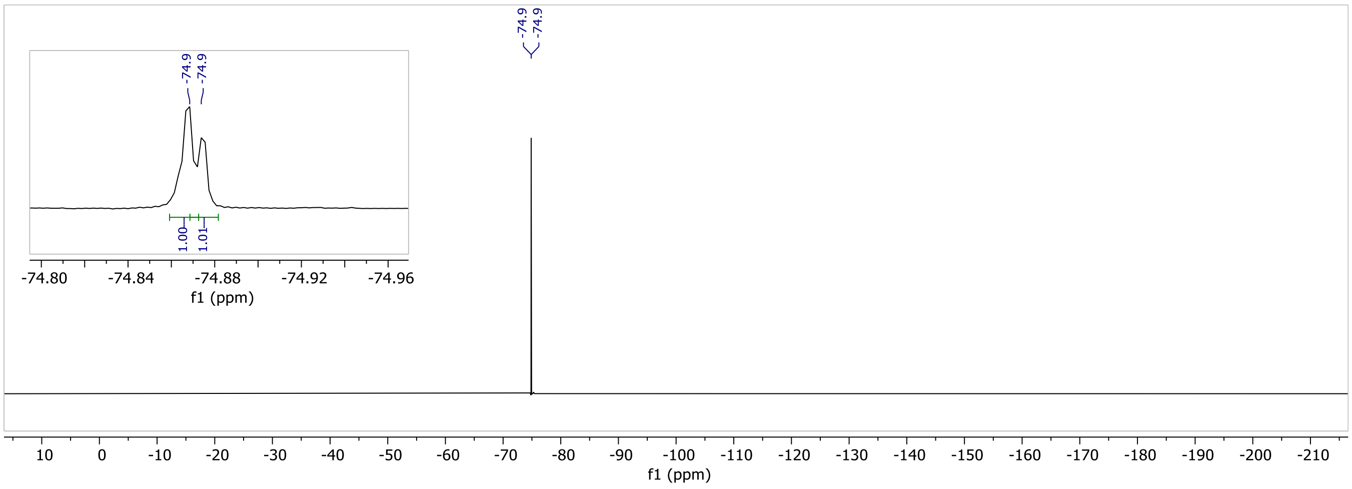

^{13}C NMR (151 MHz, $CDCl_3$) of **4TFA-A4**:

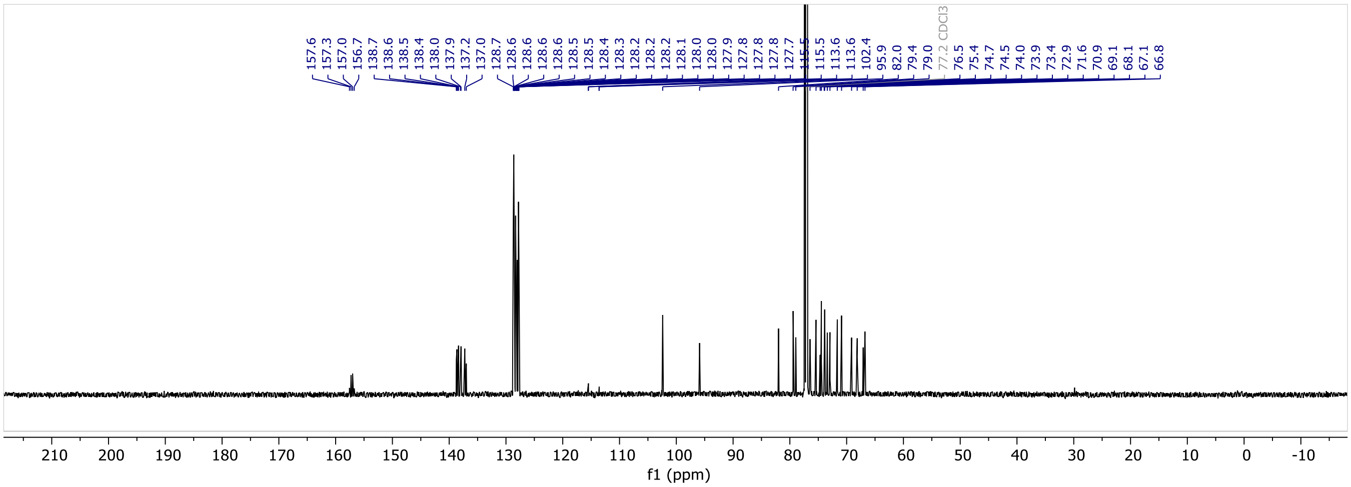

Coupled ^{13}C,^{1}H HSQC of **6TFA-A4**:

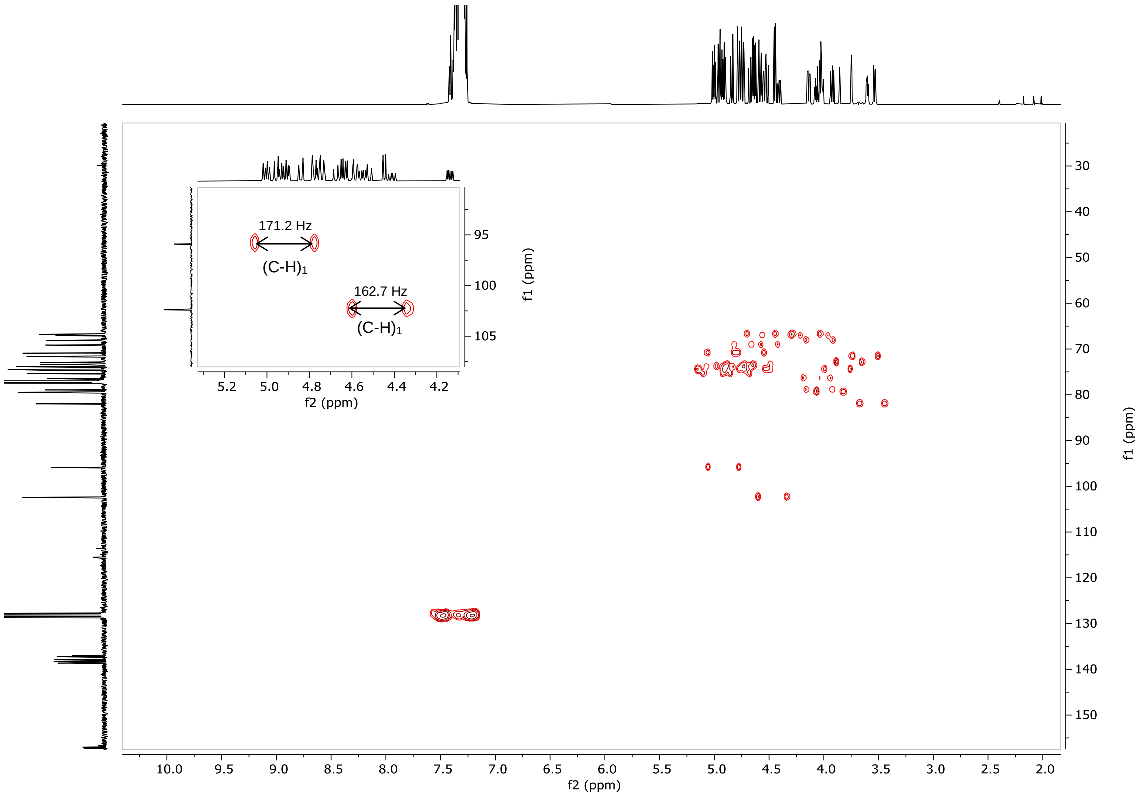

$^{13}C,^{1}H$ HSQC of **6TFA-A4**:

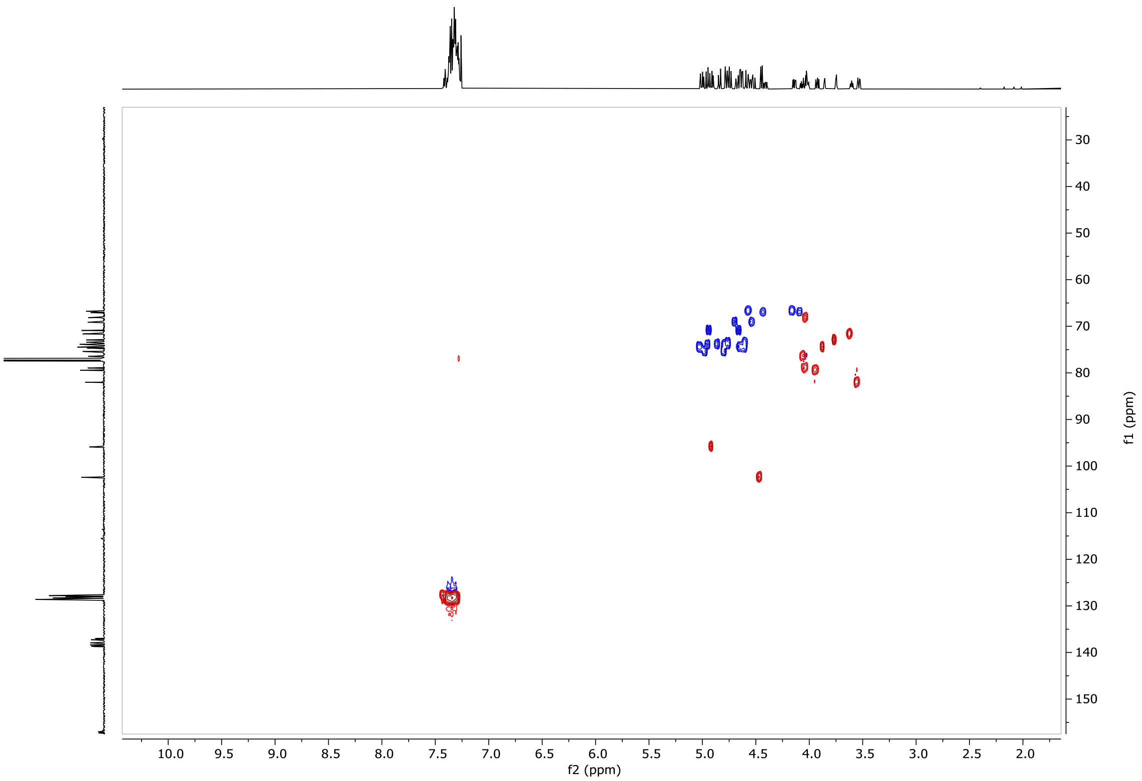

Methyl *O*-[2,3,4-tris-*O*-benzyl-6-(2,2-dimethylpropanoate)-α/β-D-galactopyranosyl]-(1→4)-2,3,6-tri-*O*-benzyl-β-D-glucopyranoside (6Piv-A2)

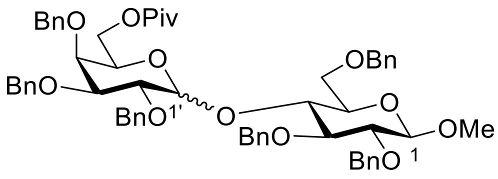

The title compound was prepared according to general procedure for glycosylations. Product **6Piv-A2** (28 mg, 28 μmol, 81%, **α/β 74:26**) was obtained as a colorless oil after purification using **Method-2b** (t_R (α) = 10.7 min, t_R (β) = 10.7 min).

Data of the major isomer (α):

^{1}**H NMR** (700 MHz, $CDCl_3$) δ 7.38 – 7.27 (m, 25H), 7.20 – 7.16 (m, 5H), 5.71 (d, *J* = 3.8 Hz, 1H, **H$_{1'}$**), 4.94 – 4.86 (m, 3H), 4.81 – 4.72 (m, 3H), 4.69 – 4.54 (m, 6H), 4.50 (d, *J* = 12.3 Hz, 1H), 4.36 (d, *J* = 7.7 Hz, 1H, **H$_1$**), 4.18 (dd, *J* = 11.1, 7.2 Hz, 1H), 4.00 (d, *J* = 3.8 Hz, 2H), 3.90 – 3.66 (m, 6H), 3.63 (ddd, *J* = 9.8, 5.9, 2.4 Hz, 1H), 3.60 (s, 3H), 3.45 (dd, *J* = 9.1, 7.7 Hz, 1H), 1.21 (s, 9H) ppm.

^{13}C NMR (176 MHz, $CDCl_3$) δ 178.1, 138.8, 138.7, 138.5, 138.4, 138.3, 138.3, 128.5, 128.5, 128.5, 128.4, 128.4, 128.3, 128.2, 128.0, 127.9, 127.7, 127.6, 127.6, 126.8, 104.6 ($\mathbf{C_1}$), 97.2 ($\mathbf{C_{1}'}$), 84.8, 82.5, 78.9, 75.7, 75.1, 74.8, 74.7, 74.6, 74.1, 73.8, 73.4, 73.3, 70.2, 69.1, 63.5, 57.1, 38.9, 27.5 ppm.

HRMS (QToF): Calcd for $C_{60}H_{68}O_{12}Na$ $[M + Na]^+$ 1003.4603; found 1003.4612.

NP-HPLC of **6Piv-A2** (ELSD trace, t_R (α) = 10.7 min, t_R (β) = 10.7 min):

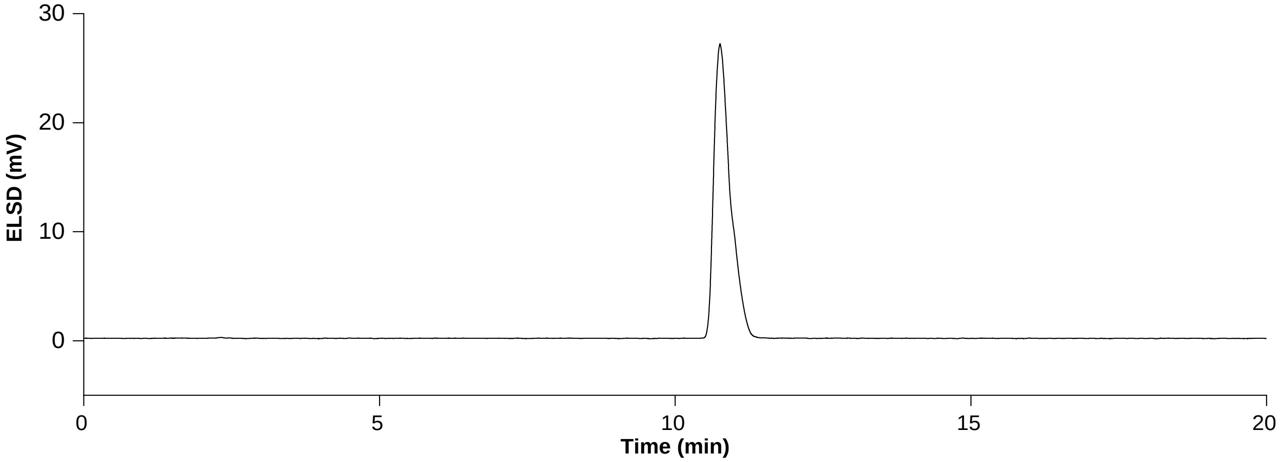

^{1}H NMR (700 MHz, $CDCl_3$) of **6Piv-A2**:

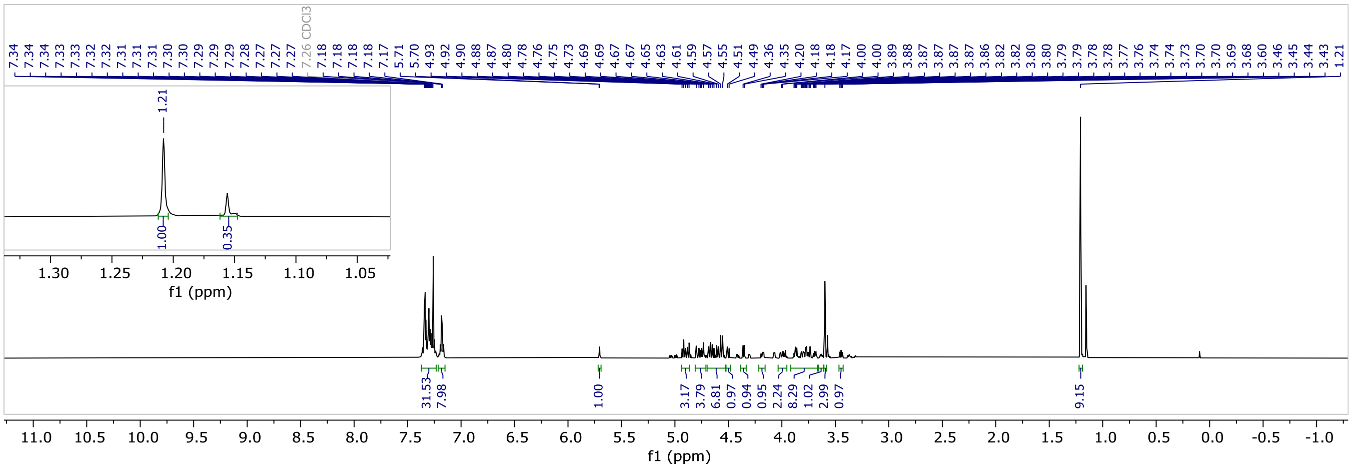

^{13}C NMR (176 MHz, $CDCl_3$) of **6Piv-A2**:

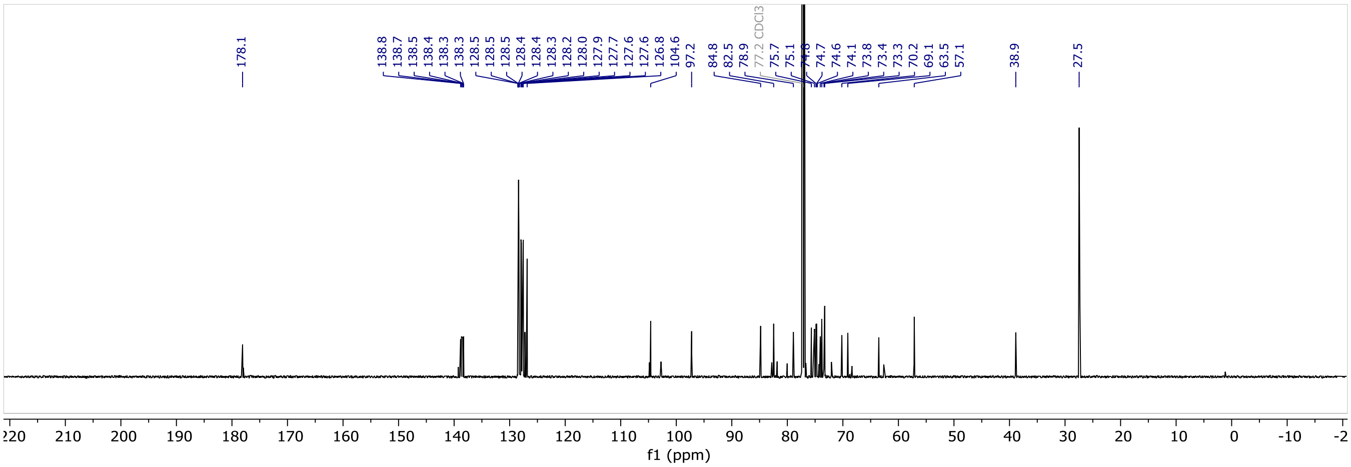

^{13}C,^{1}H HSQC of **6Piv-A2**:

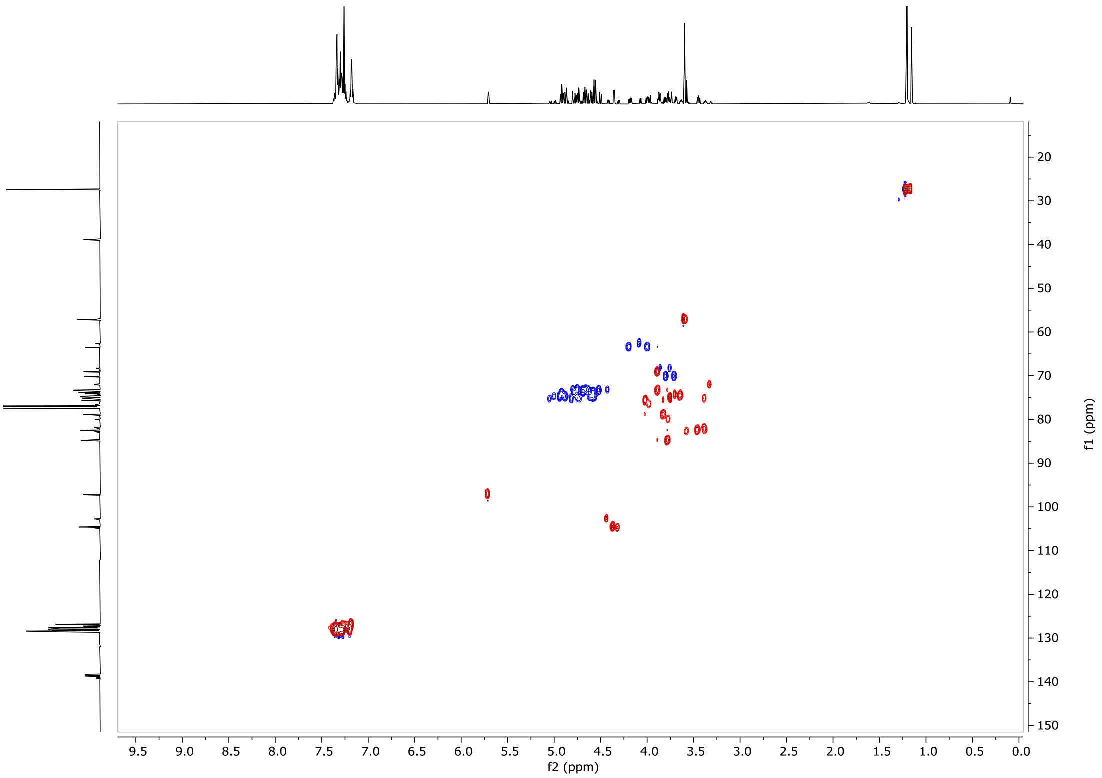

Coupled $^{13}C,^{1}H$ HSQC of **6Piv-A2**:

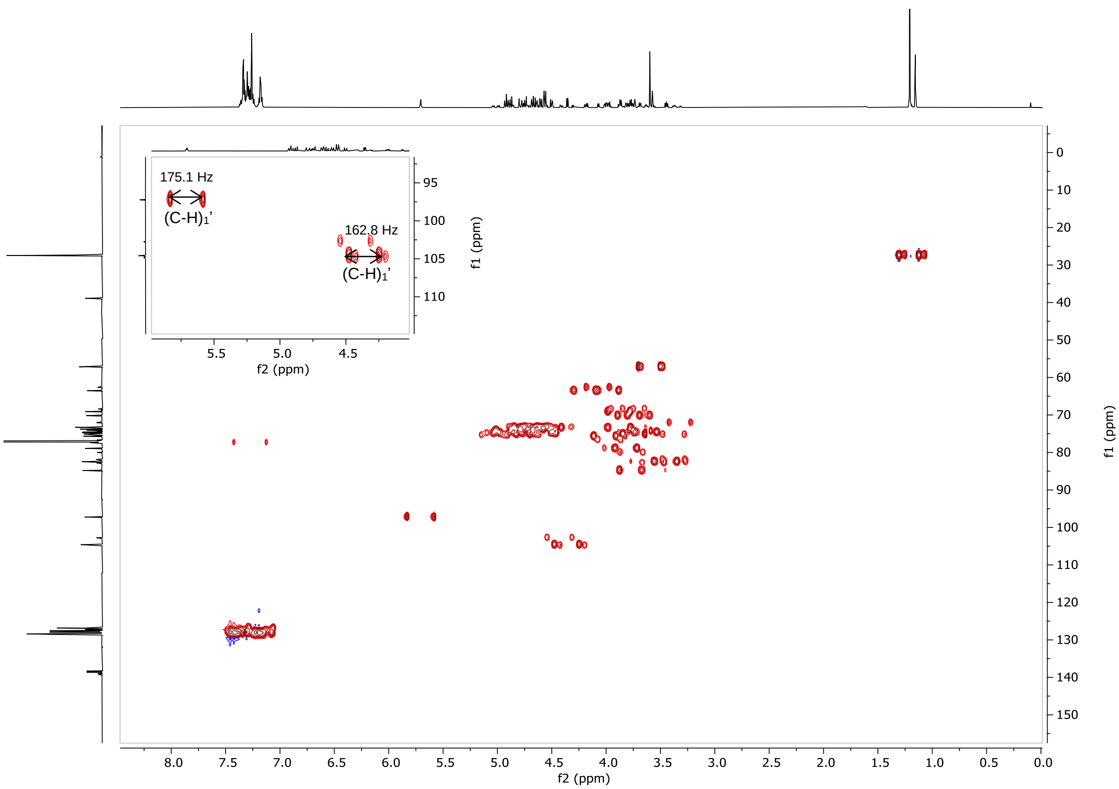

Methyl *O*-[2,3-bis-*O*-benzyl-4,6-bis-(2,2-dimethylpropanoate)-α/β-D-galactopyranosyl]-(1→4)-2,3,6-tri-*O*-benzyl-β-D-glucopyranoside (4,6Piv-A2)

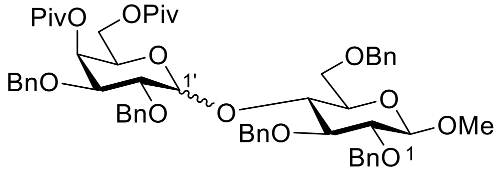

The title compound was prepared according to general procedure for glycosylations. Product **4,6Piv-A2** (26 mg, 27 μmol, 76%, **α/β 89:11**) was obtained as a colorless oil after purification using **Method-2b** (t_R (α) = 9.6 min, t_R (β) = 9.6 min).

Data of the major isomer (α):

^{1}**H NMR** (700 MHz, $CDCl_3$) δ 7.43 – 7.17 (m, 20H), 7.16 – 7.01 (m, 5H), 5.65 (d, J = 3.7 Hz, 1H, **H$_{1'}$**), 5.39 (dd, J = 3.3, 1.7 Hz, 1H), 4.95 – 4.82 (m, 2H), 4.79 (d, J = 11.7 Hz, 1H), 4.67 (d, J = 12.3 Hz, 1H), 4.61 (dd, J = 11.1, 4.9 Hz, 2H), 4.57 (d, J = 11.0 Hz, 1H), 4.51 (t, J = 11.7 Hz, 2H), 4.38 – 4.31 (m, 2H, **H$_1$**), 4.07 (td, J =

6.9, 1.8 Hz, 1H), 3.95 – 3.92 (m, 2H), 3.80 (dd, J = 10.3, 3.3 Hz, 1H), 3.77 – 3.72 (m, 2H), 3.69 (dd, J = 10.7, 5.1 Hz, 1H), 3.65 – 3.59 (m, 1H), 3.57 (s, 3H), 3.46 – 3.39 (m, 1H), 1.20 (s, 9H), 1.10 (s, 9H) ppm.

^{13}C NMR (176 MHz, $CDCl_3$) δ 178.0, 177.5, 139.0, 138.5, 138.2, 138.2, 138.0, 128.6, 128.4, 128.4, 128.3, 128.2, 128.2, 128.1, 128.0, 127.9, 127.7, 127.6, 127.6, 127.2, 126.7, 104.7 (**C_1**), 97.3 (**C_1'**), 84.8, 82.5, 76.1, 74.8, 74.4, 74.3, 74.0, 73.8, 73.5, 73.3, 72.0, 69.8, 67.3, 66.9, 62.3, 57.2, 39.1, 38.9, 27.4, 27.3 ppm.

HRMS (QToF): Calcd for $C_{58}H_{70}O_{13}Na$ [M + Na]$^+$ 997.4709; found 997.4714.

NP-HPLC of **4,6Piv-A2** (ELSD trace, t_R (α) = 9.6 min, t_R (β) = 9.6 min):

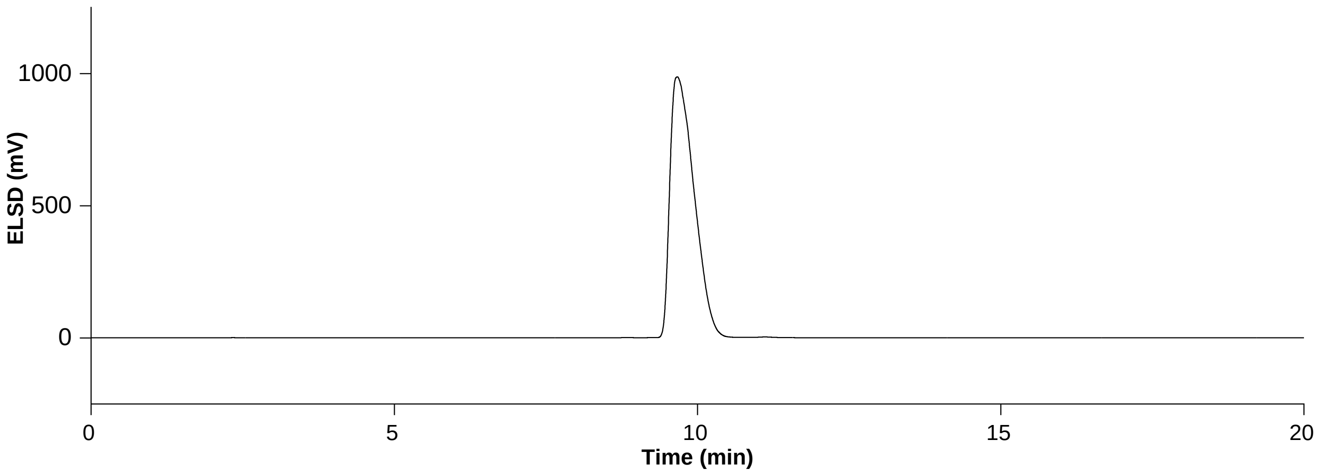

^{1}H NMR (700 MHz, $CDCl_3$) of **4,6Piv-A2**:

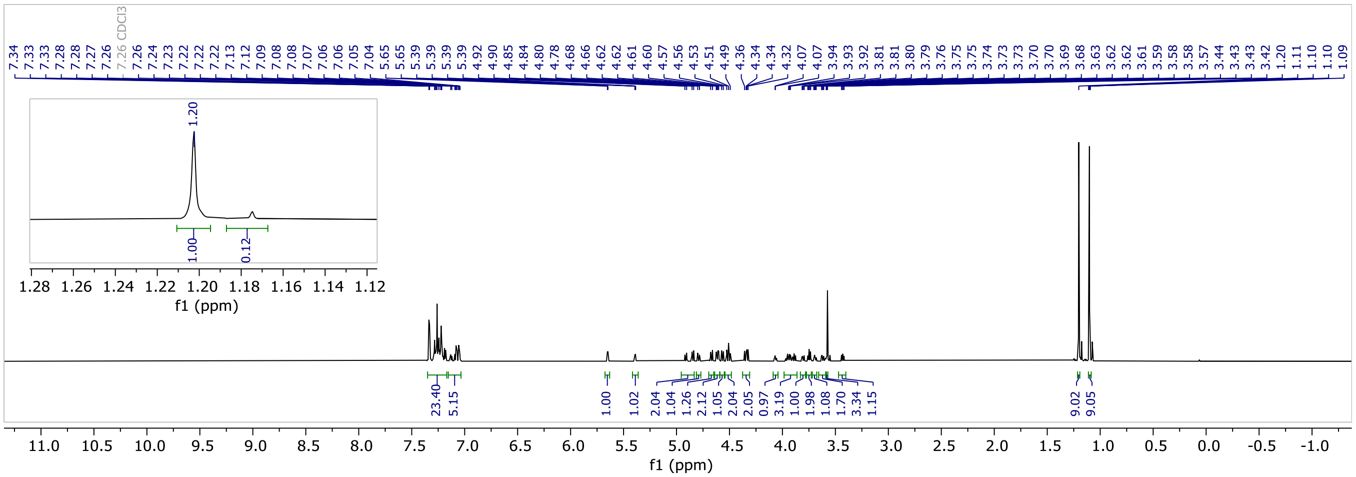

^{13}C NMR (176 MHz, $CDCl_3$) of **4,6Piv-A2**:

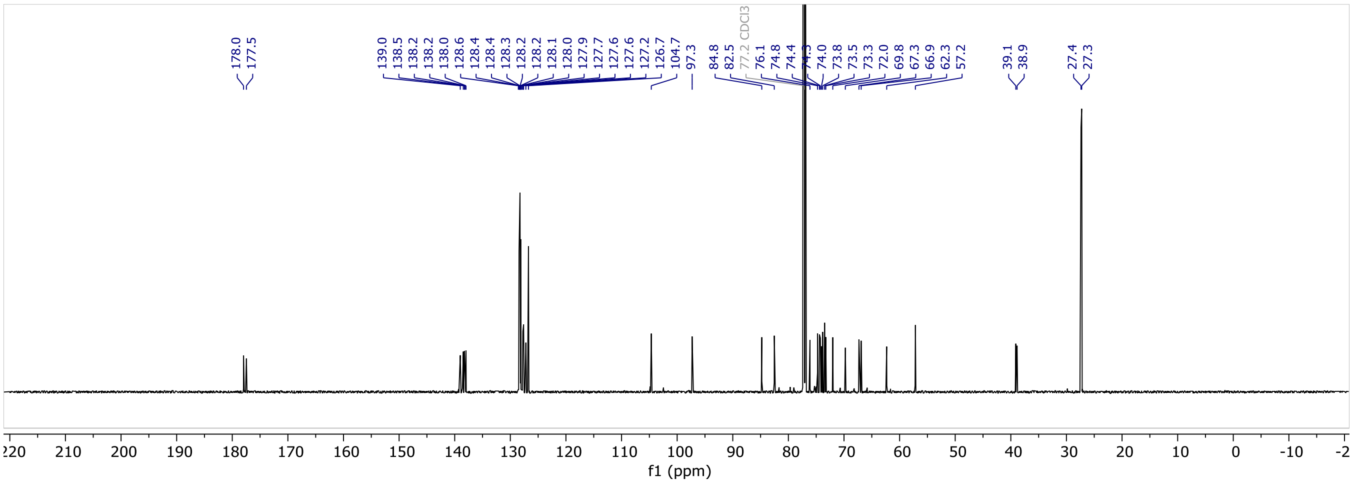

^{13}C,^{1}H HSQC of **4,6Piv-A2**:

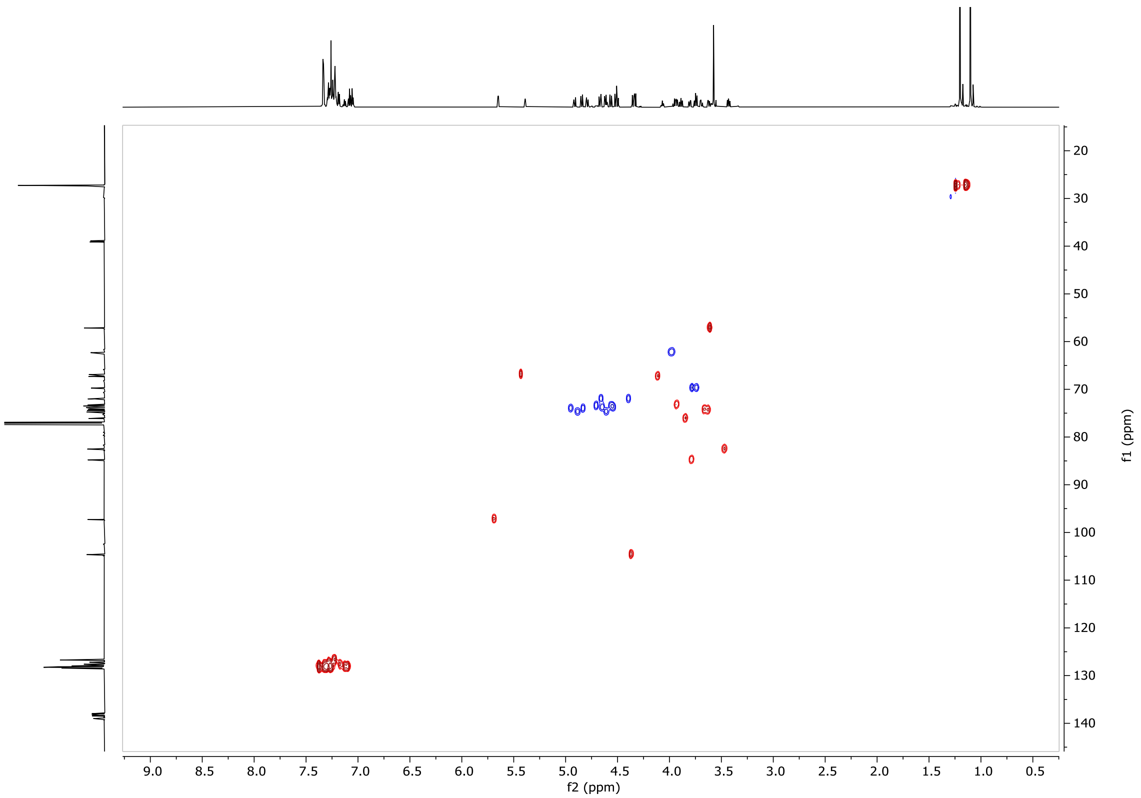

Coupled $^{13}C,^{1}H$ HSQC of **4,6Piv-A2**:

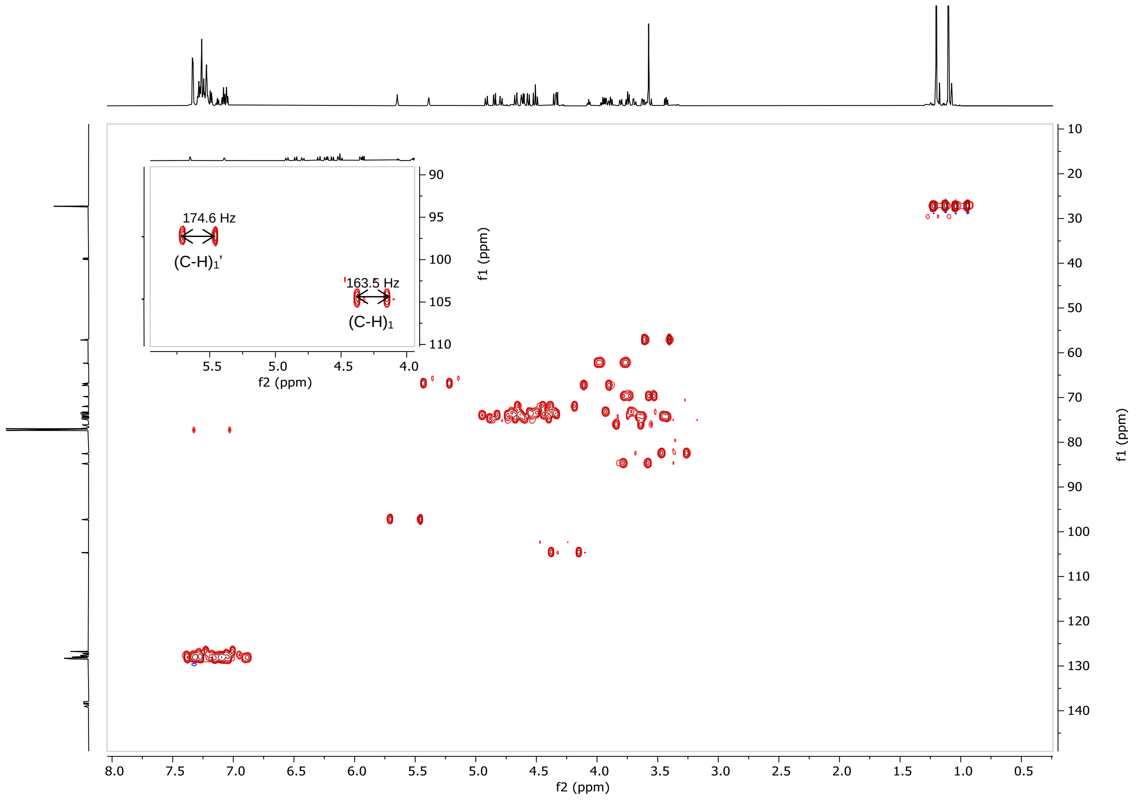

Methyl *O*-[2,3,6-tris-*O*-benzyl-4-(2,2-dimethylpropanoate)-α/β-D-galactopyranosyl]-(1→4)-2,3,6-tri-*O*-benzyl-β-D-glucopyranoside (4Piv-A2)

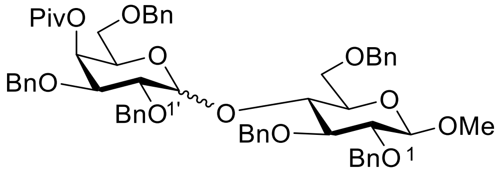

The title compound was prepared according to general procedure for glycosylations. Product **4Piv-A2** (29 mg, 30 μmol, 87%, **α/β 96:4**) was obtained as a colorless oil after purification using **Method-2b** (t_R (α) = 11.9 min, t_R (β) = 11.9 min).

Data of the major isomer (α):

^{1}H NMR (700 MHz, $CDCl_3$) δ 7.38 – 7.15 (m, 25H), 7.14 – 7.01 (m, 5H), 5.68 (d, *J* = 3.9 Hz, 1H, **H$_{1'}$**), 5.52 (dd, *J* = 3.3, 1.5 Hz, 1H), 4.92 (d, *J* = 11.8 Hz, 1H), 4.83 (dd, *J* = 21.5, 11.3 Hz, 2H), 4.66 – 4.59 (m, 3H), 4.57 (d, *J* = 11.0 Hz, 1H), 4.48 (dd, *J* = 12.0, 5.9 Hz, 2H), 4.41 (d, *J* = 11.7 Hz, 1H), 4.33 (d, *J* = 2.7 Hz, 1H), 4.32 (s, 1H, **H$_1$**), 4.29 (d, *J* = 11.7 Hz, 1H), 4.03 (t, *J* = 6.5 Hz, 1H), 3.96 (t, *J* = 9.1 Hz, 1H), 3.80 (dd, *J* = 10.2, 3.2

Hz, 1H), 3.79 – 3.74 (m, 2H), 3.71 (dd, *J* = 10.8, 5.0 Hz, 1H), 3.63 (dd, *J* = 10.2, 3.9 Hz, 1H), 3.59 (td, *J* = 4.8, 2.5 Hz, 1H), 3.57 (s, 3H), 3.45 (dd, *J* = 9.1, 7.6 Hz, 1H), 3.41 – 3.35 (m, 1H), 3.31 (dd, *J* = 9.1, 7.0 Hz, 1H), 1.07 (s, 9H) ppm.

^{13}C NMR (176 MHz, $CDCl_3$) δ 177.5, 139.2, 138.5, 138.5, 138.3, 138.1, 138.0, 128.5, 128.5, 128.4, 128.3, 128.3, 128.2, 128.1, 128.1, 127.9, 127.9, 127.7, 127.7, 127.6, 127.5, 127.1, 126.7, 104.6 (**C_1**), 97.7(**$C_{1'}$**), 84.9, 82.6, 76.6, 74.8, 74.5, 74.4, 74.0, 73.9, 73.8, 73.4, 73.3, 71.8, 69.8, 68.5, 67.3, 57.1, 39.1, 27.3 ppm.

HRMS (QToF): Calcd for $C_{60}H_{68}O_{12}Na$ [M + Na]$^+$ 1003.4603; found 1003.4614.

NP-HPLC of **4Piv-A2** (ELSD trace, t_R (α) = 11.9 min, t_R (β) = 11.9 min):

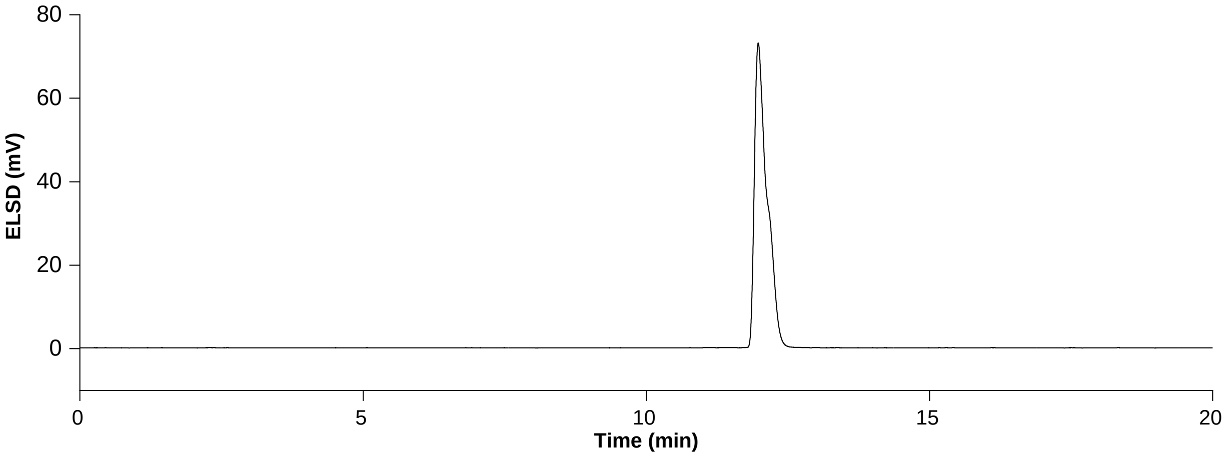

^{1}H NMR (700 MHz, $CDCl_3$) of **4Piv-A2**:

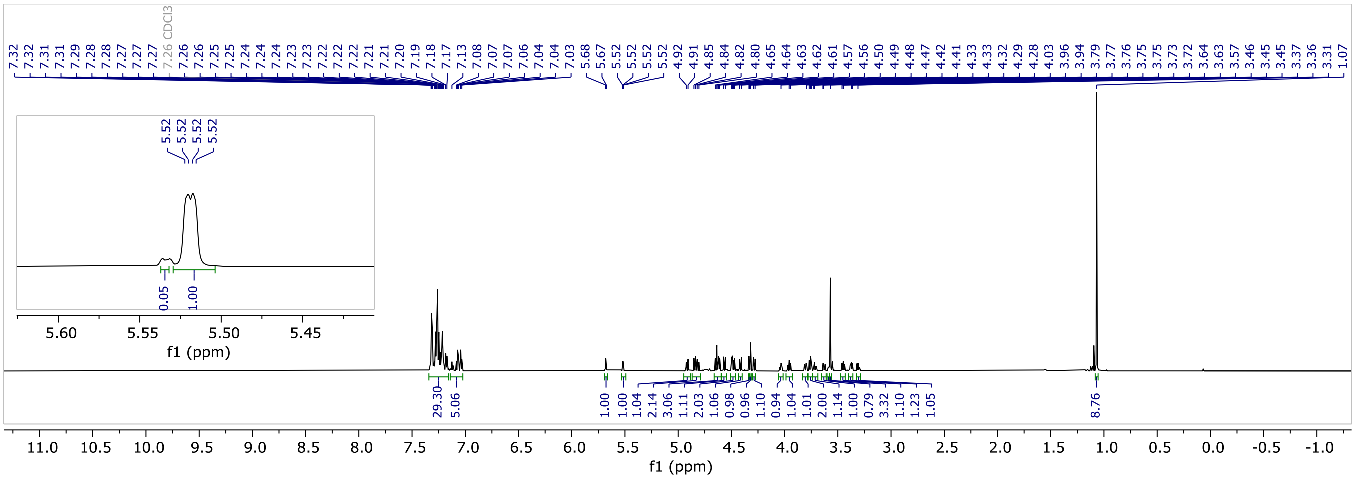

^{13}C NMR (176 MHz, CDCl$_3$) of **4Piv-A2**:

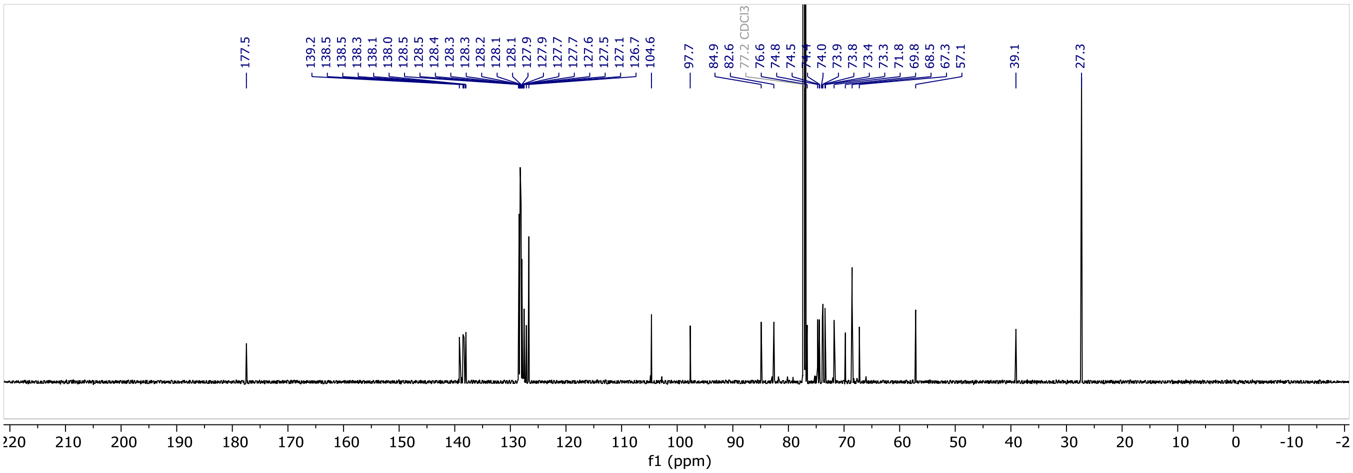

^{13}C,^{1}H HSQC of **4Piv-A2**:

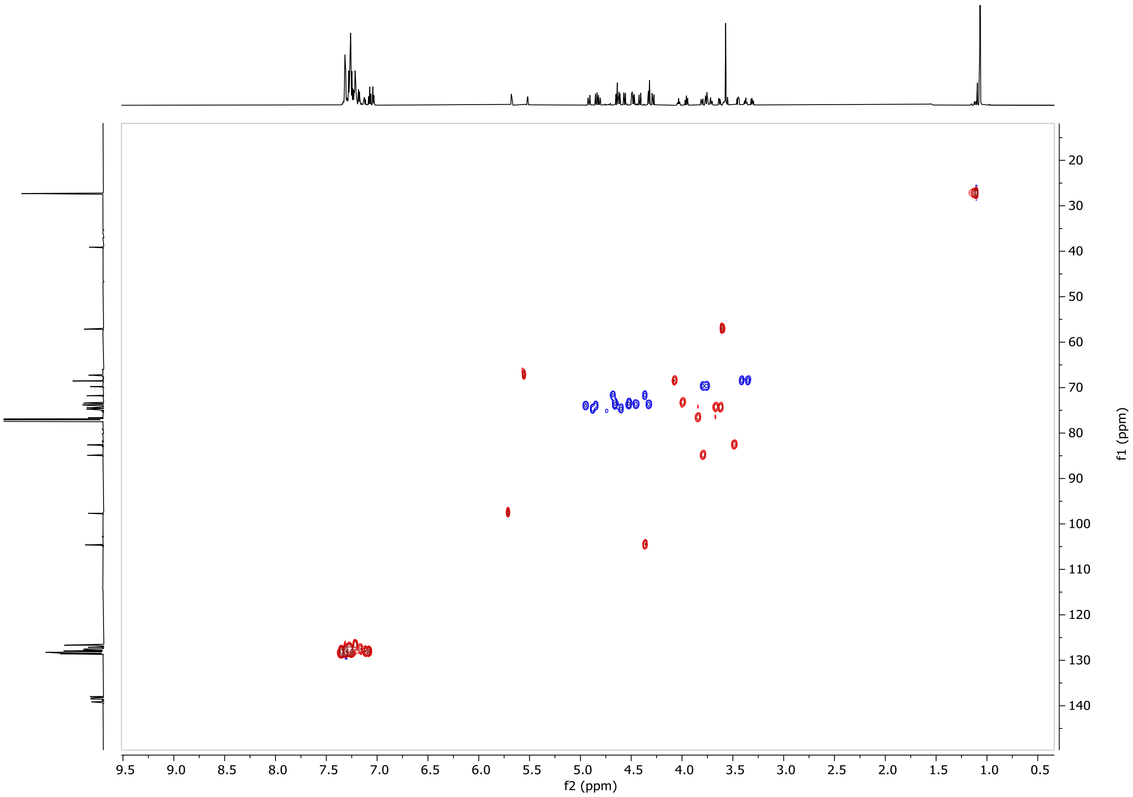

Coupled ^{13}C,^{1}H HSQC of **4Piv-A2**:

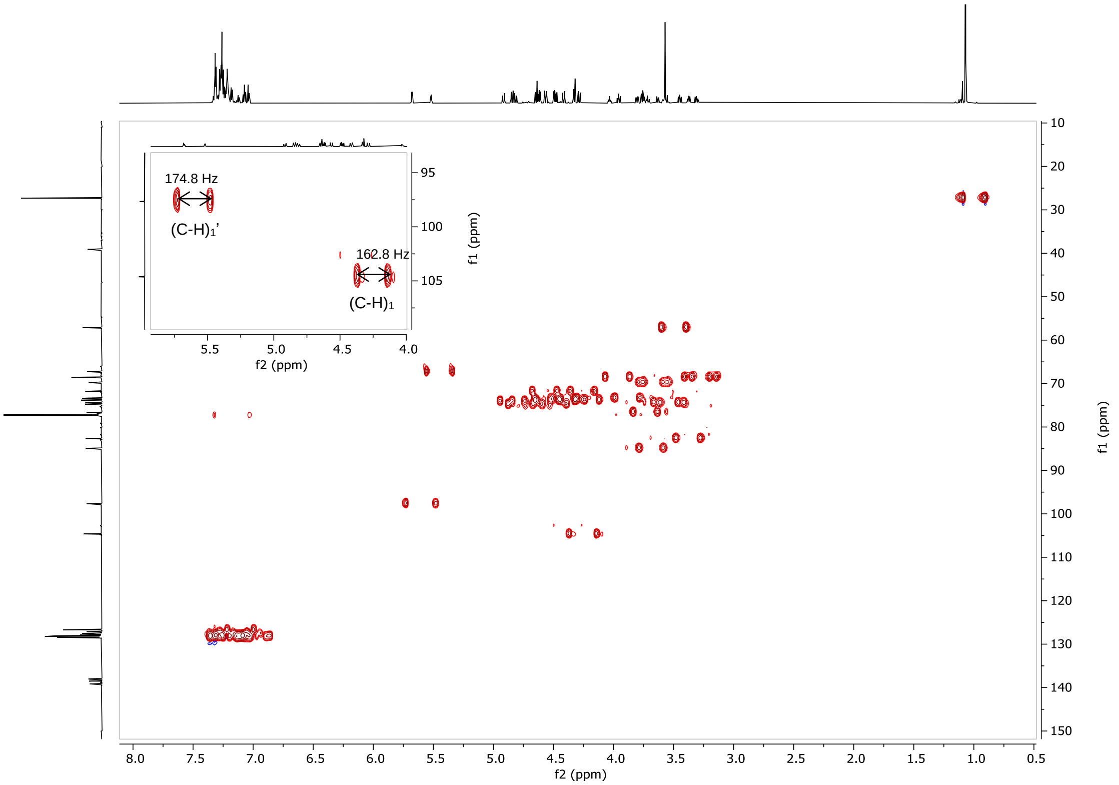

Methyl *O*-[2,3,6-tris-*O*-benzyl-4-(trifluoroacetate)-α/β-D-galactopyranosyl]-(1→4)-2,3,6-tri-*O*-benzyl-β-D-glucopyranoside (4TFA-A2)

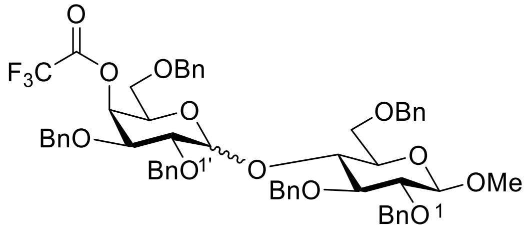

The title compound was prepared according to general procedure for glycosylations. Product **4TFA-A2** (23 mg, 23 µmol, 69%, **α/β 97:3**) was obtained as a colorless oil after purification using **Method-2b** (t_R (α) = 11.2 min, t_R (β) = 11.2 min).

Data of the major isomer (α):

^{1}H NMR (700 MHz, $CDCl_3$) δ 7.39 – 7.21 (m, 23H), 7.22 – 7.12 (m, 5H), 7.12 – 7.08 (m, 2H), 5.72 (d, *J* = 3.8 Hz, 1H, **H$_{1'}$**), 5.68 (dd, *J* = 3.3, 1.4 Hz, 1H), 4.91 (dd, *J* = 35.1, 11.3 Hz, 2H), 4.78 (d, *J* = 11.6 Hz, 1H), 4.70 – 4.57 (m, 4H), 4.56 (d, *J* = 12.0 Hz, 1H), 4.50 (d, *J* = 11.6 Hz, 1H), 4.40 (dd, *J* = 25.0, 11.2 Hz, 2H), 4.35

(d, J = 7.7 Hz, 1H), 4.26 (d, J = 11.6 Hz, 1H, **H**$_1$), 4.17 (ddd, J = 8.5, 5.4, 1.5 Hz, 1H), 4.04 (t, J = 9.1 Hz, 1H), 3.92 (dd, J = 10.2, 3.1 Hz, 1H), 3.81 – 3.71 (m, 3H), 3.67 (dd, J = 10.2, 3.8 Hz, 1H), 3.60 (s, 3H), 3.58 (ddd, J = 9.6, 4.1, 2.6 Hz, 1H), 3.48 (dd, J = 9.1, 7.7 Hz, 1H), 3.41 (dd, J = 8.8, 5.5 Hz, 1H), 3.31 (t, J = 8.7 Hz, 1H) ppm.

^{13}C NMR (176 MHz, $CDCl_3$) δ 157.00 (q, J = 42.6 Hz), 138.8, 138.4, 138.4, 137.8, 137.7, 137.5, 128.6, 128.5, 128.5, 128.4, 128.3, 128.3, 128.3, 128.2, 128.1, 128.1, 128.0, 128.0, 127.9, 127.8, 127.8, 127.7, 127.3, 126.8, 114.73 (q, J = 286.3 Hz), 104.7 (**C**$_1$), 97.3 (**C**$_{1}$**'**), 84.9, 82.6, 76.0, 74.8, 74.4, 74.3, 74.2, 73.8, 73.6, 72.8, 72.5, 72.4, 69.5, 67.3, 67.1, 57.1 ppm.

^{19}F NMR (659 MHz, $CDCl_3$) δ -74.79 (s) ppm.

HRMS (QToF): Calcd for $C_{57}H_{59}F_3O_{12}Na$ $[M + Na]^+$ 1015.3851; found 1015.3853.

NP-HPLC of **4TFA-A2** (ELSD trace, t_R (α) = 11.2 min, t_R (β) = 11.2 min):

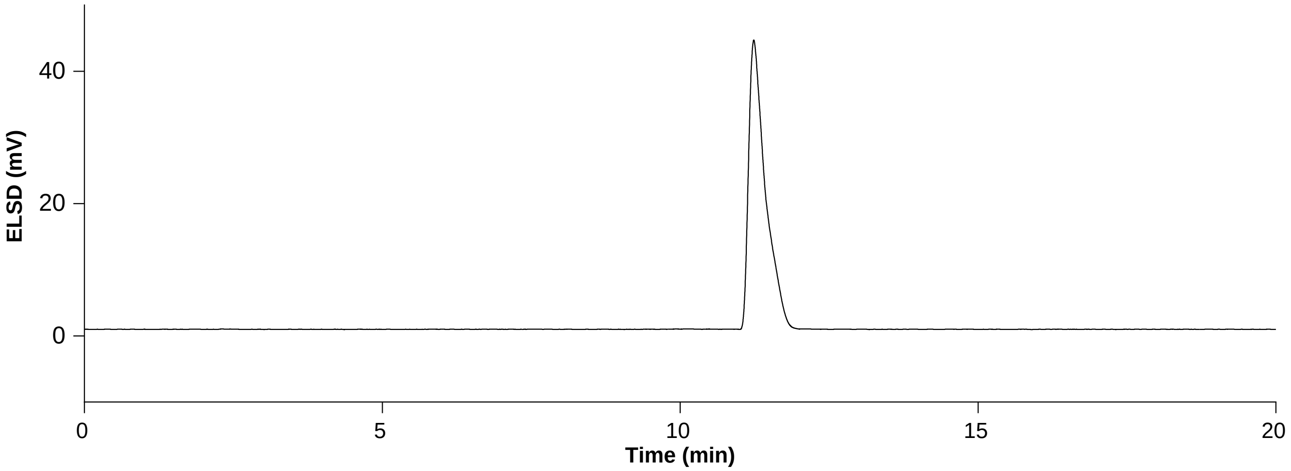

^{1}H NMR (700 MHz, $CDCl_3$) of **4TFA-A2**:

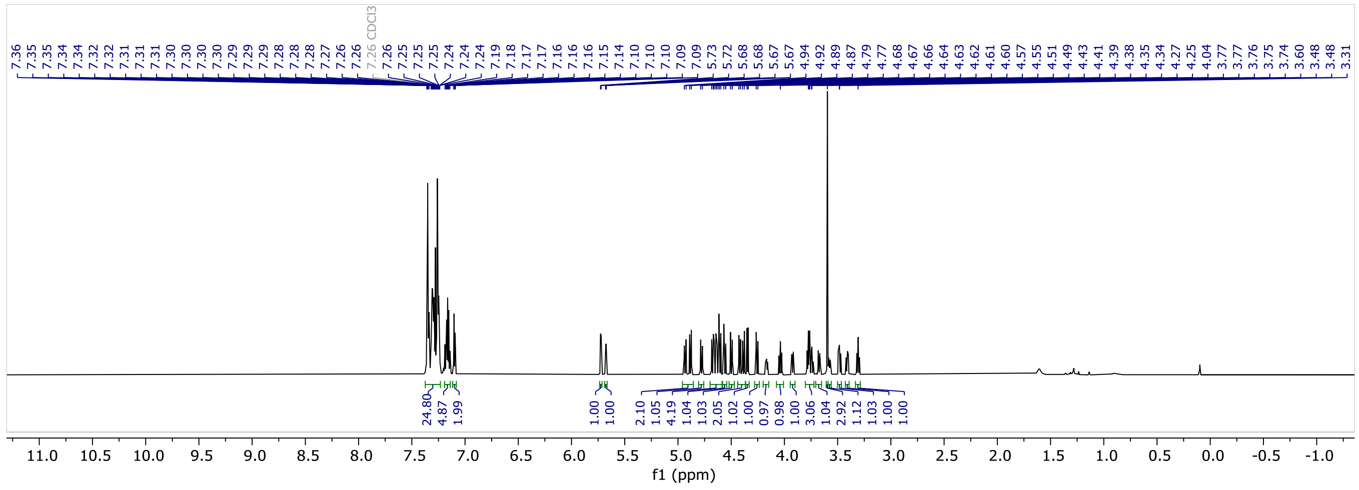

^{13}C NMR (176 MHz, $CDCl_3$) of **4TFA-A2**:

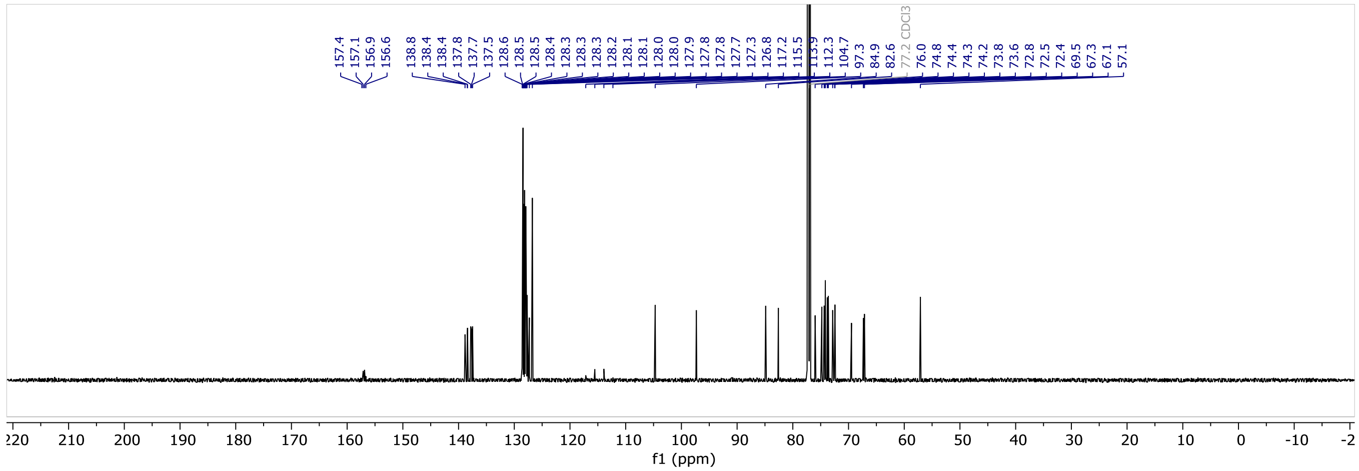

Coupled $^{13}C,^{1}H$ HSQC of **4TFA-A2**:

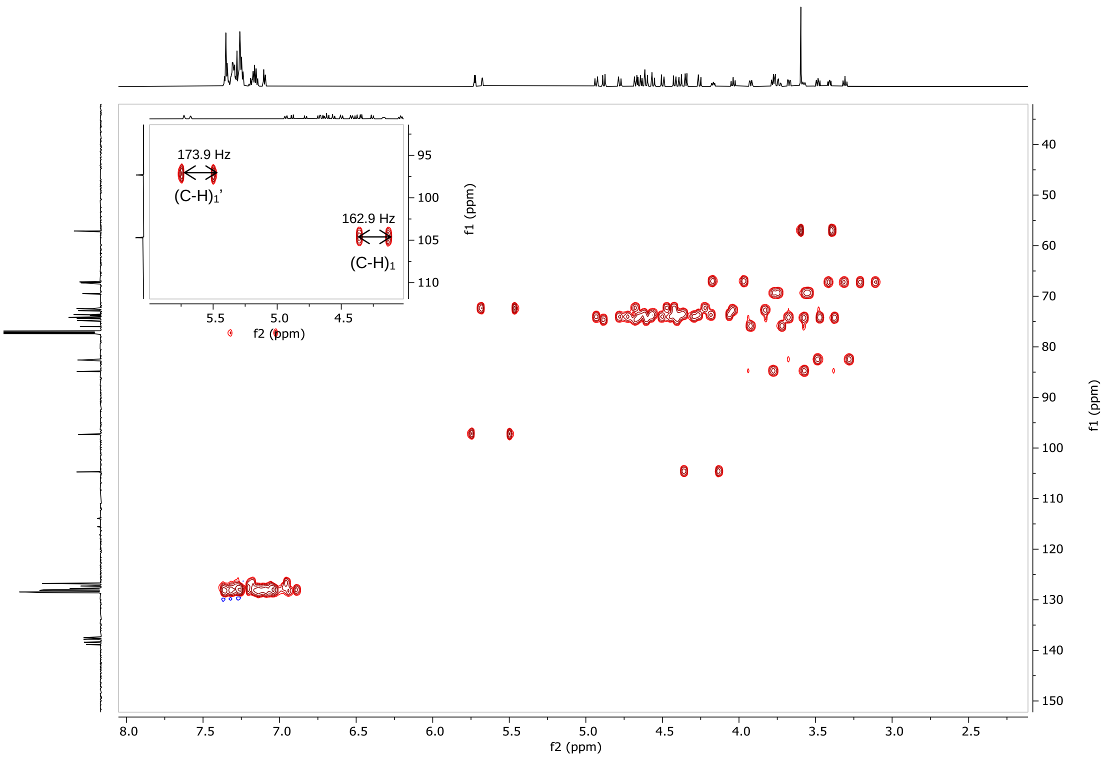

$^{13}C,^{1}H$ HSQC of **4TFA-A2**:

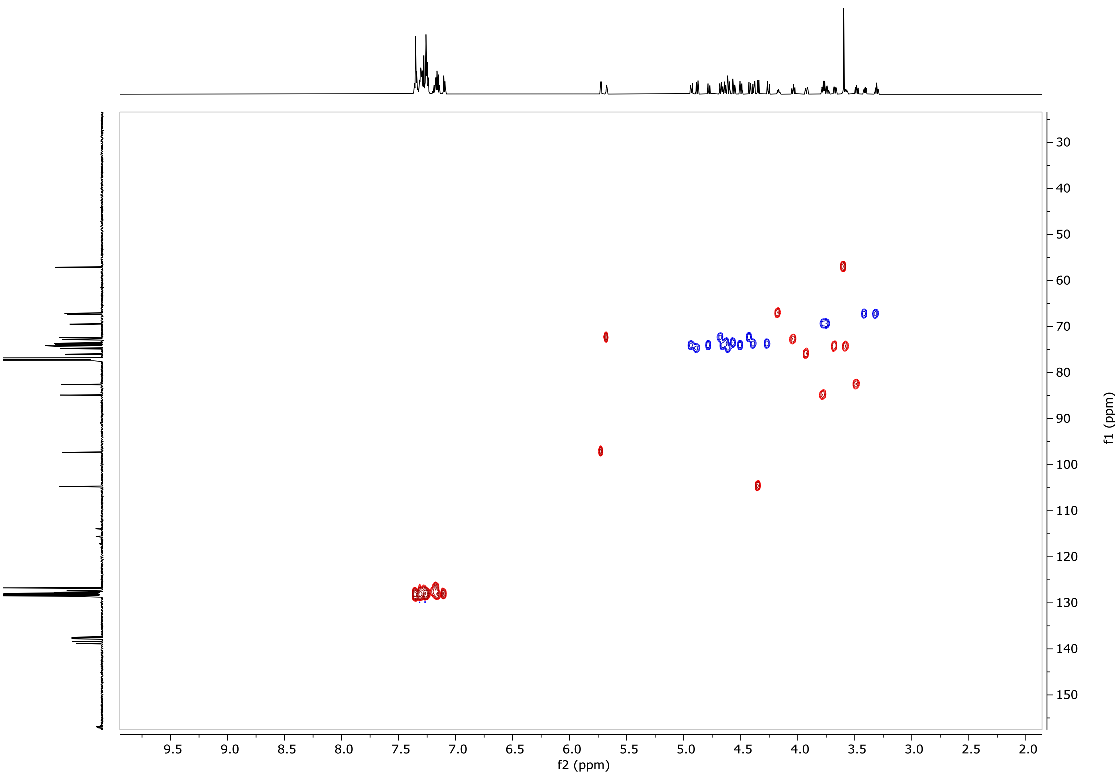

Methyl *O*-[2,3,4-tris-*O*-benzyl-6-(trifluoroacetate)-α/β-D-galactopyranosyl]-(1→4)-2,3,6-tri-*O*-benzyl-β-D-glucopyranoside (6TFA-A2)

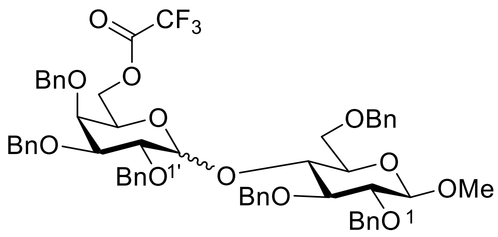

The title compound was prepared according to general procedure for glycosylations. Product **6TFA-A2** (20 mg, 20 μmol, 59%, **α/β 74:26**) was obtained as a colorless oil after purification using **Method-2b** (t_R (α) = 9.9 min, t_R (β) = 9.9 min).

Data of the major isomer (α):

^{1}H NMR (700 MHz, $CDCl_3$) δ 7.38 – 7.26 (m, 20H), 7.23 – 7.13 (m, 10H), 5.73 (d, *J* = 3.8 Hz, 1H, **$H_{1'}$**), 4.94 – 4.87 (m, 3H), 4.77 – 4.72 (m, 3H), 4.67 (dd, *J* = 15.7, 11.7 Hz, 3H), 4.60 (d, *J* = 11.1 Hz, 1H), 4.54 (dd, *J* = 14.0, 11.6 Hz, 2H), 4.48 (d, *J* = 12.3 Hz, 1H), 4.36 (d, *J* = 7.7 Hz, 1H, **H_1**), 4.32 – 4.25 (m, 1H), 3.98 (dd, *J* = 10.2, 3.9 Hz, 1H), 3.91 (dd, *J* = 11.0, 5.2 Hz, 1H), 3.88 – 3.76 (m, 4H), 3.68 – 3.64 (m, 1H), 3.60 (s, 3H), 3.46 (dd, *J* = 9.1, 7.6 Hz, 1H) ppm.

^{13}C NMR (176 MHz, $CDCl_3$) δ 156.95 (q, *J* = 42.4 Hz), 138.8, 138.5, 138.5, 138.5, 138.2, 137.9, 128.6, 128.6, 128.5, 128.4, 128.4, 128.2, 128.1, 128.0, 127.9, 127.8, 127.7, 127.7, 127.6, 127.6, 126.7, 114.55 (q, *J* = 285.5 Hz), 104.6 (**C_1**), 97.3 (**$C_{1'}$**), 84.8, 82.4, 78.7, 75.5, 74.7, 74.5, 74.5, 74.0, 73.9, 73.5, 73.4, 73.4, 69.9, 68.4, 66.9, 57.2 ppm.

^{19}F NMR (659 MHz, $CDCl_3$) δ -74.57 (s).

HRMS (QToF): Calcd for $C_{57}H_{59}F_3O_{12}Na$ $[M + Na]^+$ 1015.3851; found 1015.3860.

NP-HPLC of **6TFA-A2** (ELSD trace, t_R (α) = 9.9 min, t_R (β) = 9.9 min):

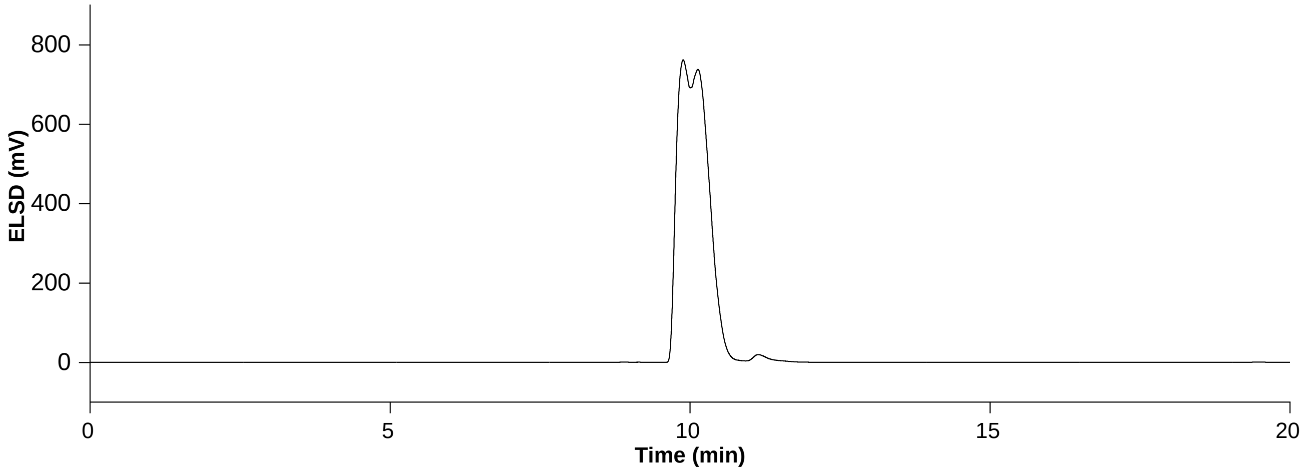

^{1}H NMR (700 MHz, $CDCl_3$) of **6TFA-A2**:

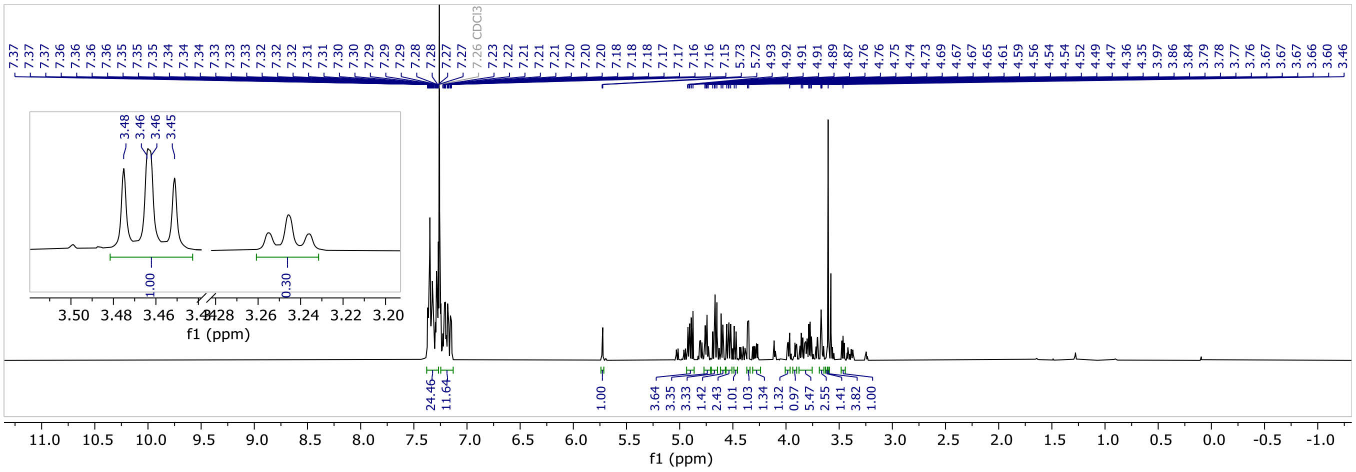

^{13}C NMR (176 MHz, $CDCl_3$) of **6TFA-A2**:

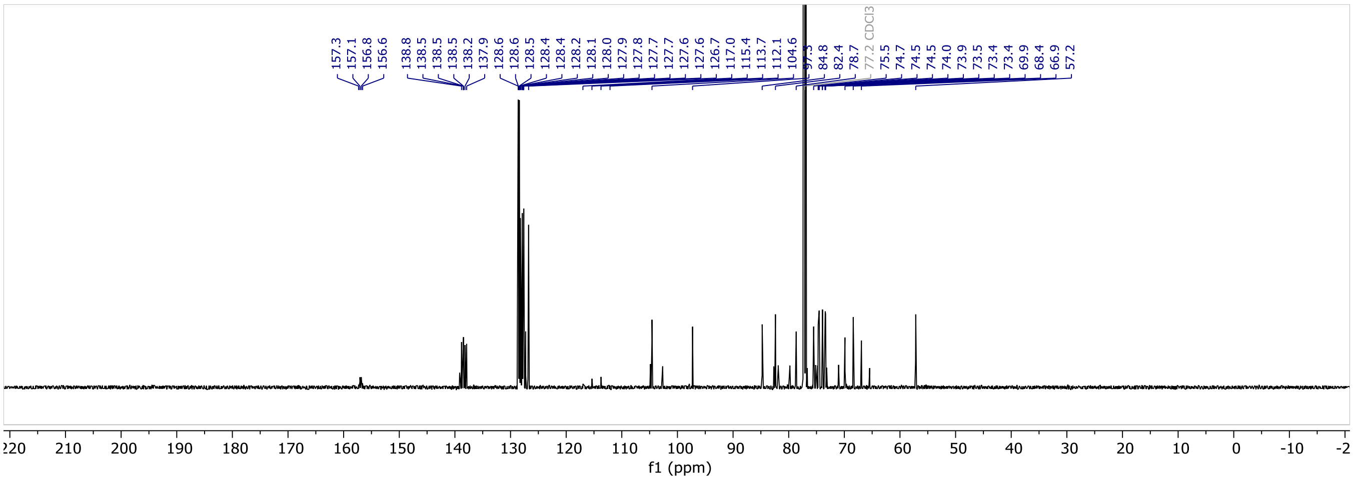

Coupled $^{13}C,^{1}H$ HSQC of **6TFA-A2**:

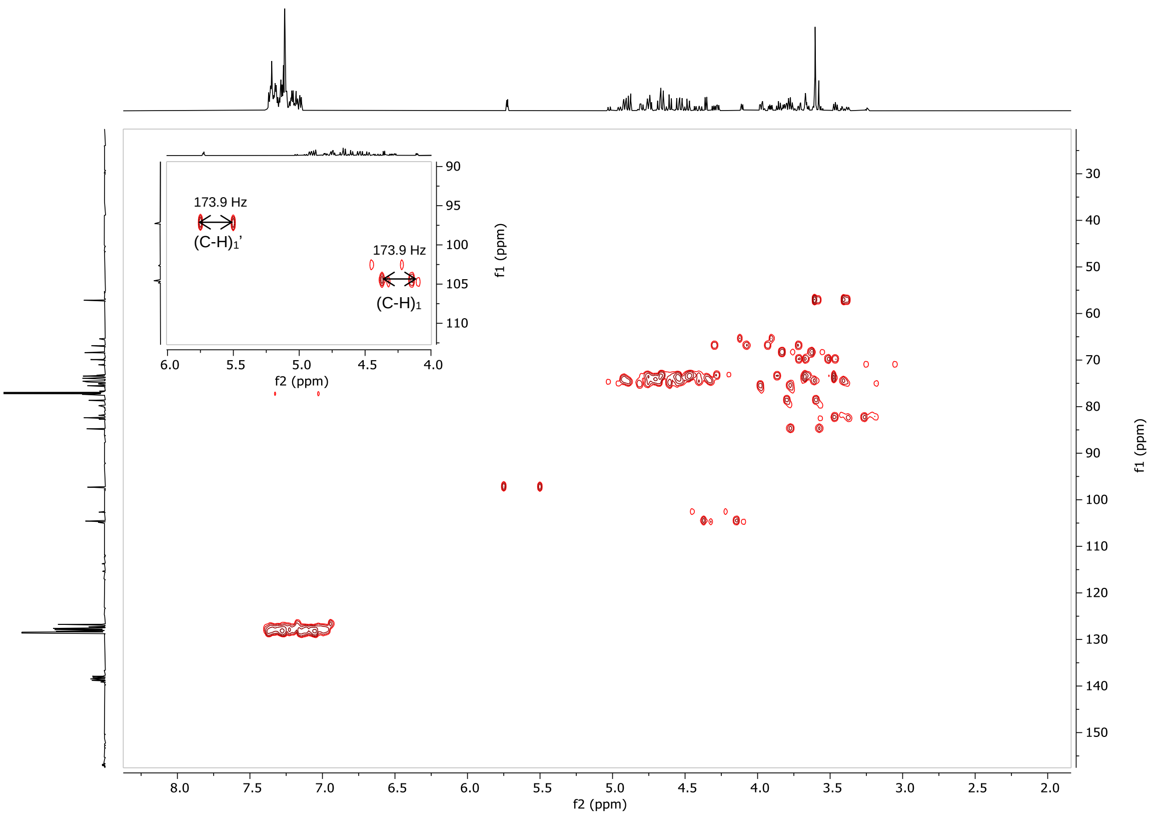

$^{13}C,^{1}H$ HSQC of **6TFA-A2**:

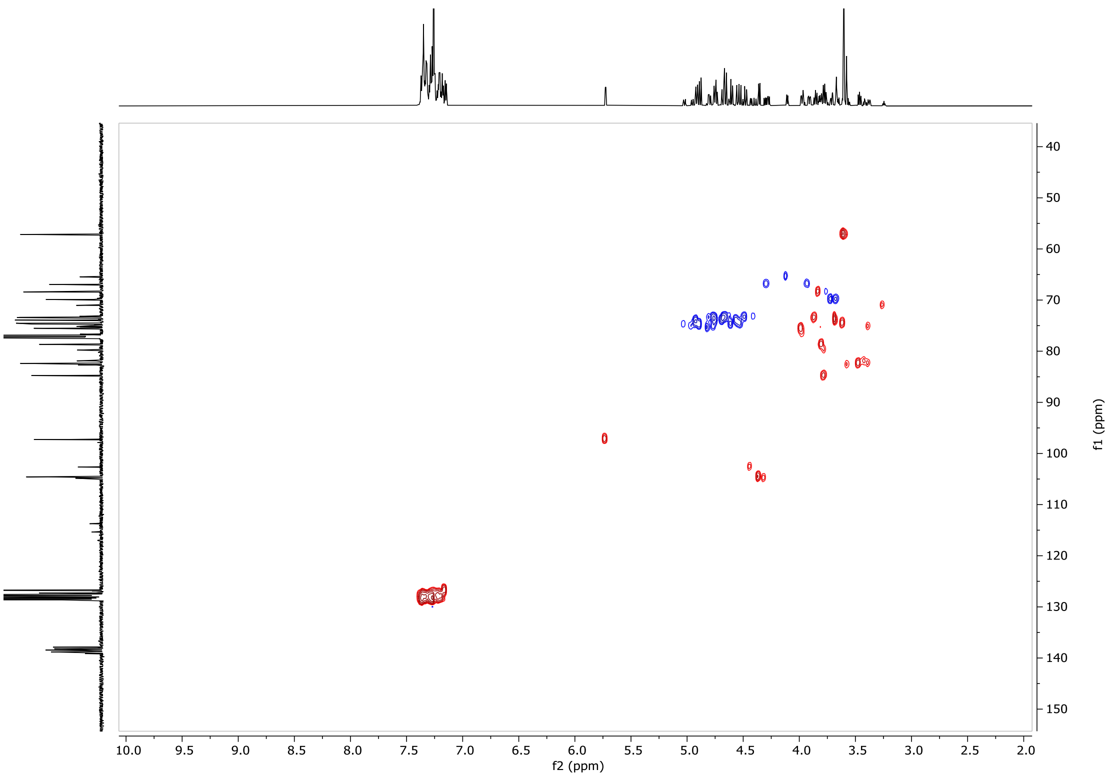

1,2:3,4-Bis-*O*-(1-methylethylidene)-6-*O*-[2,3,4,6-tetra-*O*-benzyl-α/β-D-galactopyranosyl]-α-D-galactopyranoside (4Bn-A1)

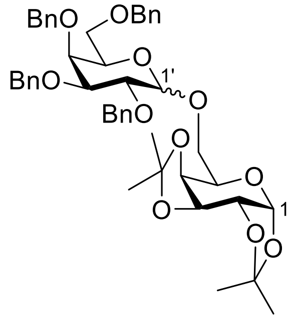

The title compound was prepared according to general procedure for glycosylations. Product **4Bn-A1** (21 mg, 27 μmol, 78%, **α/β 60:40**) was obtained as a colorless oil after purification using **Method-2b** (tR (α) = 10.4 min, tR (β) = 11.4 min).

Data of the anomeric mixture:

^{1}H NMR (600 MHz, CDCl3) δ 7.41 – 7.27 (m, 36H), 7.26 – 7.21 (m, 2H), 5.56 (d, *J* = 5.0 Hz, 1H, **H_1(α)**), 5.52 (d, *J* = 5.0 Hz, 1H, **H_1(α)**), 5.06 (d, *J* = 11.0 Hz, 1H), 5.01 (d, *J* = 3.6 Hz, 1H **H_1'(α)**), 4.94 (dd, *J* = 11.5, 3.9 Hz, 2H), 4.89 – 4.66 (m, 8H), 4.66 – 4.52 (m, 5H), 4.47 (t, *J* = 11.9 Hz, 2H), 4.45 – 4.37 (m, 4H **H_1'(β)**, CH_2Ph), 4.37 – 4.29 (m, 4H), 4.25 – 4.20 (m, 1H), 4.13 (dd, *J* = 10.7, 3.5 Hz, 1H), 4.08 – 4.01 (m, 6H), 3.96 (dd, *J* = 10.0, 2.8 Hz, 1H), 3.89 (d, *J* = 2.6 Hz, 1H), 3.81 (ddd, *J* = 23.0, 10.1, 7.1 Hz, 2H), 3.74 (dd, *J* = 10.5, 7.1 Hz, 1H), 3.69 (dd, *J* = 10.6, 7.5 Hz, 1H), 3.57 (s, *J* = 9.0, 3.4 Hz, 3H), 3.54 – 3.48 (m, 3H), 1.52 (s, 3H), 1.49 (s, 3H), 1.44 (s, 3H), 1.43 (s, 3H), 1.33 (s, 4H), 1.31 (s, 4H), 1.30 (s, 4H) ppm.

^{13}C NMR (151 MHz, $CDCl_3$) δ 139.1, 139.0, 138.8, 138.7, 138.7, 138.1, 137.9, 128.6, 128.4, 128.4, 128.4, 128.3, 128.3, 128.3, 128.3, 128.2, 128.1, 128.1, 127.9, 127.8, 127.8, 127.8, 127.7, 127.5, 127.5, 127.5, 127.4, 127.4, 127.3, 109.3, 109.2, 108.6, 108.5, 104.7(**C_1'(β)**), 97.6(**C_1'(α)**), 96.4(**C_1(α)**), 96.3(**C_1(α)**), 82.0, 79.1, 79.0, 77.3, 77.1, 76.8, 76.4, 74.9, 74.8, 74.8, 74.5, 73.6, 73.5, 73.4, 73.3, 73.1, 73.1, 72.7, 71.5, 70.9, 70.8, 70.7, 70.6, 70.5, 69.6, 69.2, 68.7, 68.7, 67.4, 66.3, 65.8, 26.2, 26.1, 26.0, 26.0, 25.1, 25.0, 24.6, 24.5 ppm.

HRMS (ESI): Calcd for $C_{46}H_{54}O_{11}Na$ $[M + Na]^+$ 805.3564; found 805.3688.

NP-HPLC of **4Bn-A1** (ELSD trace, *t*R (α) = 10.4 min, *t*R (β) = 11.4 min):

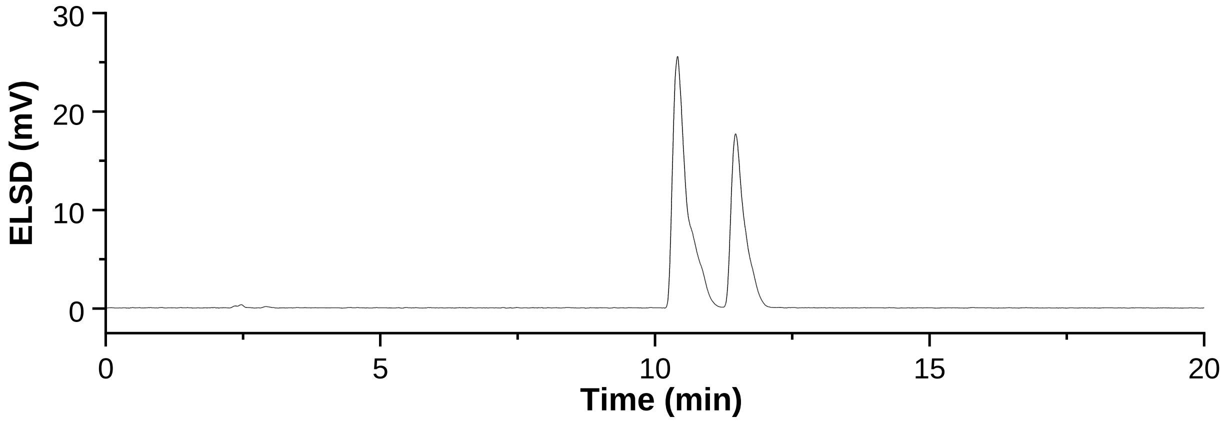

^{1}H NMR (600 MHz, $CDCl_3$) of **4Bn-A1**:

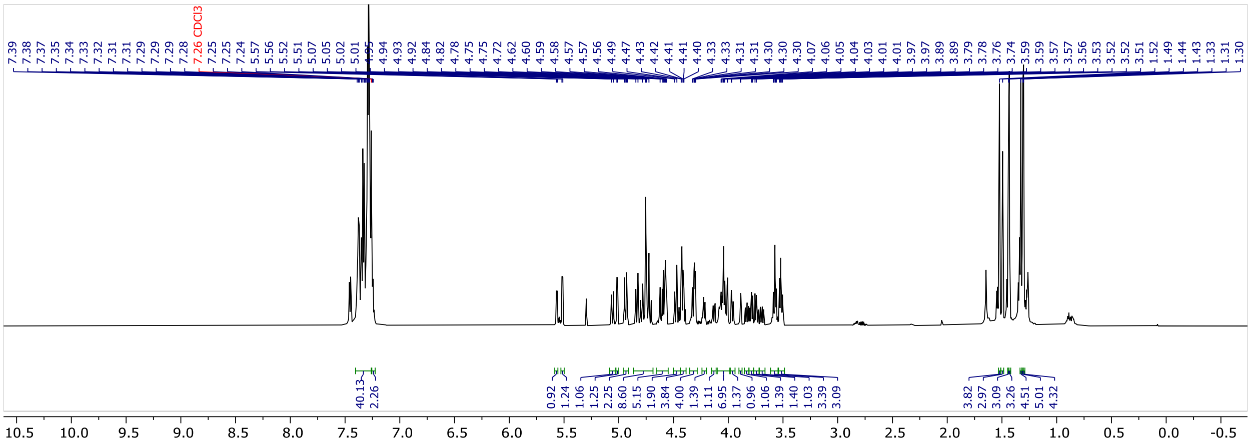

^{13}C NMR (151 MHz, $CDCl_3$) of **4Bn-A1**:

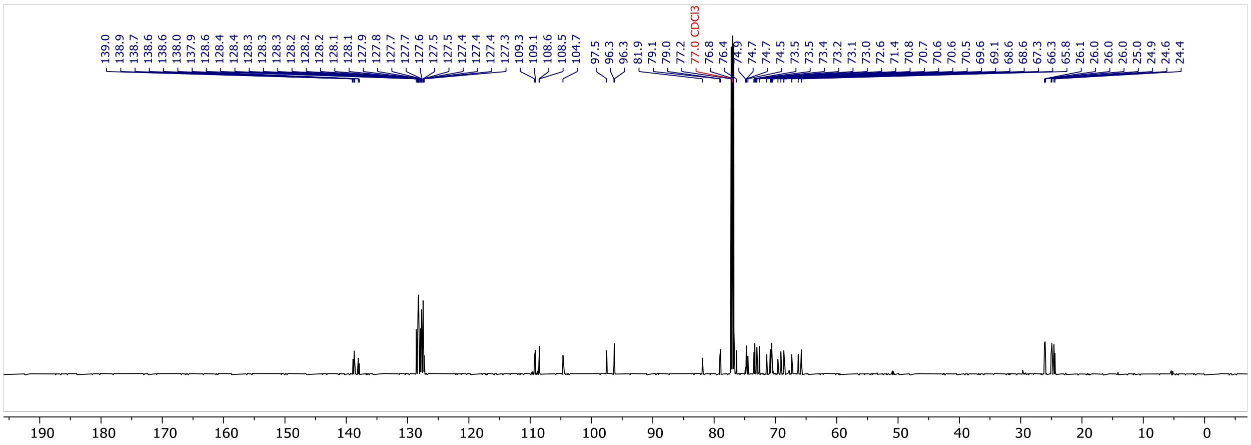

^{13}C,^{1}H HSQC of **4Bn-A1**:

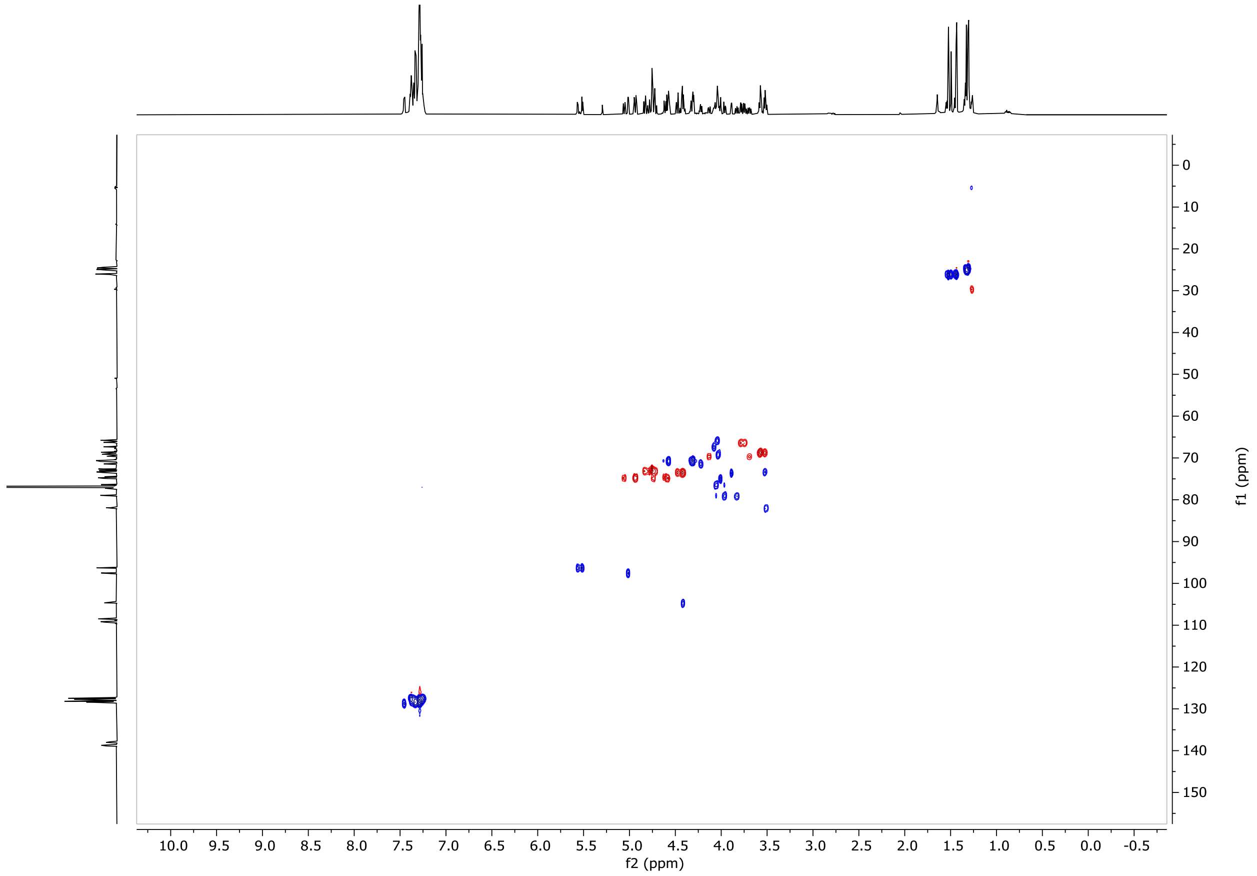

Methyl *O*-[2,3,4,6-tetra-*O*-benzyl-α/β-D-galactopyranosyl]-(1→4)-2,3,6-tri-*O*-benzyl-β-D-glucopyranoside (4Bn-A2)

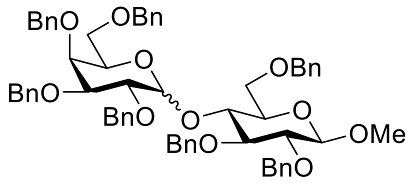

The title compound was prepared according to general procedure for glycosylations. Product **4Bn-A2** (27 mg, 28 μmol, 79%, **α/β 84:16**) was obtained as a colorless oil after purification using **Method-2b** (*t*R (α) = 11.9 min, tR (β) = 12.2 min).

Data of the α-anomer (major product):

^{1}H NMR (600 MHz, $CDCl_3$) δ 7.44 – 7.21 (m, 30H), 7.14 (m, 5H), 5.73 (d, *J* = 3.9 Hz, 1H, **H_{1}'(α)**), 4.91 (d, *J* = 11.5 Hz, 1H), 4.86 (dd, *J* = 11.2, 2.0 Hz, 2H), 4.76 (d, *J* = 11.6 Hz, 1H), 4.69 – 4.56 (m, 6H), 4.53 (d, *J* = 11.6 Hz, 2H), 4.47 (d, *J* = 12.2 Hz, 1H), 4.38 (d, *J* = 11.7 Hz, 1H), 4.33 (d, *J* = 7.7 Hz, 1H, **H_1(β)**), 4.29 (d, *J* = 11.5 Hz, 1H), 4.02 – 3.94 (m, 2H), 3.93 (s, 1H), 3.88 (t, *J* = 6.6 Hz, 1H), 3.85 – 3.73 (m, 4H), 3.70 (dd, *J* = 10.8, 5.2 Hz, 1H), 3.63 – 3.58 (m, 1H), 3.57 (s, 3H), 3.47 (m, 3H) ppm.

Data of the anomeric mixture:

^{13}C NMR (151 MHz, $CDCl_3$) δ 138.7, 138.6, 138.4, 138.3, 138.2, 138.0, 128.5, 128.4, 128.4, 128.3, 128.3, 128.3, 128.3, 128.2, 128.2, 128.1, 128.1, 128.0, 128.0, 127.9, 127.9, 127.8, 127.8, 127.7, 127.6, 127.5, 127.5, 127.4, 127.4, 127.4, 127.3, 127.0, 126.6, 104.6 **C_1'(β)**, 104.4 **C_1(β)**, 102.8 **C_1(β)**, 97.4 **C_1'(α)**, 84.8, 83.9, 82.9, 82.5, 82.4, 81.8, 81.8, 79.9, 79.1, 77.2, 77.0, 76.8, 75.4, 75.4, 75.3, 75.2, 75.1, 74.9, 74.7, 74.7, 74.7, 74.6, 74.5, 74.3, 74.0, 74.0, 73.8, 73.7, 73.5, 73.5, 73.4, 73.2, 73.1, 72.9, 72.7, 72.6, 72.5, 71.6, 70.3, 69.8, 69.6, 68.7, 68.3, 68.0, 57.1, 57.0 ppm.

HRMS (ESI): Calcd for $C_{62}H_{66}O_{11}Na$ $[M + Na]^+$ 1009.4503; found 1009.4631.

NP-HPLC of **4Bn-A2** (ELSD trace, t*R* (α) = 11.9 min, t*R* (β) = 12.2 min):

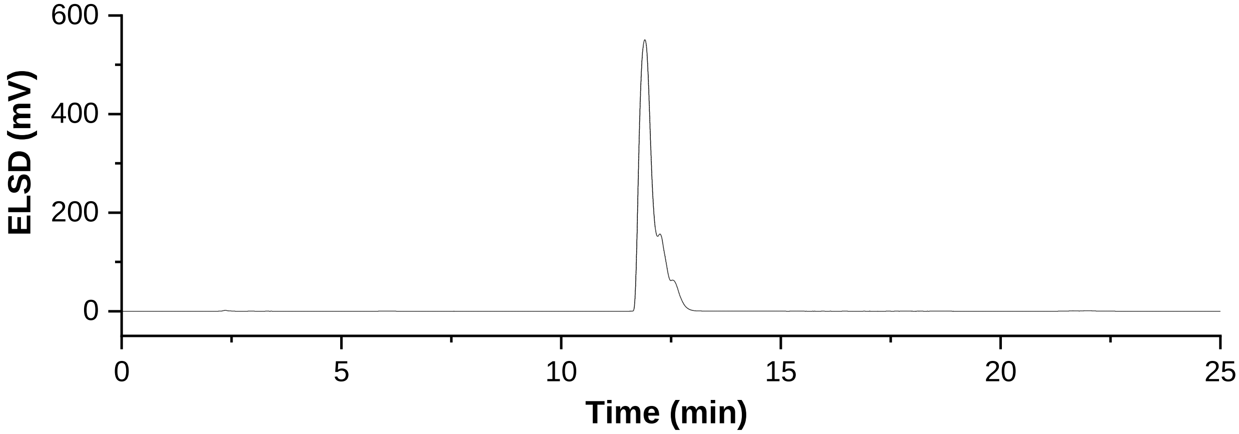

^{1}H NMR (600 MHz, $CDCl_3$) of **4Bn-A2**:

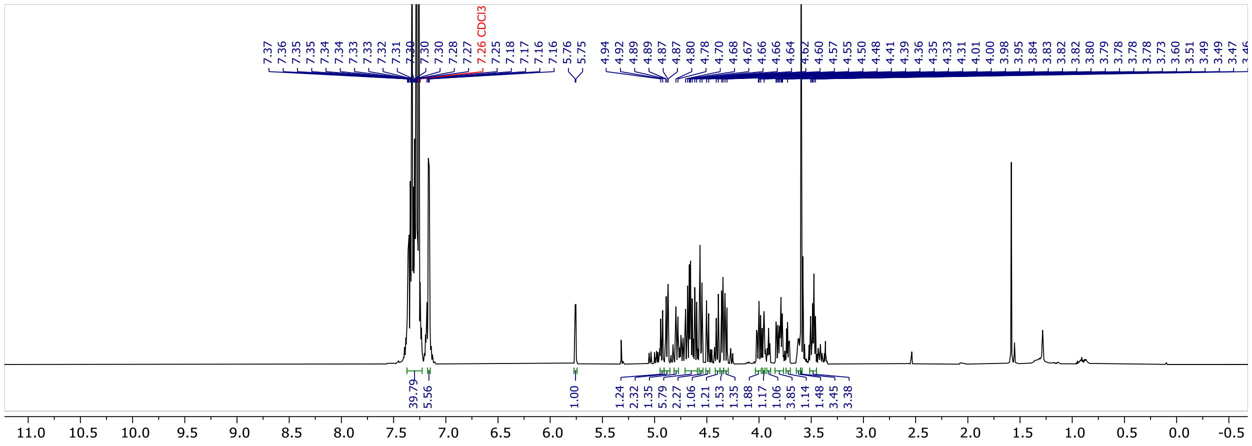

^{13}C NMR (151 MHz, $CDCl_3$) of **4Bn-A2**:

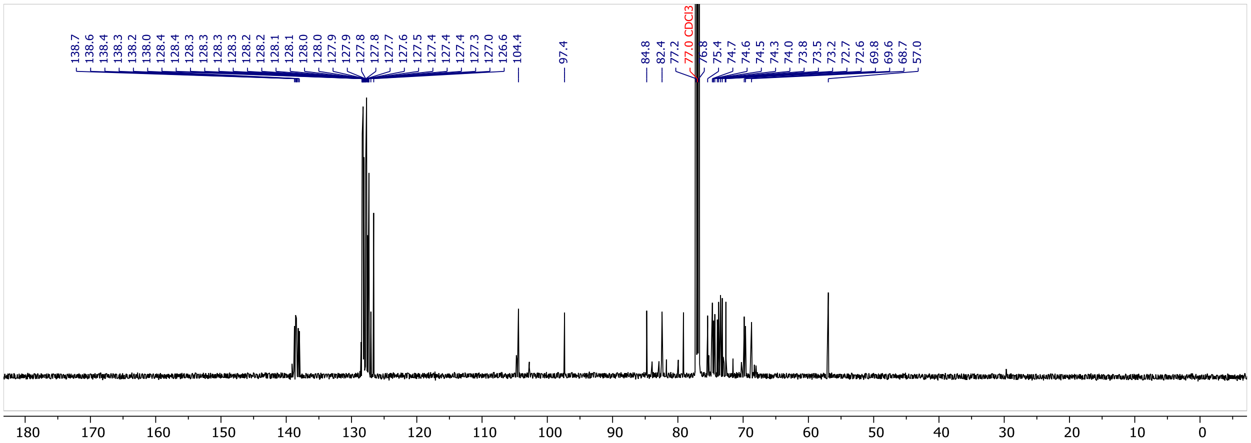

$^{13}C,^{1}H$ HSQC of **4Bn-A2**:

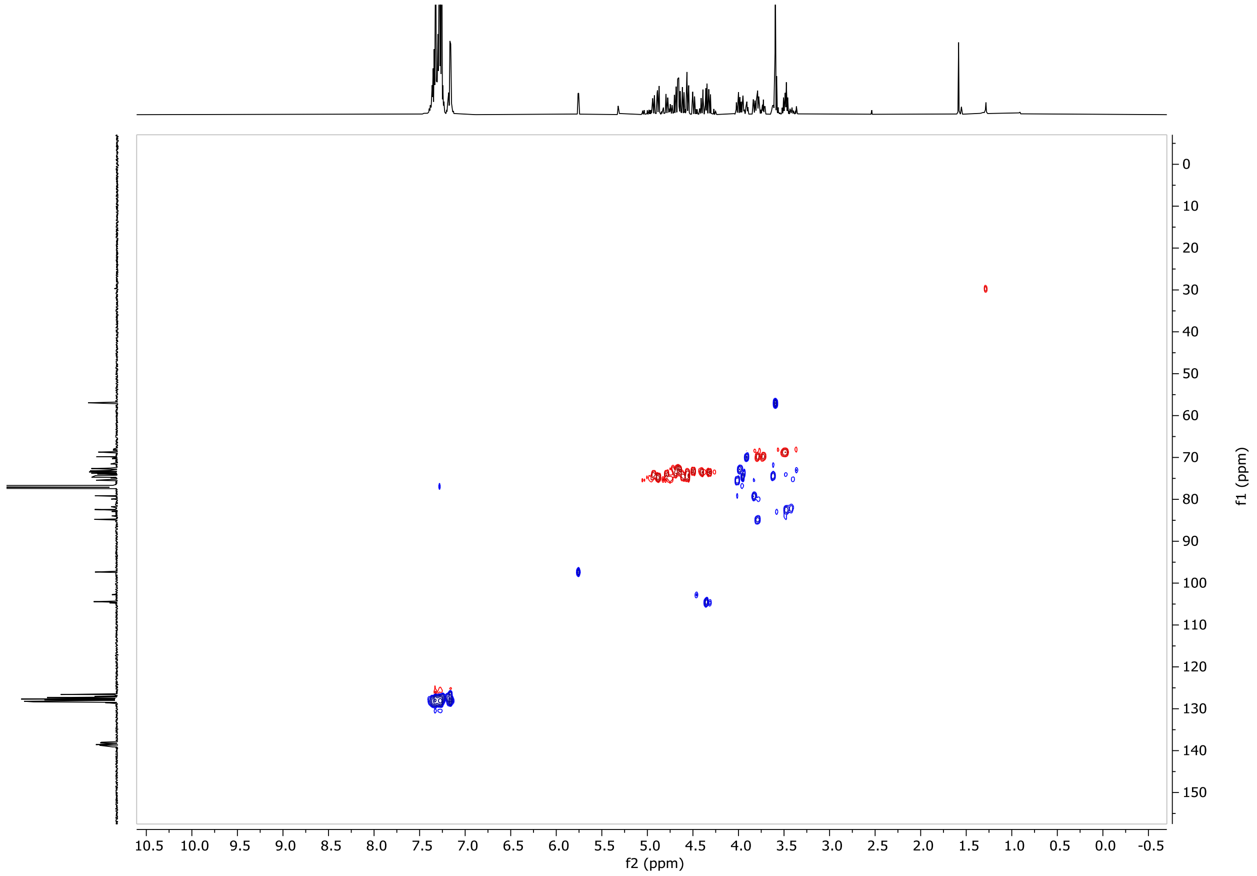

2,2,2-Trifluoroethyl 2,3,4,6-tetra-*O*-benzyl-α/β-D-galactopyranoside (4Bn-A3)

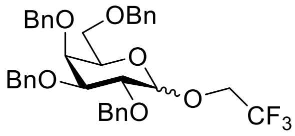

The title compound was prepared according to general procedure for glycosylations. Product **4Bn-A3** (17 mg, 28 μmol, 80%, **α/β 68:32**) was obtained as a colorless oil after purification using **Method-2b** (t*R* (α) = 6.1 min, tR (β) = 7.3 min).

Data of the anomeric mixture:

^{1}H NMR (600 MHz, $CDCl_3$) δ 7.40 – 7.26 (m, 38H), 7.25 (s, 2H), 4.94 (d, *J* = 11.5 Hz, 2H), 4.90 (d, *J* = 10.5 Hz, 1H), 4.88 (d, *J* = 3.7 Hz, 1H, **H_1(α)**), 4.85 (d, *J* = 11.6 Hz, 1H), 4.82 (d, *J* = 11.9 Hz, 1H), 4.77 (d, *J* = 11.8

Hz, 1H), 4.75 – 4.69 (m, 3H), 4.66 (d, *J* = 11.9 Hz, 2H), 4.62 (d, *J* = 11.6 Hz, 1H), 4.56 (d, *J* = 11.4 Hz, 1H), 4.47 (d, *J* = 7.0 Hz, 1H, **H_1(β)**), 4.45 (d, *J* = 7.5 Hz, 1H), 4.43 – 4.38 (m, 3H), 4.17 (dq, *J* = 12.4, 8.8 Hz, 1H), 4.08 (d, *J* = 3.7 Hz, 1H), 4.06 (d, *J* = 3.7 Hz, 1H), 3.97 (d, *J* = 2.0 Hz, 2H), 3.96 – 3.83 (m, 8H), 3.60 – 3.52 (m, 3H), 3.51 (s, 2H), 3.50 (s, 1H) ppm.

^{13}C NMR (151 MHz, $CDCl_3$) δ 138.7, 138.4, 138.4, 137.8, 137.7, 128.5, 128.5, 128.4, 128.4, 128.4, 128.4, 128.3, 128.2, 128.2, 127.9, 127.9, 127.8, 127.7, 127.7, 127.6, 127.5, 127.5, 127.5, 103.9 (**C_1(β)**), 98.2 (**C_1(α)**), 81.8, 79.0, 78.6, 77.2, 77.0, 76.8, 76.1, 75.3, 74.8, 74.8, 74.6, 73.7, 73.6, 73.5, 73.4, 73.3, 73.2, 70.0, 68.7, 64.6 ppm.

HRMS (ESI): Calcd for $C_{36}H_{37}F_3O_6Na$ $[M + Na]^+$ 645.2440; found 645.2419.

NP-HPLC of **4Bn-A3** (ELSD trace, t*R* (α) = 6.1 min, t*R* (β) = 7.3 min):

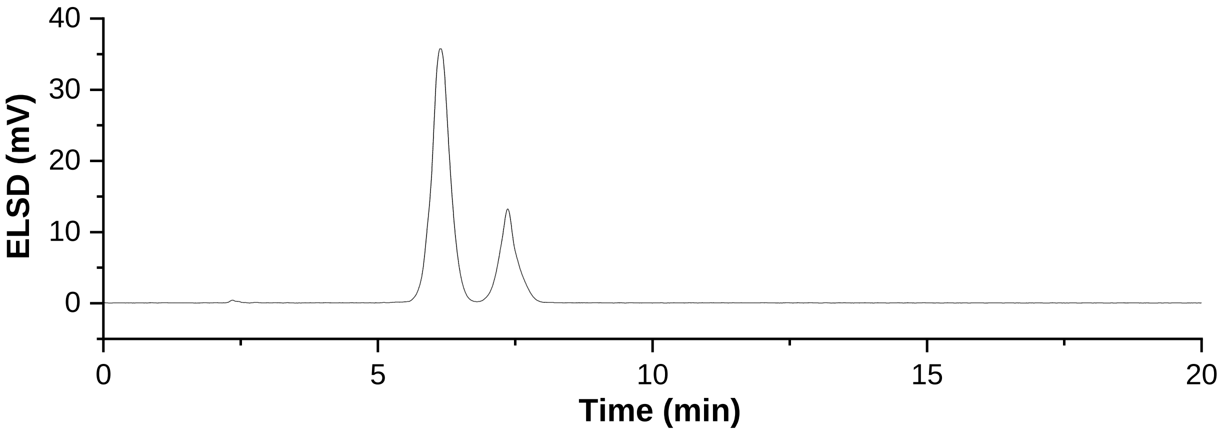

^{1}H NMR (600 MHz, $CDCl_3$) of **4Bn-A3**:

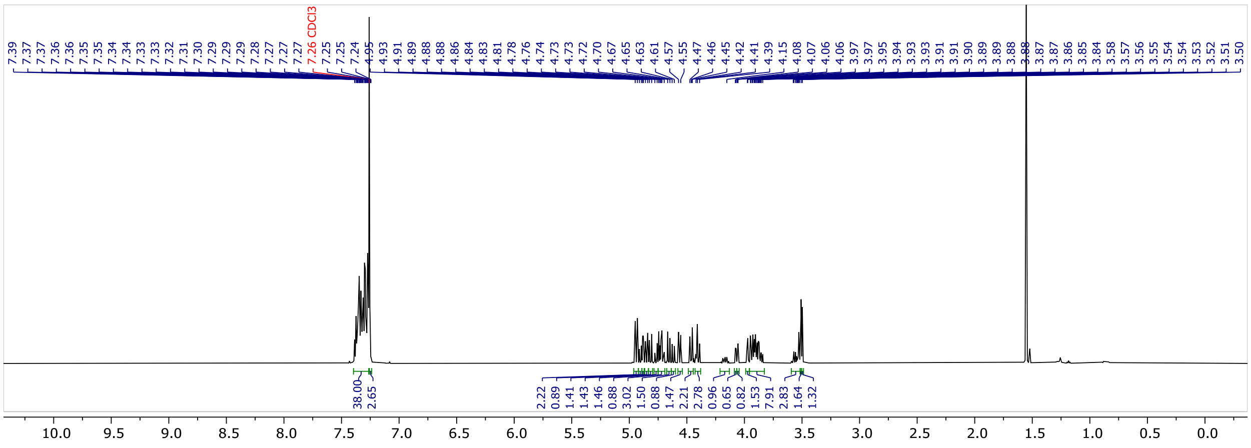

^{13}C NMR (151 MHz, $CDCl_3$) of **4Bn-A3**:

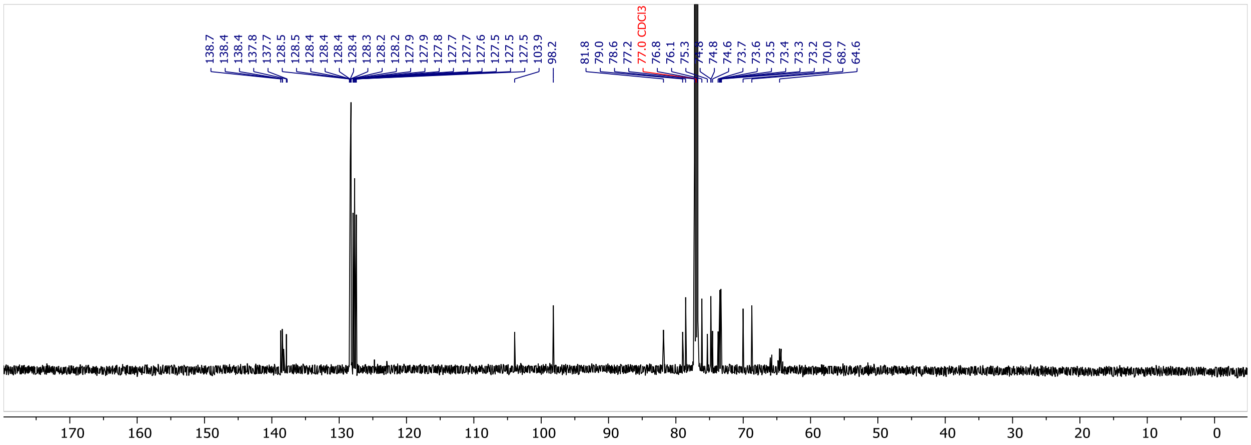

^{13}C,^{1}H HSQC of **4Bn-A3**:

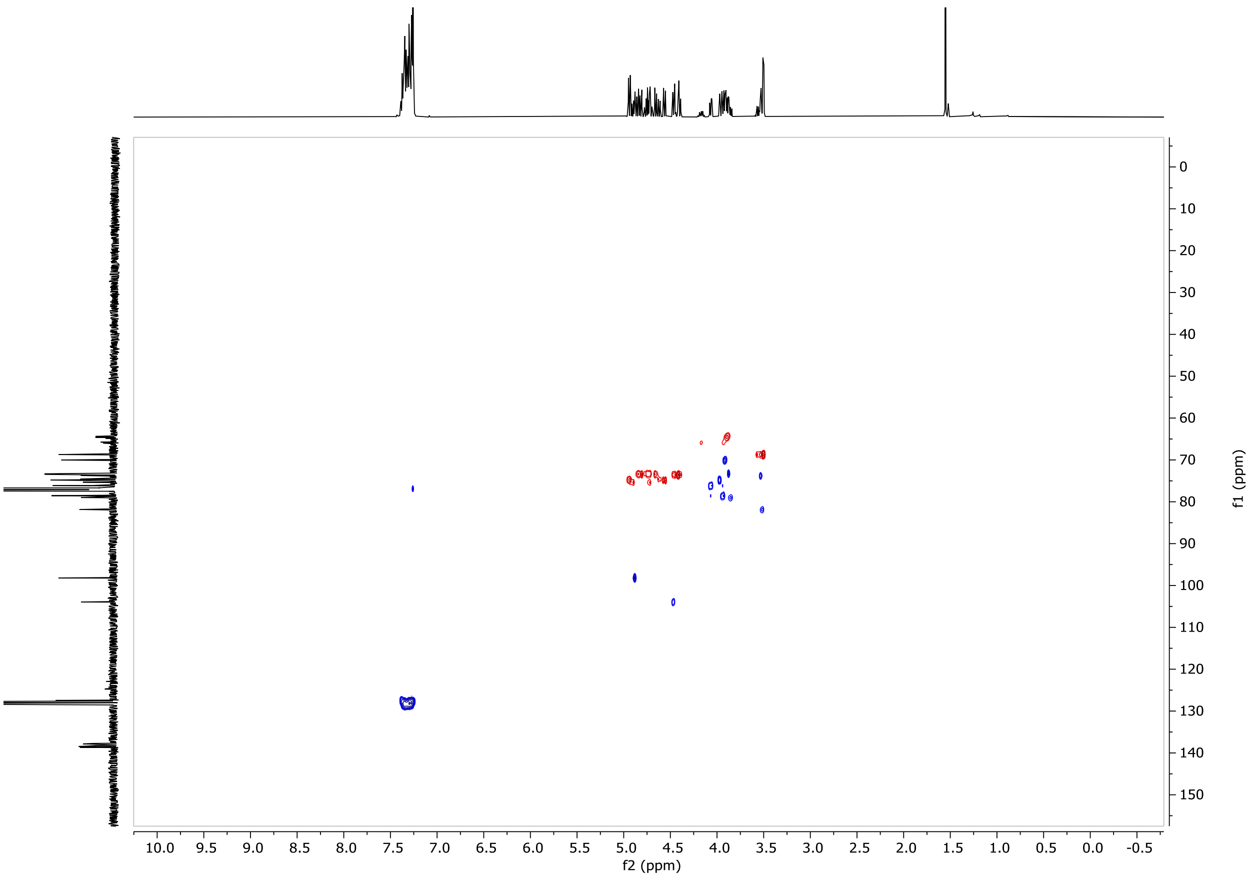

2,3,4,6-tetra-*O*-benzyl-α/β-D-galactopyranosyl (4Bn-A4)

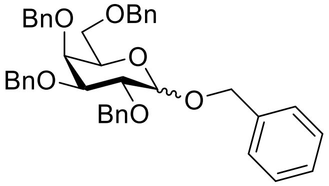

The title compound was prepared according to general procedure for glycosylations. Product **4Bn-A4** (17 mg, 27 μmol, 77%, **α/β 18:82**) was obtained as a colorless oil after purification using **Method-2b** (t*R* (α) = 8.1 min, t*R* (β) = 8.6 min).

Data of the anomeric mixture:

^{1}H NMR (600 MHz, $CDCl_3$) δ 7.41 – 7.23 (m, 50H), 4.97 – 4.91 (m, 6H), 4.90 (d, *J* = 3.5 Hz, 1H, **H1(α)**), 4.85 (d, *J* = 11.7 Hz, 1H), 4.79 – 4.67 (m, 8H), 4.63 (dd, *J* = 11.9, 2.7 Hz, 4H), 4.60 – 4.54 (m, 3H), 4.49 – 4.42 (m, 6H, **H1(β)**, CH_2Ph), 4.42 – 4.38 (m, 2H), 4.06 – 3.94 (m, 4H), 3.92 – 3.86 (m, 4H), 3.66 – 3.57 (m, 4H), 3.56 – 3.49 (m, 5H), 3.46 (dd, *J* = 9.3, 6.0 Hz, 1H) ppm.

^{13}C NMR (151 MHz, $CDCl_3$) δ 138.5, 137.6, 128.4, 128.4, 128.4, 128.3, 128.3, 128.2, 128.2, 128.2, 127.9, 127.8, 127.8, 127.8, 127.7, 127.7, 127.6, 127.6, 127.5, 127.4, 102.8 (**C1(β)**), 96.1 (**C1(α)**), 82.3, 79.6, 79.2, 77.2, 77.0, 76.8, 76.5, 75.2, 75.0, 74.7, 74.5, 73.5, 73.5, 73.4, 73.1, 70.9, 69.5, 69.0, 68.9 ppm.

HRMS (ESI): Calcd for $C_{41}H_{42}O_6Na$ $[M + Na]^+$ 653.2879; found 653.2873.

NP-HPLC of **4Bn-A4** (ELSD trace, t*R* (α) = 8.1 min, t*R* (β) = 8.6 min):

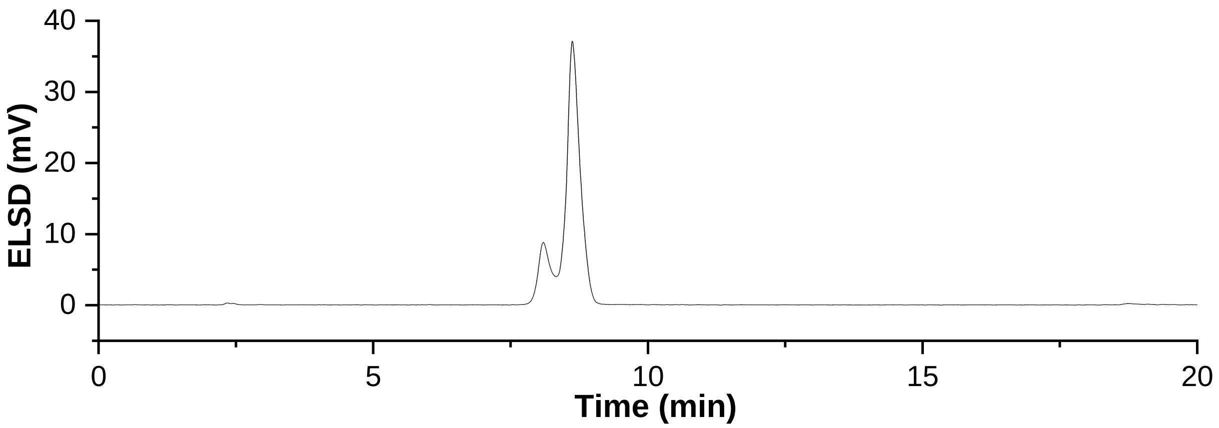

^{1}H NMR (600 MHz, $CDCl_3$) of **4Bn-A4**:

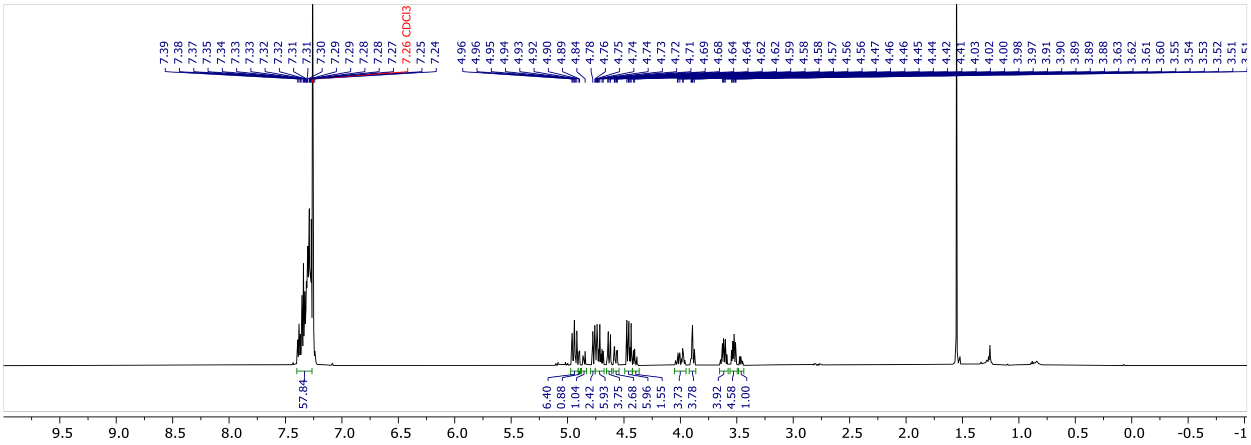

^{13}C NMR (151 MHz, $CDCl_3$) of **4Bn-A4**:

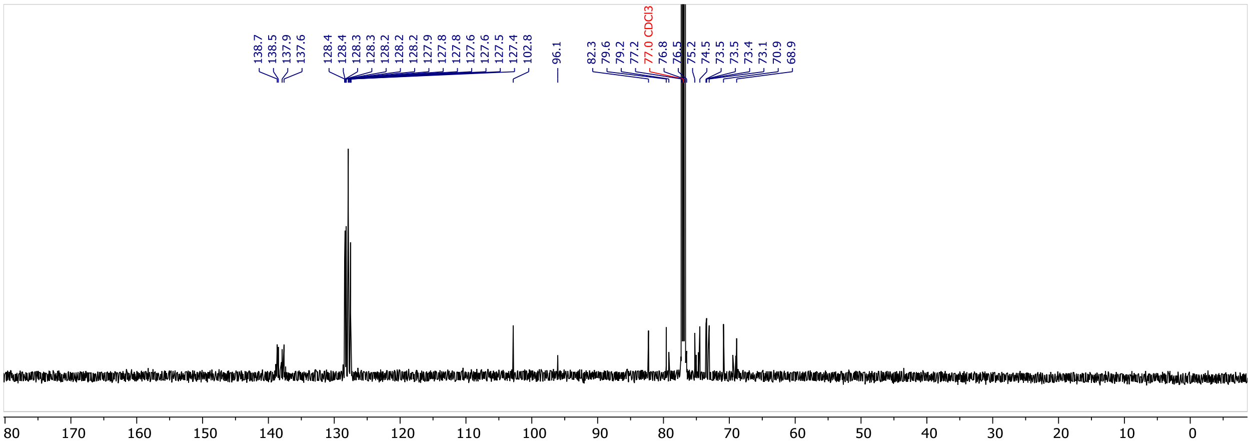

$^{13}C,^{1}H$ HSQC of **4Bn-A4**:

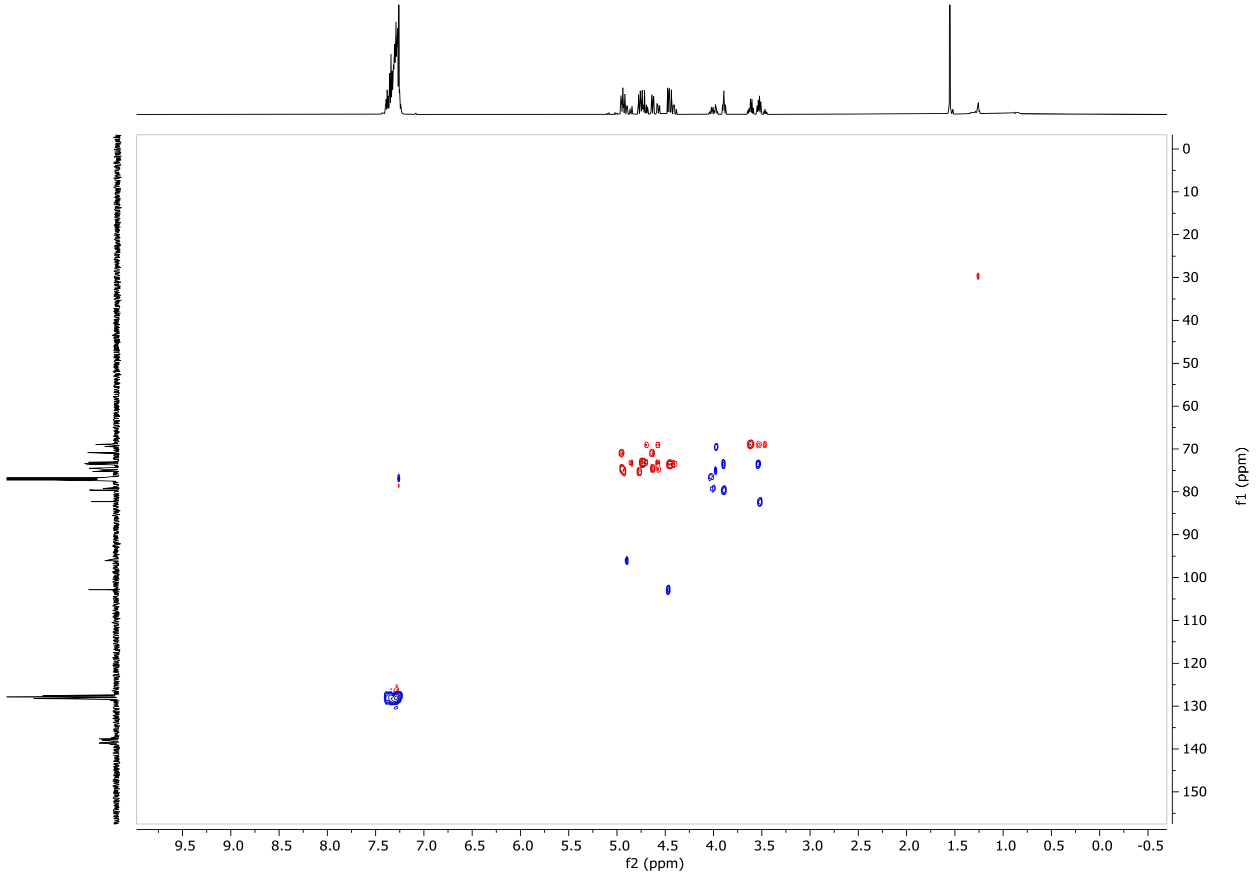

1,2:3,4-Bis-*O*-(1-methylethylidene)-6-*O*-[4-*O*-acetyl-2,3,6-tris-*O*-benzyl-α/β-D-galactopyranosyl]-α-D-galactopyranoside (4Ac-A1)

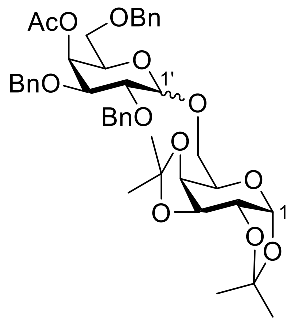

The title compound was prepared according to general procedure for glycosylations. Product **4Ac-A1** (21 mg, 28 μmol, 80%, **α/β 63:37**) was obtained as a colorless oil after purification using **Method-2b** (*tR* (α) = 11.5 min, *tR* (β) = 12.8 min).

Data of the anomeric mixture:

^{1}H NMR (600 MHz, CDCl$_3$) δ 7.43 (d, *J* = 6.9 Hz, 2H), 7.40 – 7.23 (m, 28H), 5.65 (d, *J* = 2.8 Hz, 1H), 5.59 – 5.54 (m, 2H, **H1(α)**), 5.51 (d, *J* = 5.0 Hz, 1H, **H1(α)**), 5.05 – 4.99 (m, 2H, **H1'(α)**), 4.82 – 4.73 (m, 3H), 4.72 – 4.65 (m, 2H), 4.62 (dd, *J* = 7.9, 2.4 Hz, 1H), 4.60 – 4.51 (m, 5H), 4.50 – 4.43 (m, 2H, **H1'(β)**), 4.34 (dd, *J* = 5.0, 2.4 Hz, 1H), 4.33 – 4.29 (m, 2H), 4.28 (dd, *J* = 7.9, 1.4 Hz, 1H), 4.22 (dd, *J* = 7.9, 1.9 Hz, 1H), 4.20 (t, *J* = 6.8 Hz, 1H), 4.14 (dd, *J* = 10.8, 3.6 Hz, 1H), 4.11 – 4.07 (m, 1H), 4.05 – 4.01 (m, 1H), 3.97 (dd, *J* = 10.0, 3.4 Hz, 1H), 3.92 – 3.84 (m, 2H), 3.84 – 3.77 (m, 2H), 3.77 – 3.69 (m, 3H), 3.63 – 3.53 (m, 2H), 3.53 – 3.43 (m, 2H), 2.06 (s, 3H), 2.04 (s, 3H), 1.54 (s, 2H), 1.52 (s, 3H), 1.49 (s, 2H), 1.47 (s, 2H), 1.44 (s, 2H), 1.43 (s, 2H), 1.34 (s, 3H), 1.33 (s, 2H), 1.31 (s, 3H), 1.31 (s, 3H).

^{13}C NMR (151 MHz, CDCl$_3$) δ 170.4, 170.3, 138.9, 138.6, 138.2, 137.9, 137.8, 137.6, 128.4, 128.4, 128.4, 128.3, 128.3, 128.3, 128.2, 128.2, 128.1, 128.0, 128.0, 128.0, 127.9, 127.9, 127.8, 127.7, 127.6, 127.5, 127.5, 127.5, 127.3, 109.4, 109.4, 109.2, 108.7, 108.6, 108.5, 104.6 (**C1'(β)**), 97.8 (**C1'(α)**), 96.3 (**C1(α)**), 96.3 (**C1(α)**), 78.9, 78.5, 77.2, 77.0, 76.8, 76.2, 75.5, 74.9, 73.7, 73.5, 72.9, 72.1, 72.0, 71.6, 71.4, 70.8, 70.7, 70.6, 70.6, 70.5, 70.4, 70.0, 68.2, 68.1, 68.0, 68.0, 67.5, 67.3, 66.9, 66.6, 66.0, 62.4, 26.1, 26.0, 26.0, 26.0, 25.9, 25.0, 24.9, 24.9, 24.6, 24.4, 24.3, 20.9, 20.9.

HRMS (ESI): Calcd for $C_{41}H_{50}O_{12}Na$ $[M + Na]^+$ 757.3200; found 757.3296.

NP-HPLC of **4Ac-A1** (ELSD trace, *tR* (α) = 11.5 min, *tR* (β) = 12.8 min):

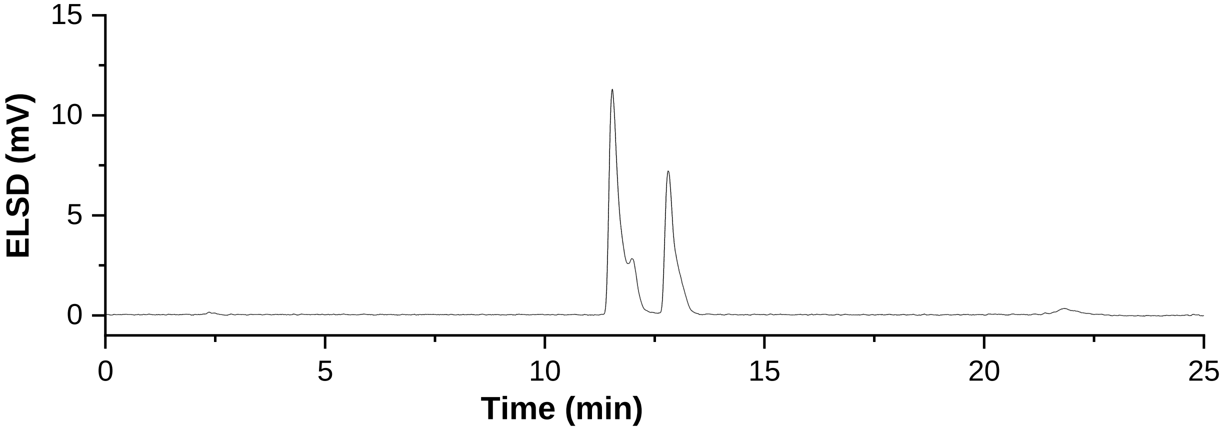

^{1}H NMR (600 MHz, $CDCl_3$) of **4Ac-A1**:

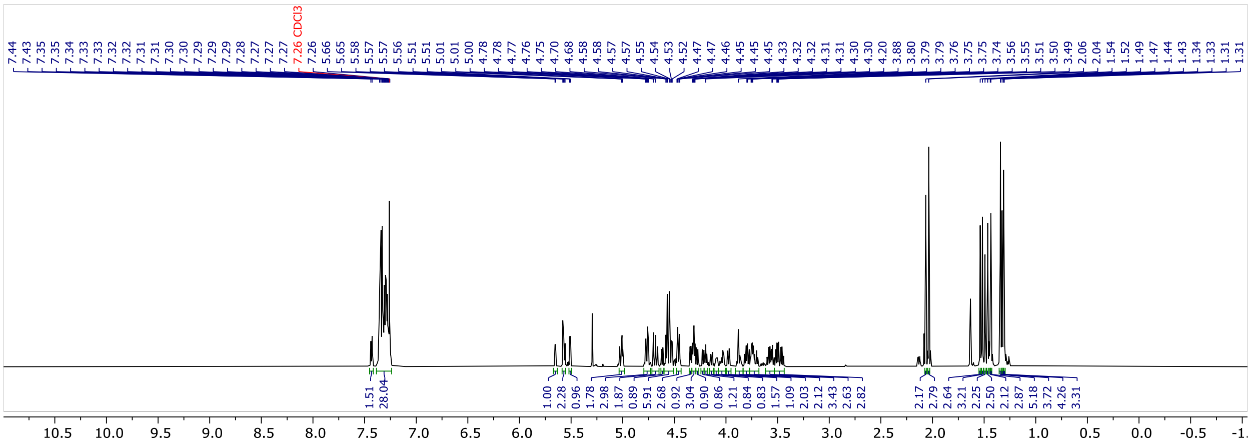

^{13}C NMR (151 MHz, $CDCl_3$) of **4Ac-A1**:

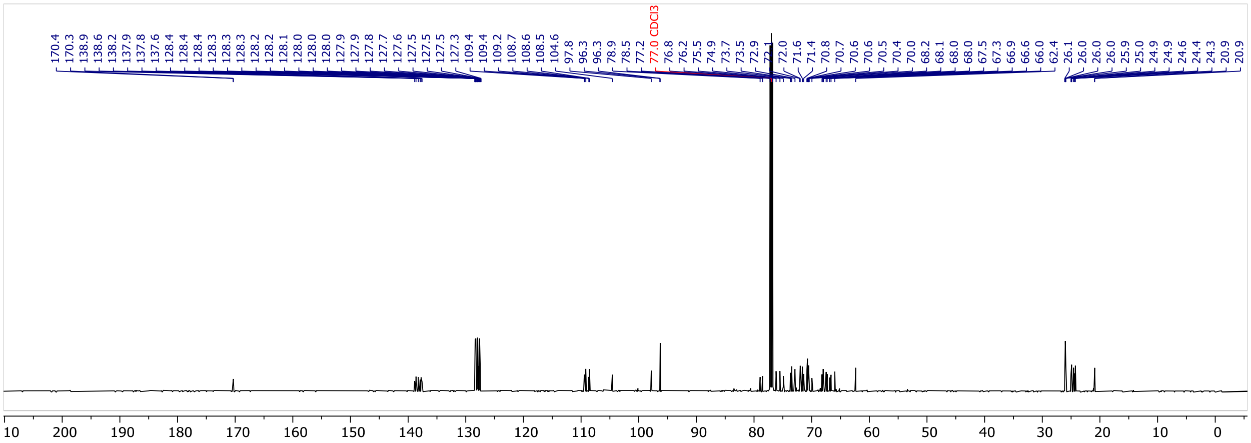

$^{13}C,^{1}H$ HSQC of **4Ac-A1**:

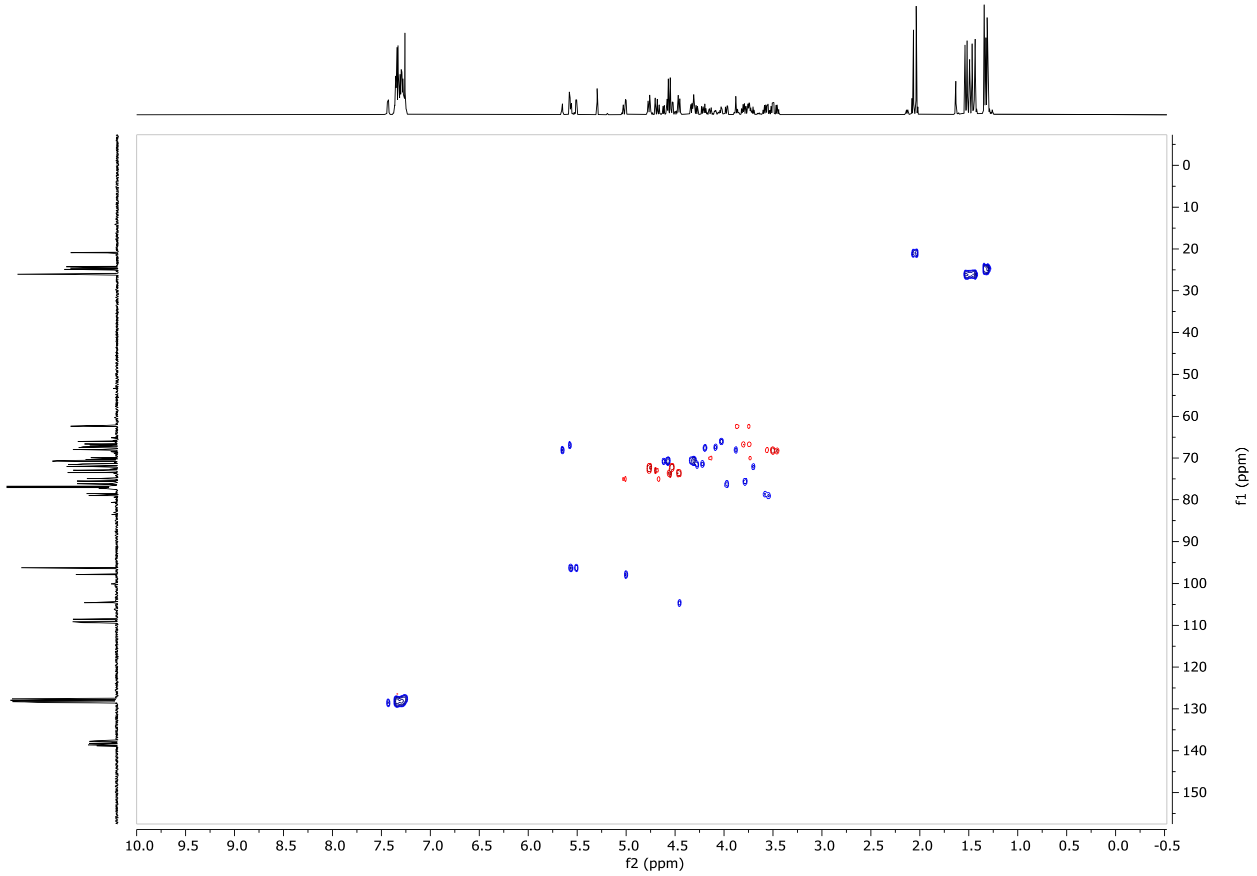

Methyl *O*-[4-*O*-acetyl-2,3,6-tris-*O*-benzyl-α/β-D-galactopyranosyl]-(1→4)-2,3,6-tri-*O*-benzyl-β-D-glucopyranoside (4Ac-A2)

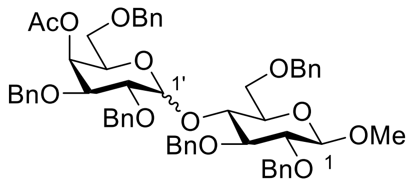

The title compound was prepared according to general procedure for glycosylations. Product **4Ac-A2** (24 mg, 25 µmol, 72%, **α/β 60:40**) was obtained as a colorless oil after purification using **Method-2b** (t*R* (α) = 12.5 min, t*R* (β) = 13.0 min).

Data of the anomeric mixture (**α**-major product):

^{1}H NMR (600 MHz, $CDCl_3$) δ 7.50 – 7.24 (m, 53H), 7.22 – 7.10 (m, 10H), 5.77 (d, *J* = 3.9 Hz, 1H, **H1'(α)**), 5.58 (s, 2H), 4.99 (d, *J* = 10.5 Hz, 1H), 4.93 (d, *J* = 11.8 Hz, 1H), 4.89 (d, *J* = 10.9 Hz, 2H), 4.84 – 4.75 (m,

3H), 4.75 – 4.67 (m, 4H), 4.66 (d, J = 12.2 Hz, 1H), 4.64 – 4.56 (m, 3H), 4.56 – 4.44 (m, 6H, **H1'(β)**), 4.44 – 4.34 (m, 3H, **H1(β)**), 4.33 – 4.25 (m, 3H, **H1(β)**), 4.06 (t, J = 6.4 Hz, 1H), 4.04 – 3.96 (m, 2H), 3.85 (dd, J = 10.3, 3.2 Hz, 1H), 3.83 – 3.70 (m, 6H), 3.64 – 3.55 (m, 9H), 3.53 – 3.44 (m, 4H), 3.44 – 3.37 (m, 3H), 3.38 – 3.30 (m, 2H), 2.06 (s, 3H), 2.01 (s, 3H) ppm.

^{13}C NMR (151 MHz, $CDCl_3$) δ 170.2, 170.2, 139.1, 138.8, 138.7, 138.6, 138.6, 138.4, 138.3, 138.3, 138.2, 138.1, 138.0, 137.9, 137.8, 137.7, 128.4, 128.4, 128.3, 128.3, 128.3, 128.3, 128.2, 128.2, 128.1, 128.1, 128.0, 128.0, 127.8, 127.7, 127.7, 127.7, 127.6, 127.6, 127.6, 127.5, 127.4, 127.0, 126.6, 104.7 (**C1(β)**), 104.5 (**C1(β)**), 102.3 (**C1'(β)**) , 97.4 (**C1'(α)**) , 84.8, 82.7, 82.4, 81.8, 79.8, 79.4, 77.2, 77.0, 76.8, 76.5, 76.4, 75.3, 75.2, 75.0, 74.9, 74.6, 74.6, 74.2, 73.9, 73.9, 73.6, 73.5, 73.3, 73.1, 72.8, 71.8, 71.8, 71.6, 71.6, 69.5, 68.1, 68.0, 67.7, 57.1, 57.0, 20.9 ppm.

HRMS (ESI): Calcd for $C_{57}H_{62}O_{12}Na$ $[M + Na]^+$ 961.4139; found 961.4299.

NP-HPLC of **4Ac-A2** (ELSD trace, *tR* (α) = 12.5 min, *tR* (β) = 13.0 min):

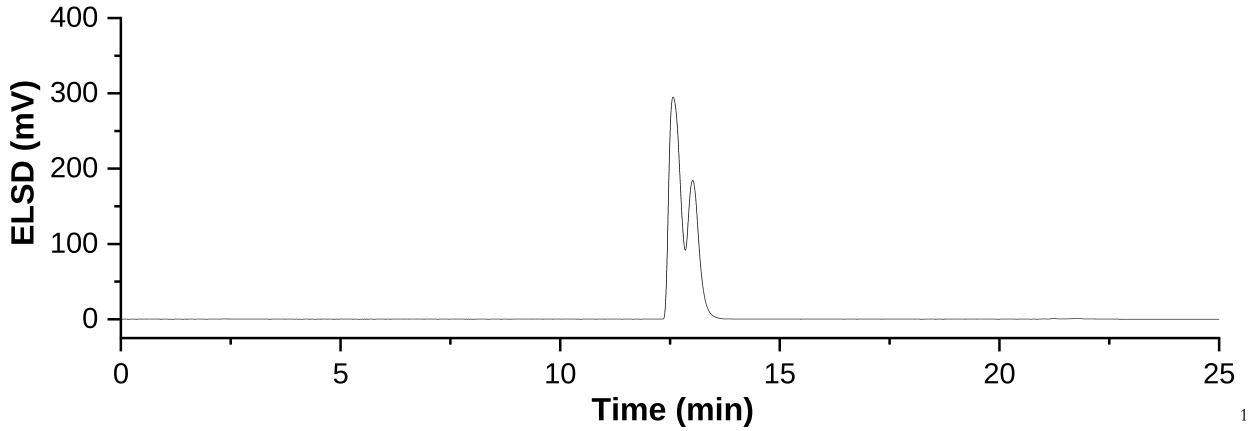

H NMR (600 MHz, $CDCl_3$) of **4Ac-A2**:

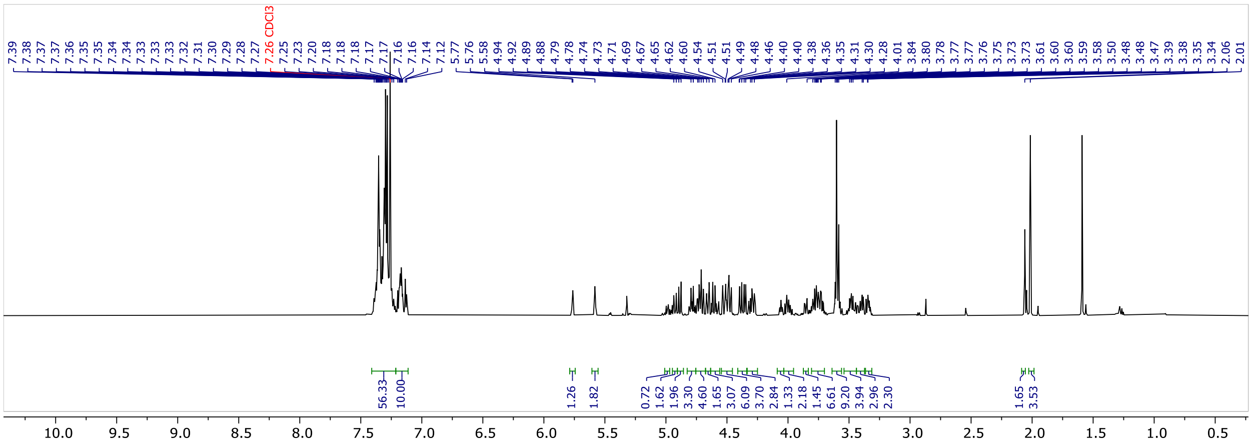

^{13}C NMR (151 MHz, $CDCl_3$) of **4Ac-A2**:

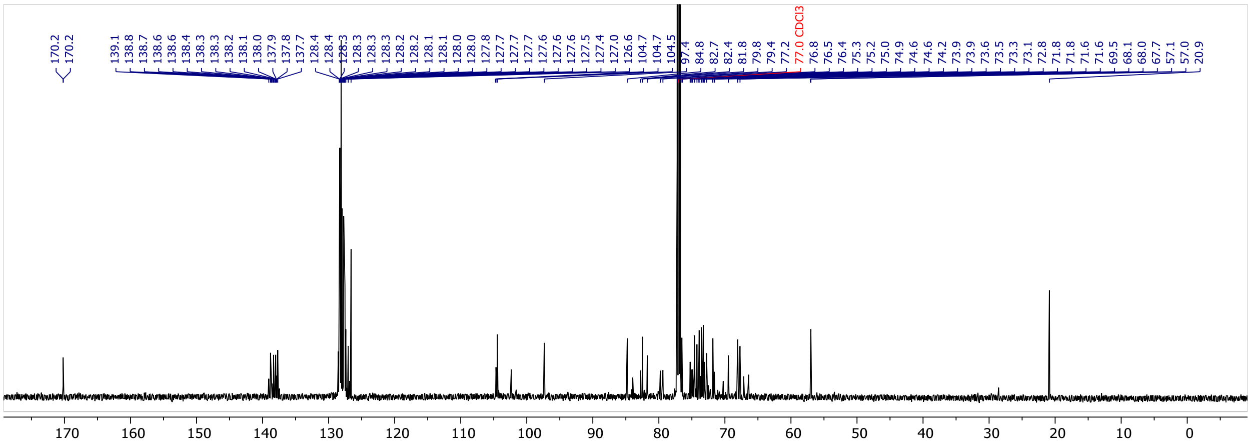

^{13}C,^{1}H HSQC of **4Ac-A2**:

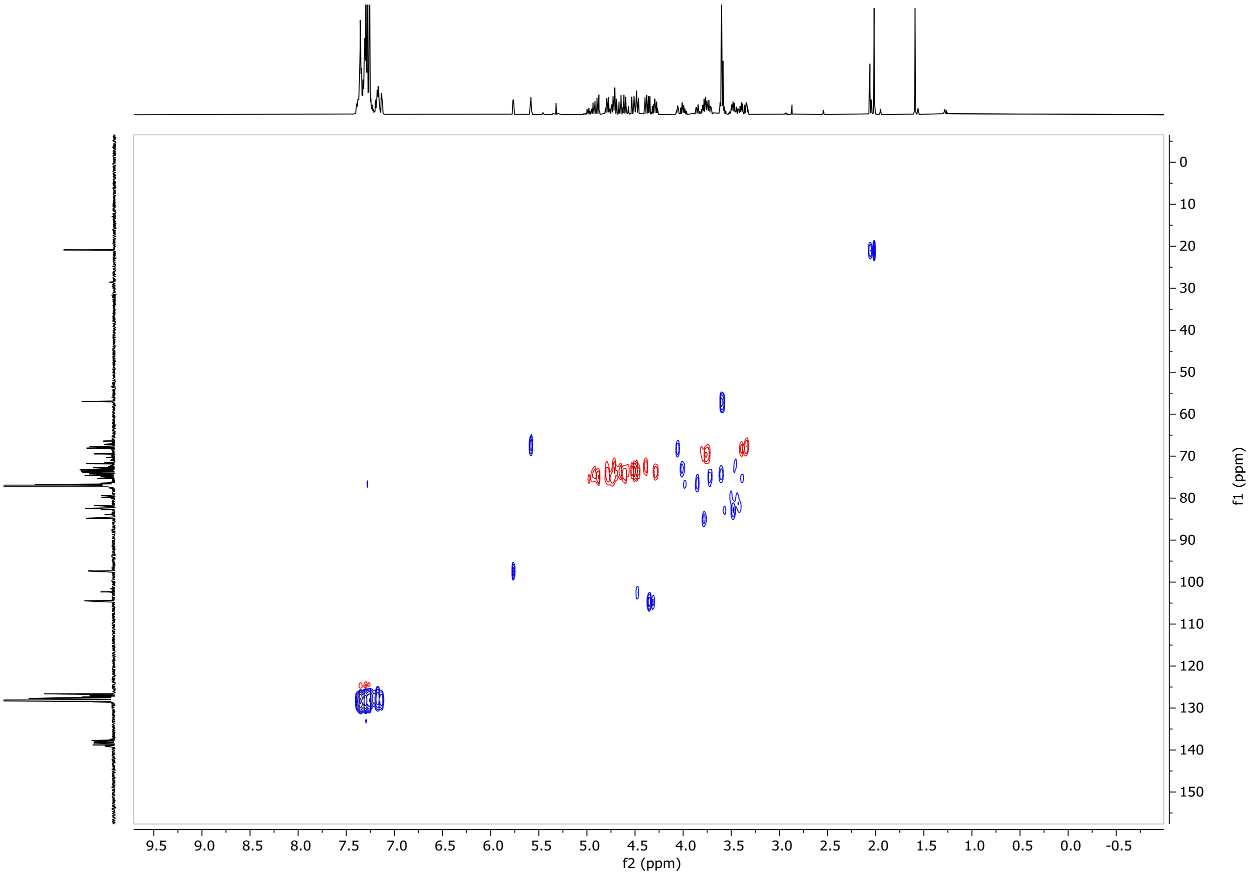

2,2,2-Trifluoroethyl-4-*O*-Acetyl-2,3,6-tri-*O*-benzyl-α-D-galactopyranoside (4Ac-A3)

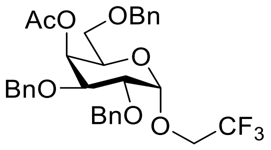

The title compound was prepared according to general procedure for glycosylations. Product **4Ac-A3** (13 mg, 23 µmol, 66%, **α/β 100:0**) was obtained as a colorless oil after purification using **Method-2b** (t*R* (α) = 8.2 min).

Data of the α-anomer:

^{1}H NMR (600 MHz, $CDCl_3$) δ 7.38 – 7.26 (m, 15H), 5.63 (d, *J* = 2.7 Hz, 1H), 4.90 (d, *J* = 3.7 Hz, 1H, **H1**), 4.83 (d, *J* = 11.9 Hz, 1H), 4.75 (d, *J* = 10.9 Hz, 1H), 4.62 (d, *J* = 11.9 Hz, 1H), 4.55 (dd, *J* = 11.4, 3.7 Hz, 2H),

4.45 (d, J = 11.9 Hz, 1H), 4.08 (t, J = 6.3 Hz, 1H), 3.96 (dd, J = 10.1, 3.4 Hz, 1H), 3.92 (m , 3H), 3.80 (dd, J = 10.1, 3.7 Hz, 1H), 3.47 (m, 2H), 2.05 (s, 3H).

^{13}C NMR (151 MHz, $CDCl_3$) δ 170.2, 138.4, 138.0, 137.6, 128.4, 128.4, 128.3, 128.0, 127.8, 127.8, 127.6, 98.4 (**C1**), 77.2, 77.0, 76.8, 75.8, 75.1, 73.6, 73.5, 72.2, 68.5, 68.3, 67.8, 64.8, 64.6, 20.9.

HRMS (ESI): Calcd for $C_{31}H_{33}F_3O_7Na$ $[M + Na]^+$ 597.2076; found 597.2153.

NP-HPLC of **4Ac-A3** (ELSD trace, tR (α) = 8.2 min):

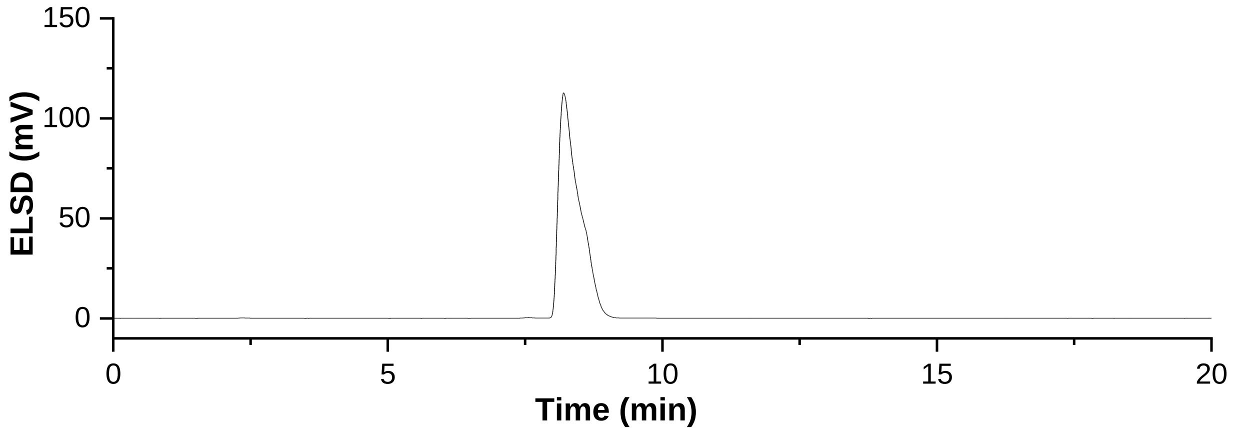

^{1}H NMR (600 MHz, $CDCl_3$) of **4Ac-A3**:

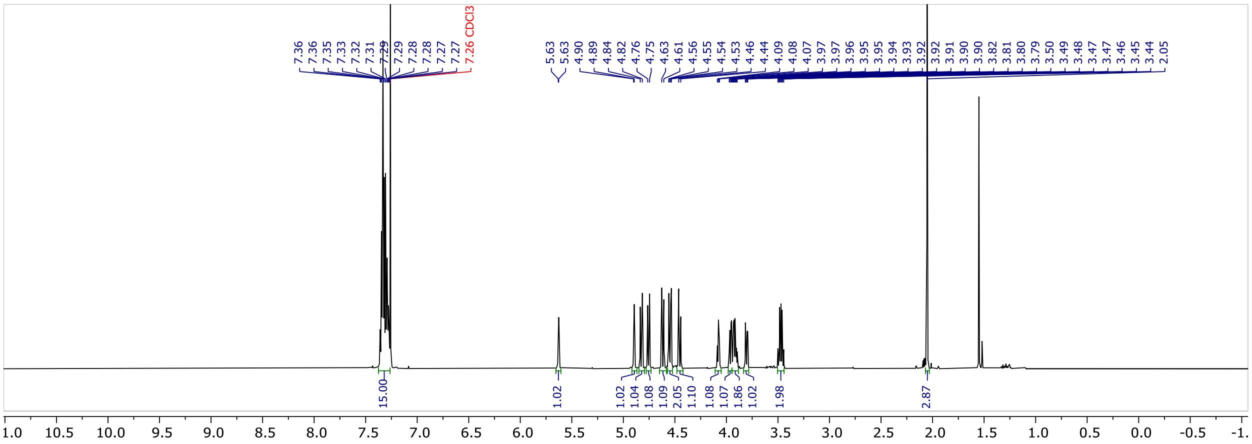

^{13}C NMR (151 MHz, $CDCl_3$) of **4Ac-A3**:

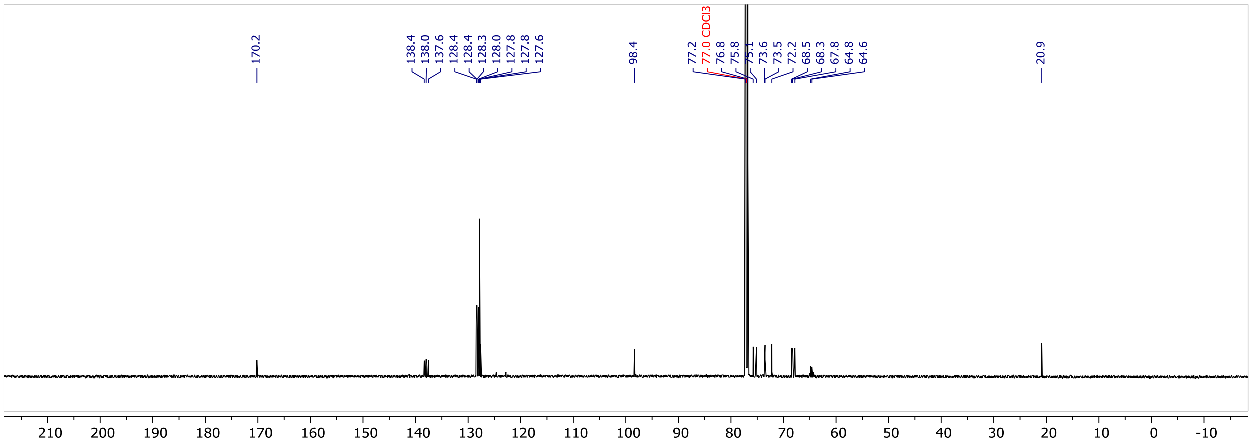

^{13}C,^{1}H HSQC of **4Ac-A3**:

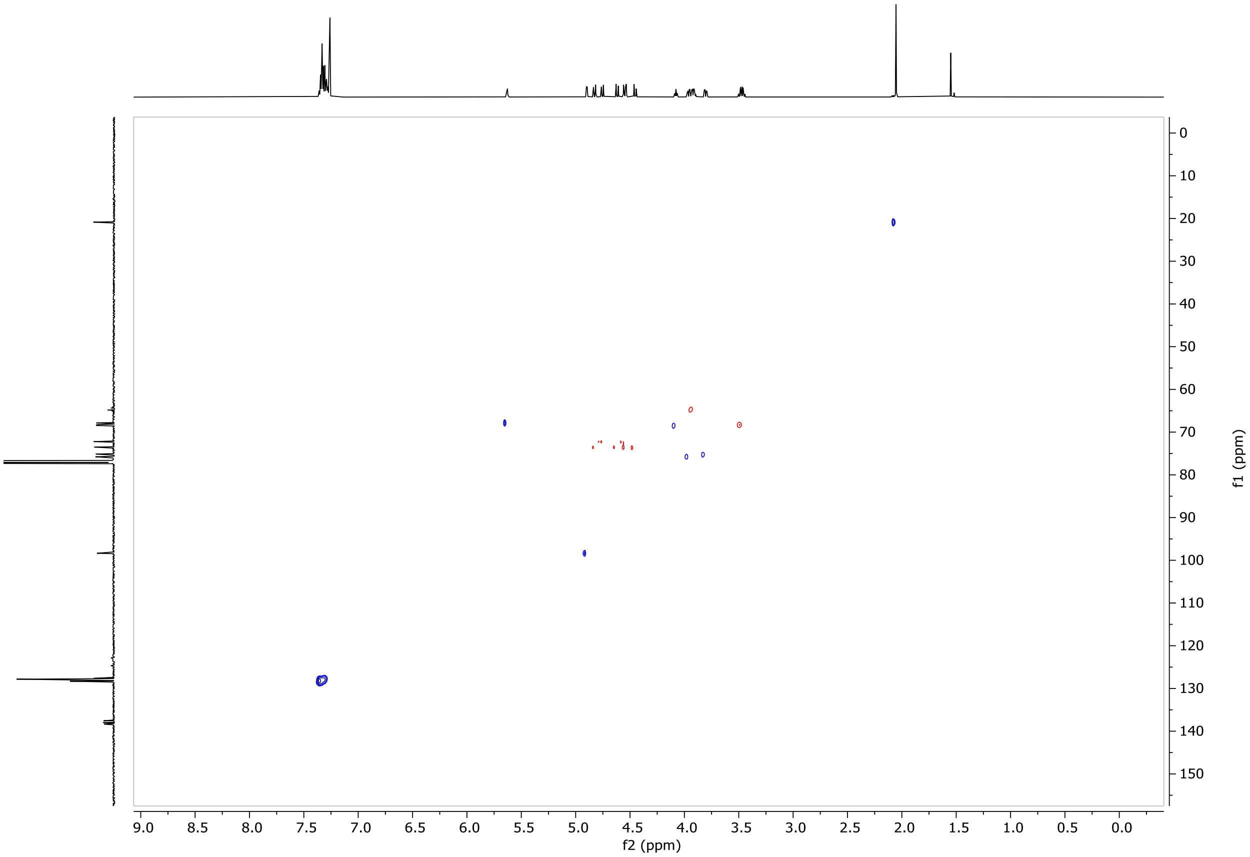

4-*O*-Acetyl-2,3,6-tris-*O*-benzyl-α/β-D-galactopyranosyl (4Ac-A4)

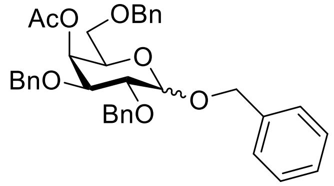

The title compound was prepared according to general procedure for glycosylations. Product **4Ac-A4** (14 mg, 24 μmol, 68%, **α/β 50:50**) was obtained as a colorless oil after purification using **Method-2b** (t*R* (α) = 7.9 min, t*R* (β) = 8.8 min).

Data of the anomeric mixture:

^{1}H NMR (600 MHz, $CDCl_3$) δ 7.45 – 7.21 (m, 46H), 5.63 (d, *J* = 2.9 Hz, 1H), 5.59 (d, *J* = 3.1 Hz, 1H), 4.97 (d, *J* = 12.0 Hz, 1H), 4.90 (d, *J* = 3.8 Hz, 1H, **H1(α)**), 4.88 (d, *J* = 10.7 Hz, 1H), 4.82 – 4.65 (m, 5H), 4.62 – 4.42 (m, 7H, **H1(β)**, CH_2Ph), 4.14 (t, *J* = 6.3 Hz, 1H), 4.02 (dd, *J* = 10.0, 3.4 Hz, 1H), 3.77 (dd, *J* = 10.0, 3.8 Hz, 1H), 3.72 (t, *J* = 6.3 Hz, 1H), 3.64 (ddd, *J* = 18.3, 9.6, 6.9 Hz, 2H), 3.56 (ddd, *J* = 9.5, 5.0, 1.4 Hz, 2H), 3.46 (s, 1H), 3.45 (s, 1H), 2.09 (s, 3H), 2.04 (s, 3H) ppm.

^{13}C NMR (151 MHz, $CDCl_3$) δ 170.4, 170.3, 138.5, 138.2, 137.8, 137.7, 137.6, 137.2, 137.0, 128.6, 128.5, 128.4, 128.4, 128.4, 128.3, 128.3, 128.3, 128.2, 128.1, 128.0, 128.0, 127.9, 127.9, 127.8, 127.8, 127.7, 127.7, 127.5, 127.0, 102.6 (**C1(β)**), 96.1 (**C1(α)**), 79.3, 78.9, 77.2, 77.0, 76.8, 76.4, 75.5, 75.4, 73.7, 73.6, 73.3, 72.2, 72.1, 71.2, 69.2, 68.5, 68.2, 68.2, 67.9, 66.9, 65.4, 29.7, 21.0, 20.9 ppm.

HRMS (ESI): Calcd for $C_{36}H_{38}O_7Na$ $[M + Na]^+$ 605.2515; found 605.2466.

NP-HPLC of **4Ac-A4** (ELSD trace, t*R* (α) = 7.9 min, t*R* (β) = 8.8 min):

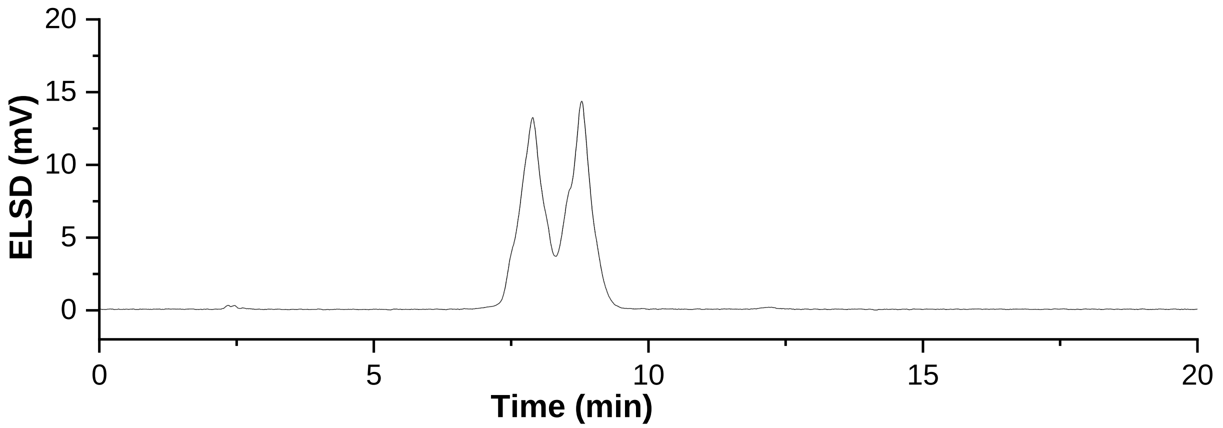

^{1}H NMR (600 MHz, $CDCl_3$) of **4Ac-A4**:

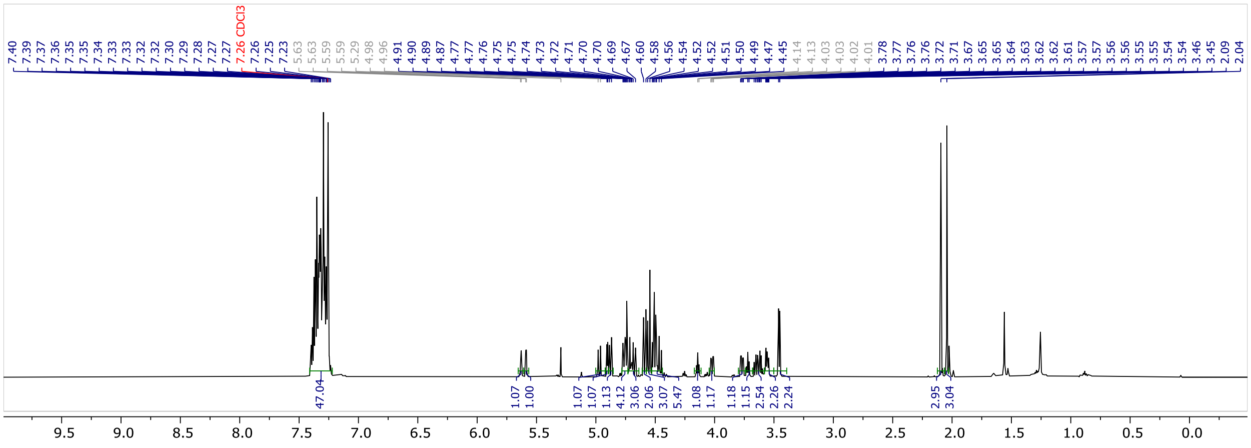

^{13}C NMR (151 MHz, $CDCl_3$) of **4Ac-A4**:

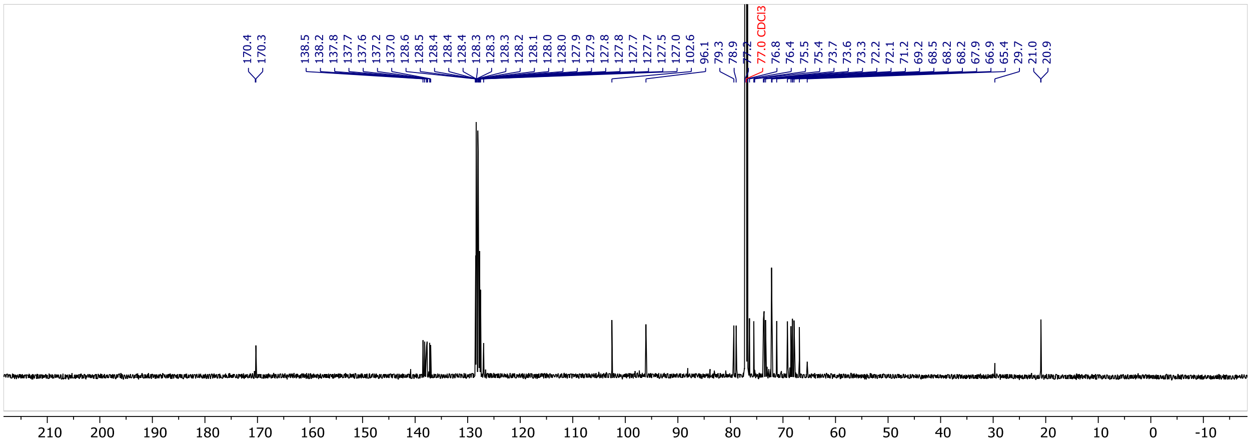

$^{13}C,^{1}H$ HSQC of **4Ac-A4**:

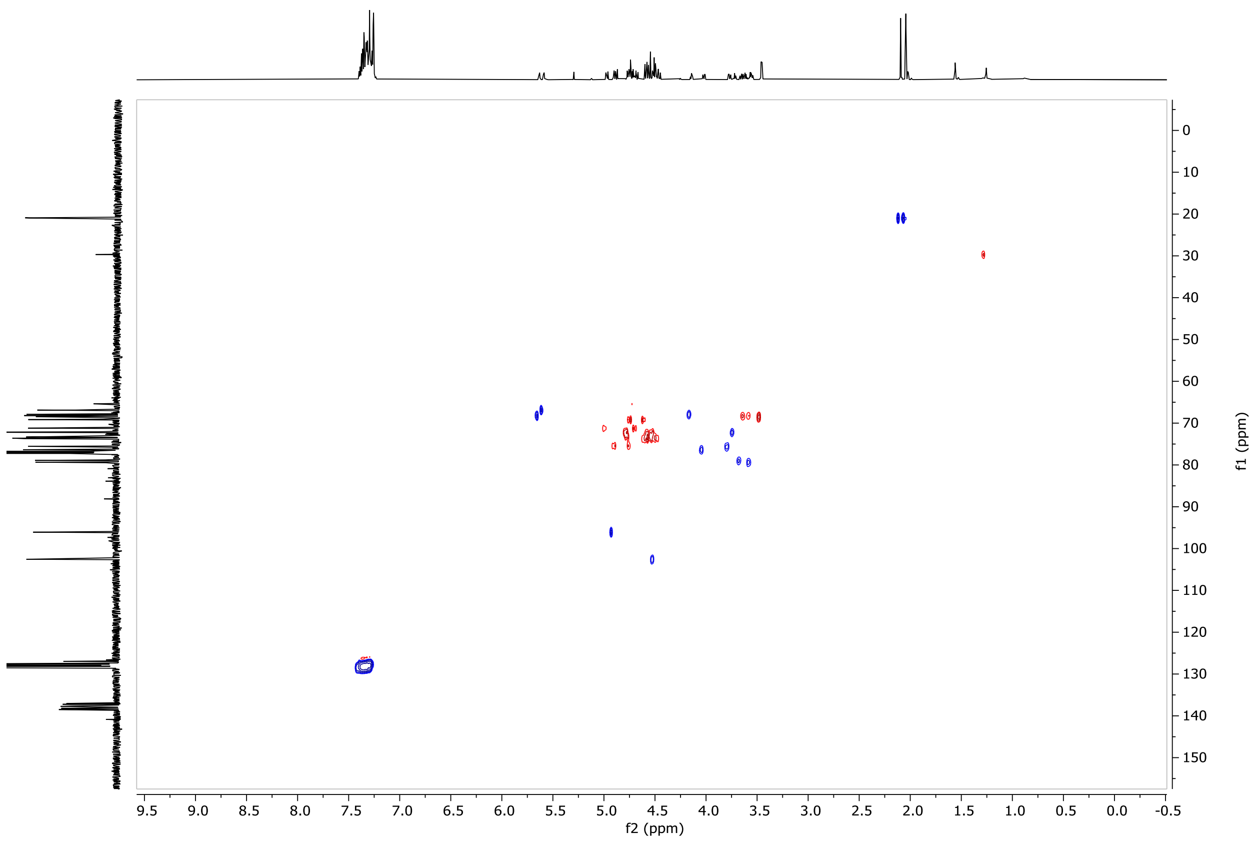

7 Automated Glycan Assembly of Building Block 1

α-(1→3)-D-Trigalactopyranoside (3)

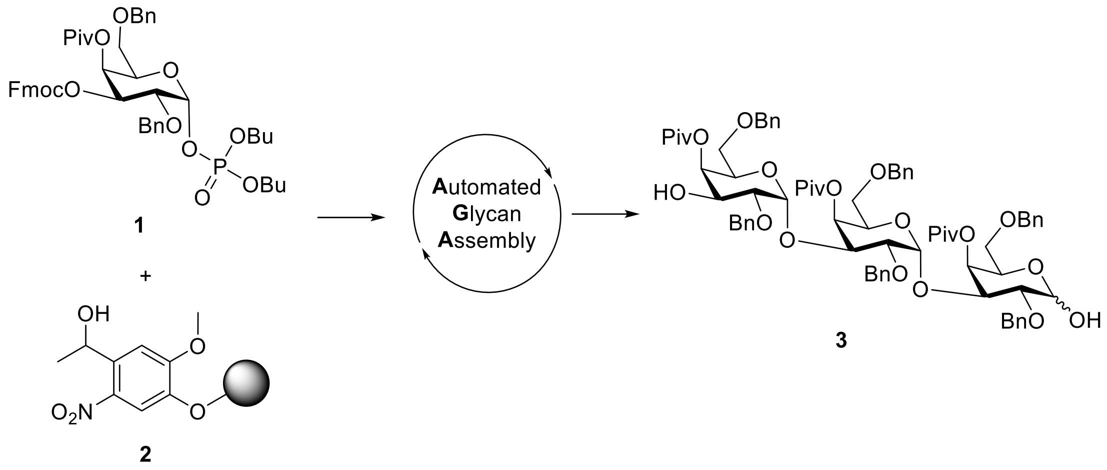

Repeat	Building Blocks	Modules	Notes	
		**I** – Acidic Wash		
	**1**	**IIb** – Glycosylation with	-35 °C (T_1)	5min (t_1)
**2x**	(2 x 4.7 equiv.)	glycosyl phosphate	-20 °C (T_2)	50 min (t_2)
		**III** – Capping		
		**IVc** – Fmoc Deprotection		
		**I** – Acidic Wash		
	**1**	**IIb** – Glycosylation with	-35 °C (T_1)	5min (t_1)
**1x**	(9.4 equiv.)	glycosyl phosphate – 2 cycles	-20 °C (T_2)	50 min (t_2)
		**III** – Capping		
		**IVc** – Fmoc Deprotection		

Trisaccharide **3** (12.5 mg, 0.014 mmol, 69%, α-only) was obtained as a colorless oil after photocleavage from solid support following **Method A** and purification by normal-phase HPLC (**Method B-1b**, t_R = 9.1 to 9.8 min).

^{1}H NMR (700 MHz, $CDCl_3$) δ 7.40 – 7.20 (m, 30H), 5.54 (d, *J* = 3.2 Hz, 1H), 5.27 (dd, *J* = 6.6, 3.5 Hz, 3H), 5.23 (d, *J* = 3.4 Hz, 1H), 5.12 (d, *J* = 3.4 Hz, 1H), 4.77 – 4.70 (m, 2H), 4.59 – 4.56 (m, 3H), 4.54 – 4.42 (m, 5H), 4.38 (s, 1H), 4.36 – 4.32 (m, 4H), 4.30 – 4.25 (m, 3H), 3.91 (dd, *J* = 10.1, 3.5 Hz, 1H), 3.84 (dd, *J* = 10.3, 3.4 Hz, 1H), 3.78 (dd, *J* = 10.1, 3.5 Hz, 1H), 3.57 – 3.26 (m, 6H), 1.15 (s, 9H), 1.09 (s, 9H), 1.02 (s, 9H) ppm.

^{13}**C NMR** (176 MHz, $CDCl_3$) δ 178.1, 177.4, 177.3, 138.3, 138.2, 138.1, 138.0, 137.9, 137.6, 128.7, 128.6, 128.5, 128.5, 128.5, 128.4, 128.3, 128.0, 128.0, 127.9, 127.7, 127.5, 93.1, 92.9, 91.9, 75.3, 74.7, 73.7, 73.2, 73.0, 72.7, 72.3, 70.5, 70.1, 69.8, 69.0, 68.6, 68.5, 68.3, 68.1, 68.0, 67.5, 66.8, 66.3, 39.2, 39.1, 39.0, 27.3, 27.2, 27.2 ppm.

HRMS (QToF): Calcd for $C_{75}H_{92}O_{19}Na$ $[M + Na]^+$ 1319.6125; found 1319.6166.

Crude NP-HPLC of **3** (ELSD trace):

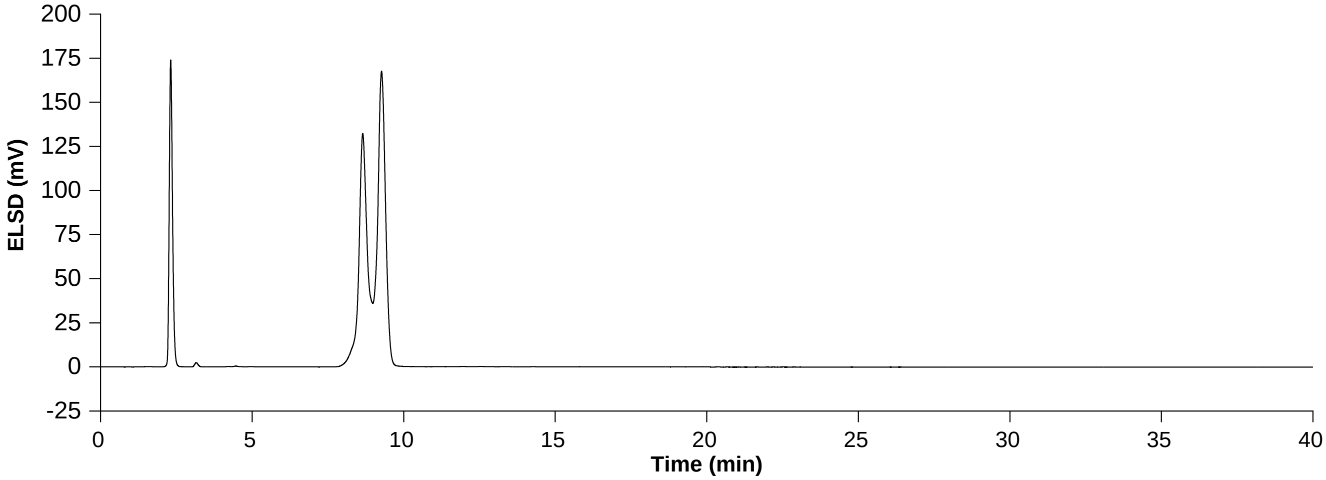

Purified NP-HPLC of **3** (ELSD trace, t_R (α) = 9.1 min, t_R (β) = 9.8 min):

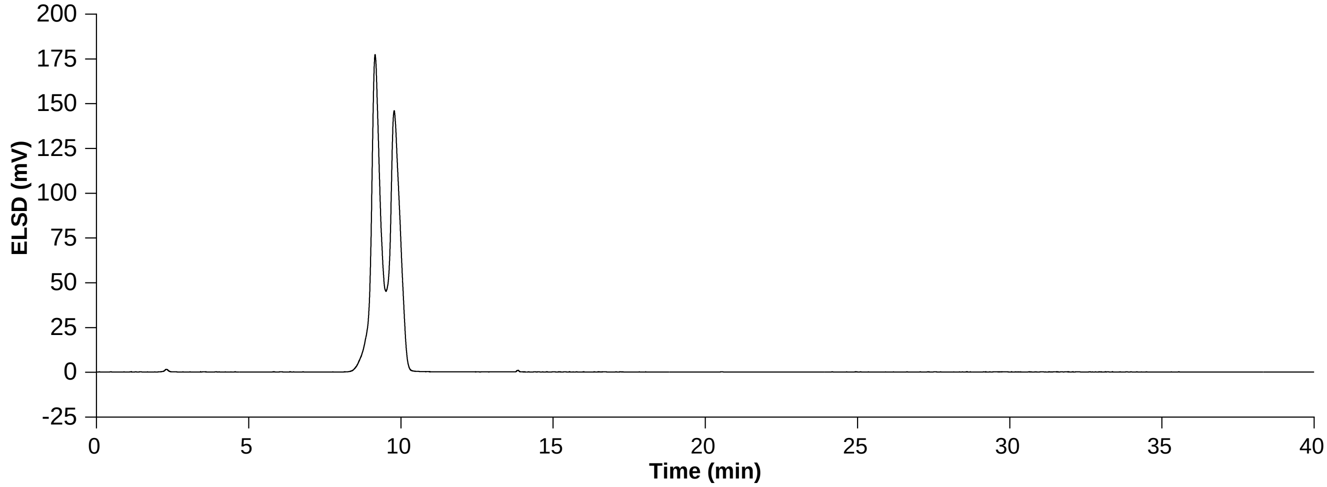

^{1}H NMR (700 MHz, $CDCl_3$) of **3 (mutarotates)**:

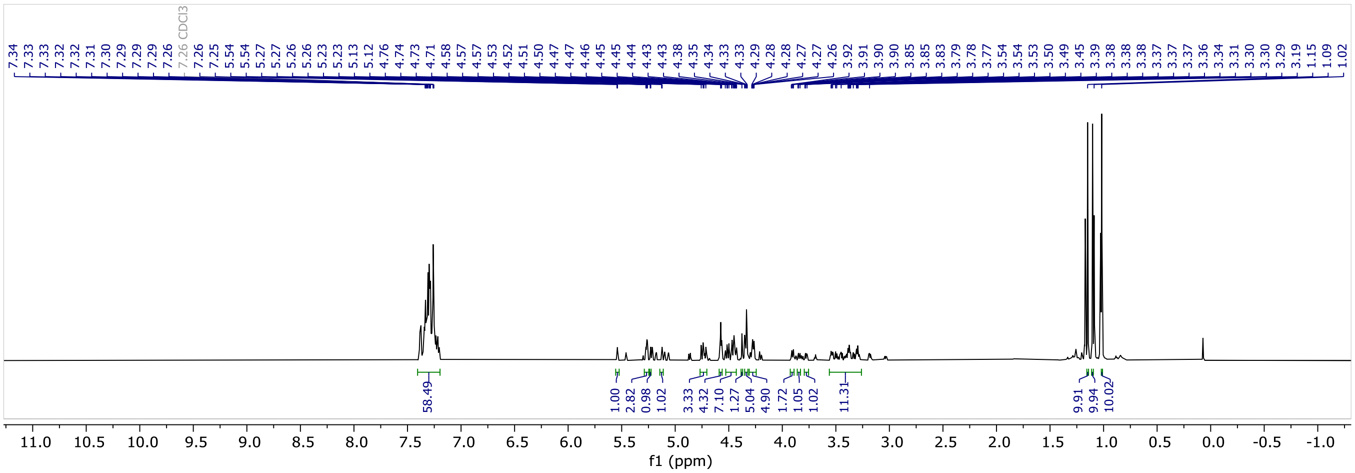

^{13}C NMR (176 MHz, $CDCl_3$) of **3 (mutarotates)**:

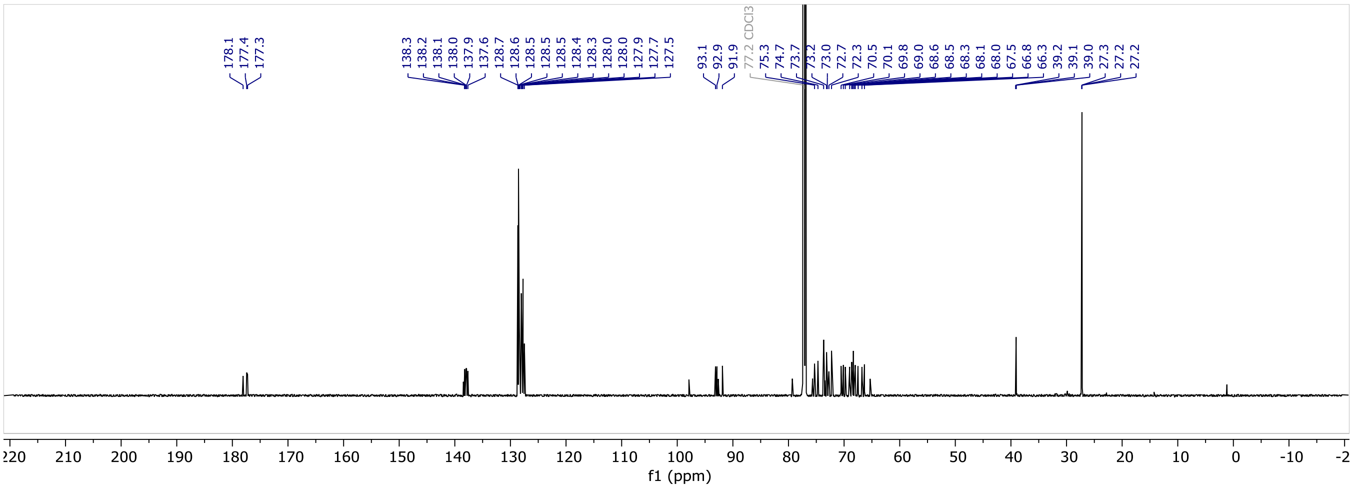

$^{13}C,^{1}H$ HSQC of **3**:

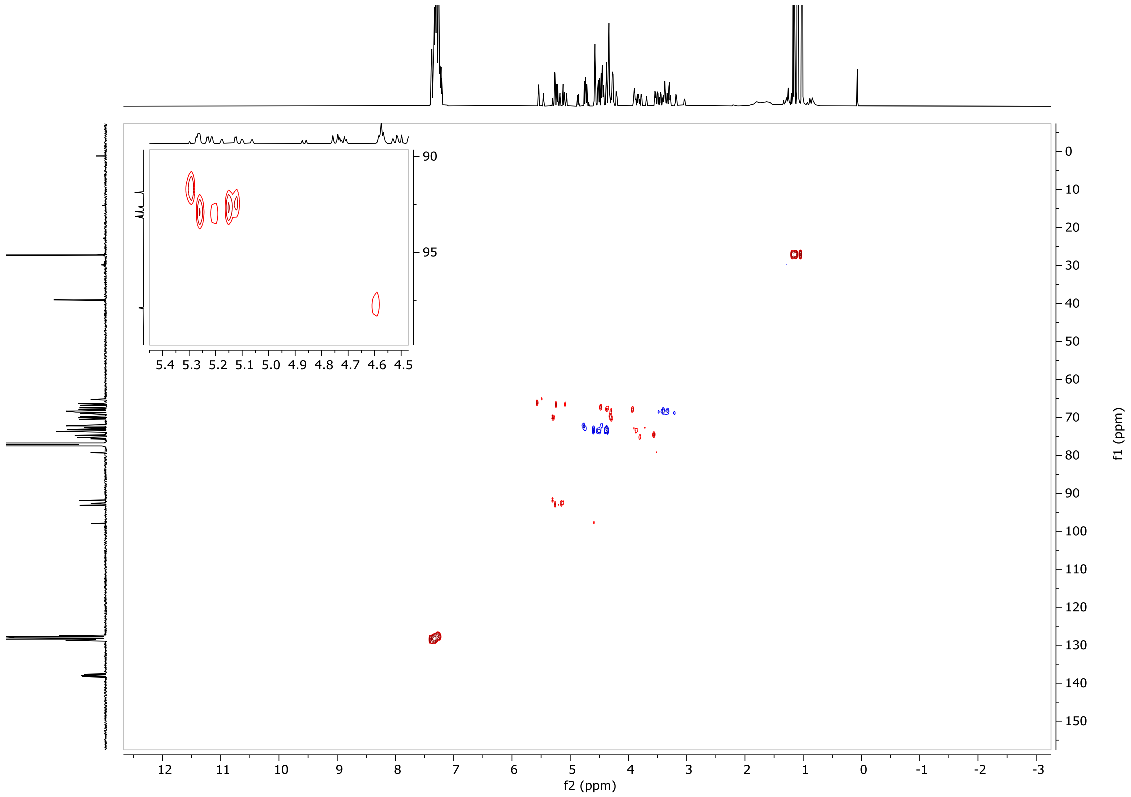

Coupled $^{13}C,^{1}H$ HSQC of **3**:

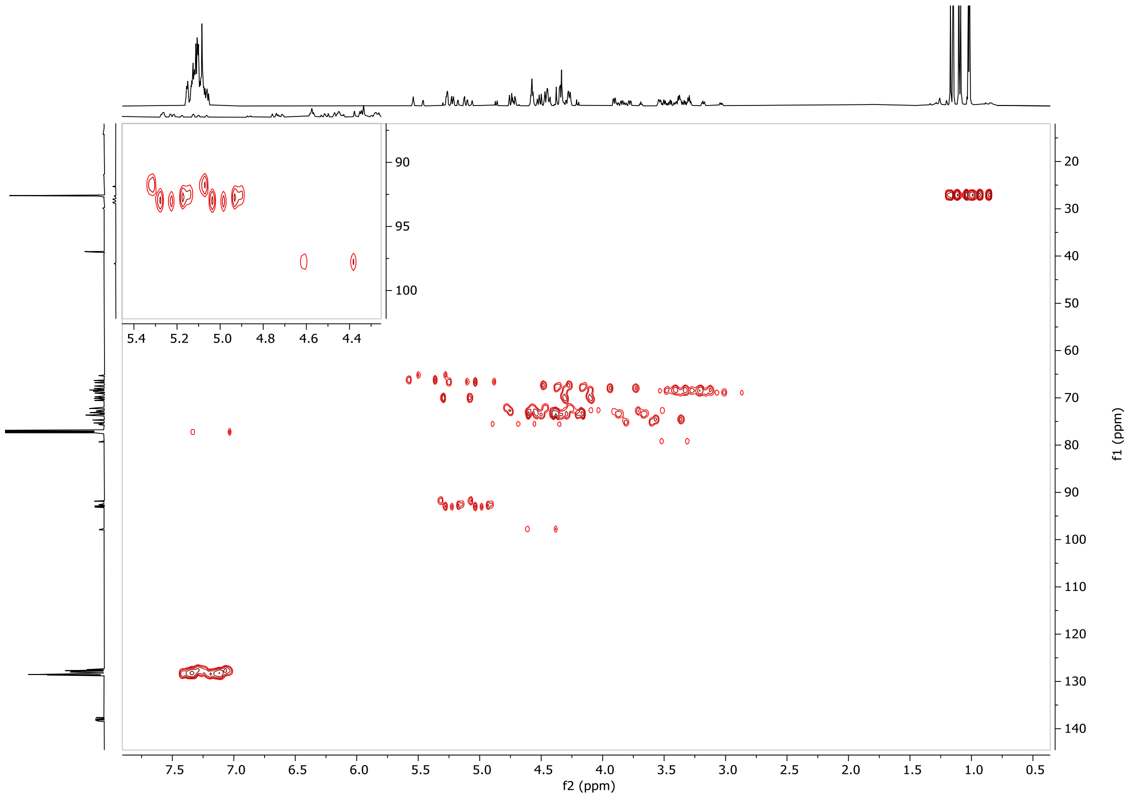

8 Literature

1. Schöllkopf, W.; Gewinner, S.; Junkes, H.; Paarmann, A.; von Helden, G.; Bluem, H. P.; Todd, A. M. M., The new IR and THz FEL facility at the Fritz Haber Institute in Berlin. *Proc. SPIE Int. Soc. Opt. Eng.* **2015,** *9512*, 95121L.

2. Supady, A.; Blum, V.; Baldauf, C., First-Principles Molecular Structure Search with a Genetic Algorithm. *J. Chem. Inf. Model.* **2015,** *55* (11), 2338-48.

3. Blum, V.; Gehrke, R.; Hanke, F.; Havu, P.; Havu, V.; Ren, X.; Reuter, K.; Scheffler, M., Ab initio molecular simulations with numeric atom-centered orbitals. *Comput. Phys. Commun.* **2009,** *180* (11), 2175-2196.

4. Perdew, J. P.; Burke, K.; Ernzerhof, M., Generalized Gradient Approximation Made Simple. *Phys. Rev. Lett.* **1996,** *77* (18), 3865-3868.

5. Tkatchenko, A.; Scheffler, M., Accurate molecular van der Waals interactions from ground-state electron density and free-atom reference data. *Phys. Rev. Lett.* **2009,** *102* (7), 073005.

6. Adamo, C.; Barone, V., Toward reliable density functional methods without adjustable parameters: The PBE0 model. *J. Chem. Phys.* **1999,** *110* (13), 6158-6170.

7. Grimme, S.; Antony, J.; Ehrlich, S.; Krieg, H., A consistent and accurate ab initio parametrization of density functional dispersion correction (DFT-D) for the 94 elements H-Pu. *J. Chem. Phys.* **2010,** *132* (15), 154104.

8. Hehre, W. J.; Ditchfield, R.; Pople, J. A., Self—Consistent Molecular Orbital Methods. XII. Further Extensions of Gaussian—Type Basis Sets for Use in Molecular Orbital Studies of Organic Molecules. *J. Chem. Phys.* **1972,** *56* (5), 2257-2261.

9. Frisch, M. J.; Trucks, G. W.; Schlegel, H. B.; Scuseria, G. E.; Robb, M. A.; Cheeseman, J. R.; Scalmani, G.; Barone, V.; Petersson, G. A.; Nakatsuji, H.; Li, X.; Caricato, M.; Marenich, A. V.; Bloino, J.;

Janesko, B. G.; Gomperts, R.; Mennucci, B.; Hratchian, H. P.; Ortiz, J. V.; Izmaylov, A. F.; Sonnenberg, J. L.; Williams; Ding, F.; Lipparini, F.; Egidi, F.; Goings, J.; Peng, B.; Petrone, A.; Henderson, T.; Ranasinghe, D.; Zakrzewski, V. G.; Gao, J.; Rega, N.; Zheng, G.; Liang, W.; Hada, M.; Ehara, M.; Toyota, K.; Fukuda, R.; Hasegawa, J.; Ishida, M.; Nakajima, T.; Honda, Y.; Kitao, O.; Nakai, H.; Vreven, T.; Throssell, K.; Montgomery Jr., J. A.; Peralta, J. E.; Ogliaro, F.; Bearpark, M. J.; Heyd, J. J.; Brothers, E. N.; Kudin, K. N.; Staroverov, V. N.; Keith, T. A.; Kobayashi, R.; Normand, J.; Raghavachari, K.; Rendell, A. P.; Burant, J. C.; Iyengar, S. S.; Tomasi, J.; Cossi, M.; Millam, J. M.; Klene, M.; Adamo, C.; Cammi, R.; Ochterski, J. W.; Martin, R. L.; Morokuma, K.; Farkas, O.; Foresman, J. B.; Fox, D. J. *Gaussian 16 Rev. A.03*, Wallingford, CT, 2016.

10. Riplinger, C.; Sandhoefer, B.; Hansen, A.; Neese, F., Natural triple excitations in local coupled cluster calculations with pair natural orbitals. *J. Chem. Phys.* **2013,** *139* (13), 134101.

11. Weigend, F.; Ahlrichs, R., Balanced basis sets of split valence, triple zeta valence and quadruple zeta valence quality for H to Rn: Design and assessment of accuracy. *Phys. Chem. Chem. Phys.* **2005,** *7* (18), 3297-305.

12. Neese, F., Software update: The ORCA program system—Version 5.0. *WIREs Comput. Mol. Sci.* **2022**.

13. Le Mai Hoang, K.; Pardo-Vargas, A.; Zhu, Y.; Yu, Y.; Loria, M.; Delbianco, M.; Seeberger, P. H., Traceless Photolabile Linker Expedites the Chemical Synthesis of Complex Oligosaccharides by Automated Glycan Assembly. *Journal of the American Chemical Society* **2019,** *141* (22), 9079-9086.

14. Gude, M.; Ryf, J.; White, P. D., An accurate method for the quantitation of Fmoc-derivatized solid phase supports. *Lett. Pept. Sci.e* **2002,** *9* (4-5), 203-206.

15. Hahm, H. S.; Hurevich, M.; Seeberger, P. H., Automated assembly of oligosaccharides containing multiple cis-glycosidic linkages. *Nat. Commun.* **2016,** *7*, 12482.

16. Chatterjee, S.; Moon, S.; Hentschel, F.; Gilmore, K.; Seeberger, P. H., An Empirical Understanding of the Glycosylation Reaction. *J. Am. Chem. Soc.* **2018,** *140* (38), 11942-11953.

17. Alex, C.; Visansirikul, S.; Demchenko, A. V., A versatile approach to the synthesis of mannosamine glycosides. *Org. Biomol. Chem.* **2020,** *18* (34), 6682-6695.

18. Scanlan, E. M.; Mackeen, M. M.; Wormald, M. R.; Davis, B. G., Synthesis and Solution-Phase Conformation of the RG-I Fragment of the Plant Polysaccharide Pectin Reveals a Modification-Modulated Assembly Mechanism. *J. Am. Chem. Soc.* **2010,** *132* (21), 7238-7239.

19. Daragics, K.; Fügedi, P., Regio- and chemoselective reductive cleavage of 4,6-O-benzylidene-type acetals of hexopyranosides using BH3·THF–TMSOTf. *Tetrahedron Lett.* **2009,** *50* (24), 2914-2916.

20. Sherman, A. A.; Mironov, Y. V.; Yudina, O. N.; Nifantiev, N. E., The presence of water improves reductive openings of benzylidene acetals with trimethylaminoborane and aluminium chloride. *Carbohydr. Res.* **2003,** *338* (8), 697-703.

21. Tian, G.; Hu, J.; Qin, C.; Li, L.; Zou, X.; Cai, J.; Seeberger, P. H.; Yin, J., Chemical Synthesis and Immunological Evaluation of Helicobacter pylori Serotype O6 Tridecasaccharide O-Antigen Containing a dd-Heptoglycan. *Angew. Chem. Int. Ed.* **2020,** *59* (32), 13362-13370.

22. Hofmann, J.; Hahm, H. S.; Seeberger, P. H.; Pagel, K., Identification of carbohydrate anomers using ion mobility–mass spectrometry. *Nature* **2015,** *526* (7572), 241-244.
